# Highly selective acylation of polyamines and aminoglycosides by 5-acyl-5-phenyl-1,5-dihydro-4*H*-pyrazol-4-ones†
†Electronic supplementary information (ESI) available: Experimental procedures, kinetic data, UV/Vis spectra, NMR spectra, and HPLC traces. X-ray data for **44**. CCDC 1563600. For ESI and crystallographic data in CIF or other electronic format see DOI: 10.1039/c7sc03184j



**DOI:** 10.1039/c7sc03184j

**Published:** 2017-08-30

**Authors:** Kostiantyn O. Marichev, Estevan C. Garcia, Kartick C. Bhowmick, Daniel J. Wherritt, Hadi Arman, Michael P. Doyle

**Affiliations:** a Department of Chemistry , The University of Texas at San Antonio , San Antonio , Texas 78249 , USA . Email: michael.doyle@utsa.edu

## Abstract

5-Acyl-5-phenyl-1,5-dihydro-4*H*-pyrazol-4-ones, accessible from arylpropargyl phenyldiazoacetates, are highly selective acyl transfer reagents for di- and polyamines, as well as aminoalcohols and aminothiols. As reagents with a carbon-based leaving group, they have been applied for benzoyl transfer with a broad selection of substrates containing aliphatic amino in combination with other competing nucleophilic functional groups. The substrate scope and levels of selectivity for direct benzoyl transfer exceed those of known benzoylating reagents. With exceptional selectivity for acylation between primary amines bound to primary and secondary carbons, these new reagents have been used in direct site-selective monobenzoylation of aminoglycoside antibiotics.

## Abstract 2

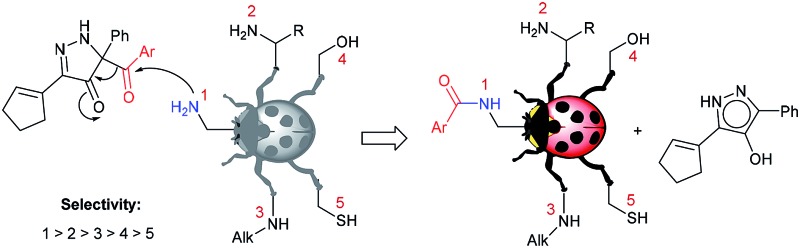
A highly selective acylating reagent with remarkable recognition of primary amines in monoacylation of polyamines and aminoglycosides.

## Introduction

The formation of an amide bond by acyl transfer is a classic chemical reaction^1^ that has been extensively studied^2^ and widely applied.^3^ Over the years numerous acyl transfer agents have been investigated; their activities have been dependent on the leaving group, which has ranged from those of the highly reactive acyl chlorides and anhydrides to those that are conjugate bases of relatively weak acids. The linkage to the acyl group has included *N*-imidazolyl^4a–g^ and *N*-benzotriazolyl,^4h–m^ succinimidyloxy,^5^ cyanide,^6^ sulfonamide,^7^ trichloromethyl,^8^ enolates,^9^ and, most recently, boron trifluoride.^10^ Selectivity in acyl transfer has become an important goal with achievements evolving beyond chemoselection (OH *vs.* SH *vs.* NH acylation)^11^ to selective acylation of primary amines over secondary amines.^10,11^ Although the use of enzymes^12^ or RNA-based aptameric protective groups^13^ for highly regio- and site-selective acylation of di- and polyamines has been reported, small molecule acyl transfer reagents that provide high to exclusive regiocontrol remain under-investigated.

Moderate to high,^10,14^ and even exclusive,^10a,14d,e^ selectivities in the acylation of the primary over the secondary amine in aliphatic diamines have been reported. Secondary amines are more basic, but they are also more sterically encumbered and, thereby, subject to steric restrictions. Although high selectivity has been reported for acylation of the amino group at the 1-position of 1,2-diaminopropane,^14*d*^ selective acylation of primary amines whose carbon attachment is primary, secondary or tertiary, has received scant attention.^15^

**Scheme 1:**
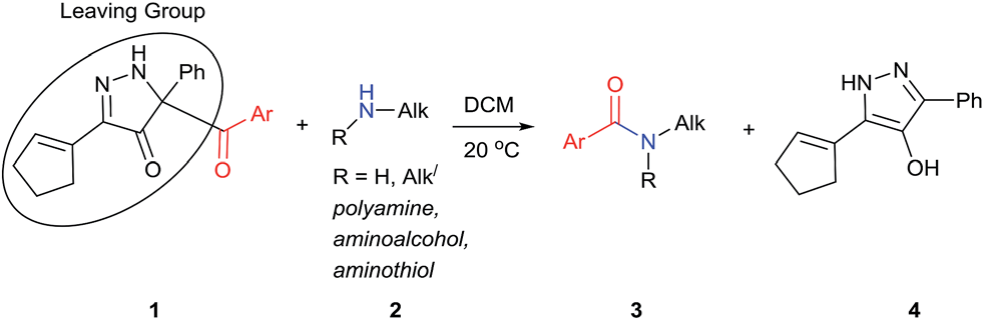
Acyl transfer from **1** to **2**.

We have recently prepared a novel heterocyclic compound that appeared to have the potential of being a selective benzoyl transfer reagent. This compound, 5-benzoyl-3-(cyclopent-1-en-1-yl)-5-phenyl-1,5-dihydro-4*H*-pyrazol-4-one (BCPP, **1a**), which is formed in one step from a propargyl phenyldiazoacetate by gold(i) catalysis,^16^ has its benzoyl group attached to carbon-5 of a pyrazolone ring so that the conjugate base of hydroxypyrazole is the leaving group (Scheme 1). Acyl transfer reagents that, like BCPP, have carbon-linked leaving groups, principally cyano,^6,14a^ trichloromethyl,^8^ 2-imidazolyl,^11f,17^ among a few others,^14d,18^ generally exhibit higher chemoselectivities than their heteroatom-linked counterparts. However, to our knowledge, acylating agents whose leaving group undergoes aromatization upon acyl transfer (transformation of **1** to **4**, Scheme 1) have not been reported.

## Results and discussion

**Scheme 2:**
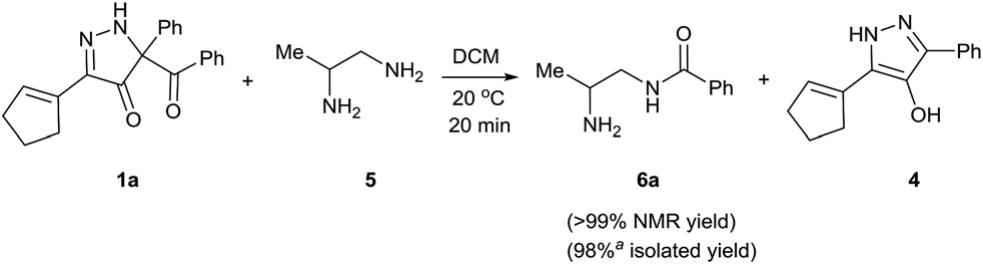
Site-selective benzoylation of 1,2-propanediamine (**5**) by BCPP. ^a^Average yield obtained from two parallel runs (±1%).

Having established that BCPP undergoes quantitative stoichiometric benzoylation of simple aliphatic amines (benzyl- and cyclohexyl-amines)^16^ at room temperature in dichloromethane, we investigated the benzoylation of 1,2-diaminopropane (**5**) by BCPP to determine its selectivity for benzoylation of the primary amino group attached to either a primary or secondary carbon atom (Scheme 2). The addition of BCPP to **5** resulted in the formation of a single regioisomer **6a** (^1^H NMR analysis) in 98% isolated yield along with 4-hydroxypyrazole **4**. Neither the 2-benzoylamido-1-aminopropane (**6b**) nor the dibenzoylated 1,2-propanediamine were detected. For a more precise analysis, however, the reaction mixture was subjected to HPLC analysis using as standards a mixture of mono- and dibenzoylated compounds formed from unselective benzoylation of **5** by benzoyl chloride. HPLC analysis of the reaction mixture from the BCPP reaction detected a minor amount of **6b** and showed the ratio **6a**/**6b** to be 98.5 : 1.5 (average of two experiments, ±0.3%); dibenzoylated 1,2-diaminopropane was not detected. Previously, Lewis acid activated *N*-acyl-2-oxazolidinone gave benzoylated products in 83% yield with a **6a**/**6b**/dibenzoylation ratio determined by ^1^H NMR of 85 : 7 : 8,^15^ and with an α-aryl-β-ketoester benzoyl transfer agent 100% selectivity for the formation of **6a** was reported by ^1^H NMR analysis but was not confirmed by HPLC analysis.^14*d*^

With the high regioselectivity established in the benzoylation of 1,2-diaminopropane, other examples that would illustrate the selectivity of BCPP were chosen. The results provided in Table 1 illustrate the substrate scope of selective benzoylation for aminoalcohols, aminothiols, and di- and polyamines by reactions with stoichiometric amounts of BCPP at room temperature in dichloromethane. (*R*)-2-Amino-2-phenylethan-1-ol (**7**) containing both primary amino and hydroxyl groups reacts with BCPP exclusively at the amino functionality and affords amide **8** in quantitative yield. In contrast, direct acylation of **7** by benzoyl chloride provides **8** in 68–85% yields along with the diacylated product.^19^ 2-(Piperidin-2-yl)ethan-1-ol (**9**), as an example with the combination of secondary amino and primary hydroxyl groups, affords only *N*-benzoylated product **10** from the reaction with BCPP in 95% isolated yield, whereas benzoyl chloride forms **10** in only 82% yield;^20^ however, the reaction of **9** with BCPP is sluggish, and complete transfer occurs only after 12 h.

**Table 1:** Substrate scope for selective acylation of aminoalcohols, aminothiols, di- and polyamines by BCPP (**1a**)

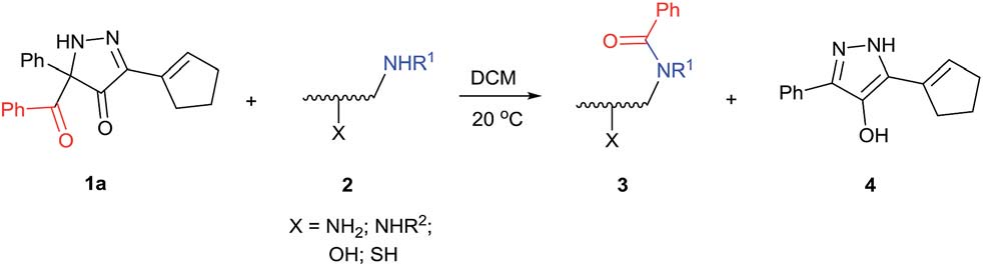				
Entry^*a*^	Reactant	Product	NMR yield^*b*^ (%)	Isolated yield^*c*^ (%)
1	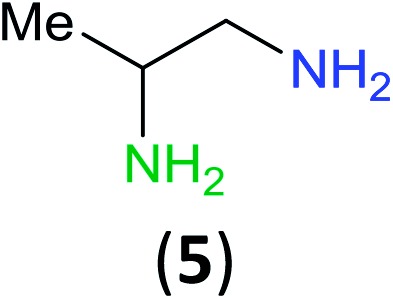	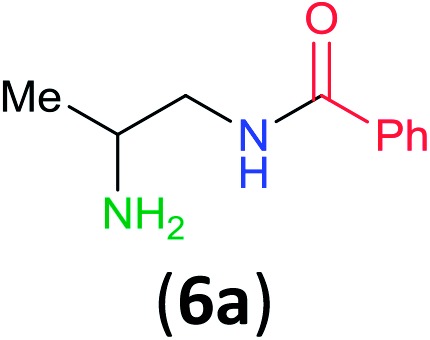	>99	98
2	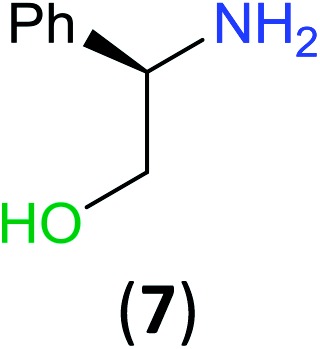	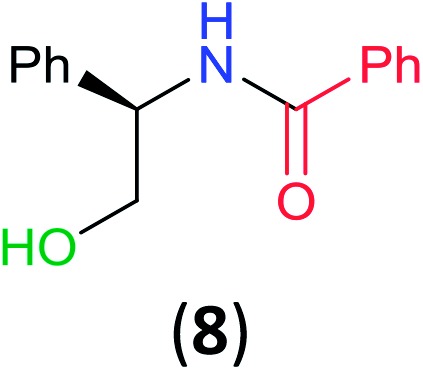	>99	>99
3^*d*^	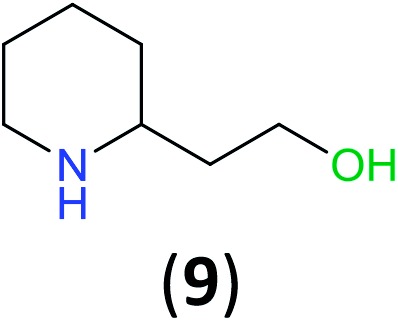	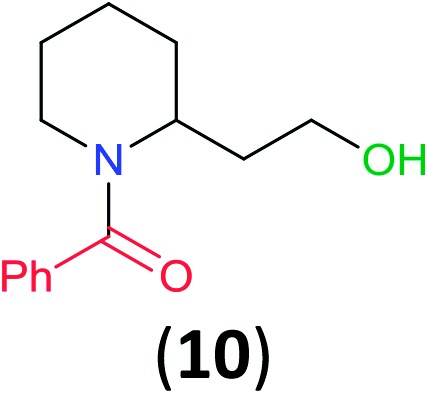	97	95
4	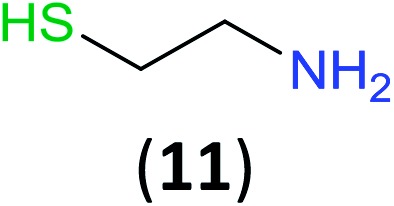	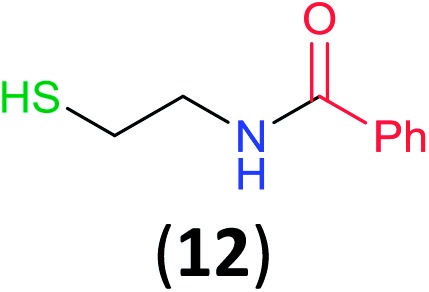	>99	97
5^*e*^	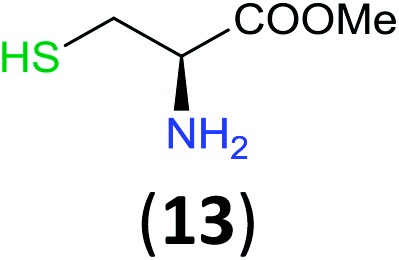	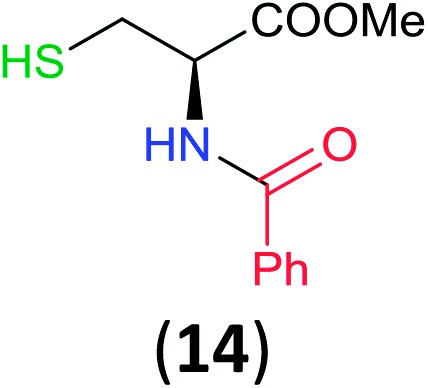	99	93
6	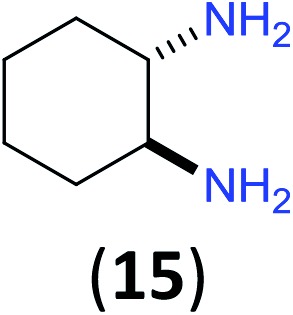	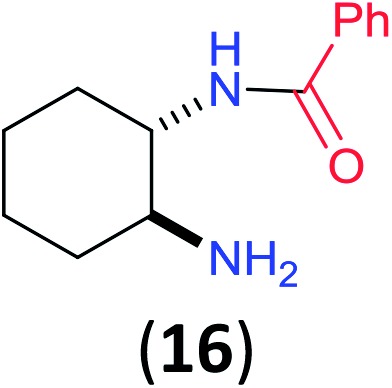	>99	98
7	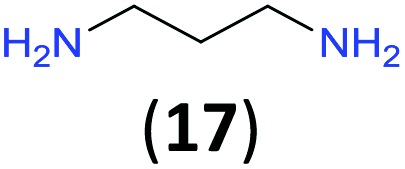	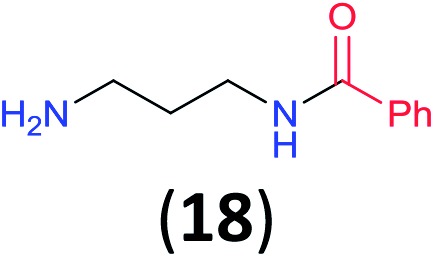	>99	98
8	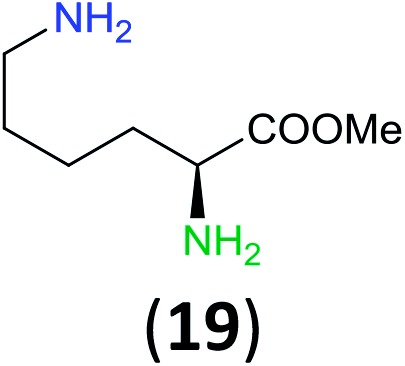	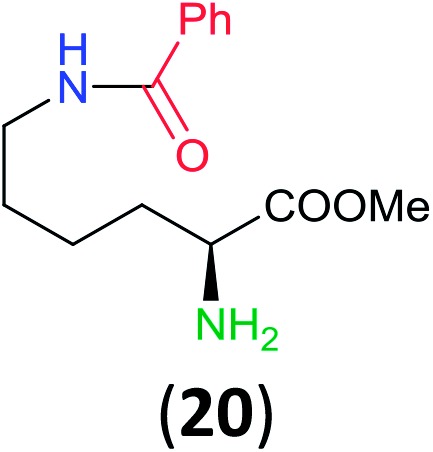	>99	94
9	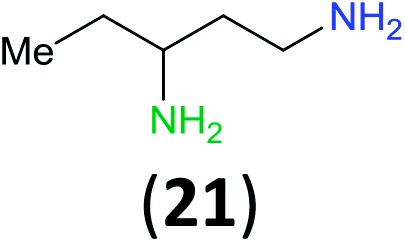	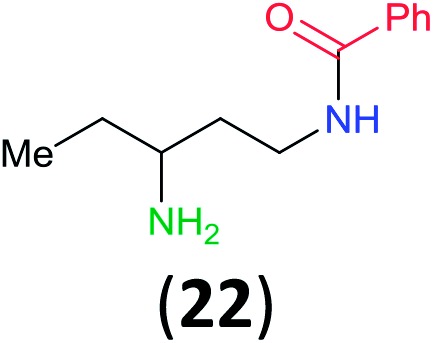	>99	>99
10^*f*^	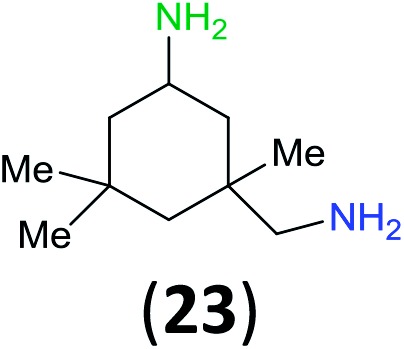	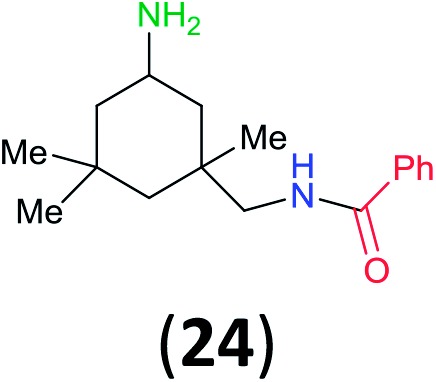	>99	97
11	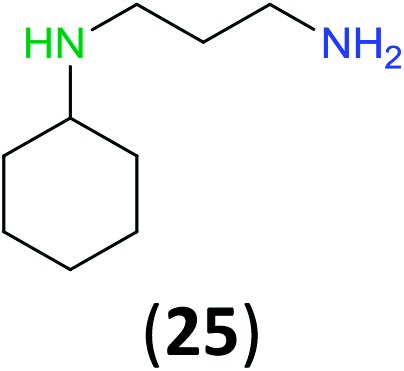	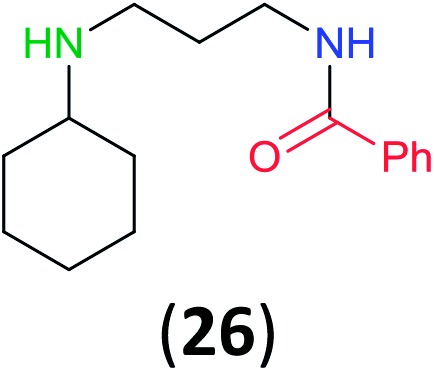	>99	>99
12^*g*^	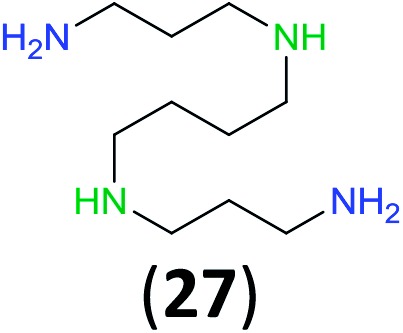	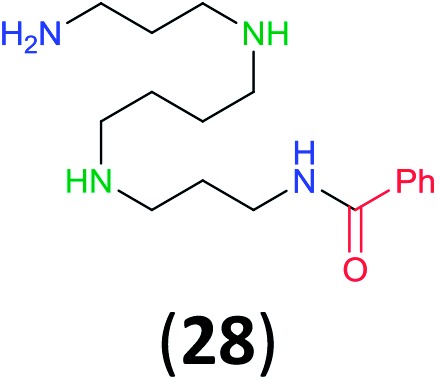	98	93
13^*h*^	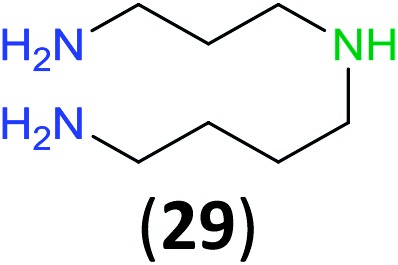	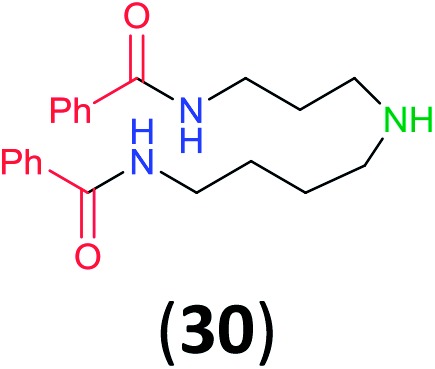	99	98
14^*i*^	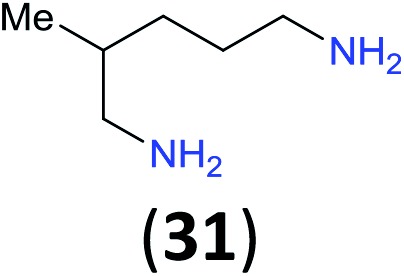	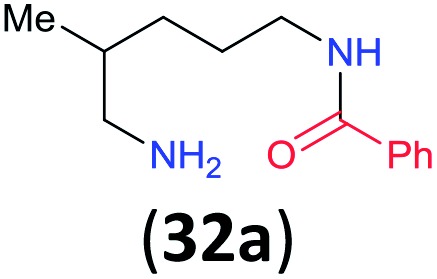	99	95
		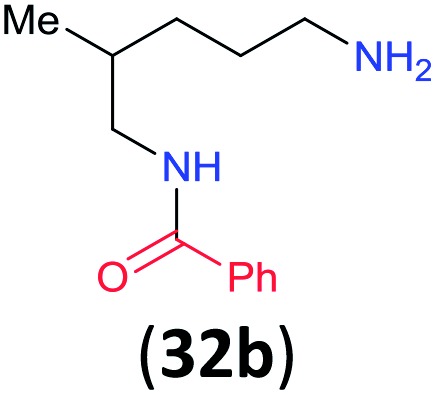		**32a**/**32b** 86 : 14

^*a*^Reactions were carried out on a 1.0 mmol scale: to a stirred solution of amine in 8 mL DCM a solution of acylating reagent **1a** in 10 mL DCM was added dropwise over 20 min at 20 °C. The resulting reaction mixture was allowed to stir at 20 °C for 30 min.

^*b*^Yields were determined by ^1^H NMR analysis of reaction mixtures with 1,3,5-trimethoxybenzene as the internal standard.

^*c*^Yields calculated from the mass of chromatographed products as average of two runs with a deviation of ±1%.

^*d*^Reaction time was 12 h.

^*e*^Reaction time was 4 h.

^*f*^A commercial mixture of *cis*- and *trans*-isomers was used (*trans*/*cis* = 85 : 15).

^*g*^C18-reversed phase silica gel was used to purify the product.

^*h*^Two equivalents of **1a** were used to avoid the formation of a mixture of two monoacylation products; reaction time 24 h.

^*i*^Ratio **32a**/**32b** = 86 : 14 determined by ^1^H NMR as an average of two runs (±0.6%).

Like aminoalcohols, aminothiols **11** and **13** react with BCPP selectively, giving only the products from benzoylation of the amino group (97% yield for **12**, and 92% isolated yield for **14** without evidence of any benzoylation of the sulfhydryl group). A previous report described the formation of **12** in 93% yield using benzoyl chloride, but only 67% of **14** was obtained when benzoic anhydride was used.^11*d*^ Only monobenzoylation of *trans*-1,2-diaminocyclohexane (**15**) or 1,3-diaminopropane (**17**) occurred with BCPP (both obtained in 98% isolated yield); monoamide **16** was obtained previously in 90% yield (95 : 5 monobenzoylation : dibenzoylation), and **18** was obtained in 80% yield with a ratio **18** to dibenzoylated product of 83 : 17 using complexation with 9-BBN to supress dibenzoylation.^21^ Exclusive benzoylation of the primary amine bonded to a primary carbon occurred in compounds **19**, **21**, and **23**, where there was a choice with benzoylation of the primary amine bonded to a secondary carbon. Highly selective direct benzoylation of l-lysine methyl ester (**19**) at the terminal amino group formed **20** in 94% isolated yield. Scaling the reaction up to 1 g of **19**·2HCl afforded **20** as the only product, which was isolated in 95% yield. Two additional substrates with primary amino groups attached to both primary and secondary carbons have been examined: selective benzoylation of 1,3-diaminopentane (**21**) by BCPP produced monoamide **22** in quantitative yield, and this is similar to the reported^15^ benzoylation of **21** by *N*-benzoyl-2-oxazolidinone catalysed by group IV-metals (98% yield of **3g**); benzoylation of commercial isophorone diamine (**23**) by BCPP afforded monoamide **24** in 97% isolated yield. For di- and polyamines with selection between primary and secondary amines, benzoylation with BCPP occurred exclusively at the primary amine. Indirect benzoylation of **25** by benzoyl chloride using TMS-protection-deprotection was reported to give **26** in 94% yield,^22^ and the dibenzoylation of spermidine **29** was previously reported to have occurred in 92% yield using two equivalents of benzoyl cyanide (with HCN generation).^14*a*^ Finally, in an extension of regioselectivity to a choice between two primary amines that are both bonded to primary carbons, 2,5-diamino-2-methylpentane (**31**) underwent BCPP benzoylation at the 5-position with an 86 : 14 monobenzoylation selectivity.

We previously reported that BCPP undergoes slow benzoylation of methanol only at 65 °C so that after 12 h methyl benzoate is obtained in 63% yield.^16^ Accordingly, we examined the reactivity of BCPP with other nucleophiles. Benzoyl transfer from **1a** to benzyl mercaptan and aniline does not occur in dichloromethane at 20 °C; reactions at 80 °C for 24 h in 1,2-dichloroethane afforded *S*-benzyl benzothioate and *N*-benzoylaniline, but only in 22% and 11% yield, respectively, the remainder being unreacted starting materials. Obviously, the basicity of the nucleophile is an important factor in determining reactivity in reactions of **1a** with nucleophiles.

**Fig. 1:**
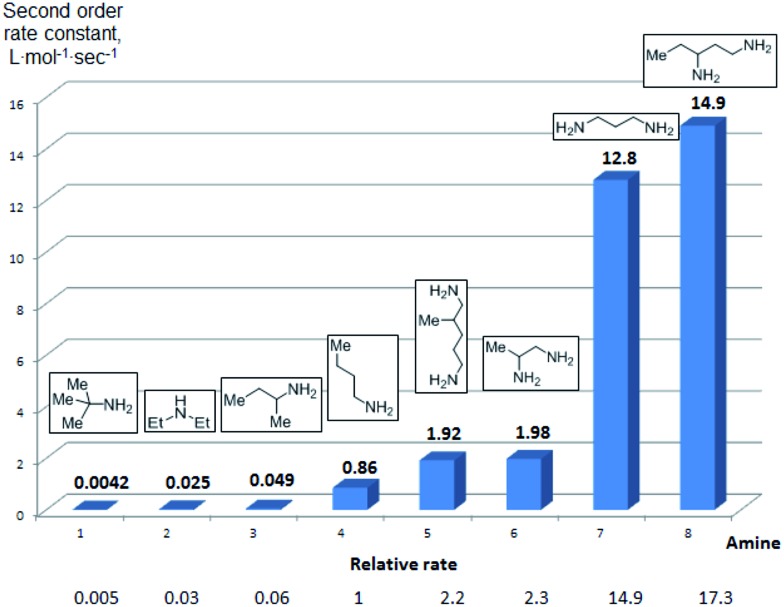
Second-order rate constants for the reaction of BCPP with mono- and diamines in DCM at 24 °C.

To further assess the selectivity observed for benzoyl transfer from BCPP, the kinetic rate law and rate constants were determined for a series of structurally different aliphatic mono- and diamines by UV/Vis-spectroscopy. The reactions were carried out under pseudo-first-order conditions in dichloromethane at 24 °C and were determined to be second order reactions from reactions performed for each amine with five different amine:BCPP ratios using standard kinetic methods.^23,24^ Fig. 1 provides a comparison of second order rate constants obtained for the reaction of BCPP with 2-amino-2-methylpropane, diethylamine, 2-aminobutane, 1-aminobutane, 2,5-diamino-2-methylpentane, 1,2-diaminopropane, 1,3-diaminopropane, and 1,3-diaminopentane. Their rate constants indicate that steric factors are the major determinants of selectivity in benzoyl transfer from BCPP; there is no correlation with nucleophilicity or basicity of the amine. Among the monoamines the fastest reaction rate was observed for 1-aminobutane (*k* = 0.86 ± 0.04 L mol^–1^ s^–1^) as the least sterically hindered amine; the introduction of an α-methyl substituent into the aliphatic chain (2-aminobutane) decreases the reaction rate by a factor of 18 (*k* = 0.049 ± 0.002 L mol^–1^ s^–1^). The presence of two methyl substituents in the α-position of a primary amine (2-amino-2-methylpropane) makes the reaction about 200 times slower (*k* = 0.0042 ± 0.0002 L mol^–1^ s^–1^) than that for 1-aminobutane. Although diethylamine is the most nucleophilic amine in the series of monoamines tested, its rate constant for reaction with BCPP (*k* = 0.025 ± 0.001 L mol^–1^ s^–1^) is 34 times slower than that for 1-aminobutane, and two times slower than that for 2-aminobutane.

Aliphatic diamines are more reactive than monoamines. While 1,2-diaminopropane (**5**) (*k* = 1.98 ± 0.05 L mol^–1^ s^–1^) and 2,5-diamino-2-methylpentane (**31**) (*k* = 1.92 ± 0.04 L mol^–1^ s^–1^) react with BCPP about two times faster than 1-aminobutane, the reaction rate constants with 1,3-diaminopropane (*k* = 12.8 ± 0.5 L mol^–1^ s^–1^) and 1,3-diaminopentane (*k* = 14.9 ± 0.3 L mol^–1^ s^–1^) are 15–17 times greater. These results are in accord with the second amino group serving as a base to remove a proton from nitrogen in the process of acyl transfer.

**Fig. 2:**
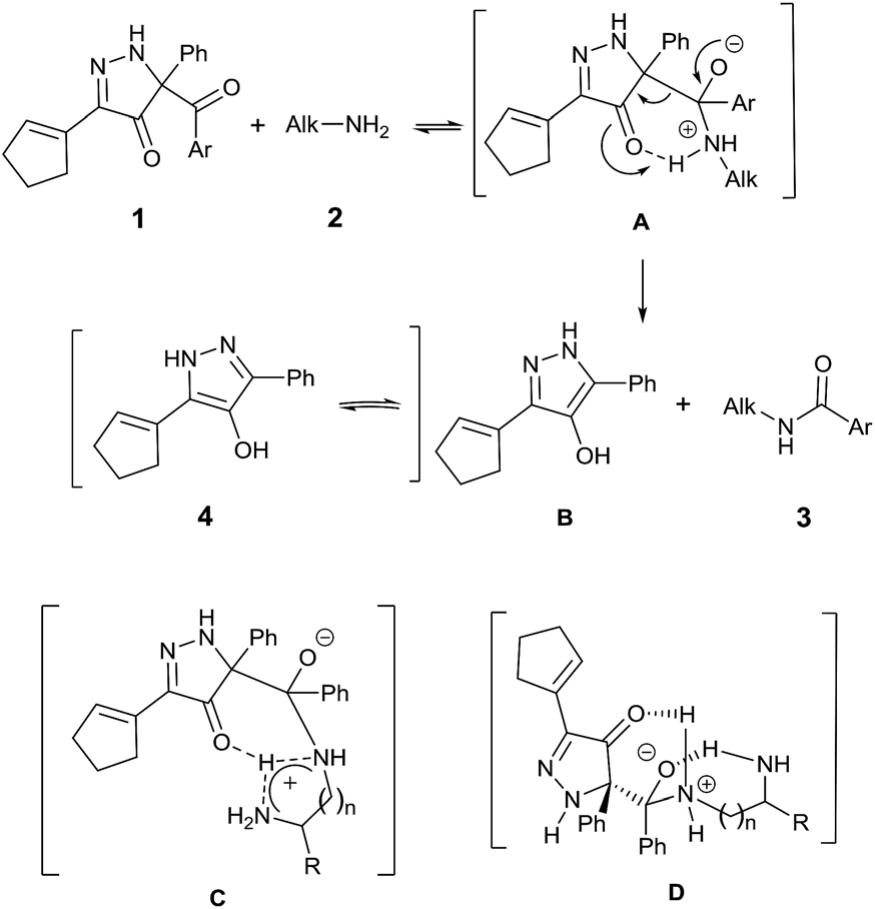
Plausible mechanism for the reaction of BCPP with aliphatic mono- and diamines.

Consistent with established mechanisms for acyl transfer,^25^ BCPP reacts with monoamine **2** to form the classic tetrahedral zwitterionic intermediate **A**, which then undergoes intramolecular proton transfer to the Lewis basic carbonyl oxygen concurrent with C–C bond cleavage yielding amide **3** and 4-hydroxypyrazole **4** (Fig. 2).

This mechanism fits the model that describes initial reversible formation of **A** followed by rate-determining product formation.^26^

Reactions of BCPP with 1,(*n* + 1)-diamines add another possible dimension to this mechanism of reaction with intramolecular proton transfer to the second amine (**C**) and/or intramolecular stabilization of the tetrahedral intermediate (**D**) to account for their dramatic changes in reaction rate. The large rate increases observed with 1,3-diaminopropane and 1,3-diaminopentane are consistent with intramolecular stabilization of **D**.

**Fig. 3:**
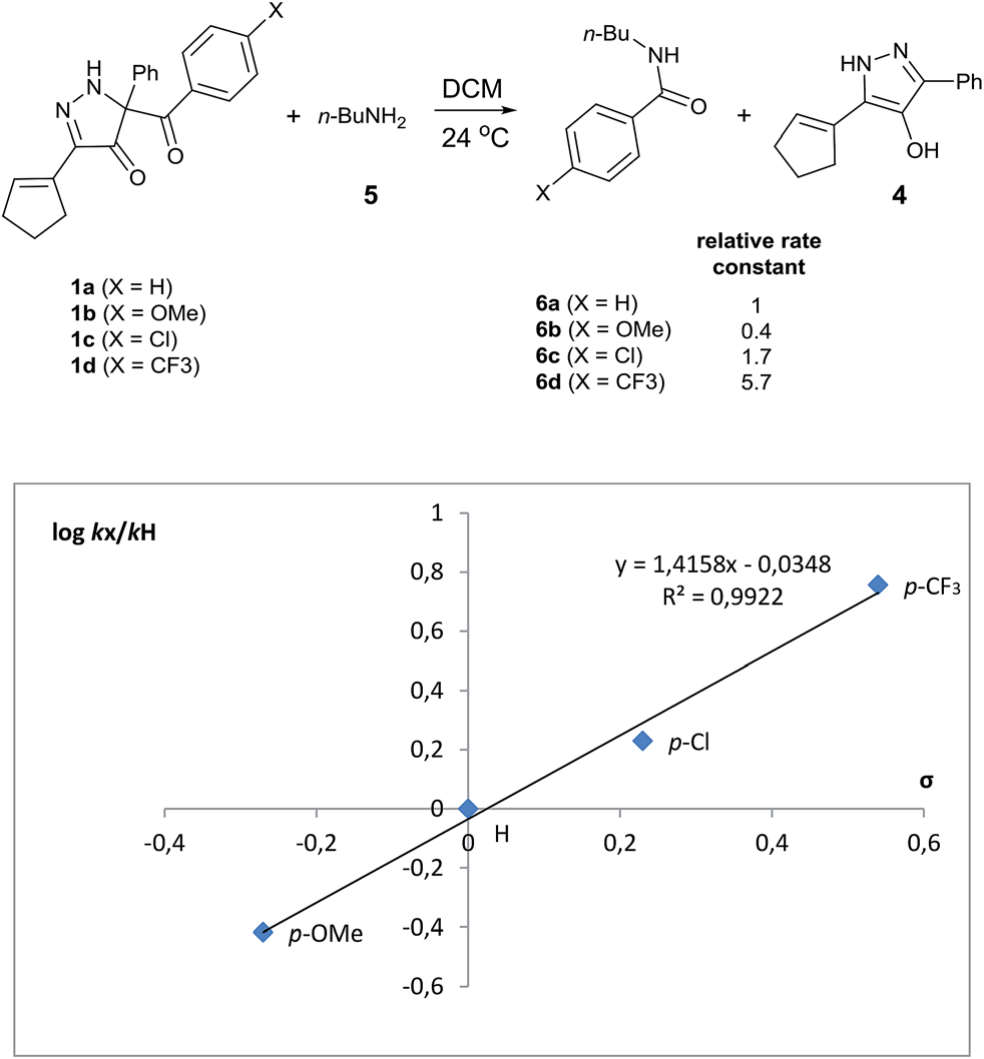
Hammett plot for the reaction of *para*-substituted acyl transfer reagents **1a–d** with 1-aminobutane in DCM at 24 °C.

Evaluation of kinetic substituent effects for benzoyl transfer was made with 5-aroyl-3-(cyclopent-1-en-1-yl)-5-phenyl-1,5-dihydro-4*H*-pyrazol-4-ones having different substituents in the *para*-position of the aromatic ring for reactions with 1-aminobutane. Second order rate constants for these reactions with three *para*-substituted acyl transfer reagents **1a–d** have been determined. Electron-withdrawing substituents increased the rate of reaction, whereas electron-donating substituents decreased the rate of reaction. A Hammett plot using *σ*-values gave *ρ* = +1.42 (Fig. 3).

Site selective *O*-acylation of amphotericin by *para*-substituted benzoylpyridinium chlorides was reported to occur with *ρ* = +1.69, and a calculated *ρ* = –0.395 projected the kinetic selectivity of acyl transfer from the corresponding acylpyridinium complexes to one hydroxyl group *versus* the four others in the same molecule (site selectivity = 48% with *p*-Cl and 64% with *p*-OMe).^27^ However, substituent effects by the BCPP acylating reagent do not have a significant influence on selectivity between two primary amino groups. As reported in Table 1, 2,5-diamino-2-methylpentane (**31**) underwent BCPP benzoylation at the 5-position with 86 : 14 selectivity.

Although kinetic studies showed significant differences in rates for a series of *para*-substituents in the benzoyl group of **1** (Fig. 3), the ratios **32a**/**32b** were similar: 88 : 12 (±0.6%) for **31** with the MeO-substituent (yield **32a** + **32b** 92%) and 85 : 15 (±0.4%) for **31** with the Cl-substituent (yield **32a** + **32b** 90%).

Selective acylation of aminoglycosides as free bases has been particularly challenging.^28^ Previous methods for the preparation of monoacyl derivatives have required either protecting groups or the acidification of the amino group(s) in aminoglycosides.^28,29^ Moreover, those methods afforded pure products in only 20–40% isolated yields. Although modern methods for acylation of 6′-position of kanamycin, tobramycin and amikacin as sulfate salts using *N*-hydroxysuccinymyl (NHS) esters have been reported^30^ with yields up to 91%, the scope of this methodology is limited to substituted acetyl derivatives. Selective introduction of acetyl groups into aminoglycosides using chemoenzymatic methods^31^ or RNA-based aptameric groups^13^ have been also reported. However, to our knowledge, there have not been reports of direct selective benzoylation of aminoglycoside antibiotics. Reported direct benzoylations of amikacin by 4-vinylbenzoylating reagents having chloride, *p*-nitrophenoxy, and *O*-(*N*-succinimidyl) leaving groups were not selective, giving a mixture of three products.^32^

**Scheme 3:**
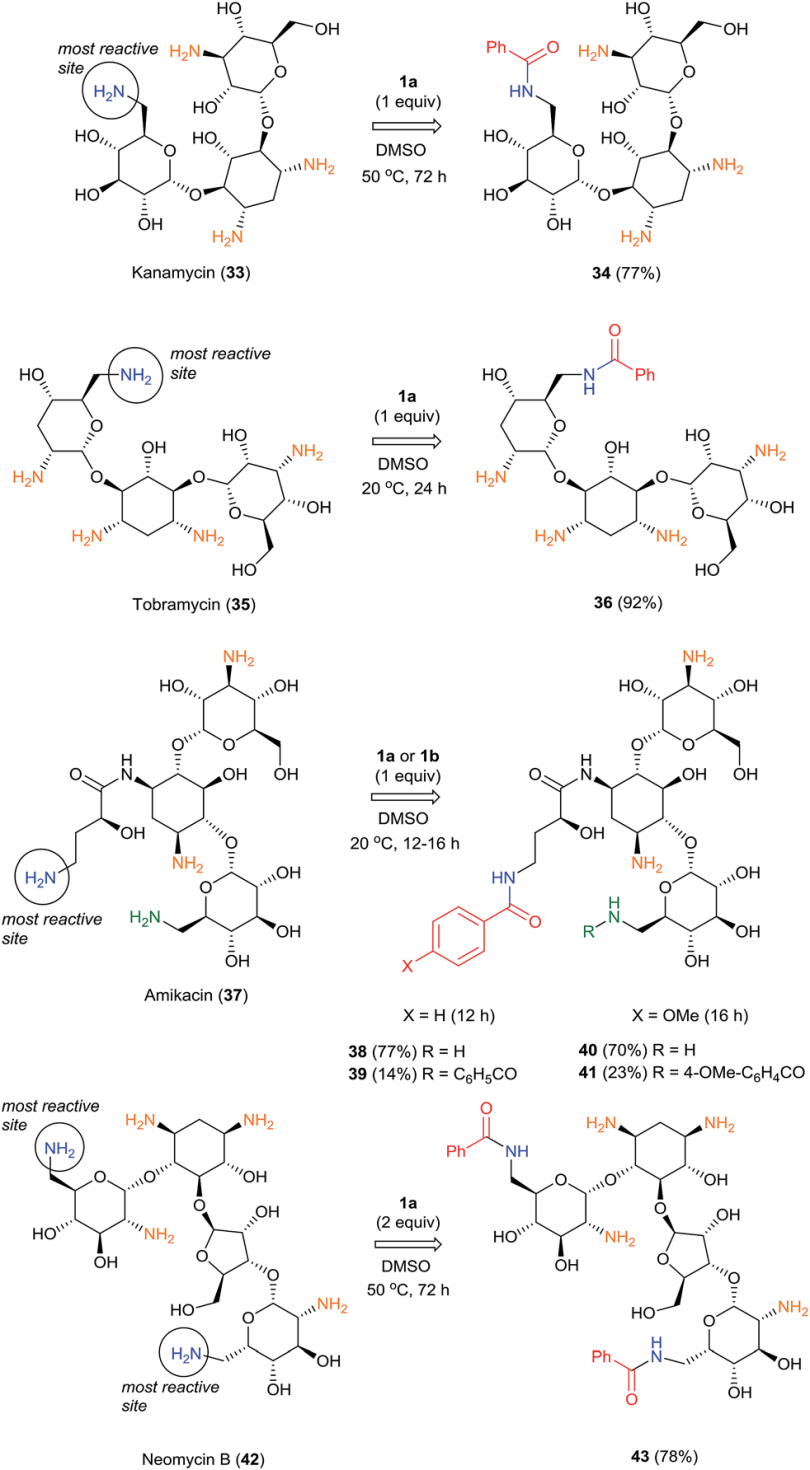
Site-selective benzoylation of aminoglycoside antibiotics by BCPP.

The highly selective reactions of kanamycin (**33**), tobramycin (**35**) and amikacin (**37**) as free bases with one equivalent of BCPP have been successfully performed in dimethylsulfoxide solutions (Scheme 3).

While the reactions with **35** and **37** occur at room temperature for 12–24 h, full conversion of **33** was observed only after 72 h at 50 °C, apparently because of low solubility of **33** in DMSO. Monobenzoylated **34** and **36** were obtained as single regioisomers in 77% and 92% isolated yields, respectively. The more complex and challenging molecule of amikacin (**37**) has two amino groups attached to primary carbon atoms. However, the γ-amino-β-hydroxybutyryl group (AHB) is much more reactive with BCPP than is the competitive aminomethylglycoside. As the result, with 1.0 equiv. of BCPP **37** affords monobenzoyl derivative **38** as a single monobenzoyl regioisomer in 77% isolated yield, however 14% of dibenzoylated product **39** was also isolated. The use of 2.0 equiv. of BCPP with **37** forms only dibenzoylated **39** in 90% isolated yield; however, the reaction goes to completion only after 20 h. To study the substituent effect in the benzoylation of **37**, the less reactive 4-OMe-BCPP **1b** was used. Although the reaction was slower (16 h), selectivity toward monoacylation (70% of mono- **40** and 23% of dibenzoyl product **41**) was lower than with BCPP **1a**. Neomycin B (**42**), which has two amino groups attached to primary carbon atoms that are predicted to have the same reactivity, reacted with 2.0 equiv. of BCPP to afford the dibenzoylated product **43** in 78% isolated yield, but only after 72 h at 50 °C. The same reaction with 1.0 equiv. of BCPP led to an inseparable mixture of two monobenzoylated products and **43**. The structures of all benzoylated aminoglycosides were confirmed by 1D- and 2D-NMR experiments.^24^

**Fig. 4:**
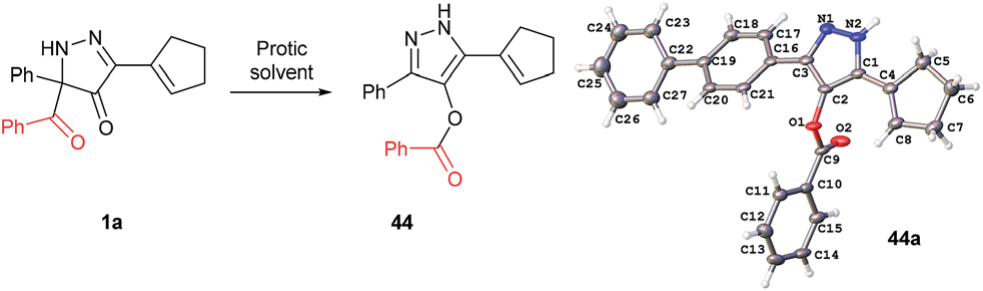
Intramolecular benzoyl transfer of BCPP in protic solvents. X-ray structure of **44a** with 50% thermal ellipsoid probability.

In all benzoylation reactions of aminoglycosides with BCPP unreacted starting materials were the only residue and no benzoylated regioisomers were detected in the reaction mixtures by NMR or HPLC analyses. A reason for incomplete benzoylation is that is decomposition of BCPP, caused by proton transfer, occurs. In protic solvents BCPP undergoes irreversible intramolecular acyl transfer to form aromatic compound **44** (Fig. 4). X-Ray analysis showed that the benzoyl group in **44** is attached to the oxygen atom. The kinetics of the transformation **1a** → **44** gave first order rate constants: *k* = 7.1×10^–5^ ± 0.3×10^–5^ s^–1^ (in MeOH/DCM 5 : 1) and *k* = 2.45×10^–4^ ± 0.05×10^–4^ s^–1^ (in THF/H_2_O 1 : 1).^24^ To confirm that **44** is not an active benzoylating reagent the reaction of 1,2-diaminopropane with 1.0 equiv. of **44** was carried out in DCM at r.t. for 24 h, and there was no reaction occurred.

Selective benzoylation of aminoglycosides by BCPP also occurs in methanol/water or THF/water solvent mixtures. The reactions occur faster (2–3 h); however, they occur with lower product yields caused by the competing reaction of **1a** → **44** (Fig. 4).

Tobramycin (**35**) showed only 12% conversion with BCPP in 1 : 1 THF/water to form mono benzoylated tobramycin (**36**) exclusively in 2 h; amikacin (**37**) underwent 23% conversion in THF/water 1 : 1 to give a mono- to di-benzoylated product ratio **38**/**39** = 7 : 2 (determined by HPLC). Reactions in methanol/water 1 : 1 went to completion in 3 h and gave higher conversions of aminoglycosides: **35** → **36** (42% yield), **37** → **38** + **39** (58% yield, **38**/**39** = 5 : 2 by HPLC). The use of 2.0 equiv. of BCPP in 1 : 1 methanol/water afforded **38** + **39** in 83% yield in 3 h (**38**/**39** = 5 : 2 by HPLC). The reduced percent conversions also indicate that compound **44**, which is formed competitively under these conditions, is not a benzoyl transfer reagent.

## Conclusions

In summary, we have discovered a new, efficient and broadly applicable carbon-based heterocyclic acyl transfer reagent, 5-acyl-5-phenyl-1,5-dihydro-4*H*-pyrazol-4-one (BCPP), that is highly selective for the synthesis of *N*-monobenzoyl (or substituted *N*-monobenzoyl) derivatives of di- and polyamines, as well as aminoalcohols and aminothiols. The BCPP reagent generally achieves levels of selectivity that significantly exceed those of previously reported acylating reagents or provide access to new compounds that are inaccessible by alternative acylation methods. Moreover, 5-acyl-5-phenyl-1,5-dihydro-4*H*-pyrazol-4-ones are rare examples of small molecule acylating reagents that are able to achieve very high levels of selectivity for the monoacylation of polyamines. Kinetic studies of a series of mono- and diamines, as well as the low reactivity of alcohols and thiols with **1a**, are consistent with the selectivities that have been obtained. Site-specific selectivity in direct benzoylation of aminoglycoside antibiotics has been demonstrated.

## Experimental

### General procedure for benzoyl transfer using BCPP (**1a**)

To a stirred solution of an amine **2** (0.50 mmol) in DCM (5 mL) a solution of BCPP (**1a**) (0.50 mmol) in DCM (5 mL) was added at 20 °C *via* syringe pump over 20 min, and the reaction mixture was stirred for 0.5–12 h. A white precipitate of 4-hydroxypyrazole **4** formed during the benzoyl transfer process was filtered, the solvent was evaporated, and the monoamide **3** was purified by flash chromatography on silica gel (with a 20 : 1 gradient of DCM/methanol to pure methanol as eluents), unless specified otherwise. Products were characterized by NMR spectroscopy, and their purity was determined by HPLC and/or NMR analyses.

### Synthetic procedure for the benzoylation of aminoglycosides by BCPP

To a screw-capped 8 mL vial an aminoglycoside (0.2 mmol), BCPP (0.2 mmol), and DMSO (1.5 mL) were added sequentially. The mixture was stirred for 12–24 h at 20 °C (for **35** and **37**) and 72 h at 50 °C (for **34** and **42**). The completion of the reaction was monitored by TLC (1 : 1 hexane/ethyl acetate as eluent, monitoring the conversion of BCPP to **4**). Benzoylated aminoglycosides were purified by column chromatography on silica gel (with a gradient of methanol/28% aqueous ammonium hydroxide 100/0–90/10–80/20 as eluents). Products were characterized by NMR spectroscopy, and their purity was determined by HPLC and/or NMR analyses.

### Kinetic determinations for benzoyl transfer by BCPP^24^

Rate constants for the reaction of BCPP with aliphatic amines were determined using UV/Vis-spectroscopy at 24 °C. Kinetic runs were performed for the reaction of BCPP with a series of amine solutions of different concentrations (*C*_amine_ ≥ 10*C*_BCPP_). Considering that the extinction coefficient determined for BCPP *ε* = 2920 at 380 nm, BCPP solutions were prepared to have an absorbance at 380 nm *A* = 0.4–0.8. The concentrations of amine solutions (*C*_amine_ ≥ 10*C*_BCPP_) were those that allowed reaction times less than 1 h. Second order constants were calculated from the slopes of the linear plot of *k* (pseudo-first order) *vs. C* (amine).

### Kinetic determination for the intramolecular benzoyl transfer of BCPP^24^

A series of BCPP solutions of different concentrations were prepared in 5 : 1 MeOH/DCM or THF/H_2_O, and the reaction course was monitored by UV/Vis-spectroscopy from the consumption of BCPP at 400 nm. First order rate constants were determined from the slopes of the linear plots ln *A vs.* time.

## Conflicts of interest

There are no conflicts to declare.
