# Supplementary material for: Highly selective acylation of polyamines and aminoglycosides by 5-acyl-5-phenyl-1,5-dihydro-4H-pyrazol-4-ones
Source: Chem Sci. 2017 Aug 30;8(10):7152–9. doi: 10.1039/c7sc03184j (PMC5635523; doi:10.1039/c7sc03184j)
Supplement: Supplementary file 1 [file SC-008-C7SC03184J-s001.pdf]

Electronic Supplementary Material (ESI) for Chemical Science.
This journal is © The Royal Society of Chemistry 2017

Supporting Information for

Highly Selective Acylation of Polyamines and Aminoglycosides by 5-Acyl-5-phenyl-1,5-dihydro-4*H*-pyrazol-4-ones

Kostiantyn O. Marichev, Estevan C. Garcia, Kartick C. Bhowmick, Daniel J. Wherritt, Hadi Arman and Michael P. Doyle*

Department of Chemistry, The University of Texas at San Antonio,
San Antonio, Texas 78249, United States

michael.doyle@utsa.edu

Contents

- General Information.......... S2
- A Modified Procedure for the Preparation of BCPP (**1a**).......... S2
- Characterization of Products.......... S3
- Experimental Procedure for the Kinetic Study of the Reaction Between BCPP and Amines.......... S10
- Substituents Effect and Hammett Plot for the Reaction of **1a**–**d** with 1-Aminobutane.......... S26
- Kinetic Determination for the Intramolecular Benzoyl Transfer of BCPP.......... S34
- Crystal Structure Report for **44a** (cd1436a).......... S37
- References.......... S40
- ^{1}H and ^{13}C NMR Spectra of Products.......... S41
- HPLC Traces.......... S88

General Information. All solvents were purified and dried using standard techniques. Thin layer chromatography (TLC) analyses were performed on pre-coated analytical plates Silica Gel 60 F_{254}, and visualized with the use of UV light or iodine stain (I_2 and Silica Gel 60). High-resolution mass spectra (HRMS) were performed on a Bruker microTOF-ESI mass spectrometer. Exact masses were reported for the molecular ions $[M+H]^+$ or $[M+2H]^{2+}$. Melting points were measured uncorrected from an Electro Thermo Mel-Temp DLX 104. Column chromatography was performed on a CombiFlash® Rf 200 purification system using normal phase disposable columns. C18-Reversed phased silica gel was used for column chromatography of highly polar compounds. The purity of monoamides was determined on an Agilent 1260 Infinity Series HPLC instrument using a Phenomenex silica-based column (Lichrosorb 5 Sil 60A 250×4.60 mm). Kinetic experiments were performed on a Cary 5000 UV-Vis spectrometer. NMR spectra were recorded at 300 or 500 MHz (^{1}H NMR) and 76 or 126 MHz (^{13}C NMR). Chemical shifts are reported in ppm using residual $CHCl_3$ (δ 7.26 ppm)/H_2O (δ 1.56 ppm), CH_3OH (δ 3.31 ppm)/H_2O (δ 4.87 ppm), DMSO (δ 2.50 ppm)/H_2O (δ 3.33 ppm), or D_2O (δ 4.79 ppm) for ^{1}H NMR reference, and the central resonance of $CDCl_3$ (δ 77.16 ppm), CD_3OD (δ 49.00 ppm), or DMSO-d_6 (δ 39.52 ppm) for ^{13}C NMR reference. Multiplicities in ^{1}H NMR spectra are reported as: s (singlet); br (broad singlet); d (doublet); t (triplet); q (quartet); p (pentet); dd (doublet of doublets); ddd (doublet of doublet of doublets); dt and td (doublet of triplets or triplet of doublets); dq (doublet of quartets); tt (triplet of triplets); m (multiplet); comp (composite of magnetically nonequivalent protons). The number of protons (n) for a given resonance is reported as nH; coupling constants (*J*) are given in Hertz (Hz).

Materials. $AuCl(C_4H_8S)$ was purchased from Strem Chemicals. C18-Reversed phased silica gel was purchased from Sigma-Aldrich. BCPP (**1a**) and analogues **1b**−**d** were synthesised from arylpropargyl phenyldiazoacetates and $AuCl(C_4H_8S)$[1] using a modified procedure. BCPP can be stored at 2−8 °C over three months without any decomposition. All other chemicals were obtained from commercial sources and used as received without further purification.

A Modified Procedure for the Preparation of BCPP (1a)

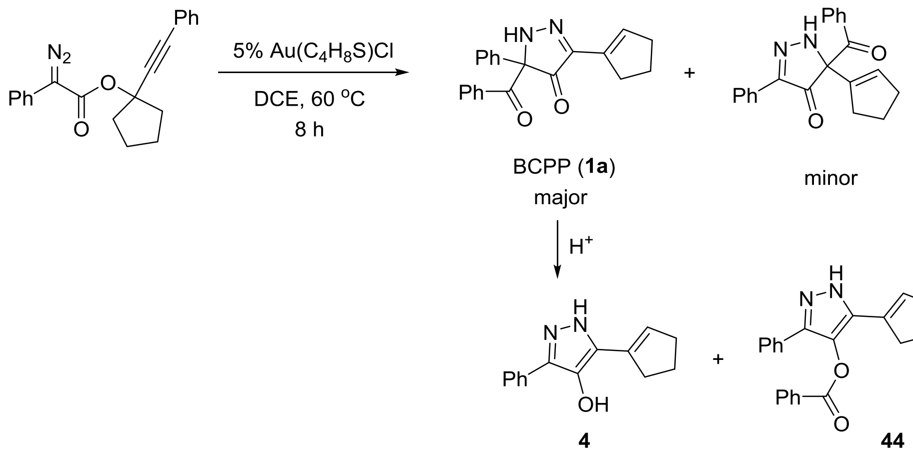

To a solution of 1-(phenylethynyl)cyclopentyl 2-diazo-2-phenylacetate (1.5 g, 4.54 mmol) in 50 mL of 1,2-dichloroethane (DCE) chloro(tetrahydrothiophene)gold(I) $AuCl(C_4H_8S)$ (73 mg, 0.227 mmol) was added in one portion, the reaction flask was degassed and filled with nitrogen, and

the reaction solution was stirred at 60 °C for 8h. Solvent was evaporated until its volume was ca. 5 mL, and the product mixture was purified by flash chromatography on silica gel (with a 20:1 to 4:1 gradient of hexane:ethyl acetate as eluent) to afford BCPP (**1a**) as a light yellow solid (825 mg, 55% yield). Characterization data are in accordance with those in the literature.[1] Note: BCPP undergoes decomposition on silica gel to form **4** and **44**, therefore column chromatography must be performed rapidly.

Characterization of Products

N-(2-Aminopropyl)benzamide (6a)

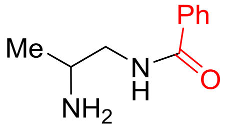

6a

Reported compound.[2] Purified by flash chromatography on silica gel (with a 10:1 to 1:1 gradient of DCM:methanol as eluent): 87 mg, 98% yield; sticky pale yellow oil. ^{1}H NMR (500 MHz, $CDCl_3$) δ 7.77 (d, *J* = 7.5 Hz, 2H, *ortho*-Bz), 7.42 (t, *J* = 7.5 Hz, 1H, *para*-Bz), 7.34 (t, *J* = 7.5 Hz, 2H, *meta*-Bz), 7.22 (br, 1H, NH), 3.48 – 3.37 (m, 1H, C^1–H), 3.24 – 3.01 (comp, 2H, C^1–H, C^2–H), 1.82 (br, 2H, NH_2), 1.07 (d, *J* = 5.9 Hz, 3H, Me). ^{13}C NMR (126 MHz, $CDCl_3$) δ 167.8, 134.6, 131.3, 128.4, 127.0, 47.4, 46.6, 21.8. HRMS (ESI) *m/z* calcd for $C_{10}H_{15}N_2O$ $[M+H]^+$ 179.1179, found: 179.1182.

N-[2-(4-Nitrophenylsulfonamido)propyl]benzamide (6a’)

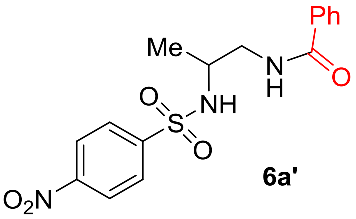

6a'

Compound **6a’** was prepared for HPLC determination of selectivity in benzoylation of 1,2-diaminopropane by BCPP **1a**. To a stirred solution of **6a** (87 mg, 0.49 mmol) in DCM (5 mL) triethylamine (0.14 mL, 1.0 mmol) was added in one portion followed by a dropwise addition of 4-nitrobenzenesulfonyl chloride (163 mg, 0.74 mmol) in DCM (3 mL) at 0 °C. The mixture was stirred at 20 °C for 30 min. Column chromatography on silica gel (with a 3:1 to 1:1 gradient of hexane:ethyl acetate as eluent) afforded **6a’** in quantitative yield (178 mg) as a white solid: mp 170–171 °C. ^{1}H NMR (500 MHz, $CDCl_3$) δ 8.12 (d, *J* = 8.7 Hz, 2H, Ar in Ns), 8.00 (d, *J* = 8.7 Hz, 2H, Ar in Ns), 7.65 (d, *J* = 7.4 Hz, 2H, *ortho*-Bz), 7.53 (t, *J* = 7.4 Hz, 1H, *para*-Bz), 7.41 (t, *J* = 7.4 Hz, 2H, *meta*-Bz), 6.64 (br, 1H, NHBz), 6.13 (br, 1H, NHNs), 3.65 – 3.57 (m, 1H, C<u>H</u>-NHNs), 3.56 – 3.47 (m, 1H, C<u>H$_2$</u>-NHBz), 3.42 – 3.34 (m, 1H, C<u>H$_2$</u>-NHBz), 1.24 (d, *J* = 6.5 Hz, 3H, Me). ^{13}C NMR (126 MHz, $CDCl_3$) δ 168.6, 149.7, 146.6, 132.9, 132.3, 128.7, 128.0, 126.8, 124.2, 52.0, 45.3, 20.1. HRMS (ESI) *m/z* calcd for $C_{16}H_{18}N_3O_5S$ $[M+H]^+$ 364.0962, found: 364.0963.

(*R*)-*N*-(2-Hydroxy-1-phenylethyl)benzamide (8)

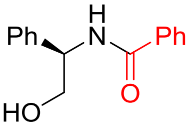

8

Reported compound.[3] Product was purified by flash chromatography on silica gel (with a 3:1 to 1:1 gradient of hexane:ethyl acetate as eluent): 120 mg, 100% yield; white solid, mp 146–147 °C. ^{1}H NMR (300 MHz, DMSO-d_6) δ 8.71 (d, *J* = 8.1 Hz, 1H, NH), 7.92 (dd, *J* = 8.1, 1.7 Hz, 2H, *ortho*-Bz), 7.56 – 7.18 (comp, 8H, Ar), 5.08 (td, *J* = 8.1, 5.7 Hz, 1H, CHN), 4.95 (t, *J* = 5.9 Hz, 1H, OH), 3.77 – 3.58 (comp, 2H, C<u>H$_2$</u>OH). ^{13}C NMR (75 MHz, DMSO-d_6) δ 166.6, 141.8, 135.1, 131.6,

128.6, 128.5, 127.8, 127.4, 127.3, 65.0, 56.4. HRMS (ESI) *m/z* calcd for $C_{15}H_{16}NO_2$ $[M+H]^+$ 242.1176, found: 242.1186.

[2-(2-Hydroxyethyl)piperidin-1-yl](phenyl)methanone (10)

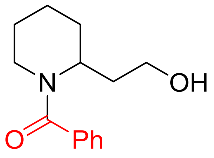

10

Reported compound.[4] Product was purified by flash chromatography on silica gel (with a 2:1 to 1:1 gradient of hexane:ethyl acetate as eluent): 111 mg, 95% yield; thick colorless oil. ^{1}H NMR (300 MHz, $CDCl_3$) δ 7.44 – 7.35 (comp, 5H, Ar), 4.95 (br, 1H, OH), 4.19 – 4.11 (m, 1H, CH–N), 3.75 – 3.55 (comp, 2H, CH_2–O), 3.46 (tt, *J* = 12.0, 3.1 Hz, 1H, CH_2–N), 2.90 (td, *J* = 11.6, 2.7 Hz, 1H, CH_2–N), 2.05 (ddt, *J* = 14.8, 11.9, 2.7 Hz, 1H, CH_2), 1.96 – 1.76 (comp, 2H, CH_2), 1.75 – 1.50 (comp, 5H, CH_2). ^{13}C NMR (75 MHz, $CDCl_3$) δ 172.1, 135.9, 129.7, 128.4, 126.4, 58.4, 45.7, 43.4, 32.1, 29.1, 25.9, 19.2. HRMS (ESI) *m/z* calcd for $C_{14}H_{20}NO_2$ $[M+H]^+$ 234.1489, found: 234.1500.

***N*-(2-Mercaptoethyl)benzamide (12)**

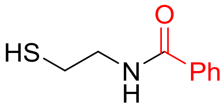

12

Reported compound.[5] Product was purified by flash chromatography on silica gel (with a 4:1 to 2:1 gradient of hexane:ethyl acetate as eluent): 88 mg, 97% yield; white solid, mp 69–70 °C. ^{1}H NMR (500 MHz, $CDCl_3$) δ 7.78 (dd, *J* = 7.5, 1.3 Hz, 2H, *ortho*-Bz), 7.48 (t, *J* = 7.5, 1H, *para*-Bz), 7.40 (t, *J* = 7.5 Hz, 2H, *meta*-Bz), 7.01 (br, 1H, NH), 3.59 (dd, *J* = 12.6, 6.4 Hz, 2H, CH_2–N), 2.74 (dt, *J* = 8.5, 6.4 Hz, 2H, CH_2–S), 1.41 (t, *J* = 8.5 Hz, 1H, SH). ^{13}C NMR (126 MHz, $CDCl_3$) δ 167.7, 134.3, 131.6, 128.6, 127.0, 42.9, 24.6. HRMS (ESI) *m/z* calcd for $C_9H_{12}NOS$ $[M+H]^+$ 182.0634, found: 182.0635.

Methyl *N*-benzoyl-*L*-cysteinate (14)

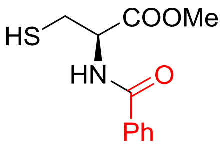

14

Reported compound.[5] The base form of *L*-cysteine methyl ester was prepared from the hydrochloride (86 mg, 0.5 mmol) by treatment with sodium bicarbonate (126 mg, 1.5 mmol) in water (2 mL). After extraction by DCM (3×5 mL), drying over anhydrous magnesium sulfate and evaporation of the solvent, the base form of *L*-cysteine methyl ester was obtained (67 mg, 99% yield) as a grey liquid. Monoamide **14** was purified by flash chromatography on silica gel (with a 3:1 to 1:1 gradient of hexane:ethyl acetate as eluent) to give 111 mg (93% yield) of a white solid, mp 62–63 °C. ^{1}H NMR (500 MHz, $CDCl_3$) δ 7.83 (dd, *J* = 7.6, 1.3 Hz, 2H, *ortho*-Bz), 7.52 (t, *J* = 7.6 Hz, 1H, *para*-Bz), 7.44 (t, *J* = 7.6 Hz, 2H, *meta*-Bz), 7.09 (br, 1H, NH), 5.07 (dt, *J* = 7.9, 4.1 Hz, 1H, C<u>H</u>-NH), 3.81 (s, 3H, Me), 3.17 – 3.07 (comp, 2H, CH_2–S), 1.41 (t, *J* = 9.0 Hz, 1H, SH). ^{13}C NMR (126 MHz, $CDCl_3$) δ 170.7, 167.1, 133.5, 131.9, 128.5, 127.2, 54.2, 52.8, 26.7. HRMS (ESI) *m/z* calcd for $C_{11}H_{14}NO_3S$ $[M+H]^+$ 240.0689, found: 240.0699.

***(±) trans-N*-(2-Aminocyclohexyl)benzamide (16)**

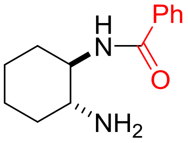

***rac*-16**

Reported compound.[6] Product was purified by flash chromatography on silica gel (with a 5:1 gradient of DCM:methanol to pure methanol as eluent): 107 mg, 98% yield; white solid, mp 125–126 °C. ^{1}H NMR (300 MHz, $CDCl_3$) δ 7.82 – 7.72 (comp, 2H, *ortho*-Bz), 7.51 – 7.39 (comp, 3H, Ar), 6.19 (br, 1H, NH), 3.75 – 3.69

(m, 1H, C^{1}H–N), 2.52 – 2.46 (m, 1H, C^{2}H–N), 2.16 – 2.08 (m, 1H, CH_2), 2.02 – 1.94 (m, 1H, CH_2), 1.80 – 1.70 (comp, 2H, CH_2), 1.56 (br, 2H, NH_2), 1.35 – 1.13 (comp, 4H, CH_2). ^{13}C NMR (75 MHz, $CDCl_3$) δ 168.0, 134.8, 131.4, 128.5, 127.1, 56.7, 55.4, 35.5, 32.5, 25.2, 25.1. HRMS (ESI) *m/z* calcd for $C_{13}H_{19}N_2O$ $[M+H]^+$ 219.1492, found: 219.1499.

N-(3-Aminopropyl)benzamide (18)

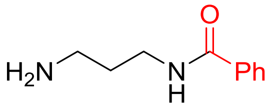

18

Reported compound.[7] Product was purified by flash chromatography on silica gel (with a 5:1 gradient of DCM:methanol to pure methanol as eluent): 87 mg, 98% yield; white solid, mp 44–45 °C. ^{1}H NMR (500 MHz, CD_3OD) δ 7.87 (dd, *J* = 7.5, 1.3 Hz, 2H, *ortho*-Bz), 7.53 (t, *J* = 7.5 Hz, 1H, *para*-Bz), 7.46 (t, *J* = 7.5 Hz, 2H, *meta*-Bz), 4.97 (br, 5H, NH, NH_2, HDO, H_2O), 3.51 (t, *J* = 6.6 Hz, 2H, C^{1}H–N), 3.02 (t, *J* = 7.4 Hz, 2H, C[3]H–N), 2.03 – 1.97 (comp, 2H, C^{2}H). ^{13}C NMR (126 MHz, CD_3OD) δ 169.3, 133.7, 131.4, 128.1, 127.0, 37.0, 36.1, 27.4. HRMS (ESI) *m/z* calcd for $C_{10}H_{15}N_2O$ $[M+H]^+$ 179.1179, found: 179.1185.

Methyl N[6]-benzoyl-L-lysinate (20)

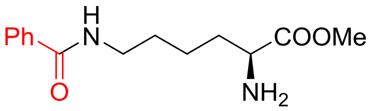

20

The base form of *L*-lysine methyl ester was prepared from its dihydrochloride (110 mg, 0.5 mmol) by treatment with sodium bicarbonate (126 mg, 1.5 mmol) in water (2 mL). Water was evaporated and the residue was dissolved in a mixture DCM:methanol 9:1 (5 mL), then the inorganic salts were filtered and washed with a mixture DCM:methanol 9:1 (2×5 mL). The solvents of the organic filtrate were evaporated to afford the base form of *L*-lysine methyl ester (80 mg, >99% yield) as a colorless oil. Monoamide **20** was purified by flash chromatography on silica gel (with a 10:1 to 3:1 gradient of DCM:methanol as eluent) to give 124 mg (94% yield) as a white solid, mp 238–239 °C (decomp). The reaction scale-up to 1.00 g (4.53 mmol) of *L*-lysine methyl ester after purification on silica gel afforded 1.13 g of **20** (95% yield). ^{1}H NMR (500 MHz, CD_3OD) δ 7.84 (d, *J* = 7.5 Hz, 2H, *ortho*-Bz), 7.51 (t, *J* = 7.5 Hz, 1H, *para*-Bz), 7.44 (t, *J* = 7.5 Hz, 2H, *meta*-Bz), 3.79 (t, *J* = 6.4 Hz, 1H, C^{2}H), 3.74 (s, 3H, Me), 3.40 (t, *J* = 6.9 Hz, 2H, C[6]H), 1.92 – 1.85 (m, 1H, CH_2), 1.84 – 1.77 (m, 1H, CH_2), 1.68 – 1.63 (comp, 2H, CH_2), 1.53 – 1.42 (comp, 2H, CH_2). ^{13}C NMR (126 MHz, CD_3OD) δ 172.5, 168.7, 134.3, 131.2, 128.2, 126.9, 53.1, 51.7, 39.1, 31.7, 28.7, 22.3. HRMS (ESI) *m/z* calcd for $C_{14}H_{21}N_2O_3$ $[M+H]^+$ 265.1547, found: 265.1558.

N-(3-Aminopentyl)benzamide (22)

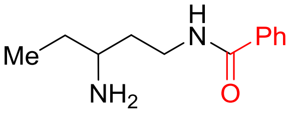

22

Reported compound.[2] Product was purified by flash chromatography on silica gel (with a 10:1 to 5:1 gradient of DCM:methanol as eluent): 103 mg, 100% yield; white solid, mp 116–117 °C. ^{1}H NMR (500 MHz, CD_3OD) δ 7.86 (d, *J* = 7.6 Hz, 2H, *ortho*-Bz), 7.54 (t, *J* = 7.6 Hz, 1H, *para*-Bz), 7.46 (t, *J* = 7.6 Hz, 2H, *meta*-Bz), 4.86 (br, 5H, NH, NH_2, HDO, H_2O), 3.64 – 3.55 (m, 1H, C[1]–H), 3.50 – 3.42 (m, 1H, C[1]–H), 3.14 (dq, *J* = 8.2, 6.3 Hz, 1H, C[3]–H), 2.04 – 1.96 (m, 1H, C[2]–H), 1.91 – 1.82 (m, 1H, C[2]–H), 1.78 – 1.66 (comp, 2H, C[4]–H), 1.02 (t, *J* = 7.5 Hz, 3H, Me). ^{13}C NMR

(126 MHz, CD_3OD) δ 169.5, 133.5, 131.6, 128.2, 127.0, 50.6, 35.5, 32.2, 25.5, 8.5. HRMS (ESI) *m/z* calcd for $C_{12}H_{19}N_2O$ $[M+H]^+$ 207.1492, found: 207.1500.

***N*-(3-[(4-Nitrophenyl)sulfonamido)pentyl]benzamide (22′)**

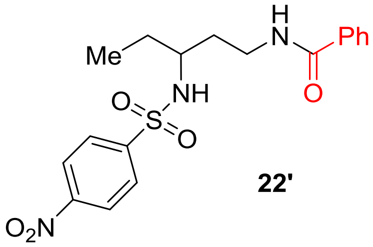

22'

Prepared for HPLC analysis of selectivity in benzoylation of 1,3-diaminopentane by BCPP **1a** following the procedure for **6a′**. Purified by flash chromatography on silica gel (with a 3:1 to 1:1 gradient of hexane:ethyl acetate as eluent): 195 mg, >99% yield; white solid, mp 79–80 °C. ^{1}H NMR (500 MHz, $CDCl_3$) δ 8.26 (d, *J* = 8.7 Hz, 2H, Ar in Ns), 8.02 (d, *J* = 8.7 Hz, 2H, Ar in Ns), 7.80 (d, *J* = 7.5 Hz, 2H, *ortho*-Bz), 7.50 (t, *J* = 7.5 Hz, 1H, *para*-Bz), 7.43 (t, *J* = 7.5 Hz, 2H, *meta*-Bz), 7.14 (br, 1H, NHBz), 6.13 (br, 1H, NHNs), 3.77 – 3.71 (m, 1H, C$\underline{H_2}$-NHBz), 3.44 – 3.26 (comp, 2H, C$\underline{H_2}$-NHBz, C$\underline{H}$-NHNs), 1.94 – 1.85 (m, 1H, C^2–H), 1.65 – 1.56 (m, 1H, C^2–H), 1.43 – 1.26 (comp, 2H, C^4–H), 0.60 (t, *J* = 7.4 Hz, 3H, Me). ^{13}C NMR (126 MHz, $CDCl_3$) δ 167.7, 149.8, 147.2, 134.1, 131.7, 128.7, 128.1, 126.9, 124.3, 54.1, 36.3, 34.6, 28.3, 10.2. HRMS (ESI) *m/z* calcd for $C_{18}H_{22}N_3O_5S$ $[M+H]^+$ 392.1275, found: 392.1276.

***N*-[(5-Amino-1,3,3-trimethylcyclohexyl]methyl)benzamide (24)**

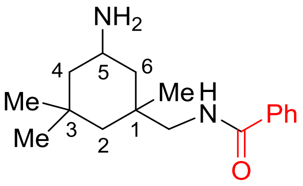

24

Purified by flash chromatography on silica gel (with a 5:1 to 1:1 gradient of DCM:methanol as eluents): 133 mg, 97% yield; sticky colorless oil. Obtained as a mixture of *trans*- and *cis*-isomers (*trans*/*cis* = 85:15), ratio *trans*/*cis* is the same with starting material. ^{1}H NMR of *trans*-**24** (500 MHz, $CDCl_3$) δ 7.76 (d, *J* = 7.4 Hz, 2H, *ortho*-Bz), 7.50 (t, *J* = 7.4 Hz, 1H, *para*-Bz), 7.43 (t, *J* = 7.4 Hz, 2H, *meta*-Bz), 6.23 (br, 1H, NH), 3.22 (d, *J* = 6.4 Hz, 2H, CH_2–N), 3.10 – 2.98 (m, 1H, C^5H–N), 1.76 (br, 2H, NH_2), 1.68 – 1.60 (comp, 2H, Cy), 1.33 – 1.26 (comp, 2H, Cy), 1.09 (s, 3H, Me), 1.03 (s, 3H, Me), 0.97 – 0.94 (m, 1H, Cy), 0.94 (s, 3H, Me), 0.92 – 0.85 (m, 1H, Cy). ^{13}C NMR (126 MHz, $CDCl_3$) δ 167.8, 134.9, 131.3, 128.5, 126.9, 53.5, 49.8, 47.4, 45.5, 44.0, 36.8, 35.2, 32.0, 27.9, 23.7. HRMS (ESI) *m/z* calcd for $C_{17}H_{27}N_2O$ $[M+H]^+$ 275.2118, found: 275.2127.

***N*-(3-(Cyclohexylamino)propyl)benzamide (26)**

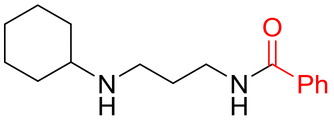

26

Reported compound.[8] Product was purified by flash chromatography on silica gel (with a 5:1 to 1:1 gradient of DCM:methanol as eluent): 130 mg, 100% yield; white solid, mp 190–191 °C. ^{1}H NMR (500 MHz, CD_3OD) δ 7.88 (d, *J* = 7.5 Hz, 2H, *ortho*-Bz), 7.58 (t, *J* = 7.5 Hz, 1H, *para*-Bz), 7.50 (t, *J* = 7.5 Hz, 2H, *meta*-Bz), 3.54 (t, *J* = 6.2 Hz, 2H, C$\underline{H_2}$NHBz), 3.14 – 3.04 (comp, 3H, C$\underline{H_2}$NHCy, NHC$\underline{H}$Cy), 2.22 – 2.08 (comp, 2H, CH_2), 2.08 – 1.98 (comp, 2H, CH_2), 1.98 – 1.83 (comp, 2H, CH_2), 1.78 – 1.69 (m, 1H, CH_2), 1.46 – 1.34 (comp, 4H, CH_2), 1.33 – 1.20 (m, 1H, CH_2). ^{13}C NMR (126 MHz, CD_3OD) δ 169.7, 133.6, 131.6, 128.3, 126.9, 57.1, 41.9, 36.1, 29.0, 26.6, 24.7, 24.0. HRMS (ESI) *m/z* calcd for $C_{16}H_{25}N_2O$ $[M+H]^+$ 261.1961, found: 261.1972.

N-[3-([4-(3-Aminopropylamino)butyl]amino)propyl]benzamide (28)

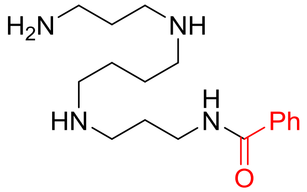

28

Purified by C18-reversed phase chromatography (with a gradient of pure water to water:acetonitrile 1:1 as eluent): 142 mg, 93% yield; pale yellow oil. ^{1}H NMR (500 MHz, $CDCl_3$) δ 8.20 (br, 1H, NHBz), 7.77 (t, *J* = 7.6 Hz, 2H, *ortho*-Bz), 7.43 (t, *J* = 7.6 Hz, 1H, *para*-Bz), 7.36 (t, *J* = 7.6 Hz, 2H, *meta*-Bz), 3.51 (t, *J* = 6.4 Hz, 2H, C$\underline{H_2}$NHBz), 2.80 – 2.10 (comp, 14H, NH, CH_2), 1.75 – 1.71 (comp, 2H, CH_2), 1.65 – 1.54 (comp, 2H, CH_2), 1.48 (br, 4H, NH). ^{13}C NMR (126 MHz, $CDCl_3$) δ 167.3, 134.8, 131.1, 128.4, 127.0, 49.7, 48.8, 47.8, 40.5, 39.9, 39.8, 33.5, 28.3, 28.2, 27.8. HRMS (ESI) *m/z* calcd for $C_{17}H_{31}N_4O$ $[M+H]^+$ 307.2492, found: 307.2499.

N-[3-(4-Benzamidobutylamino)propyl]benzamide (30)

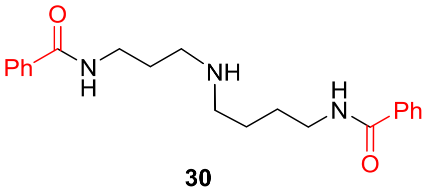

30

Reported compound.[9] Product was purified by flash chromatography on silica gel (with a gradient of DCM:methanol 3:1 to pure methanol as eluent): 173 mg, 98% yield; white solid, mp 130–131 °C. ^{1}H NMR (500 MHz, CD_3OD) δ 7.89 – 7.83 (comp, 4H, *ortho*-Bz), 7.55 – 7.51 (comp, 2H, *para*-Bz), 7.45 (td, *J* = 7.6, 2.9 Hz, 4H, *meta*-Bz), 4.83 (br, 9H, NH, HDO, H_2O), 3.52 (t, *J* = 6.4 Hz, 2H, C$\underline{H_2}$NHBz), 3.45 (t, *J* = 6.4 Hz, 2H, C$\underline{H_2}$NHBz), 3.08 (t, *J* = 7.4 Hz, 4H, C$\underline{H_2}$NH), 2.10 – 1.98 (comp, 2H, CH_2), 1.88 – 1.76 (comp, 2H, CH_2), 1.78 – 1.69 (comp, 2H, CH_2). ^{13}C NMR (126 MHz, CD_3OD) δ 169.4, 168.9, 134.2, 133.6, 131.5, 131.3, 128.2, 127.0, 126.9, 46.5, 45.2, 38.6, 36.1, 26.3, 26.1, 23.3. HRMS (ESI) *m/z* calcd for $C_{21}H_{28}N_3O_2$ $[M+H]^+$ 354.2176, found: 354.2186.

N-6'-Benzoylkanamycin (34)

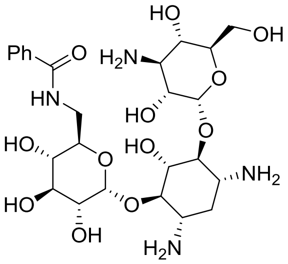

34

Product was purified by flash chromatography on silica gel (with a gradient of methanol/28wt% aqueous ammonium hydroxide 100:0 to 80:20 as eluent): 91 mg, 77% yield; white solid, mp 255–256 °C. ^{1}H NMR (500 MHz, D_2O) δ 7.77 (d, *J* = 7.4 Hz, 2H), 7.62 (t, *J* = 7.4 Hz, 1H), 7.53 (t, *J* = 7.4 Hz, 2H), 5.23 (d, *J* = 3.8 Hz, 1H), 5.00 (d, *J* = 3.6 Hz, 1H), 3.99 – 3.67 (comp, 7H), 3.63 – 3.46 (comp, 4H), 3.37 – 3.29 (comp, 2H), 3.21 (dt, *J* = 15.3, 9.5 Hz, 2H), 3.00 (t, *J* = 9.9 Hz, 1H), 2.90 – 2.81 (m, 1H), 2.79 – 2.71 (m, 1H), 1.82 (dt, *J* = 9.1, 4.0 Hz, 1H), 1.07 (dd, *J* = 12.4 Hz, 1H). ^{13}C NMR (126 MHz, D_2O) δ 170.9, 133.3, 132.4, 129.0, 127.1, 100.4, 100.0, 88.0, 87.3, 74.2, 72.9, 72.2, 72.0, 71.6, 71.4, 71.4, 69.1, 60.3, 54.3, 50.3, 49.3, 40.9, 35.0. HRMS (ESI) *m/z* calcd for $C_{25}H_{42}N_4O_{12}$ $[M+2H]^{2+}$ 295.1394, found: 295.1392.

N-6'-Benzoyltobramycin (36)

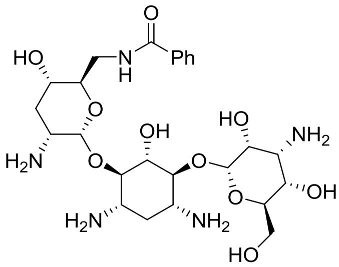

36

Product was purified by flash chromatography on silica gel (with a gradient of methanol/28wt% aqueous ammonium hydroxide 100:0 to 80:20 as eluent): 105 mg, 92% yield; white solid, mp 212–213 °C. ^{1}H NMR (500 MHz, D_2O) δ 7.66 (d, *J* = 7.4 Hz, 2H), 7.51 (t, *J* = 7.4 Hz,

1H), 7.42 (t, J = 7.4 Hz, 2H), 4.96 (d, J = 2.8 Hz, 1H), 4.89 (d, J = 3.4 Hz, 1H), 3.80 – 3.75 (m, 1H), 3.74 – 3.59 (comp, 4H), 3.49 – 3.33 (comp, 4H), 3.26 – 3.17 (comp, 2H), 3.12 (t, J = 9.3 Hz, 1H), 3.06 (t, J = 9.3 Hz, 1H), 2.94 – 2.85 (comp, 2H), 2.78 – 2.67 (m, 1H), 2.67 – 2.57 (m, 1H), 1.99 – 1.90 (m, 1H), 1.71 – 1.63 (m, 1H), 1.53 (dd, 11.9 Hz, 1H), 0.95 (dd, J = 12.5 Hz, 1H). ^{13}C NMR (126 MHz, D_2O) δ 170.7, 133.2, 132.3, 128.8, 127.0, 99.9, 99.7, 87.7, 87.0, 74.2, 72.1, 71.6, 69.1, 66.4, 60.2, 54.2, 50.2, 49.5, 49.1, 48.8, 40.8, 35.1, 34.3. HRMS (ESI) *m/z* calcd for $C_{25}H_{42}N_5O_{10}$ $[M+H]^+$ 572.2926, found: 572.2925.

***N*-AHB-Benzoylamikacin (38)**

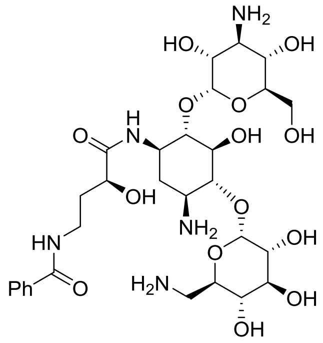

38

Product was purified by flash chromatography on silica gel (with a gradient of methanol/28wt% aqueous ammonium hydroxide 100:0 to 90:10 as eluent): 106 mg, 77% yield; white solid, mp 262–263 °C. ^{1}H NMR (500 MHz, D_2O) δ 7.73 (d, J = 7.6 Hz, 2H), 7.59 (t, J = 7.6 Hz, 1H), 7.50 (t, J = 7.6 Hz, 2H), 5.49 (d, J = 3.8 Hz, 1H), 5.11 (d, J = 3.6 Hz, 1H), 4.20 (dd, J = 8.8, 3.6 Hz, 1H), 4.02 – 3.92 (comp, 3H), 3.80 – 3.69 (comp, 5H), 3.63 – 3.57 (comp, 2H), 3.56 – 3.47 (comp, 4H), 3.41 – 3.30 (comp, 2H), 3.21 (t, J = 10.3 Hz, 1H), 3.16 – 3.04 (comp, 2H), 2.15 – 2.05 (m, 1H), 2.00 – 1.84 (comp, 2H), 1.52 (dd, J = 12.6 Hz, 1H). ^{13}C NMR (126 MHz, D_2O) δ 176.2, 170.9, 133.6, 132.2, 128.8, 127.1, 98.1, 97.5, 82.6, 80.6, 74.2, 72.4, 72.1, 71.3, 71.1, 69.5, 69.3, 68.8, 66.9, 60.0, 54.9, 49.3, 48.4, 40.5, 38.8, 36.4, 33.0. HRMS (ESI) *m/z* calcd for $C_{29}H_{49}N_5O_{14}$ $[M+2H]^{2+}$ 345.6633, found: 345.6631.

***N*-6'-*N*-AHB-Dibenzoylamikacin (39)**

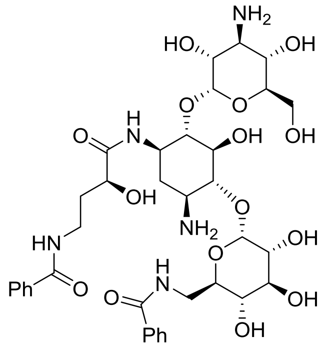

39

Product was purified by flash chromatography on silica gel (with a gradient of methanol/28wt% aqueous ammonium hydroxide 100:0 to 90:10 as eluent): 22 mg, 14% yield; white solid, mp 237–238 °C. ^{1}H NMR (500 MHz, D_2O) δ 7.78 – 7.71 (comp, 4H), 7.62 – 7.56 (comp, 2H), 7.51 (t, J = 7.6 Hz, 2H), 7.48 (t, J = 7.6 Hz, 2H), 5.33 (d, J = 3.9 Hz, 1H), 5.10 (d, J = 3.7 Hz, 1H), 4.20 (dd, J = 8.8, 3.6 Hz, 1H), 4.05 – 3.98 (m, 1H), 3.97 – 3.91 (comp, 2H), 3.82 – 3.67 (comp, 7H), 3.66 – 3.59 (comp, 3H), 3.58 – 3.47 (comp, 3H), 3.38 – 3.30 (comp, 2H), 3.19 – 3.10 (m, 1H), 2.14 – 2.05 (m, 1H), 1.97 (dt, J = 13.0, 4.3 Hz, 1H), 1.92 – 1.83 (m, 1H), 1.50 (dd, J = 12.6 Hz, 1H). ^{13}C NMR (126 MHz, D_2O) δ 176.2, 171.2, 170.9, 133.6, 133.4, 132.4, 132.2, 128.9, 128.8, 127.2, 127.1, 99.0, 97.9, 83.3, 80.0, 73.8, 72.5, 72.0, 71.5, 71.1, 69.5, 68.3, 65.8, 59.7, 55.2, 49.0, 48.8, 40.6, 38.8, 36.4, 33.0, 31.8. HRMS (ESI) *m/z* calcd for $C_{36}H_{53}N_5O_{15}$ $[M+2H]^{2+}$ 397.6764, found: 397.6760.

N-AHB-(4-Methoxybenzoyl)amikacin (40)

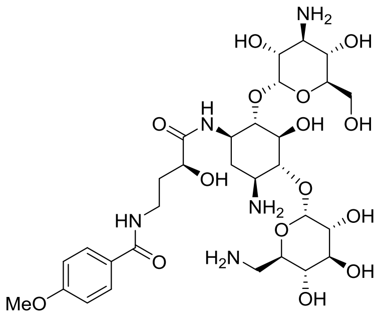

40

Product was purified by flash chromatography on silica gel (with a gradient of methanol/28wt% aqueous ammonium hydroxide 100:0 to 80:20 as eluent): 101 mg, 70% yield; off-white solid, mp 210–211 °C. ^{1}H NMR (500 MHz, D_2O) for two rotamers: δ 7.71 (dd, *J* = 8.9, 3.6 Hz, 4H), 7.07 – 6.97 (comp, 4H), 5.38 (d, *J* = 4.0 Hz, 1H), 5.23 (dd, *J* = 7.1, 4.0 Hz, 1H), 5.05 (d, *J* = 3.4 Hz, 2H), 4.22 – 4.13 (comp, 2H), 4.00 – 3.86 (comp, 6H), 3.84 (s, 6H, OMe), 3.80 – 3.66 (comp, 11H), 3.60 – 3.53 (comp, 3H), 3.52 – 3.45 (comp, 4H), 3.41 – 3.28 (comp, 8H), 3.26 – 3.15 (comp, 2H), 3.06 – 2.82 (comp, 5H), 2.14 – 2.04 (comp, 2H), 1.95 – 1.81 (comp, 4H), 1.48 (dd, *J* = 12.6 Hz, 1H), 1.37 (dd, *J* = 12.6 Hz, 1H). ^{13}C NMR (126 MHz, D_2O) for two rotamers: δ 176.1, 176.0, 170.1, 170.0, 163.4, 162.0, 129.1, 129.1, 126.0, 114.1, 100.6, 100.1, 98.7, 98.5, 98.4, 85.3, 84.8, 80.8, 79.9, 75.6, 74.7, 72.7, 72.5, 72.1, 72.0, 71.9, 71.7, 71.6, 71.3, 71.1, 70.7, 69.6, 69.5, 68.9, 68.8, 68.6, 60.3, 55.6, 54.2, 49.7, 49.6, 48.5, 41.0, 40.9, 36.4, 36.3, 34.1, 33.0. HRMS (ESI) *m/z* calcd for $C_{30}H_{51}N_5O_{15}$ $[M+2H]^{2+}$ 360.6685, found: 360.6686.

N-6'-N-AHB-Bis(4-methoxybenzoyl)amikacin (41)

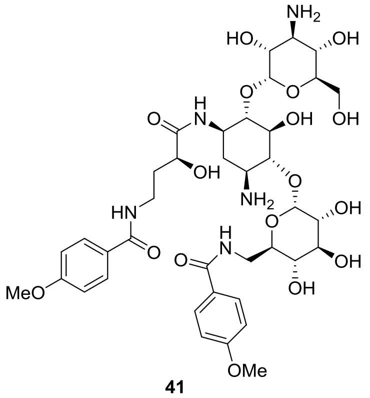

41

Product was purified by flash chromatography on silica gel (with a gradient of methanol/28wt% aqueous ammonium hydroxide 100:0 to 80:20 as eluent): 38 mg, 23% yield; off-white solid, mp 222–223 °C. ^{1}H NMR (500 MHz, D_2O) δ 7.63 (dd, *J* = 15.0, 8.9 Hz, 4H), 6.94 (dd, *J* = 12.6, 8.9 Hz, 4H), 5.13 (d, *J* = 3.8 Hz, 1H), 4.97 (d, *J* = 3.7 Hz, 1H), 4.07 (dd, *J* = 8.7, 3.8 Hz, 1H), 3.92 – 3.88 (dt, *J* = 10.0, 3.0 Hz, 1H), 3.84 – 3.77 (comp, 2H), 3.75 (s, 3H, OMe), 3.73 (s, 3H, OMe), 3.73 – 3.67 (m, 1H), 3.64 – 3.55 (comp, 5H), 3.54 – 3.48 (comp, 3H), 3.47 – 3.34 (comp, 5H), 3.26 – 3.20 (comp, 2H), 3.16 (t, *J* = 10.4 Hz, 1H), 2.04 – 1.92 (m, 1H), 1.80 – 1.72 (m, 1H), 1.22 (dd, *J* = 12.6 Hz, 1H). ^{13}C NMR (126 MHz, DMSO-d_6) δ 174.6, 167.1, 166.6, 162.0, 161.9, 129.6, 129.5, 127.1, 126.8, 113.9, 101.6, 98.0, 90.3, 80.5, 75.3, 73.2, 72.8, 72.4, 71.9, 71.2, 69.8, 68.1, 60.7, 55.8, 55.6, 50.0, 49.4, 41.5, 36.8, 35.6, 34.4. HRMS (ESI) *m/z* calcd for $C_{38}H_{57}N_5O_{17}$ $[M+2H]^{2+}$ 427.6869, found: 427.6868.

N-6'-N-6'''-Dibenzoylneomycin B (43)

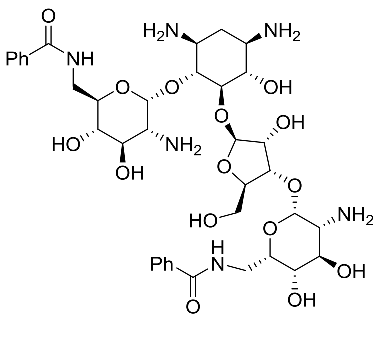

43

Product was purified by flash chromatography on silica gel (with a gradient of methanol/28wt% aqueous ammonium hydroxide 100:0 to 90:10 as eluent): 128 mg, 78% yield; white solid, mp 110–111 °C. ^{1}H NMR (500 MHz, D_2O) δ 7.73 (comp, 4H), 7.58 (t, *J* = 7.5 Hz, 1H), 7.49 (comp, 5H), 5.30 (d, *J* = 3.6 Hz, 1H), 5.20 (d, *J* = 2.1 Hz, 1H), 4.88 (d, *J* = 1.3 Hz, 1H), 4.28 – 4.23 (m, 1H), 4.18 – 4.12 (comp, 2H), 4.08 – 4.03 (comp, 2H), 3.97 – 3.90 (m, 1H),

3.81 – 3.44 (comp, 11H), 3.34 – 3.27 (m, 1H), 3.20 (t, *J* = 9.3 Hz, 1H), 3.03 (s, 1H), 2.85 (t, *J* = 9.3 Hz, 1H), 2.76 – 2.65 (comp, 2H), 2.63 – 2.53 (m, 1H), 1.75 (dt, *J* = 12.8, 4.0 Hz, 1H), 0.93 (dd, *J* = 12.5 Hz, 1H). ^{13}C NMR (75 MHz, D_2O) δ 170.8, 170.6, 133.3, 133.2, 132.4, 132.3, 128.9, 128.8, 127.1, 127.0, 108.8, 99.1, 98.2, 84.2, 82.1, 81.3, 77.1, 76.1, 73.2, 73.0, 72.9, 71.8, 71.1, 70.5, 68.1, 61.6, 55.3, 52.5, 50.2, 50.0, 40.9, 40.7, 35.2. HRMS (ESI) *m/z* calcd for $C_{37}H_{56}N_6O_{15}$ $[M+2H]^{2+}$ 412.1896, found: 412.1894.

5-(Cyclopent-1-en-1-yl)-3-phenyl-1*H*-pyrazol-4-yl benzoate (44)

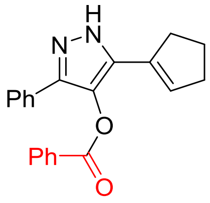

44

Synthetic procedure. A solution of BCPP **1a** (66.0 mg, 0.2 mmol) in methanol (15 mL) was kept at 20 °C for 12 h. Compound **44** was formed quantitatively and isolated as a white solid (mp 169–170 °C) after evaporation of methanol. ^{1}H NMR (500 MHz, $CDCl_3$) δ 8.31 (d, *J* = 7.5 Hz, 2H, Ar), 7.78 – 7.65 (comp, 3H, Ar), 7.58 (t, *J* = 7.7 Hz, 2H, Ar), 7.35 – 7.21 (comp, 3H, Ar), 6.10 – 6.07 (m, 1H, =CH), 2.72 – 2.69 (comp, 2H, CH_2C=), 2.42 – 2.40 (comp, 2H, CH_2C=), 1.85 (p, *J* = 7.4 Hz, 2H, CH_2). ^{13}C NMR (126 MHz, $CDCl_3$) δ 164.7, 133.9, 131.1, 130.4, 129.1, 129.0, 128.8, 128.7, 128.0, 126.2, 33.4, 33.0, 22.5. HRMS (ESI) *m/z* calcd for $C_{21}H_{19}N_2O_2$ $[M+H]^+$ 331.1441, found: 331.1444.

3-([1,1'-Biphenyl]-4-yl)-5-(cyclopent-1-en-1-yl)-1*H*-pyrazol-4-yl benzoate (44a)

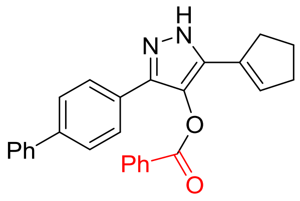

44a

Under the same reaction conditions with **44** compound **44a** was formed quantitatively from the corresponding BCPP and isolated as a white solid: mp 213–214 °C. ^{1}H NMR (500 MHz, $CDCl_3$) δ 8.27 (d, *J* = 7.2 Hz, 2H, Ar), 7.74 (d, *J* = 8.3 Hz, 2H, Ar), 7.69 (t, *J* = 7.5 Hz, 1H, Ar), 7.63 – 7.49 (comp, 6H, Ar), 7.39 (t, *J* = 7.5 Hz, 2H, Ar), 7.31 (t, *J* = 7.5 Hz, 1H, Ar), 6.12 – 6.09 (m, 1H, =CH), 2.75 – 2.71 (comp, 2H, CH_2C=), 2.45 – 2.42 (comp, 2H, CH_2C=), 1.92 (p, *J* = 7.4 Hz, 2H, CH_2). ^{13}C NMR (126 MHz, $CDCl_3$) δ 164.7, 140.8, 140.5, 139.9, 136.1, 134.0, 131.0, 130.4, 130.1, 129.3, 129.0, 128.8, 128.7, 128.4, 127.4, 127.3, 127.0, 126.5, 33.5, 33.0, 22.6. HRMS (ESI) *m/z* calcd for $C_{27}H_{23}N_2O_2$ $[M+H]^+$ 407.1754, found: 407.1756.

Experimental Procedure for the Kinetic Study of the Reaction Between BCPP and Amines

Rate constants for the reaction of BCPP with a series of amines were determined using UV-Vis spectroscopy at 24±0.5 ^{o}C in dichloromethane. BCPP (**1a**) has an absorbance in the range 300 – 440 nm (covers visible light range), while other reactants and products absorb only in UV range. It allows monitoring the disappearance of BCPP at a certain wavelength (e.g. 380 nm). All kinetic measurements have been carried out above DCM cutoff (λ = 245 nm).

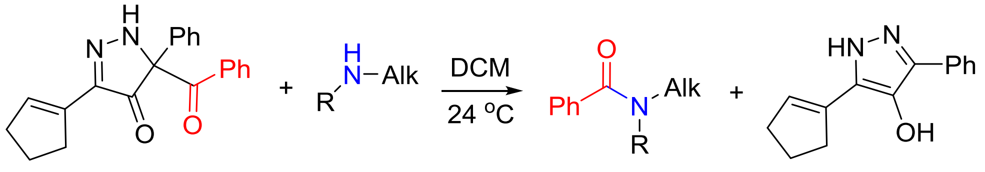

1a **2** **3** **4**

UV-Vis spectrum of BCPP (**1a**):

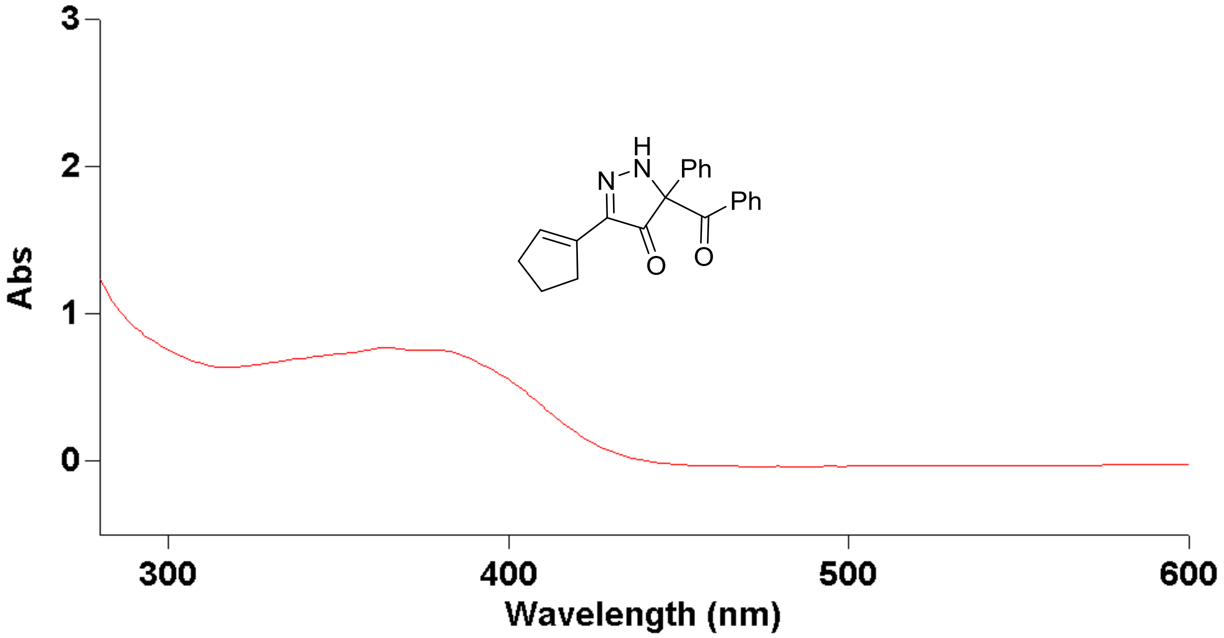

UV-Vis spectrum of **4**:

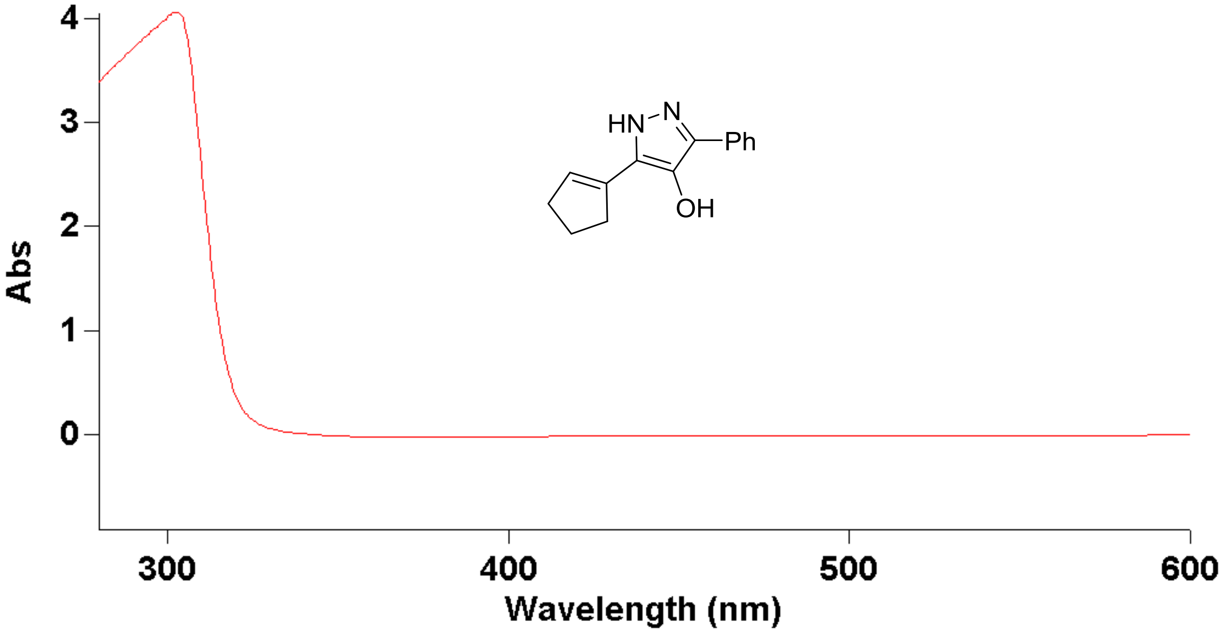

Extinction coefficient determination for BCPP (**1a**) at 380 nm.

Absorbance for a series of solutions of BCPP in DCM was measured at 24 °C. Extinction coefficient ε was determined from Beer-Lambert law: A = εcl (l = 1 cm) as slope of the linear plot A = f(c).

c (BCPP), mol/L	A
0	0
0.000115	0.3098
0.00021	0.6118
0.000254	0.7495
0.000393	1.2026
0.000533	1.5308

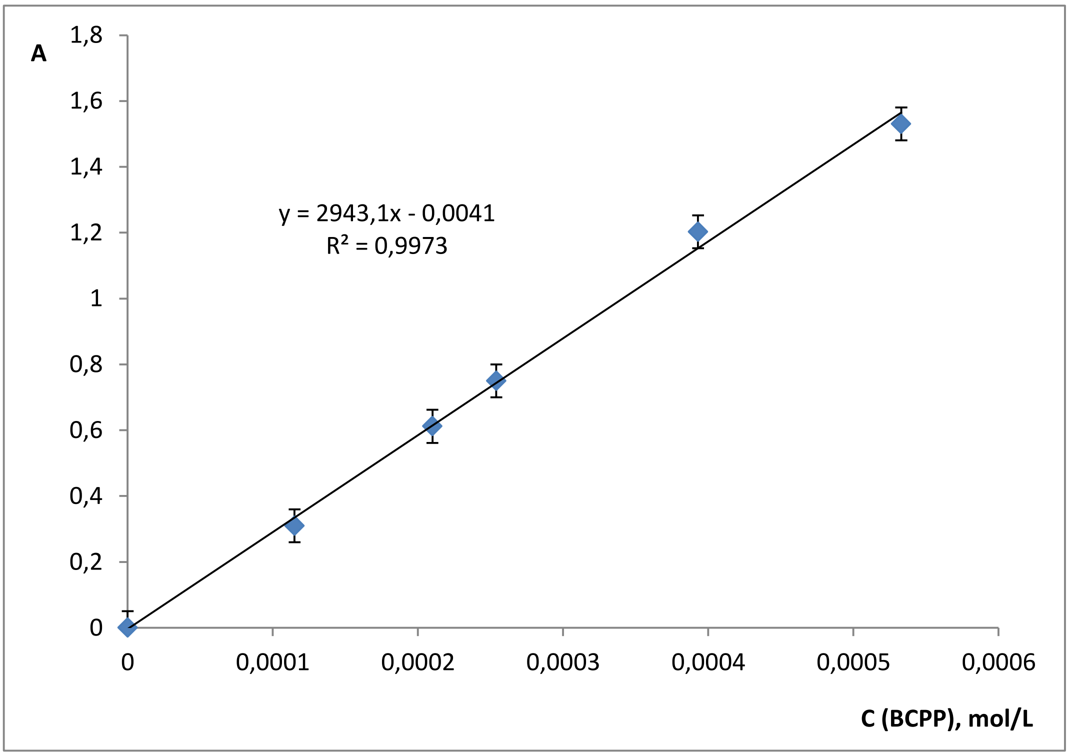

Extinction coefficient ε = 2920±70 was determined from three parallel experiments.

Determination of second order reaction rate.

Kinetic runs were performed for the reaction of BCPP with a series of amine solutions of different concentrations ($C_{amine} \geq 10 \cdot C_{BCPP}$).

Considering that the extinction coefficient for BCPP at 380 nm ε = 2920, a BCPP solution was prepared to have an absorbance A = 0.4–0.8 (at 380 nm). Concentrations of amine solutions ($C_{amine} \geq 10 \cdot C_{BCPP}$) were used to have reaction times less than one hour.

Kinetic data for the reaction of BCPP with amines.

1) 2-amino-2-methylpropane (C_{BCPP} = $2.4 \cdot 10^{-4}$ M):

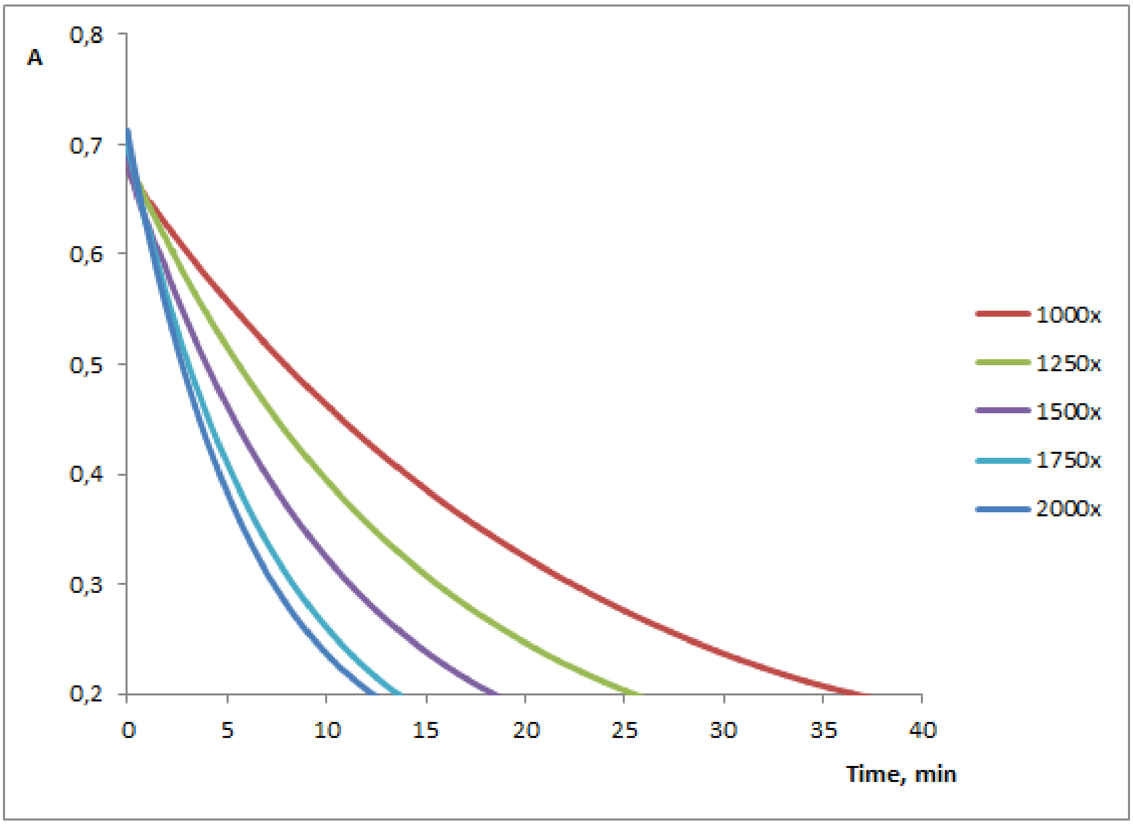

* 1000x, 1250x, 1500x, 1750x, 2000x – excess of amine taken

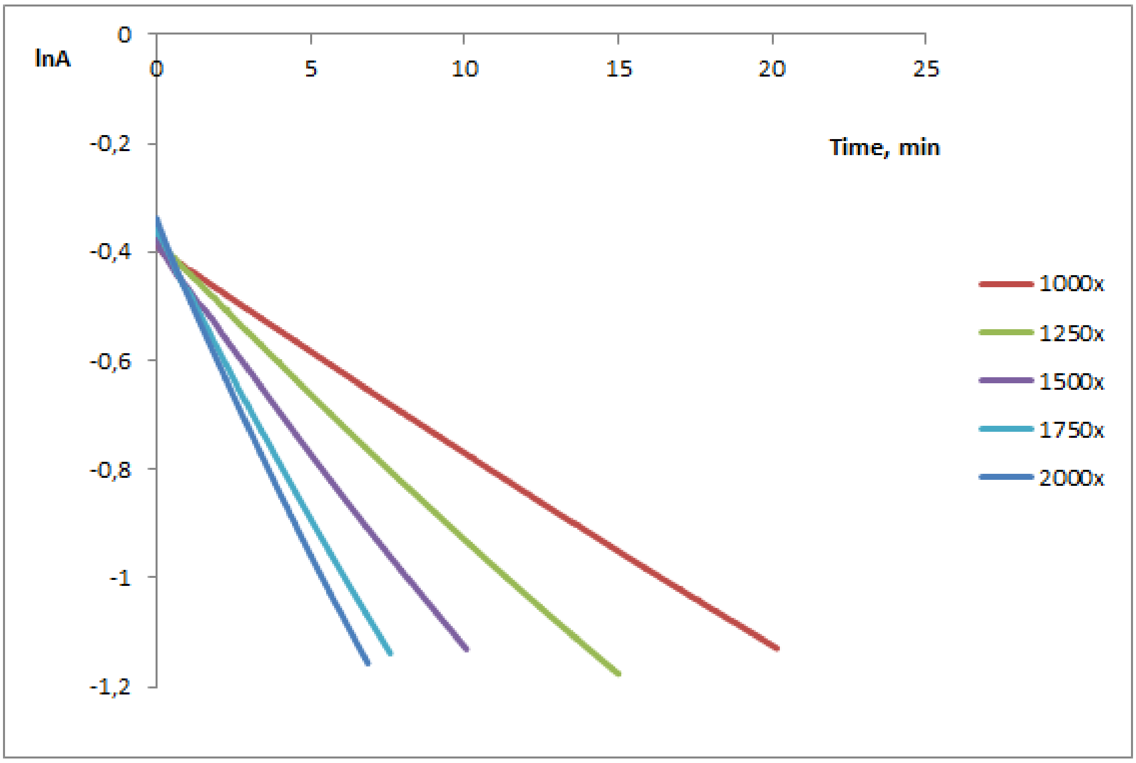

* 1000x, 1250x, 1500x, 1750x, 2000x – excess of amine taken

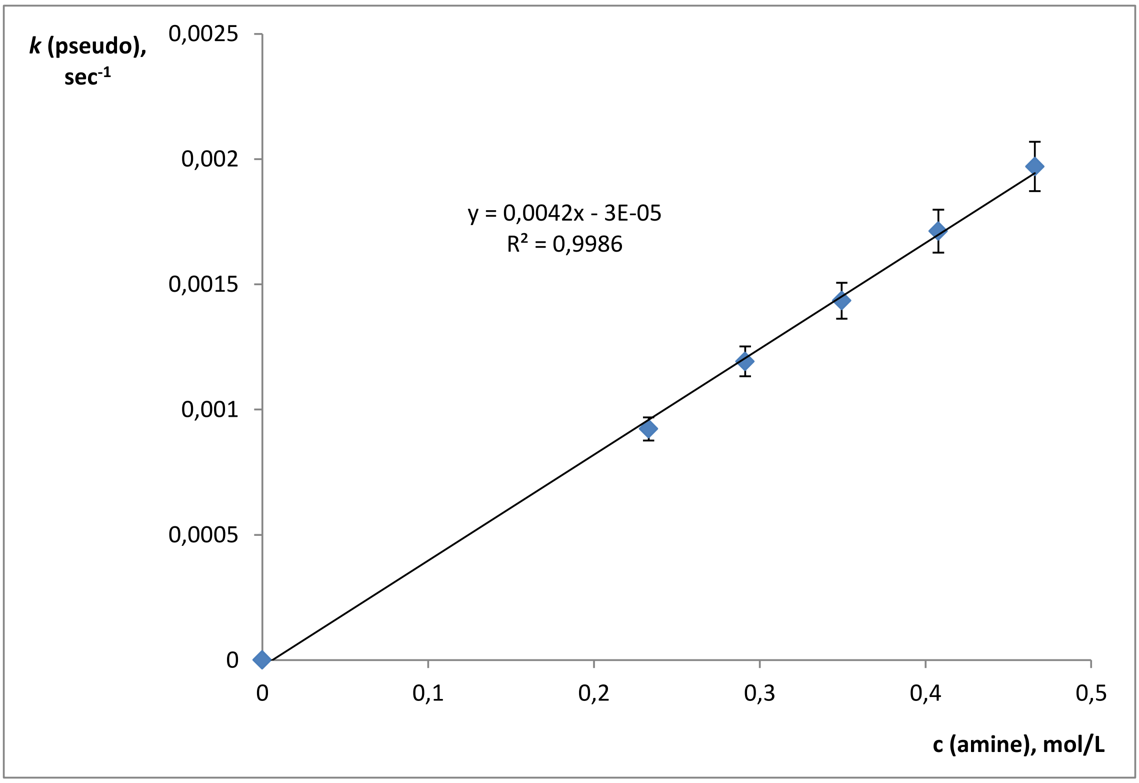

Second order rate constant in DCM at 24 °C obtained from three parallel experiments: k = 0.0042±0.0002 (L·mol^{-1}·sec^{-1}).

2) 2-Aminobutane (C_{BCPP} = 2.2·10^{-4} M):

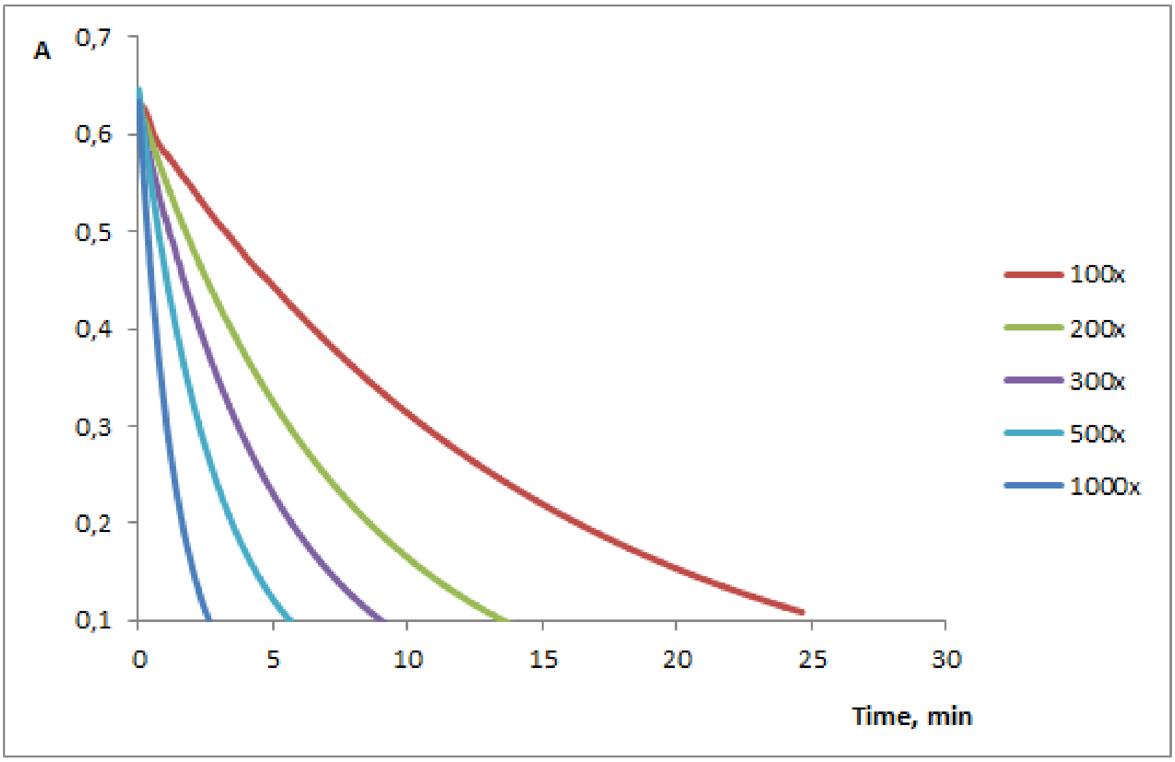

* 100x, 200x, 300x, 500x, 1000x – excess of amine taken

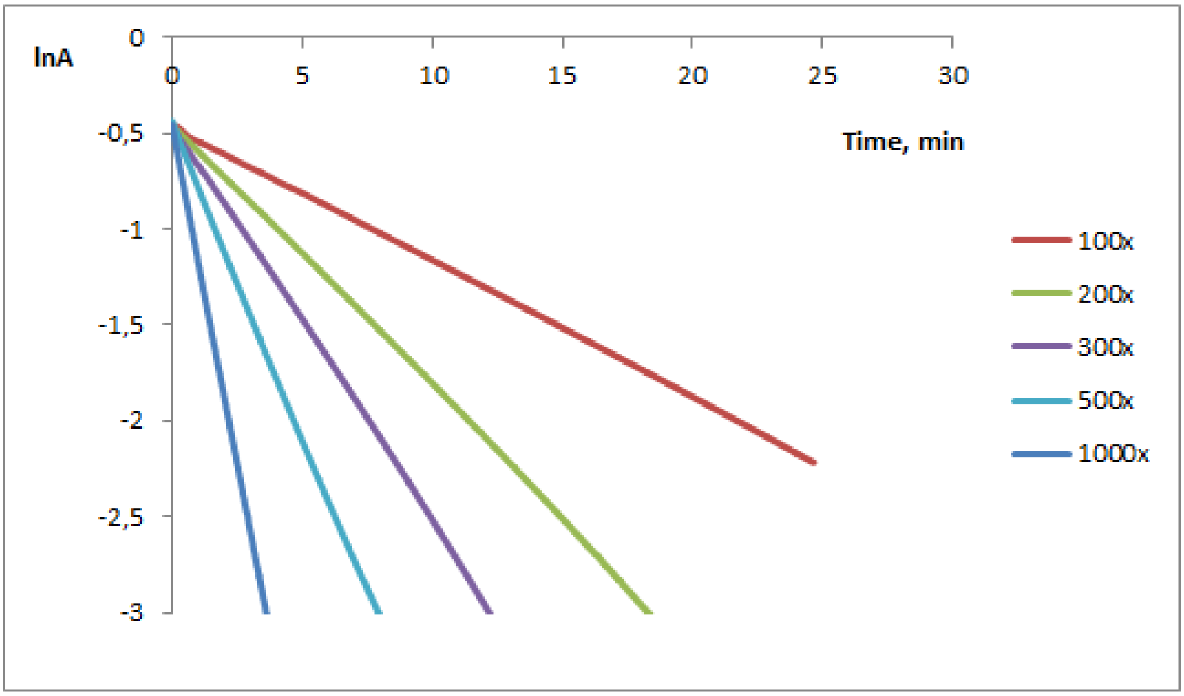

* 100x, 200x, 300x, 500x, 1000x – excess of amine taken

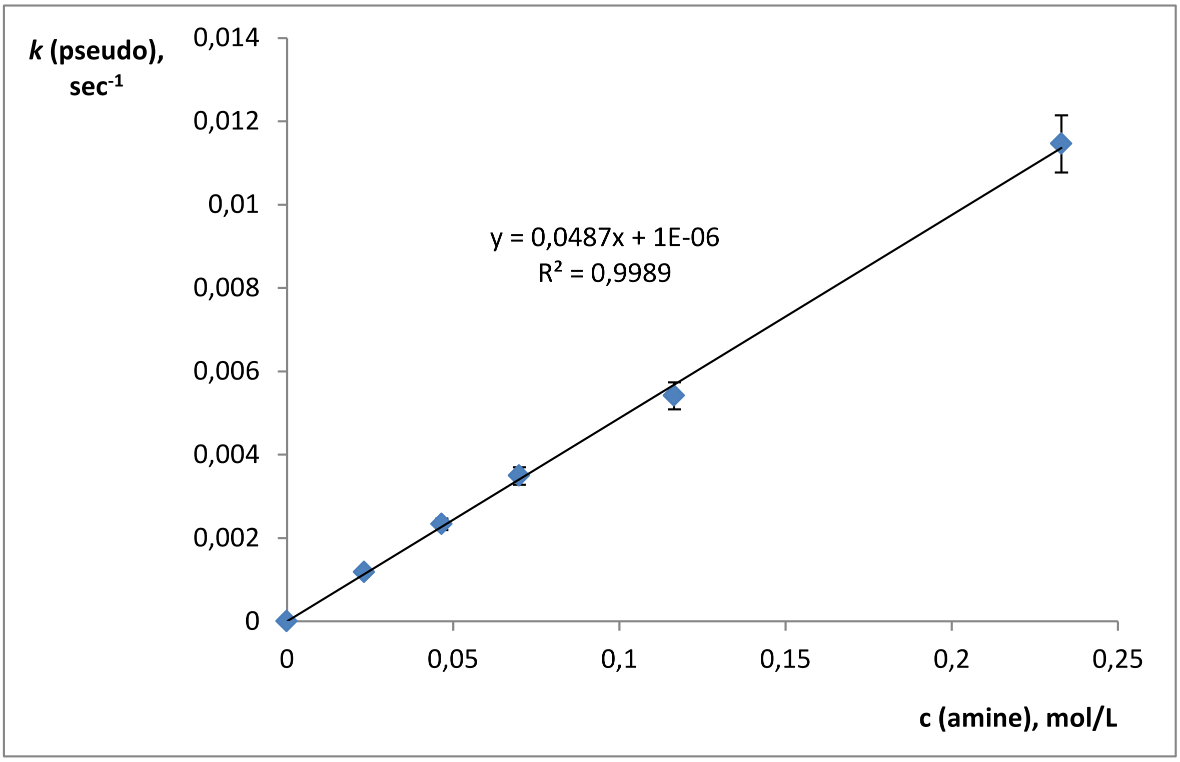

Second order rate constant in DCM at 24 ^{o}C obtained from three parallel experiments:
k = 0.049±0.002 (L·mol^{-1}·sec^{-1}).

3) 1-Aminobutane (C_{BCPP} = 2.1·10^{-4} M):

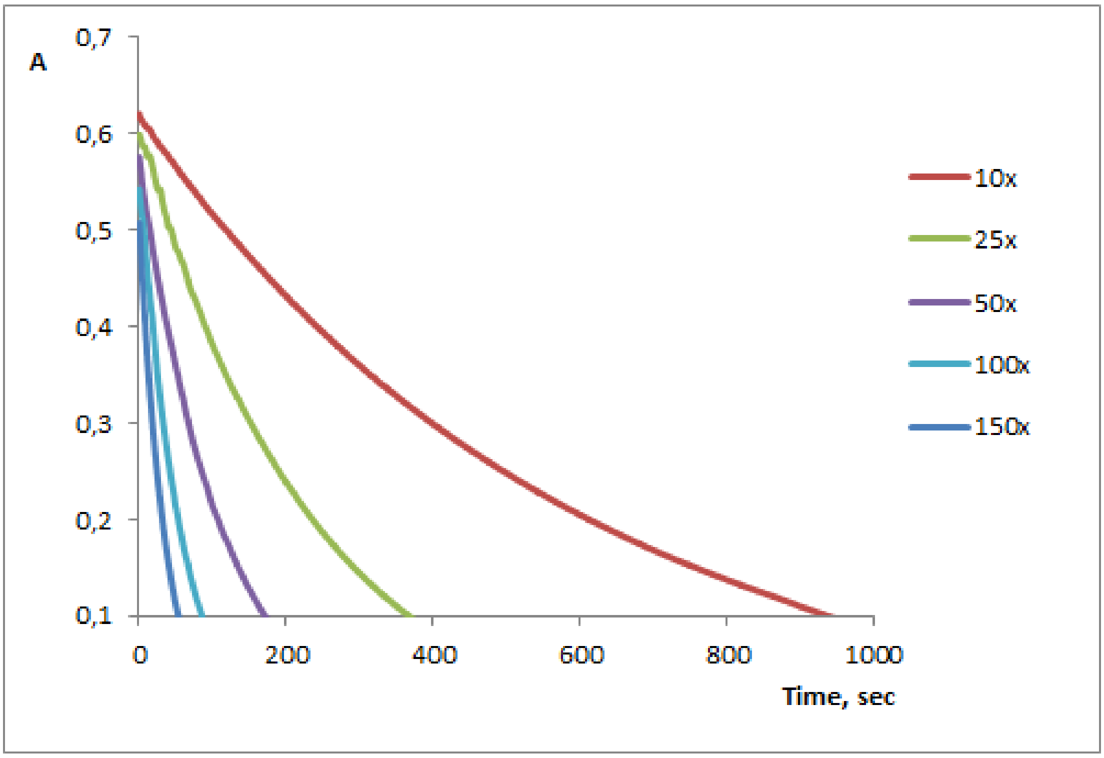

* 10x, 25x, 50x, 100x, 150x – excess of amine taken

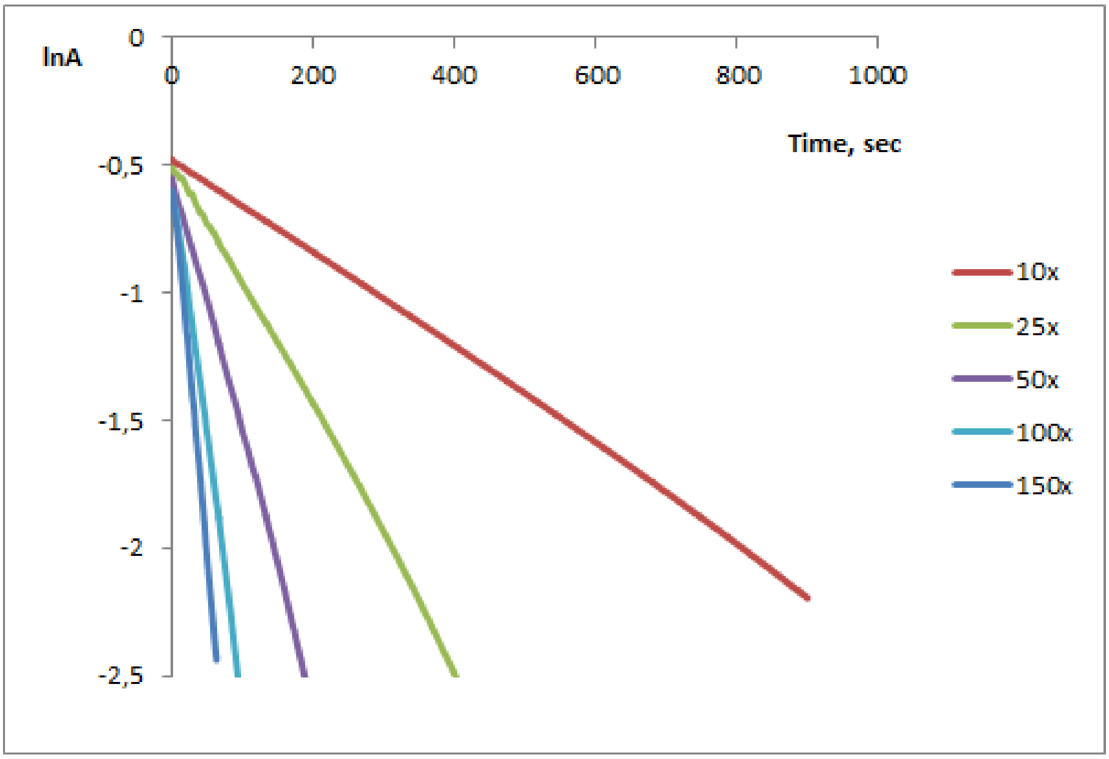

* 10x, 25x, 50x, 100x, 150x – excess of amine taken

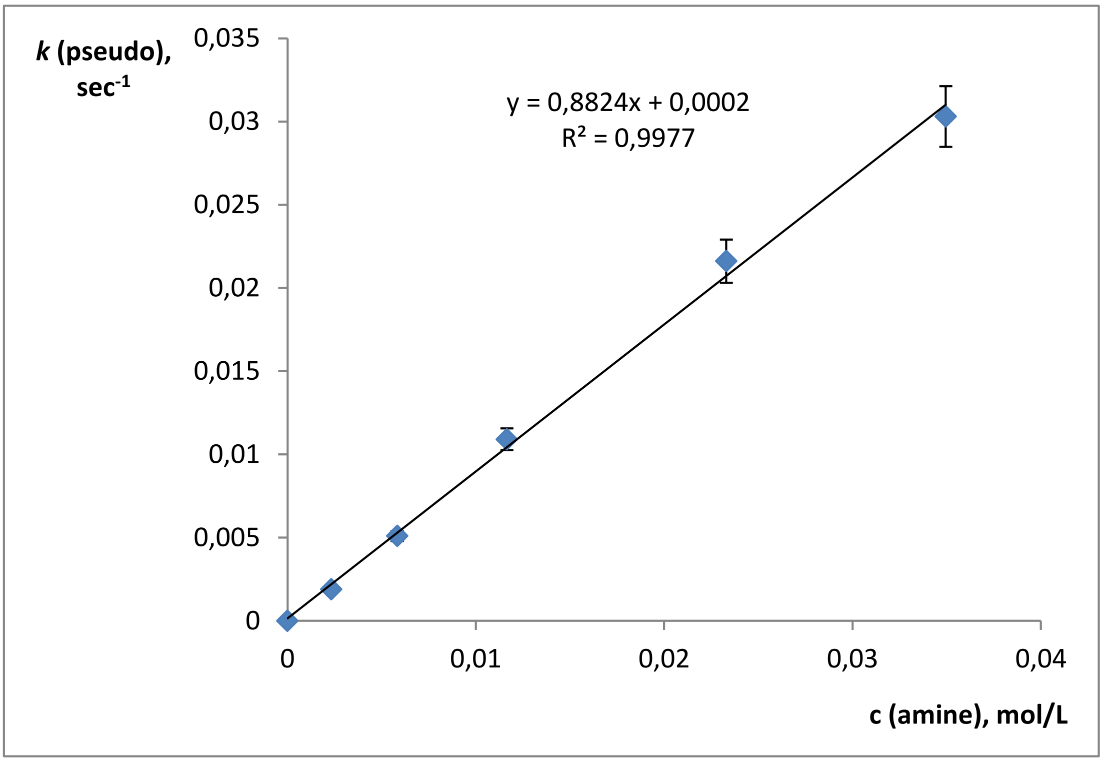

Second order rate constant in DCM at 24 ^{o}C obtained from three parallel experiments:
k = 0.86±0.04 (L·mol^{-1}·sec^{-1}).

4) 2-Methyl-1,5-diaminopentane (C_{BCPP} = 1.9·10^{-4} M):

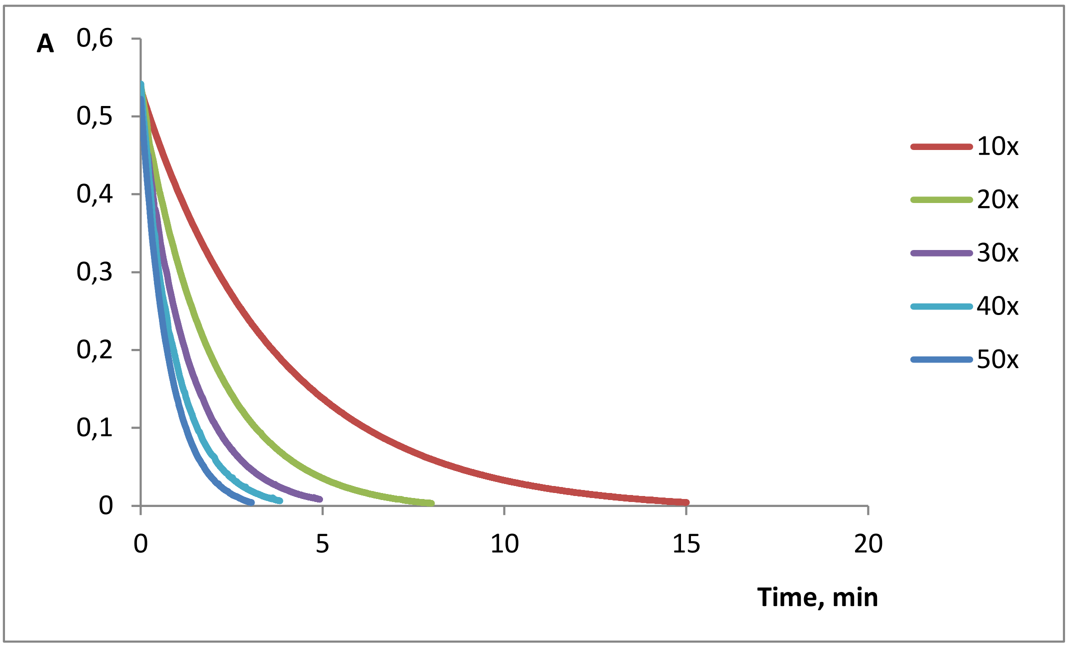

* 10x, 20x, 30x, 40x, 50x – excess of amine taken

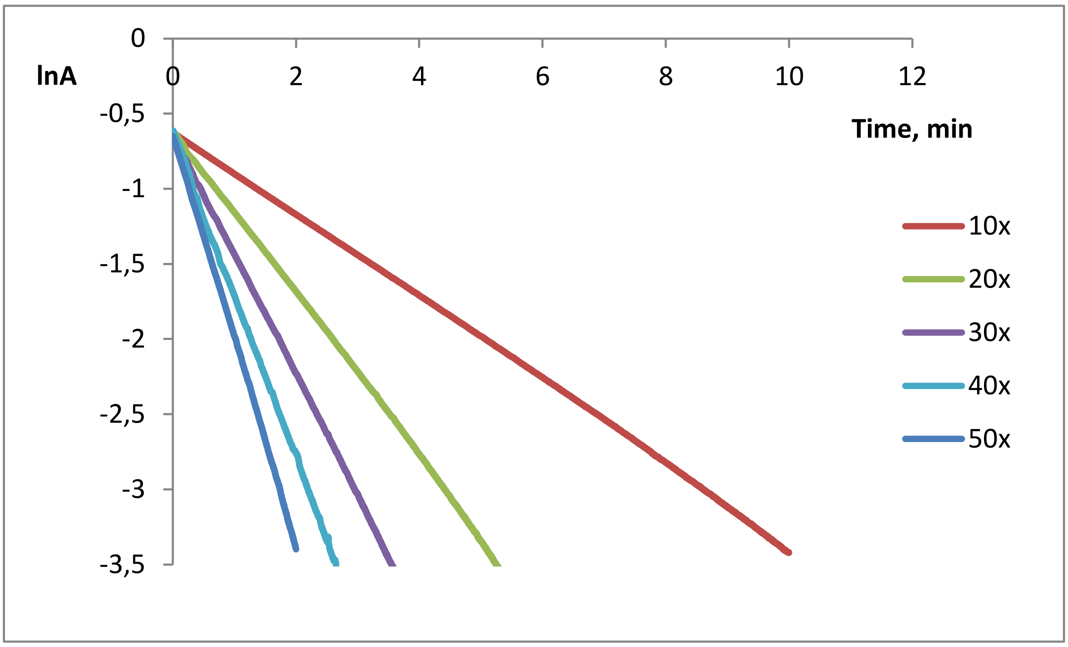

* 10x, 20x, 30x, 40x, 50x – excess of amine taken

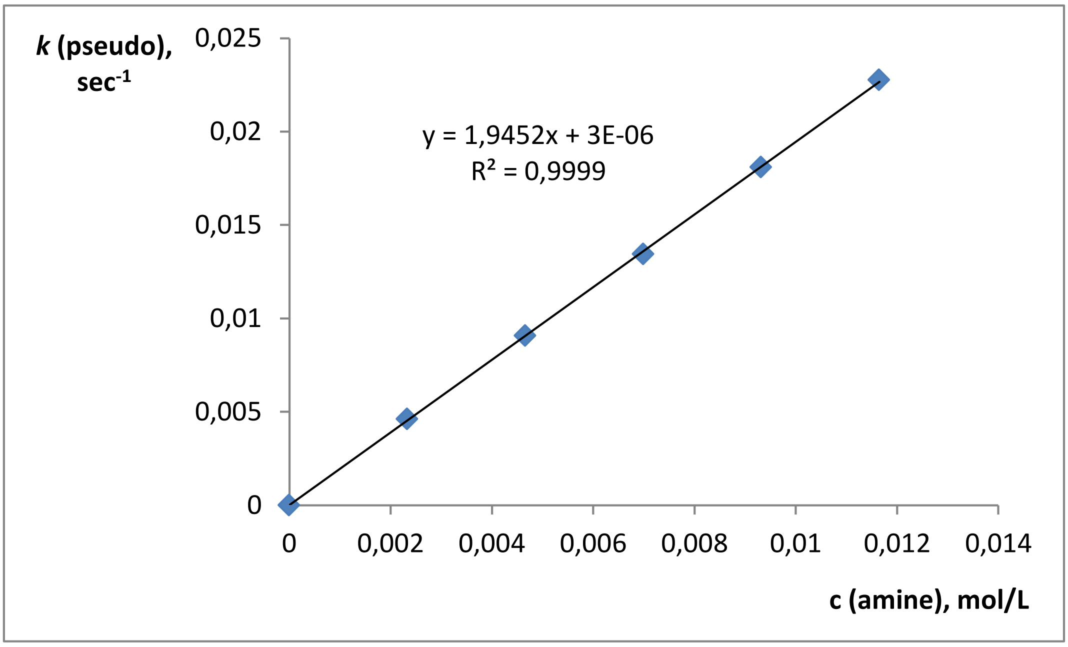

Second order rate constant in DCM at 24 °C obtained from three parallel experiments:
<u>k = 1.92±0.04 (L·mol^{-1}·sec^{-1}).</u>

5) 1,2-Diaminopropane (C_{BCPP} = 2.2·10^{-4} M):

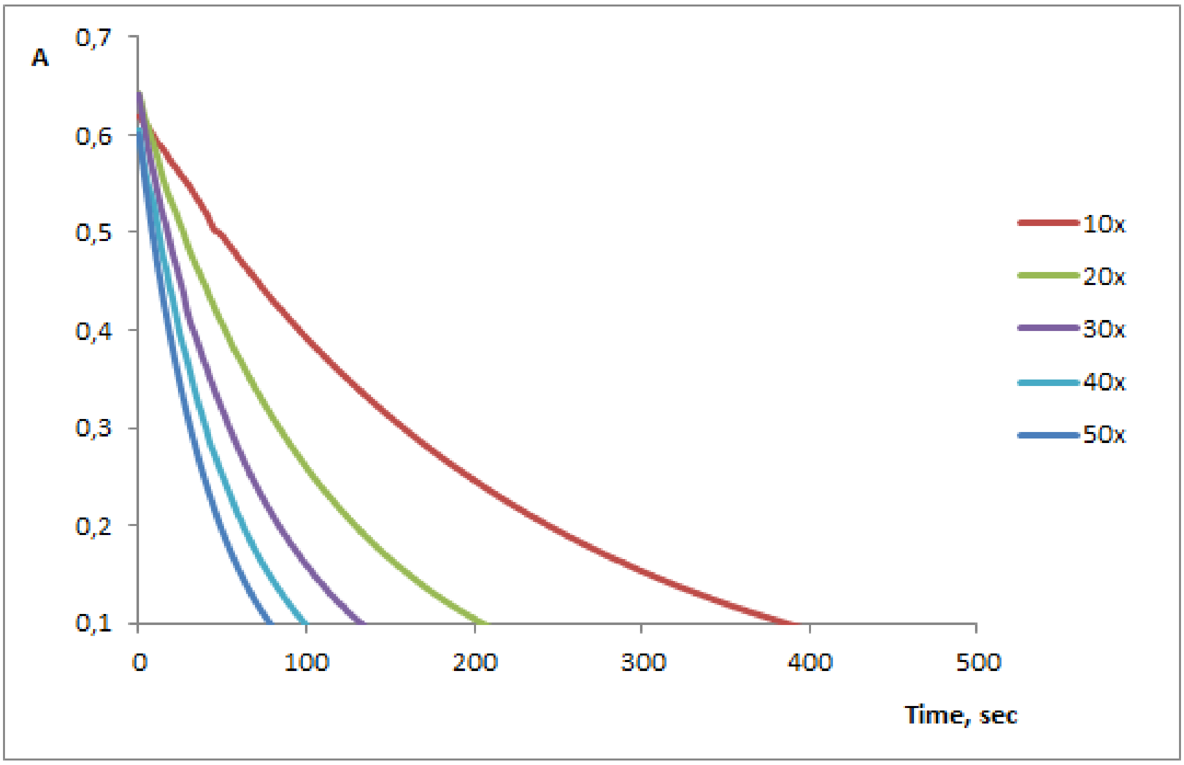

* 10x, 20x, 30x, 40x, 50x – excess of amine taken

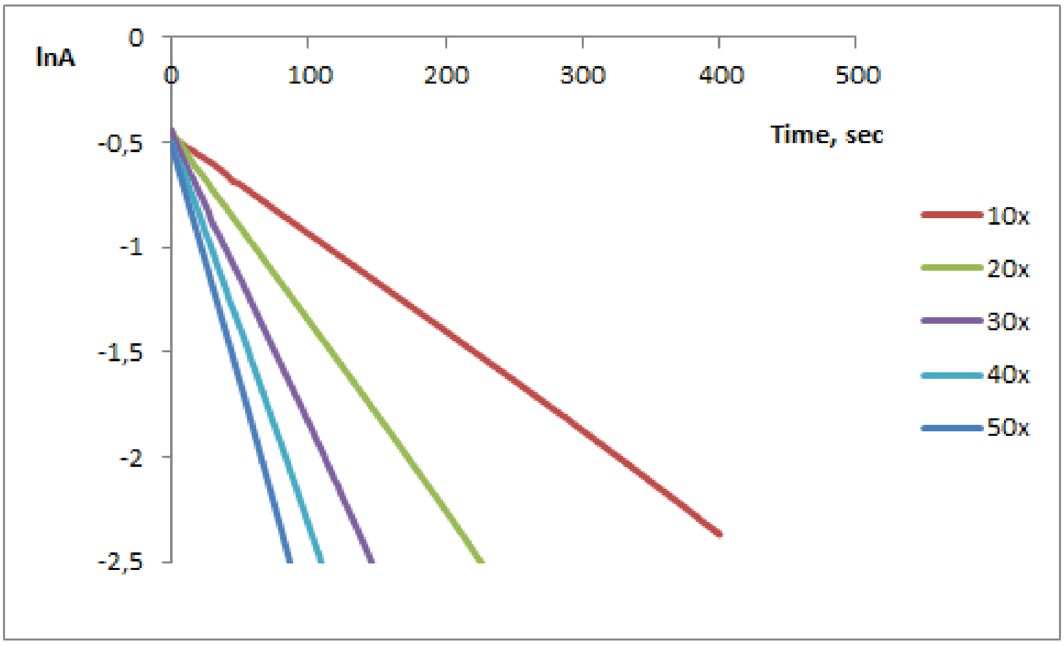

* 10x, 20x, 30x, 40x, 50x – excess of amine taken

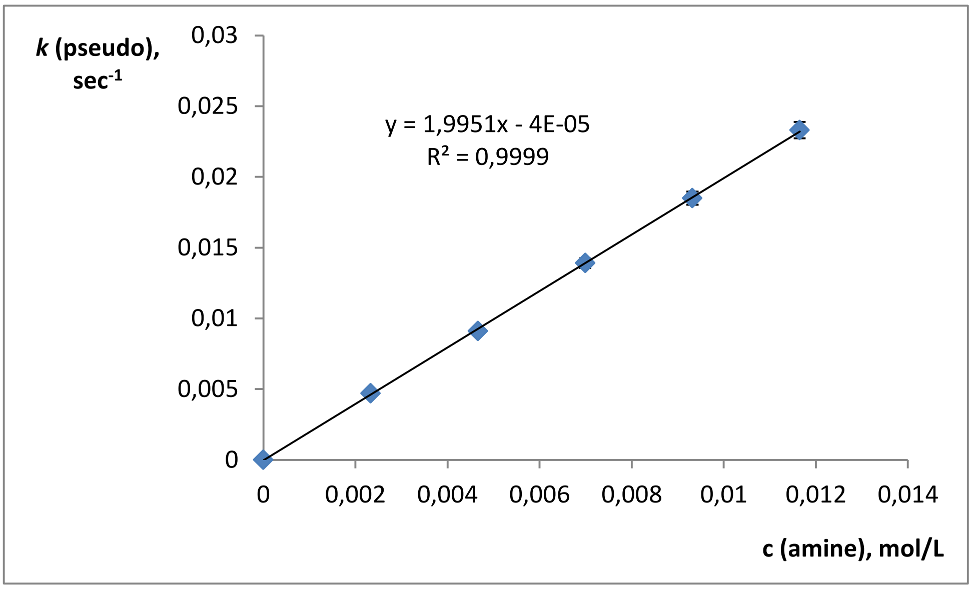

Second order rate constant in DCM at 24 ^{o}C obtained from three parallel experiments:
<u>*k* = 1.98±0.05 (L·mol^{-1}·sec^{-1}).</u>

6) 1,3-Diaminopropane (C_{BCPP} = 1.7·10^{-4} M):

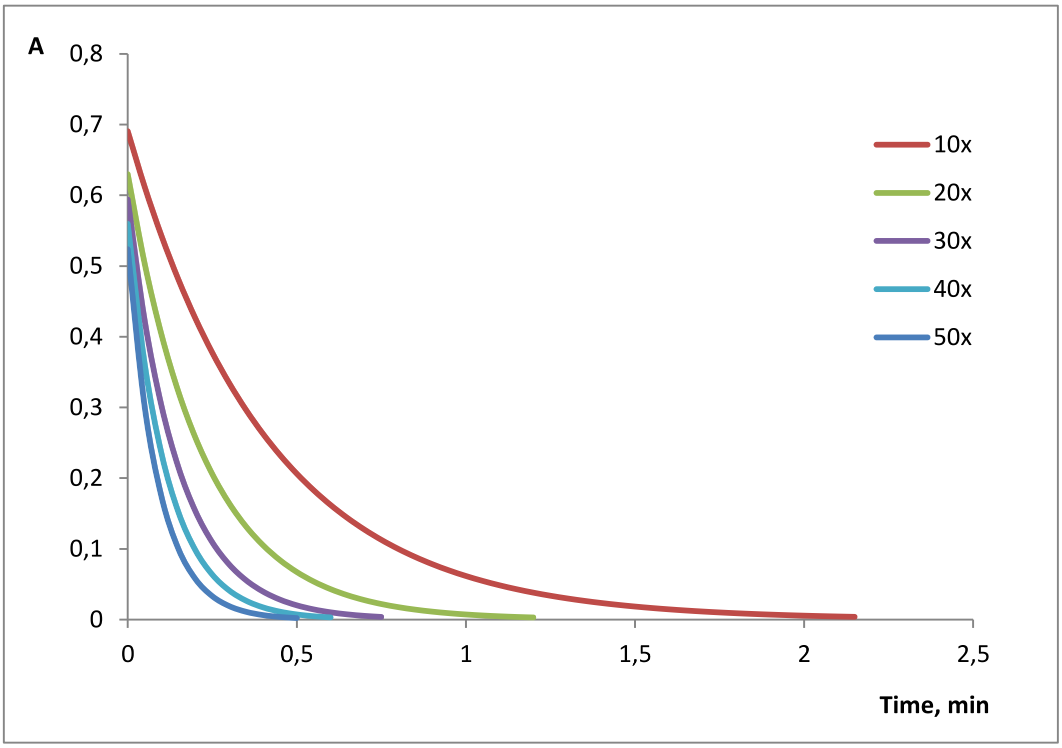

* 10x, 20x, 30x, 40x, 50x – excess of amine taken

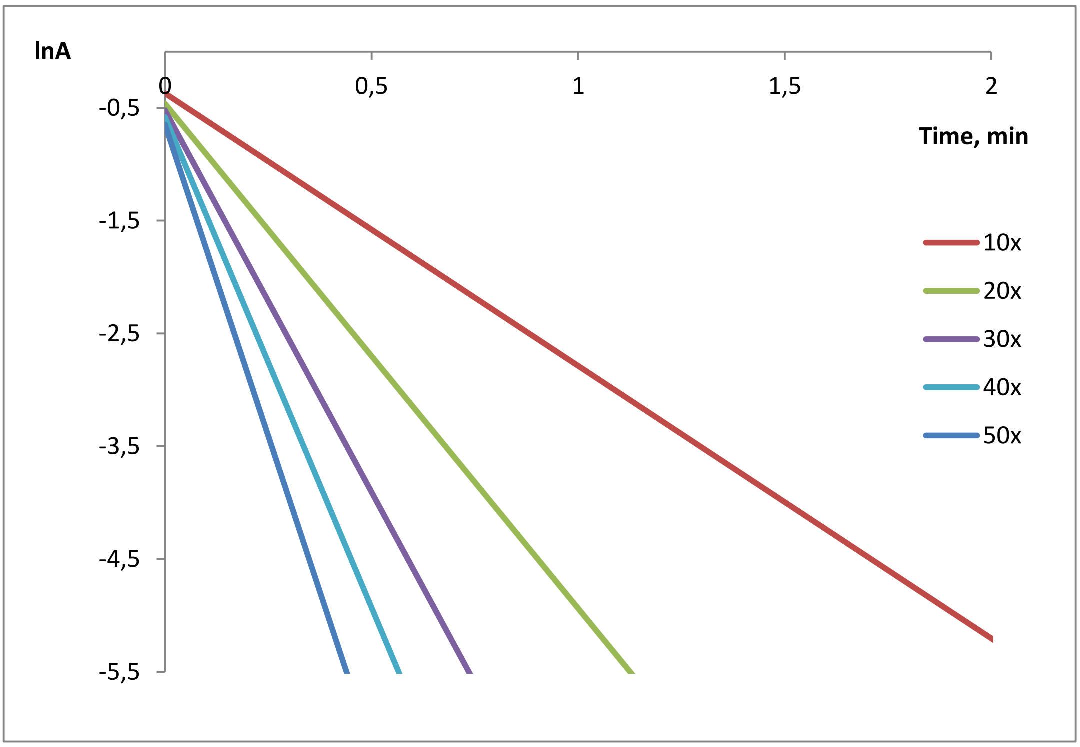

* 10x, 20x, 30x, 40x, 50x – excess of amine taken

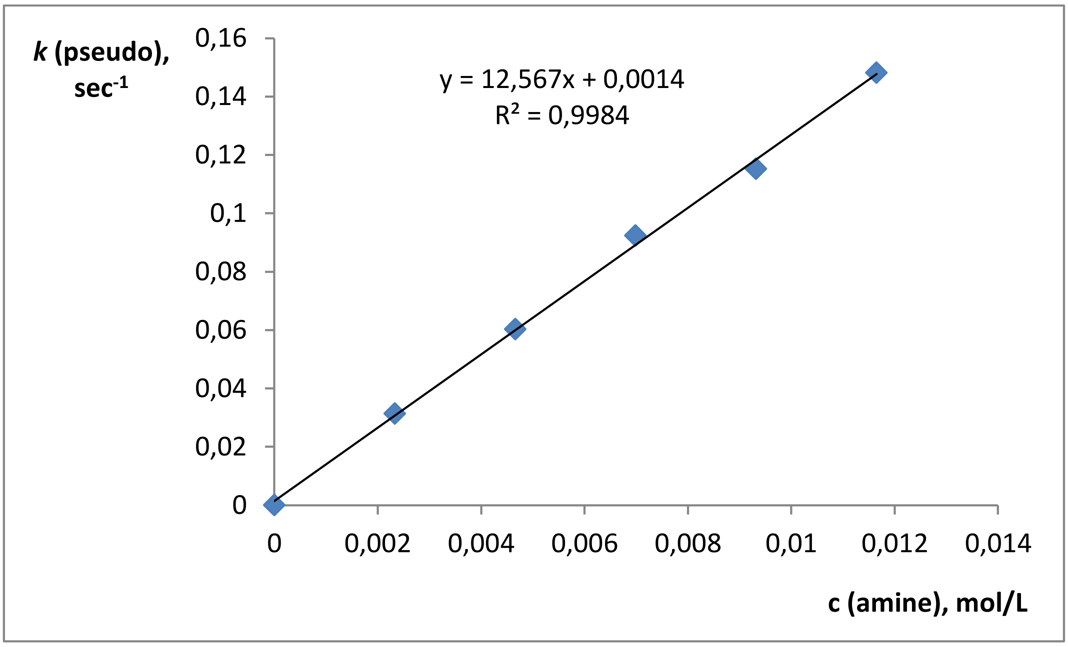

Second order rate constant in DCM at 24 °C obtained from three parallel experiments:
<u>k = 12.8±0.5 (L·mol^{-1}·sec^{-1})</u>

7) 1,3-Diaminopentane (C_{BCPP} = 2.1·10^{-4} M):

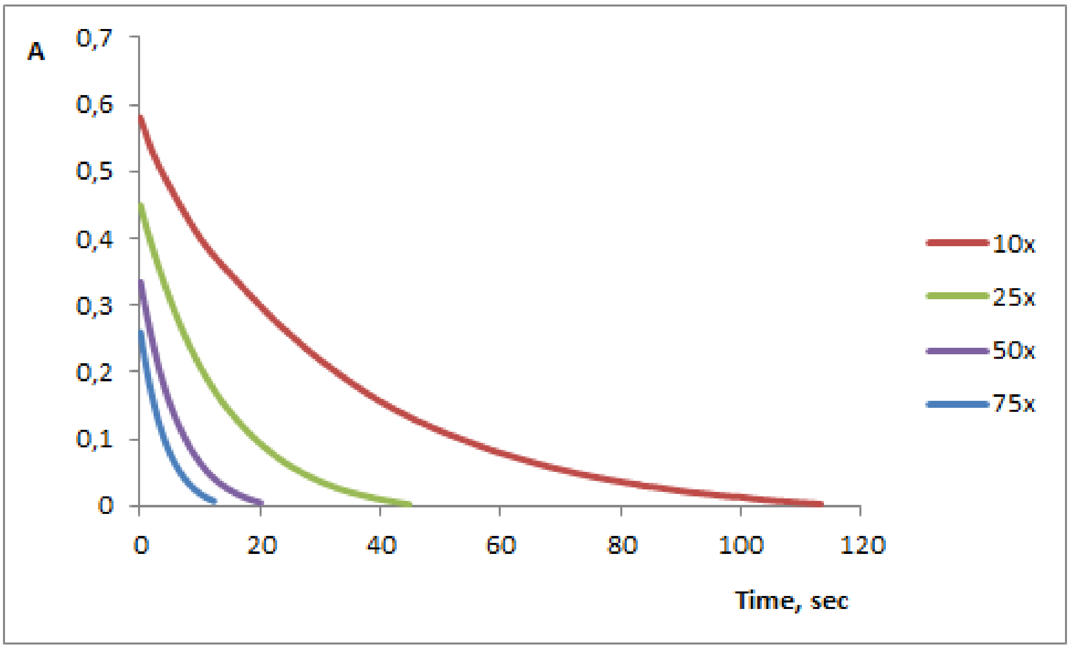

* 10x, 25x, 50x, 75x – excess of amine taken

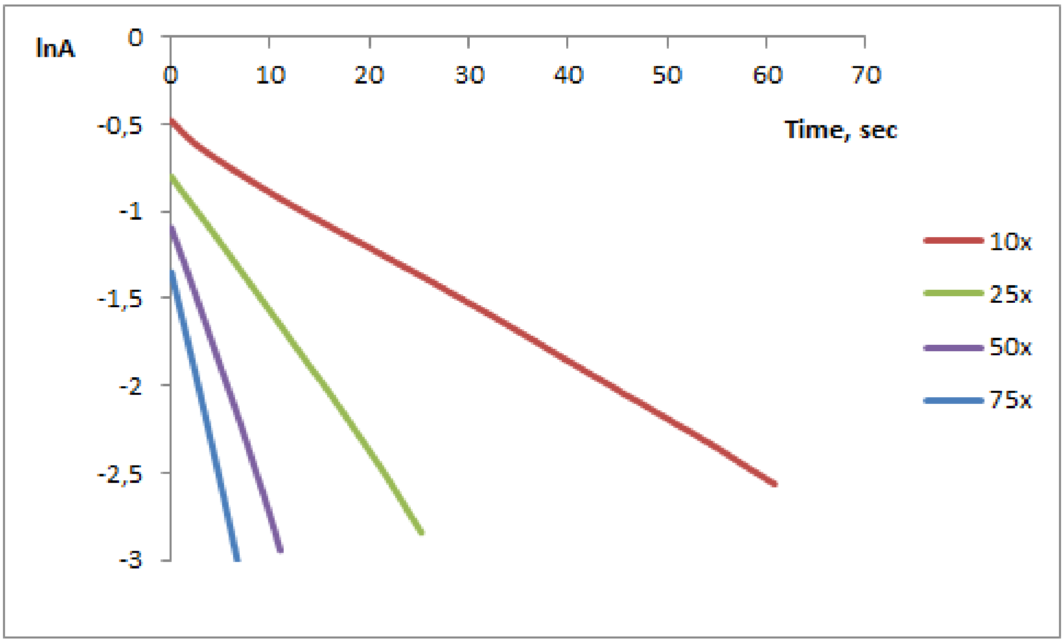

* 10x, 25x, 50x, 75x – excess of amine taken

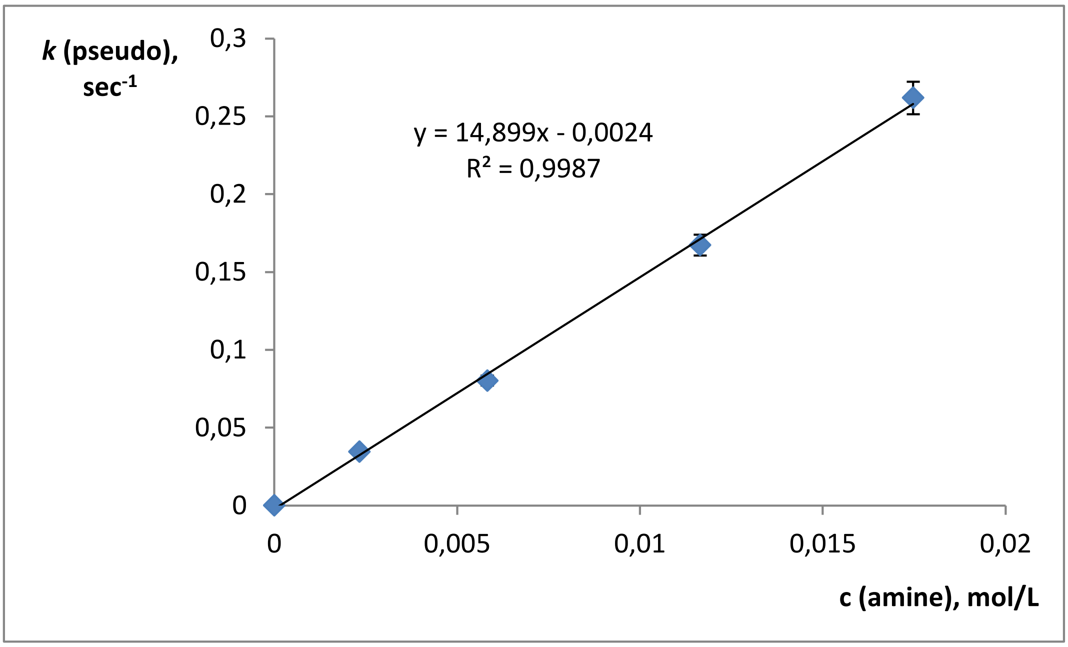

Second order rate constant in DCM at 24 ^{o}C obtained from three parallel experiments:
k = 14.9±0.3 (L·mol^{-1}·sec^{-1})

8) Diethylamine:

Because of its high basicity diethylamine forms with 4-hydroxypyrazole **4** compound **4**-Et_2NH, which has an absorbance at 380 nm.

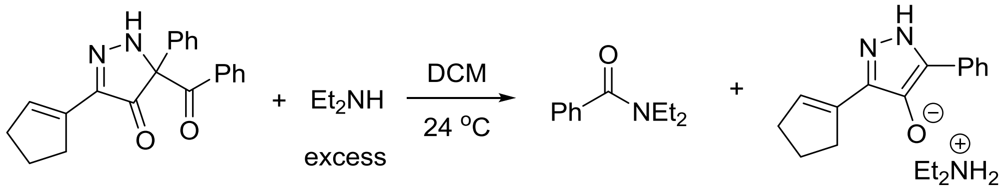

From UV-Vis spectra a suitable range has been found (e.g. λ = 420 nm). Red line – spectrum of BCPP **1a**; black line – spectrum of **4**-Et_2NH.

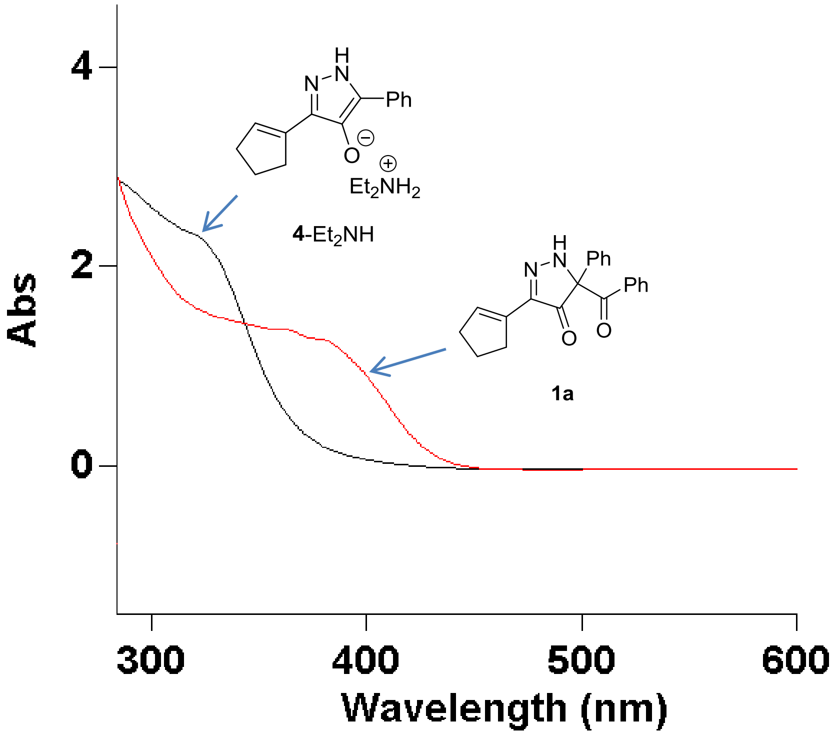

Kinetic measurements were carried out at 420 nm with a zero absorbance for the products.

$C_{BCPP} = 2.2\cdot10^{-4}$ M

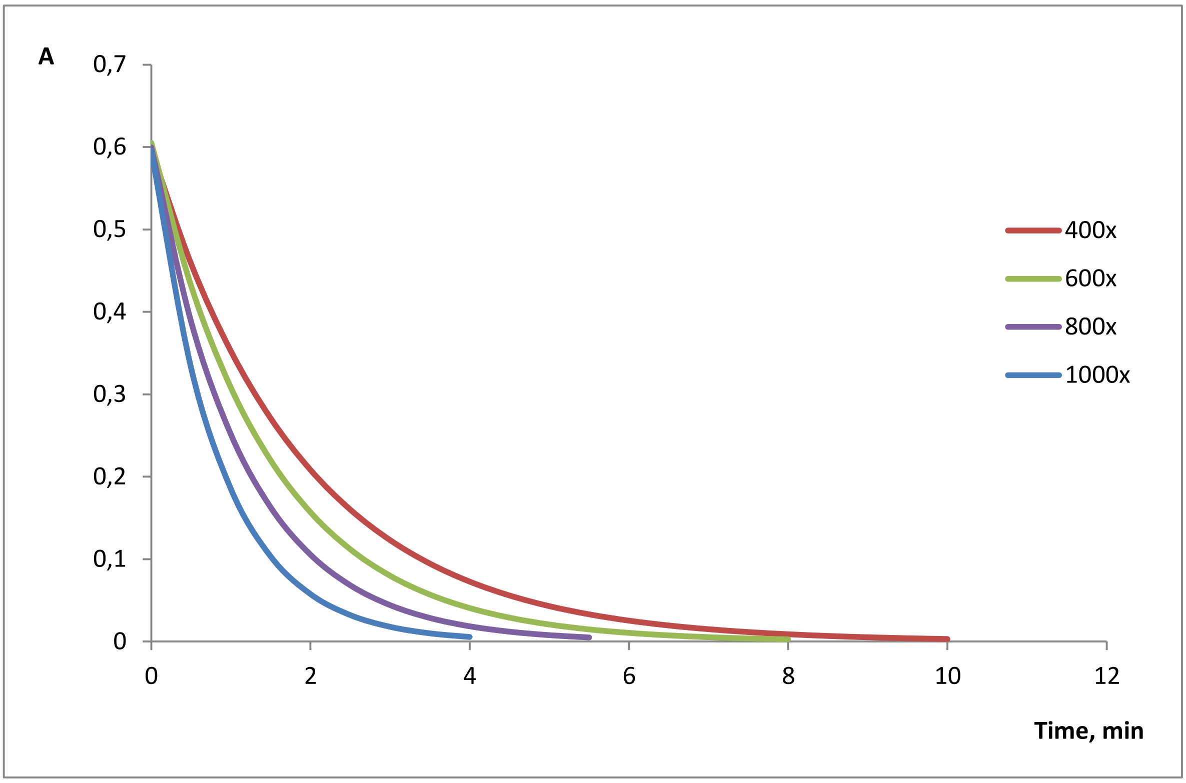

* 400x, 600x, 800x, 1000x – excess of amine taken

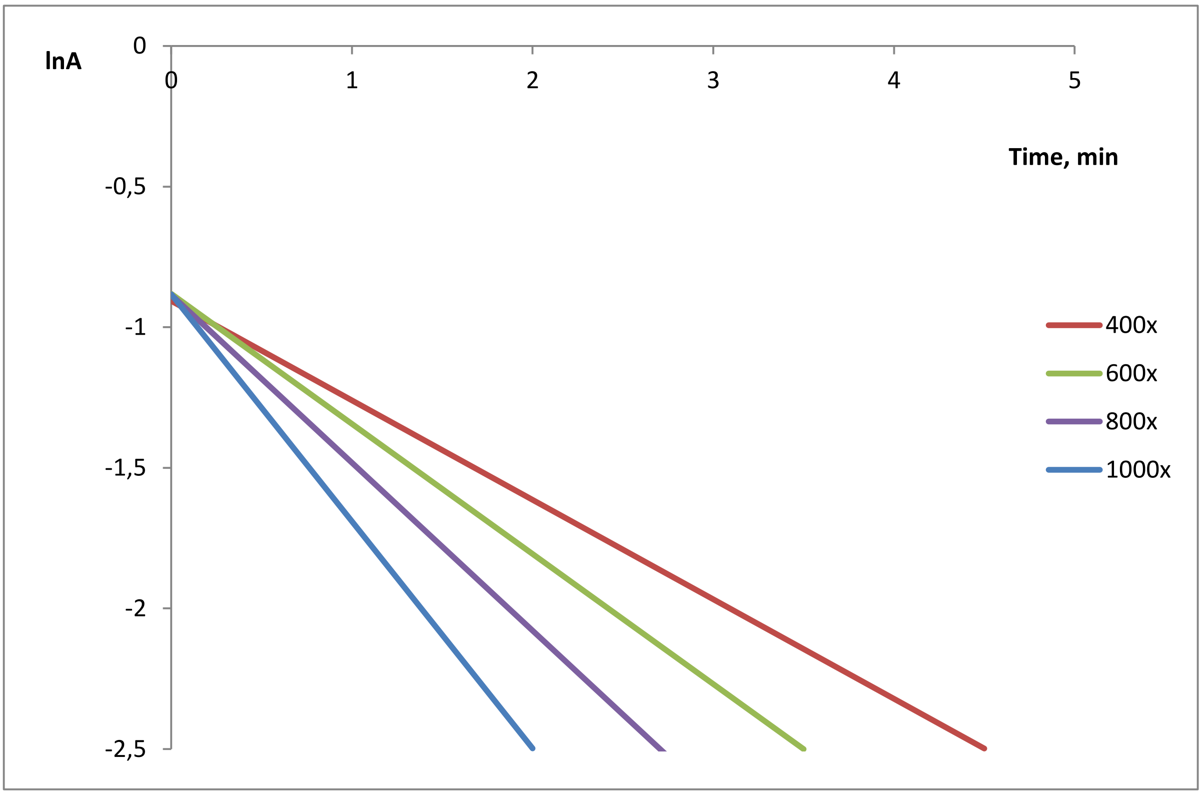

* 400x, 600x, 800x, 1000x – excess of amine taken

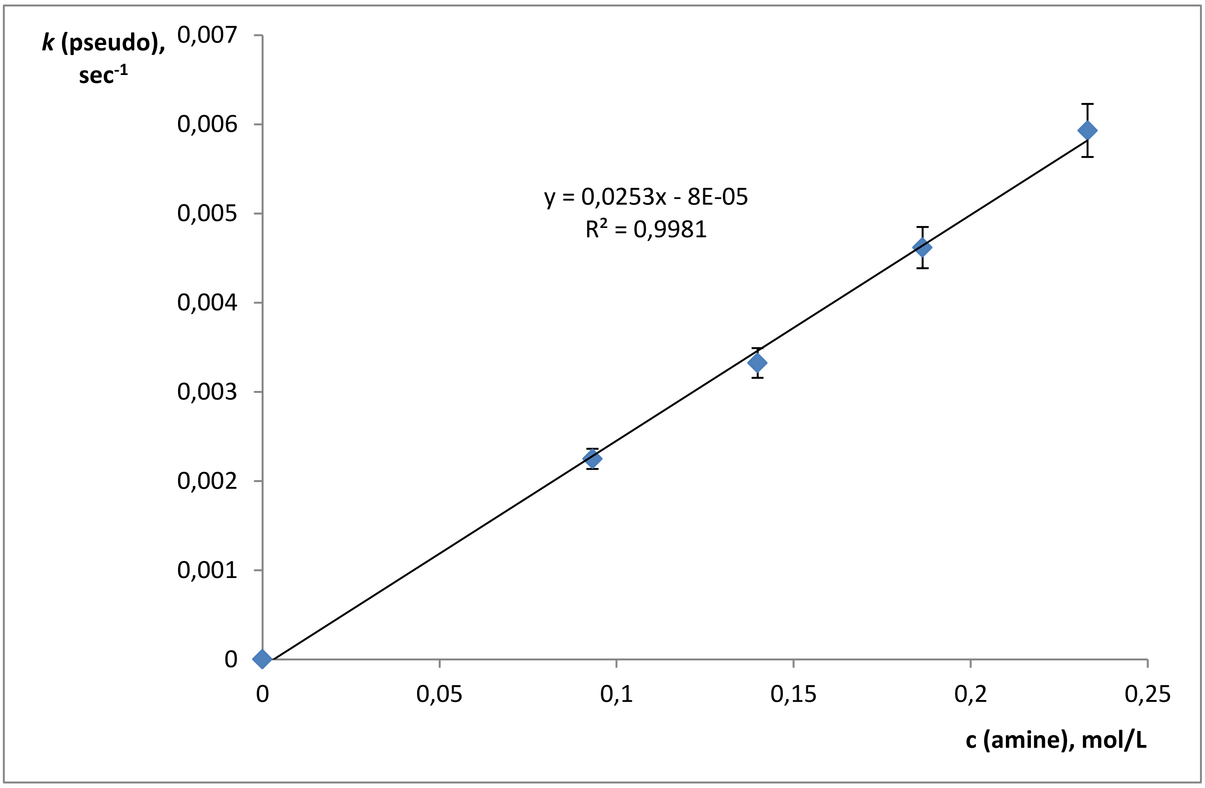

Second order rate constant in DCM at 24 °C obtained from three parallel experiments:
<u>k = 0.025±0.001 (L·mol^{-1}·sec^{-1})</u>

Substituents Effect and Hammett Plot for the Reaction of 1a–d with 1-Aminobutane

General scheme for the reaction of *para*-substituted acyl transfer reagents **1a–d** with 1-aminobutane.

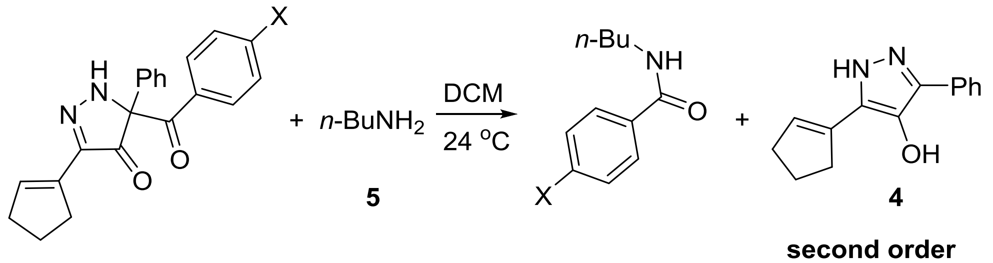

		second order rate constant (L/mol·sec)	relative rate constant
**1a** (X = H)	**6a** (X = H)	0.86	1
**1b** (X = OMe)	**6b** (X = OMe)	0.30	0.4
**1c** (X = Cl)	**6c** (X = Cl)	1.46	1.7
**1d** (X = CF_3)	**6d** (X = CF_3)	4.90	5.7

<u>Extinction coefficient determination for **1b** (X = OMe) at 380 nm:</u>

UV-Vis Spectra of 4-MeO-BCPP (**1b**):

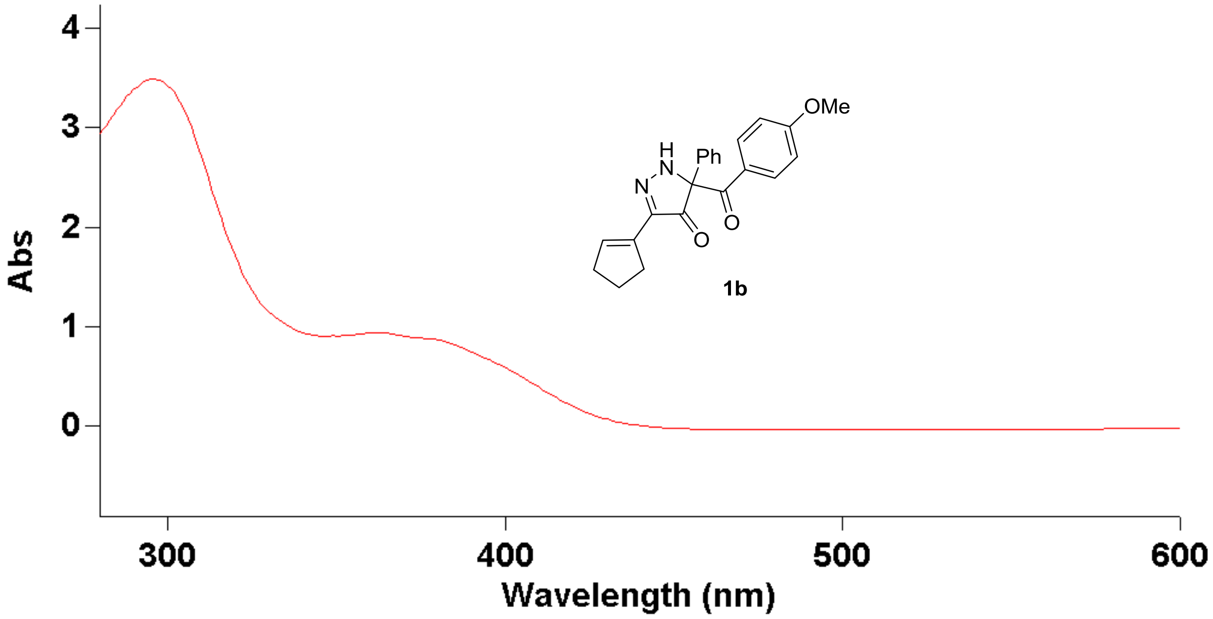

c (4-MeO-BCPP), mol/L	A
0	0
0.00010	0.3156
0.00015	0.4922
0.00020	0.6700
0.00025	0.8418
0.00030	1.0216
0.00035	1.2092
0.00040	1.3972

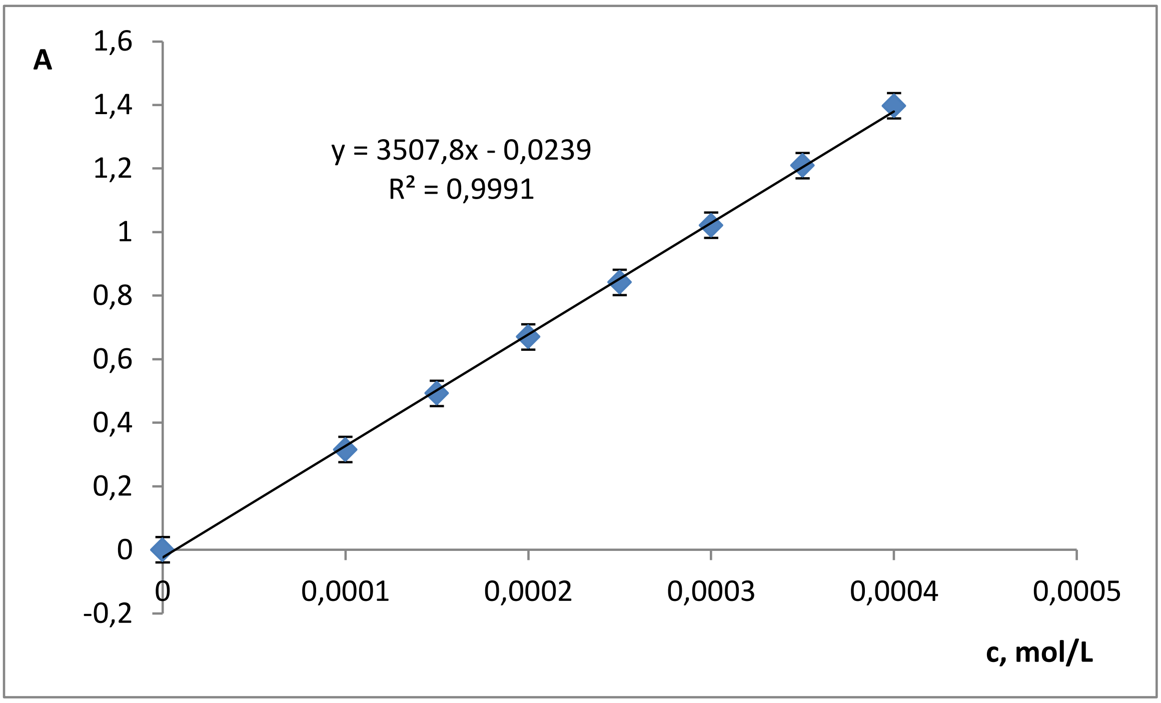

Extinction coefficient ε = 3506±93 was determined from three parallel experiments.

Kinetics of the reaction of 4-MeO-BCPP with 1-aminobutane (at 380 nm, $C_{4\text{-MeO-BCPP}} = 2.0\cdot10^{-4}$ M).

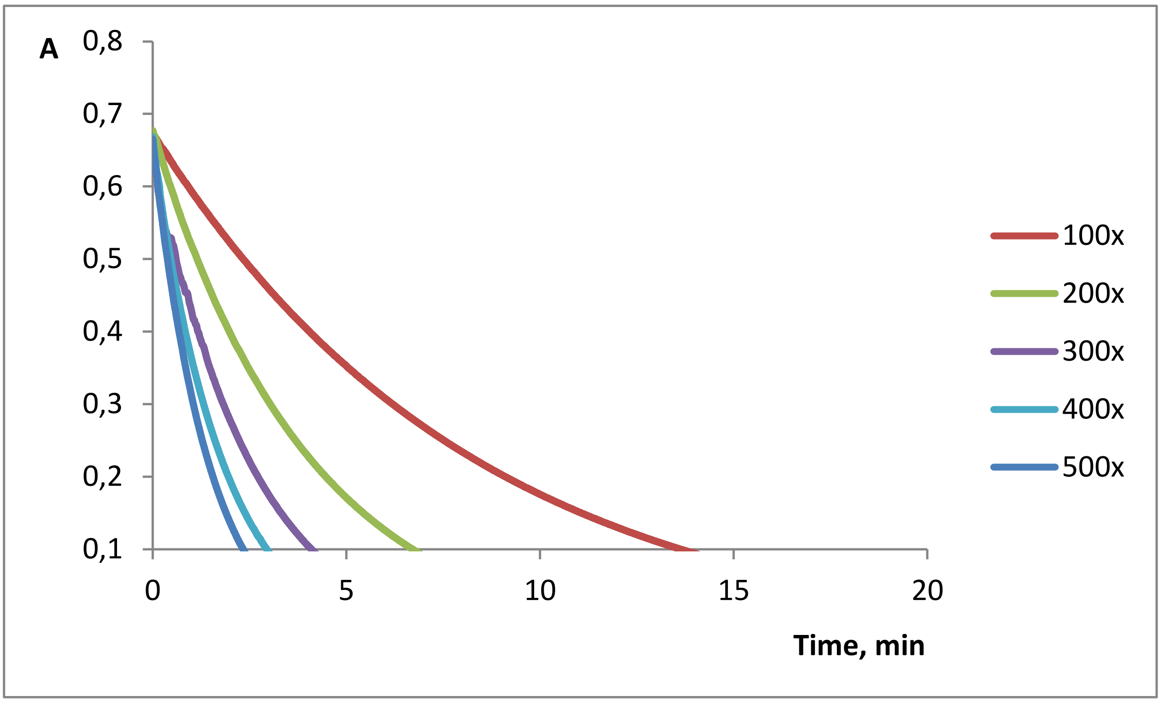

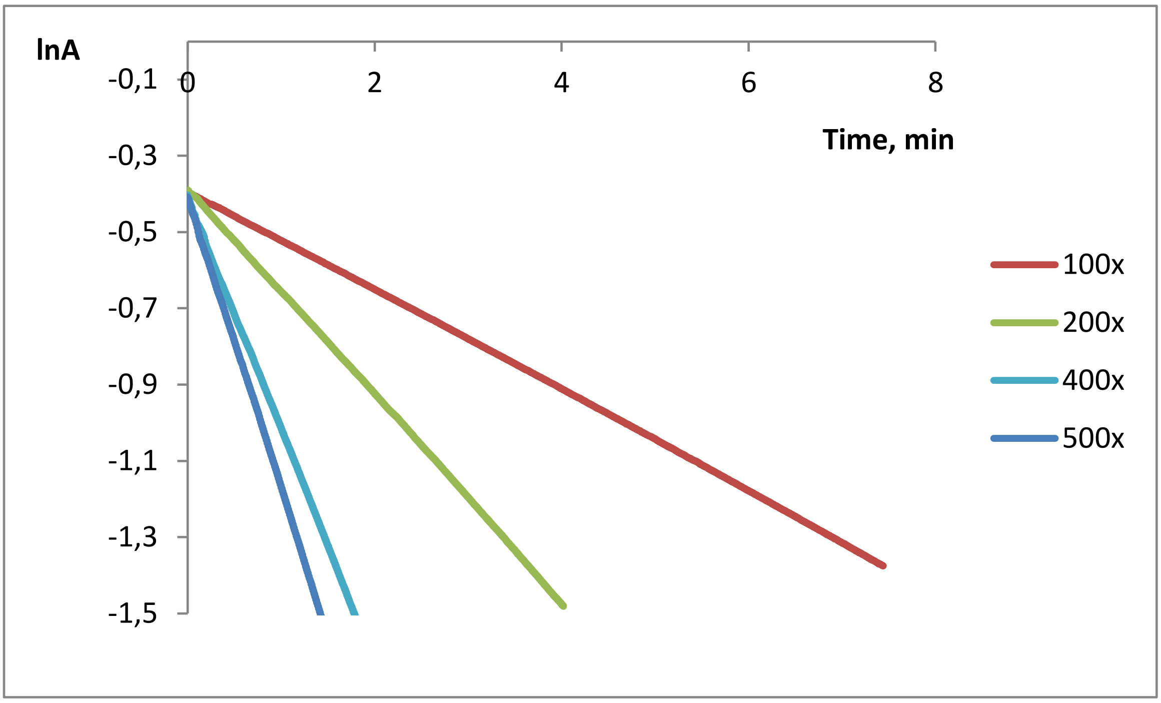

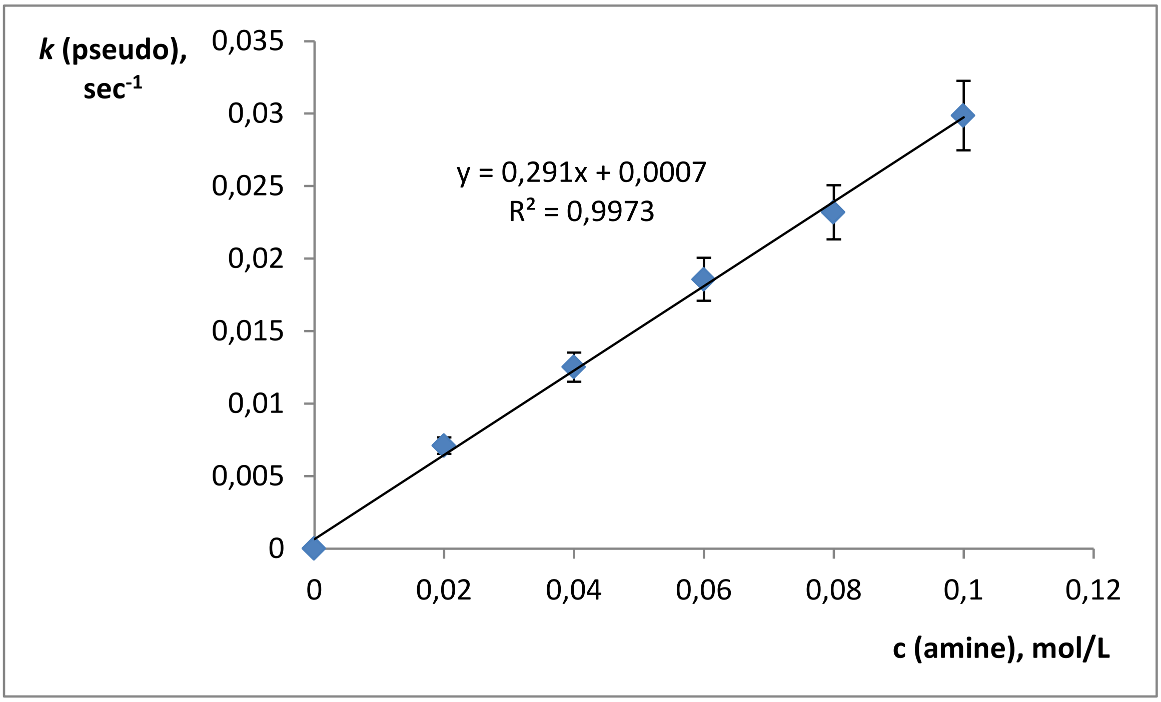

Second order rate constant in DCM at 24±0.5 °C obtained from three parallel experiments: $\underline{k = 0.30\pm0.01\ (\mathrm{L\cdot mol^{-1}\cdot sec^{-1}})}$.

Extinction coefficient determination for **1c** (X = Cl) at 380 nm:

UV-Vis Spectra of 4-Cl-BCPP (**1c**):

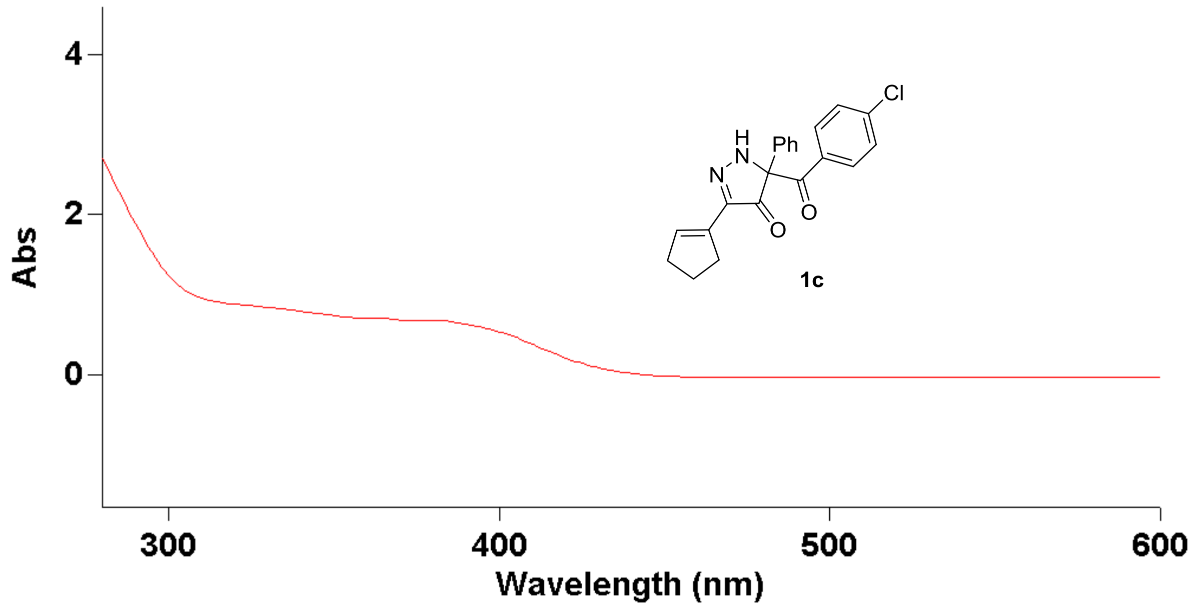

c (4-Cl-BCPP), mol/L	A
0	0
0.00005	0.1025
0.00010	0.2469
0.00015	0.3893
0.00020	0.5300
0.00025	0.6710
0.00030	0.8250
0.00035	0.9741
0.00040	1.1268

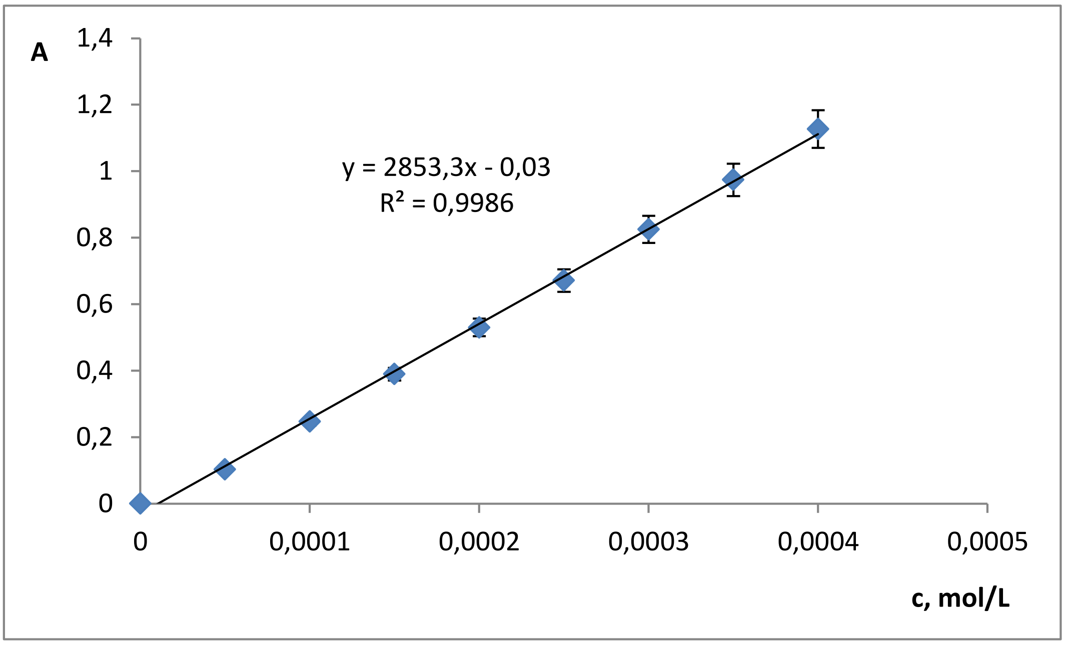

Extinction coefficient ε = 2853±65 was determined from three parallel experiments.

Kinetics of the reaction of 4-Cl-BCPP with 1-aminobutane (at 380 nm, $C_{4\text{-Cl-BCPP}}$ = 2.1·10^{-4} M).

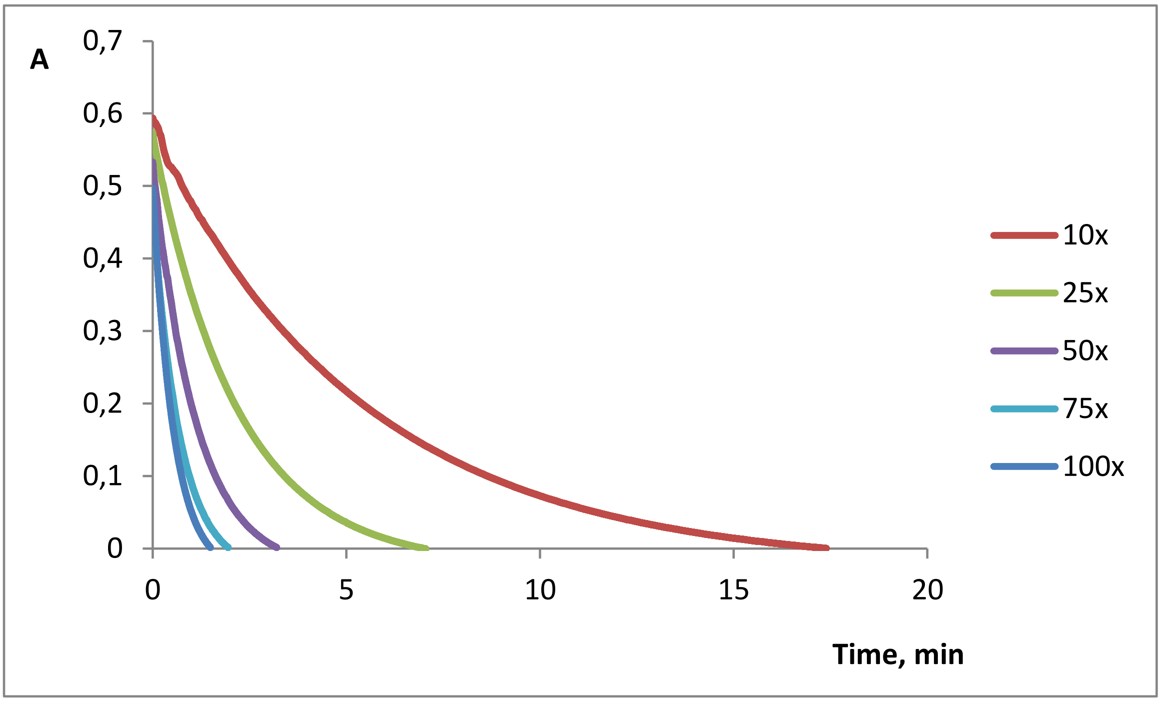

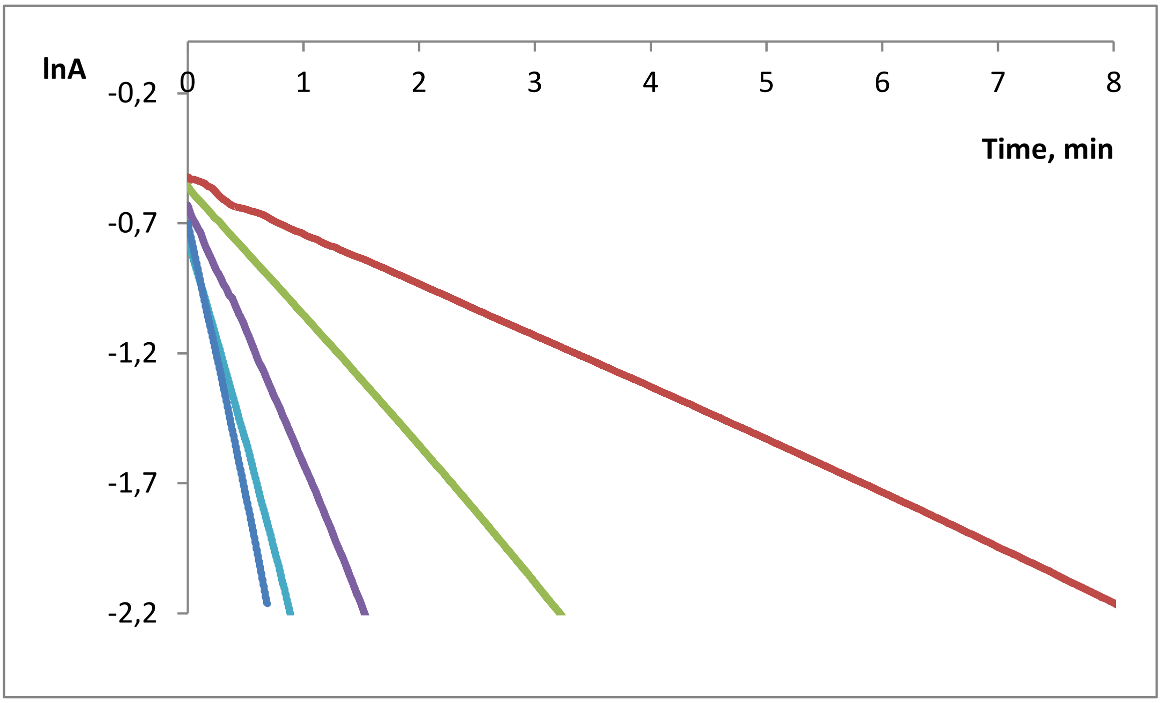

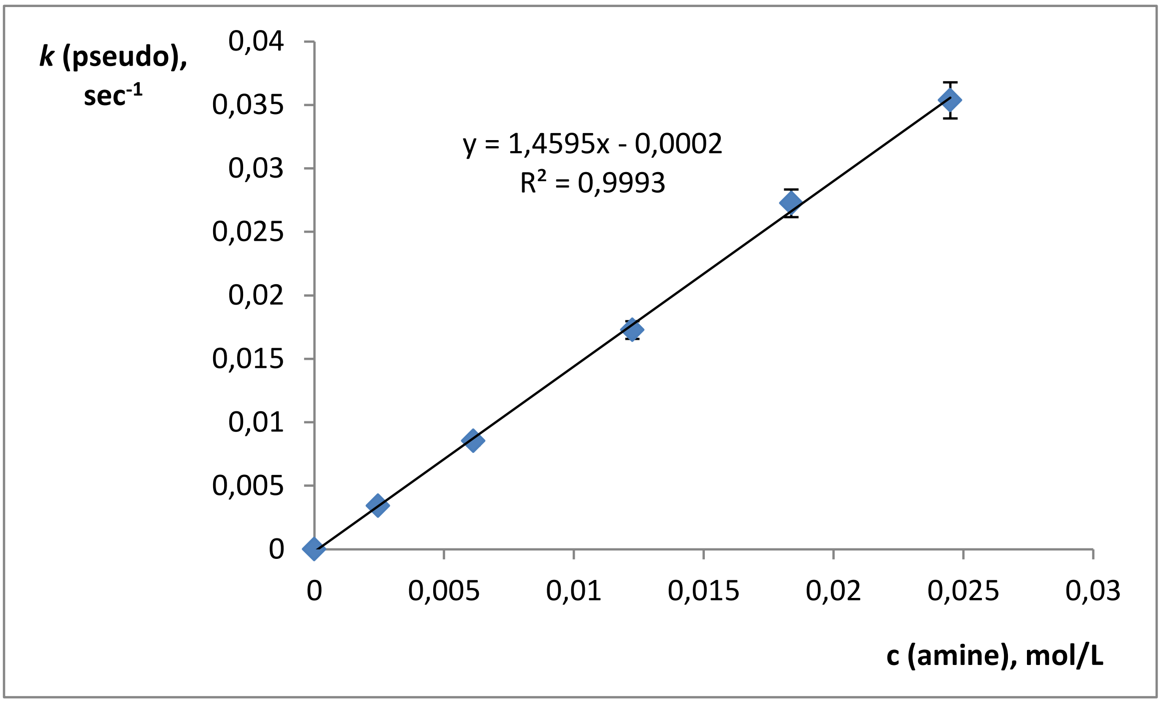

Second order rate constant in DCM at 24±0.5 °C obtained from three parallel experiments: <u>k = 1.46±0.04 (L·mol^{-1}·sec^{-1}).</u>

<u>Extinction coefficient determination for **1d** (X = CF_3) at 380 nm:</u>

UV-Vis Spectra of 4-CF_3-BCPP (**1d**):

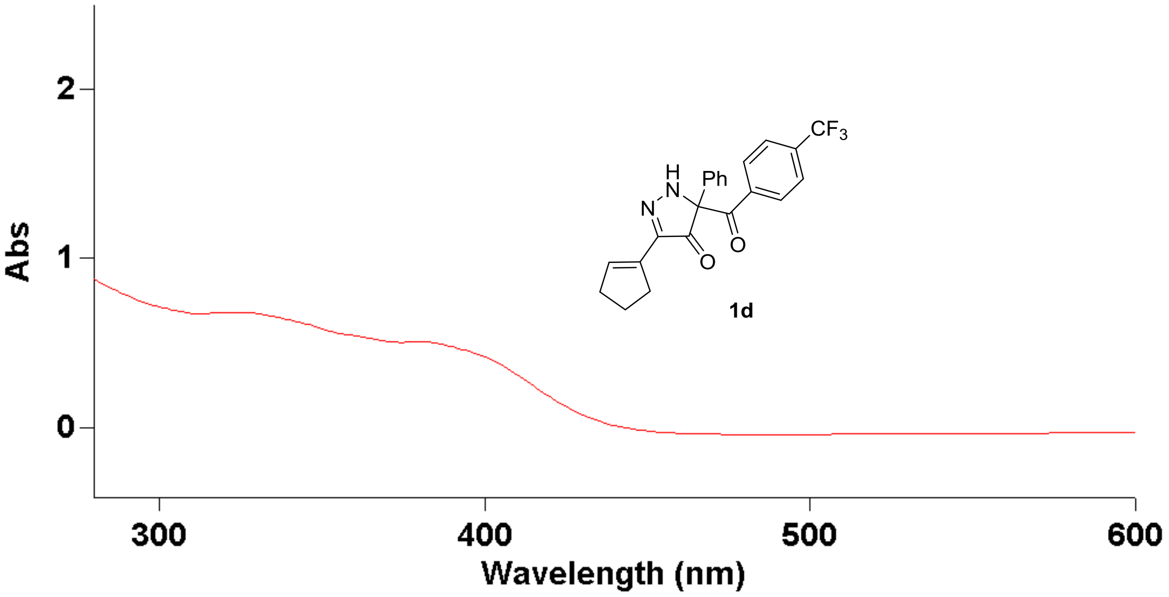

c (4-CF_3-BCPP), mol/L	A
0	0
0.00005	0.1096
0.00010	0.2381
0.00015	0.3786

0.00020	0.5028
0.00025	0.6447
0.00030	0.7578
0.00035	0.9216
0.00040	1.0096
0.00045	1.2015
0.00050	1.2960

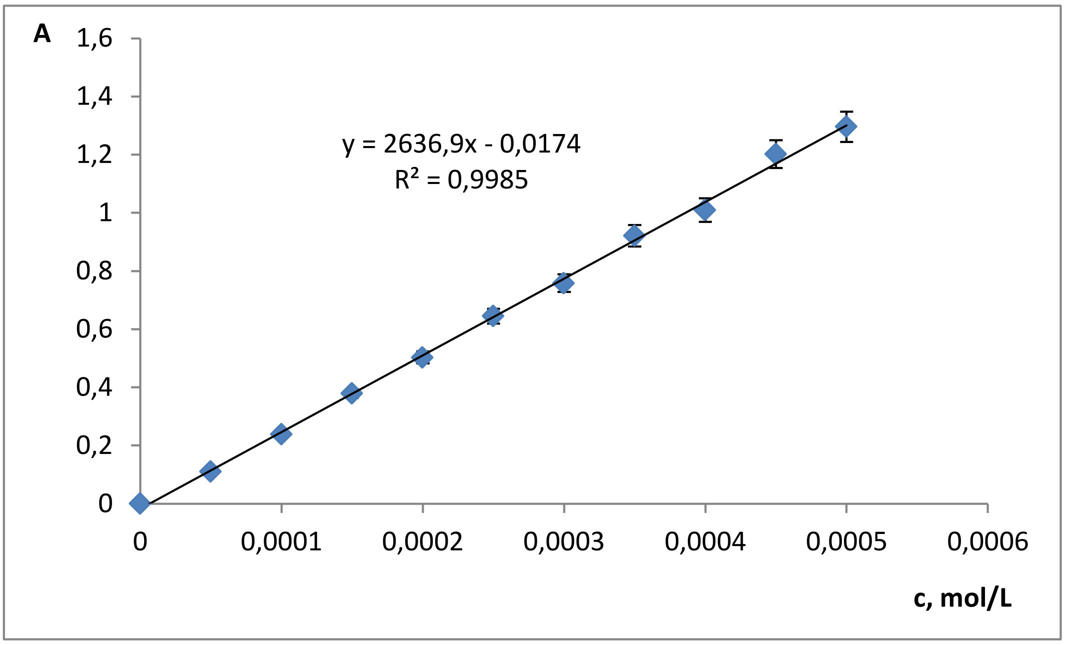

Extinction coefficient ε = 2637±50 was determined from three parallel experiments.

Kinetics of the reaction of 4-CF_3-BCPP with 1-aminobutane (at 380 nm, $C_{4\text{-}CF3\text{-}BCPP}$ = $2.6\cdot10^{-4}$ M).

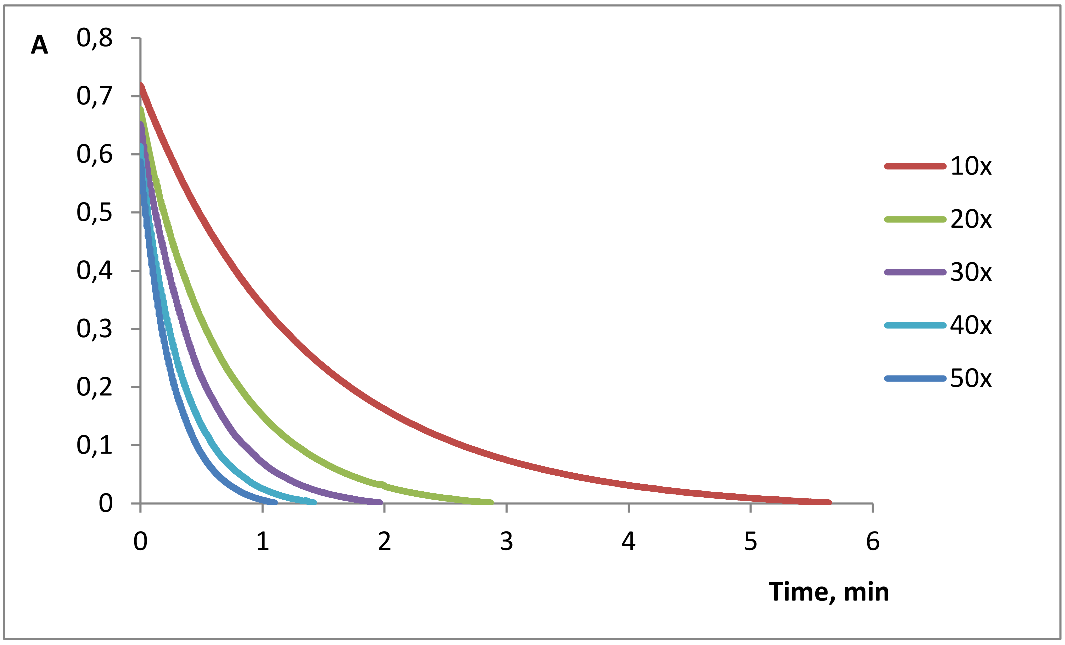

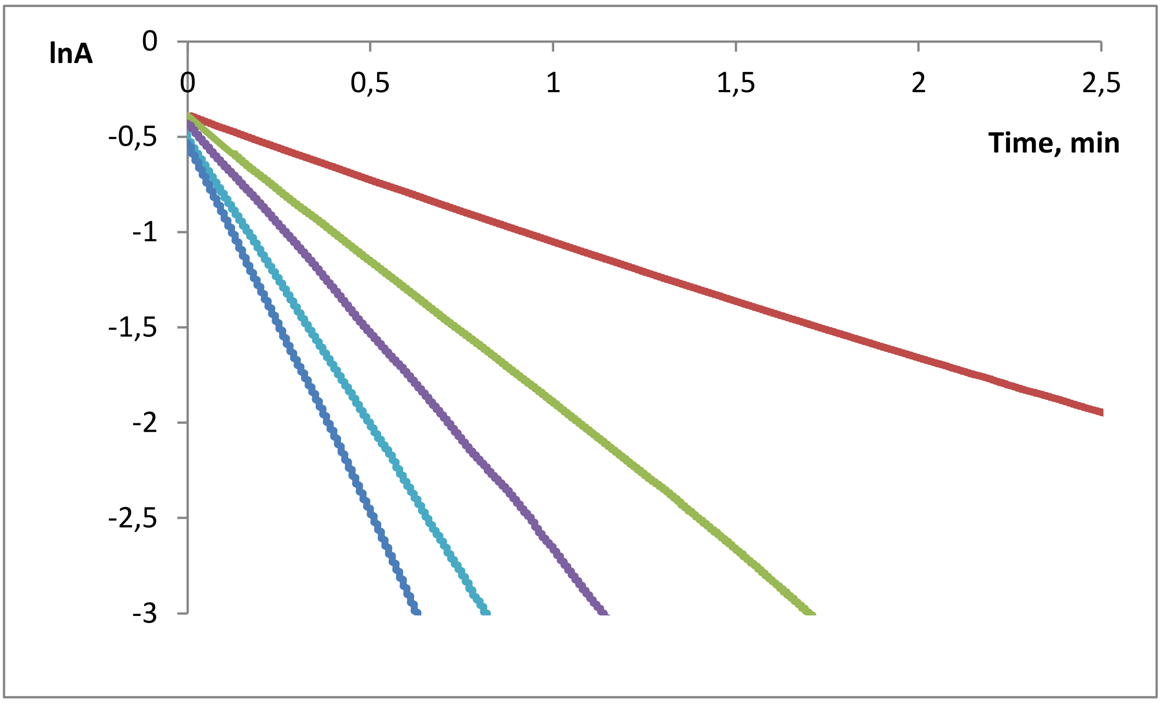

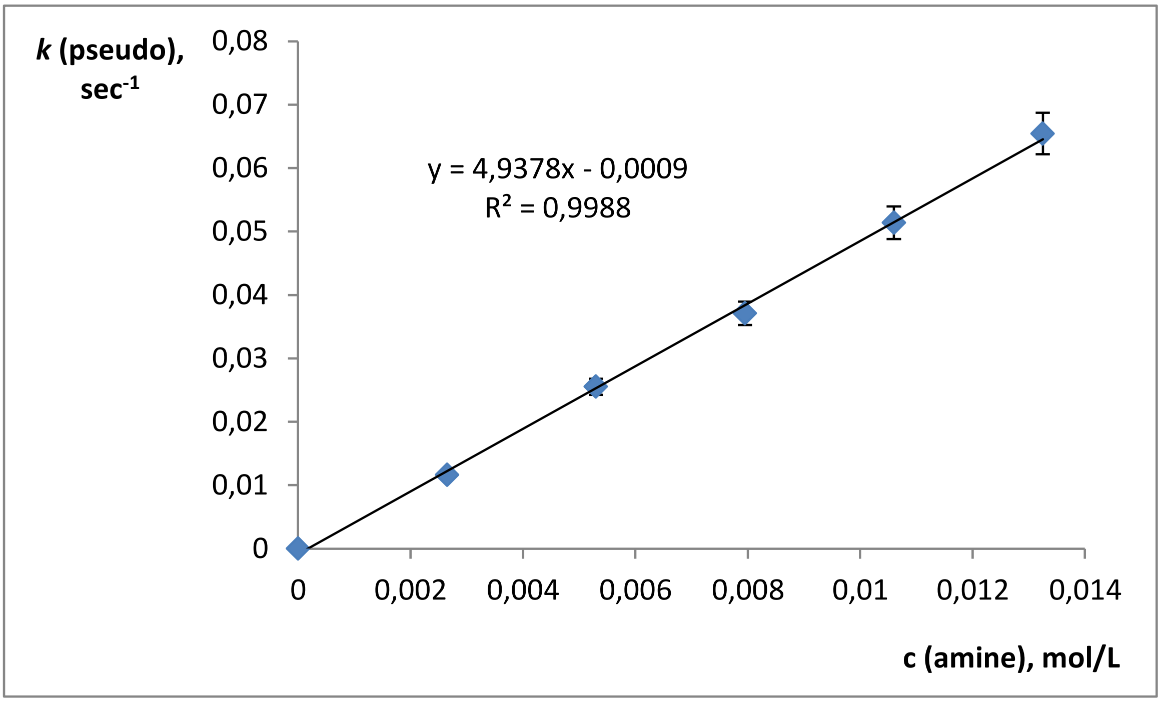

Second order rate constant in DCM at 24±0.5 ^{o}C obtained from three parallel experiments: *k* = 4.9±0.2 (L·mol^{-1}·sec^{-1}).

Hammett plot for the reaction of 1a–d with 1-aminobutane (ρ = 1.42)

σ	$\log(k_x/k_H)$
-0.27	-0.417
0	0
0.23	0.2298
0.54	0.7557

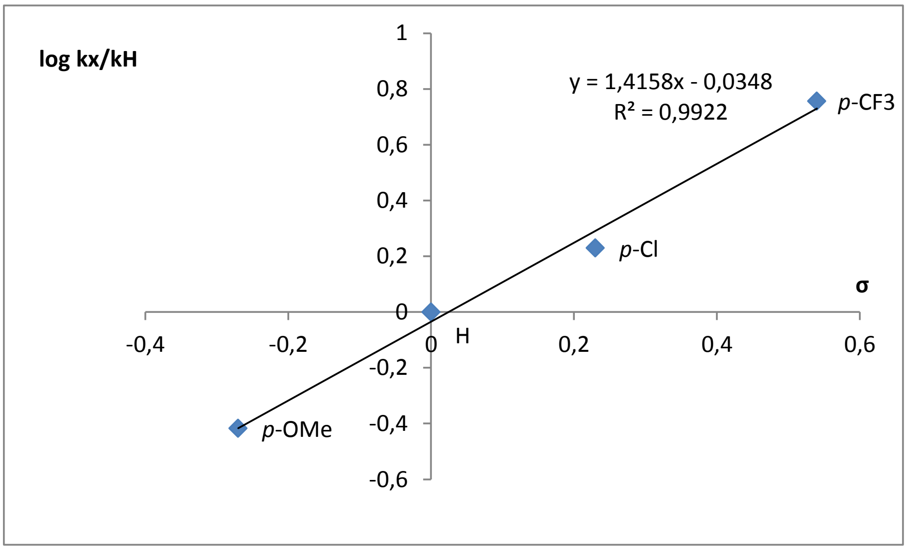

Kinetic Determination for the Intramolecular Benzoyl Transfer of BCPP

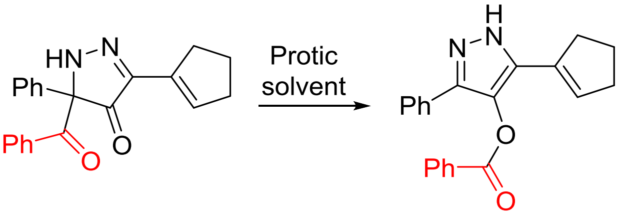

1a **44**

UV-Vis spectra of **1a** and **44** in methanol

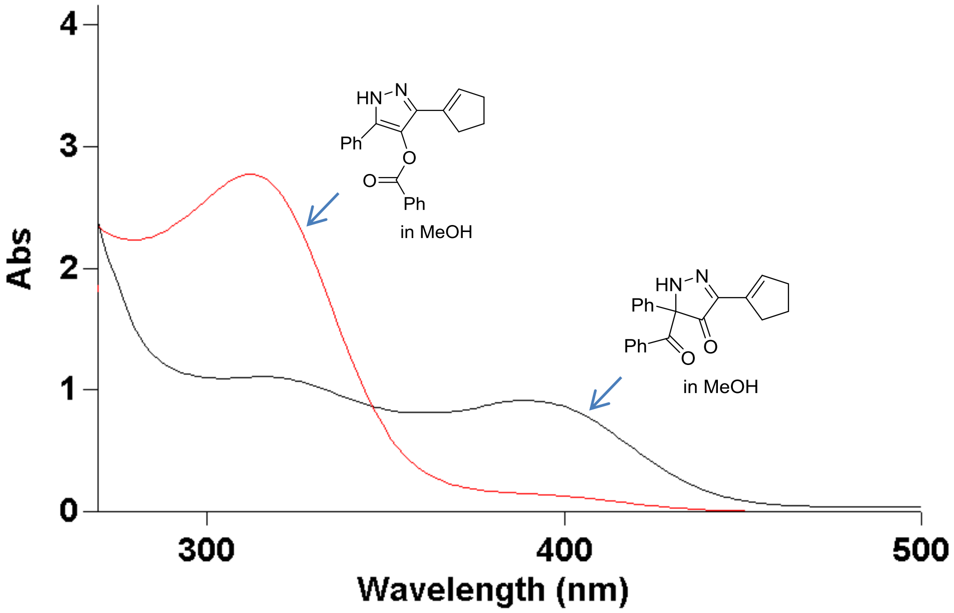

Kinetics of the rearrangement **1a** → **44** in MeOH/DCM 5:1 at 400 nm – Monitoring the disappearance of **1a**:

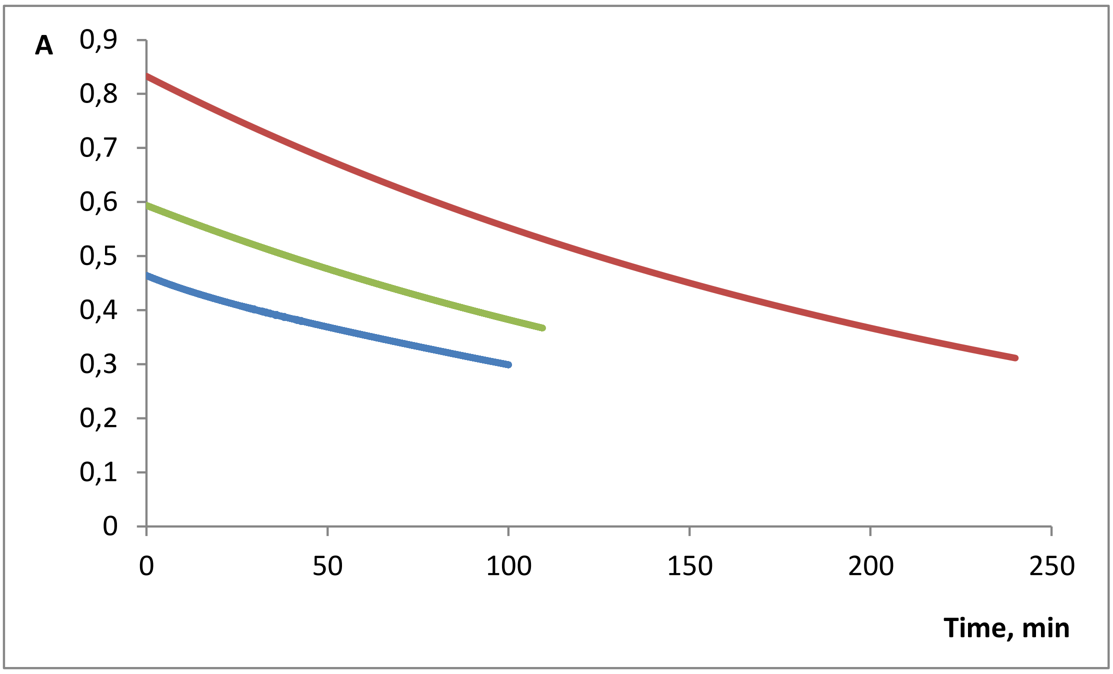

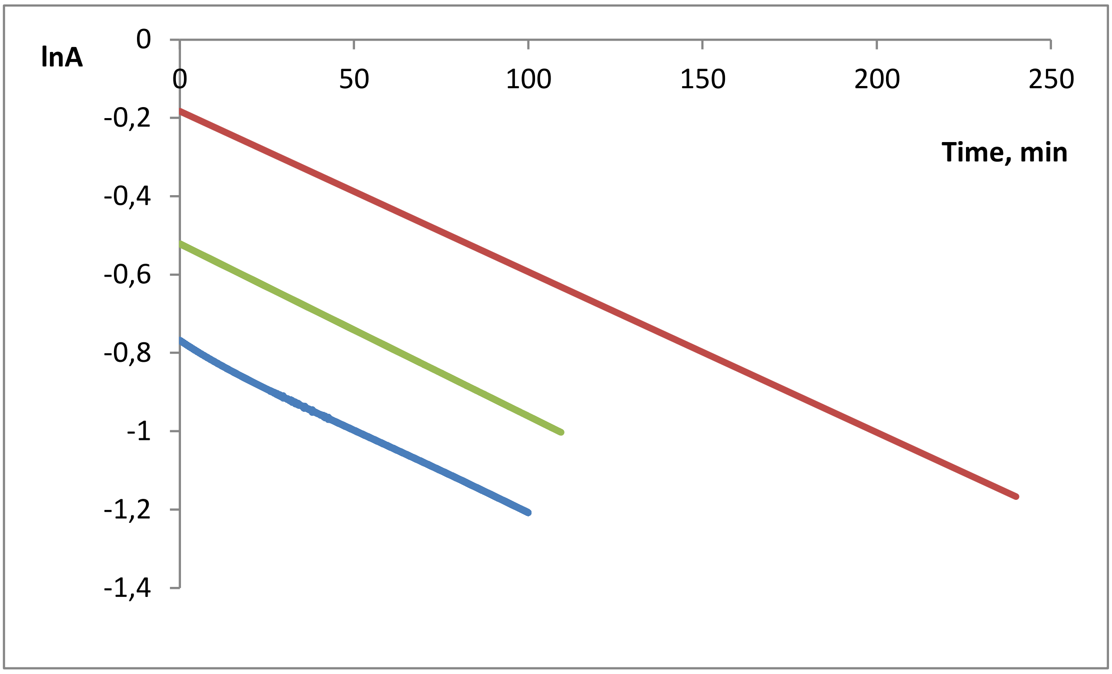

First order reaction rate constants determined at different concentrations of BCPP:

$k = 7.33 \cdot 10^{-5}$; $7.16 \cdot 10^{-5}$; $6.83 \cdot 10^{-5}$ sec^{-1}

<u>**$k = 7.1 \cdot 10^{-5} \pm 0.3 \cdot 10^{-5}$ sec^{-1}**</u>

UV-Vis spectra of **1a** and **44** in THF/H_2O 1:1

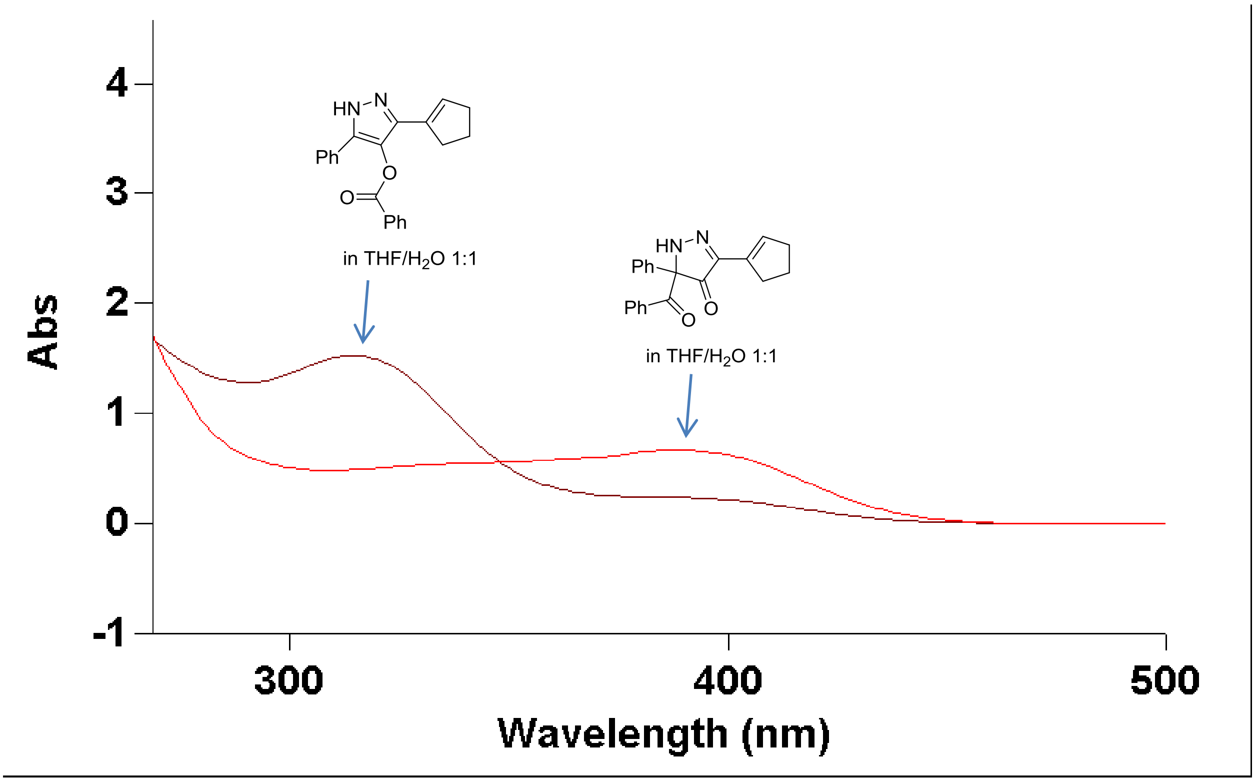

Kinetics of the rearrangement **1a** → **44** in THF/H_2O 1:1 at 400 nm – Monitoring the disappearance of **1a**:

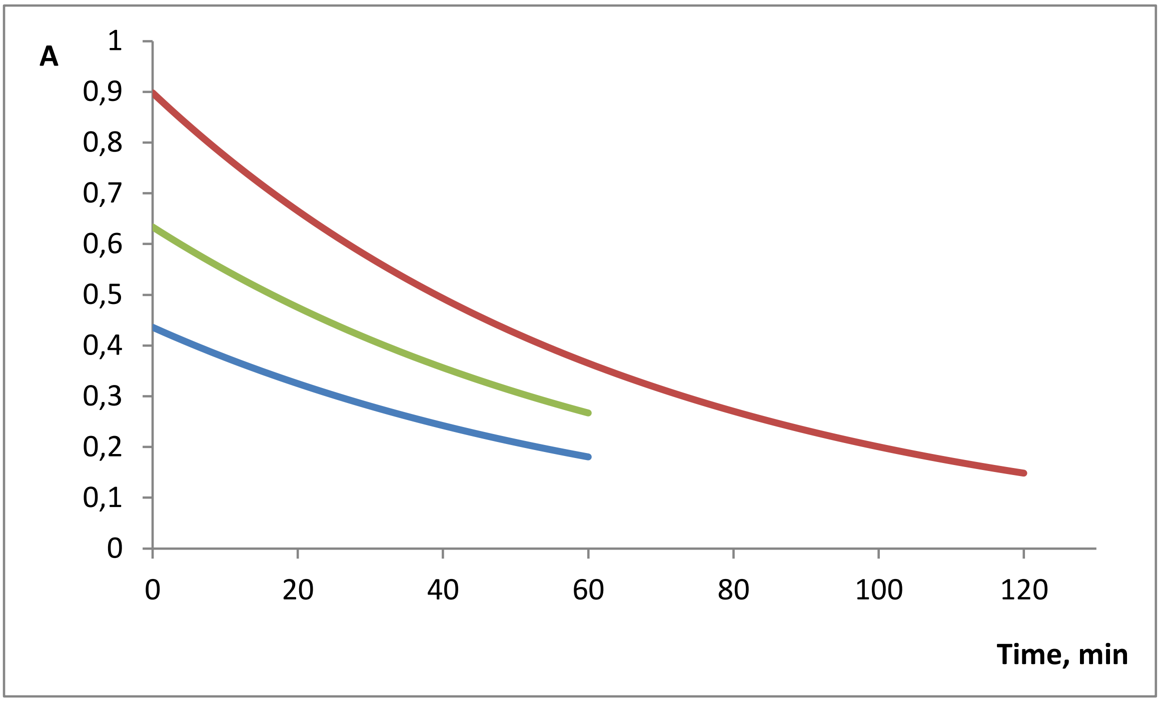

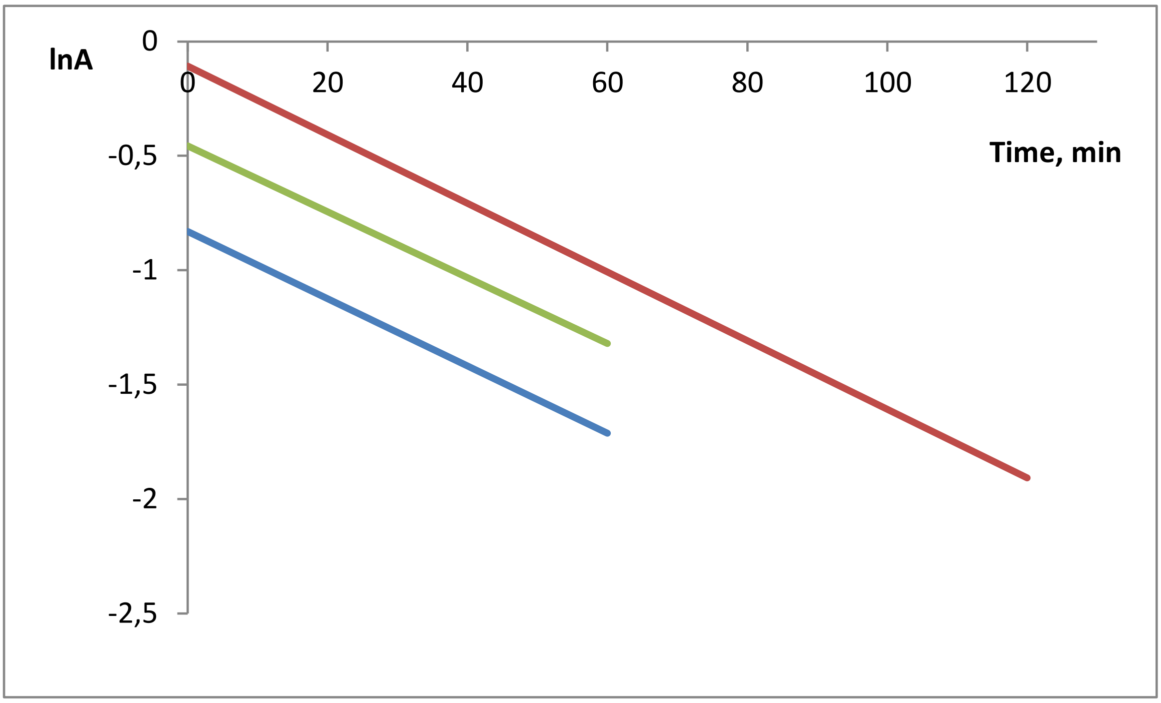

First order reaction rate constants determined at different concentrations of BCPP:

k = 2.40·10^{-4}; 2.45·10^{-4}; 2.49·10^{-4} sec^{-1}

k = 2.45·10^{-4}±0.05·10^{-4} sec^{-1}

Crystal Structure Report for 44a (cd1436a)

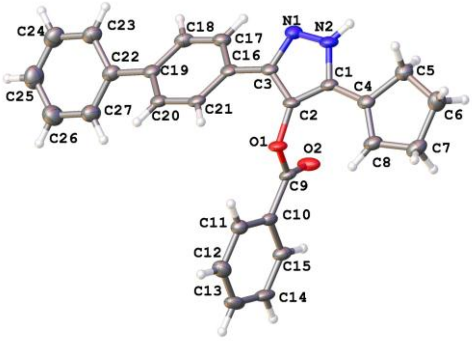

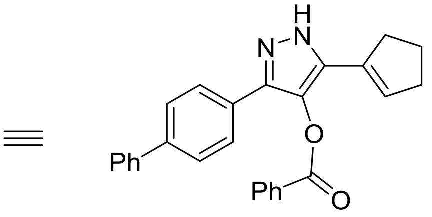

44a

Single crystals of **44a** ($C_{27}H_{22}N_2O_2$) were prepared by slow evaporation of a 1:1 hexane:DCM mixture. A suitable colorless plate-like crystal, with dimensions of 0.33 mm × 0.13 mm × 0.10 mm, was mounted, using Paratone oil, onto a nylon loop. The data were collected at 98(2) K using a Rigaku AFC12 / Saturn 724 CCD fitted with MoKα radiation (λ = 0.71075 Å). Data collection and unit cell refinement were performed using *CrystalClear* software.[10] The total number of data were measured in the range 6.46° < 2θ < 50.1° using ω scans. Data processing and absorption correction, giving minimum and maximum transmission factors (0.546, 1.000), were accomplished with *CrystalClear*[10] and *ABSCOR*[11], respectively. The structure, using

Olex2[12], was solved with the ShelXT[13] structure solution program using direct methods and refined (on F^2) with the ShelXL[14] refinement package using full-matrix, least-squares techniques. All non-hydrogen atoms were refined with anisotropic displacement parameters. Electron density peaks were used to determine the hydrogen atoms bound to N2, C5, C6 and C7 atoms. All other hydrogen atom positions were determined by geometry and refined by a riding model.

Table S1. Crystallographic Data and Structure Refinement for cd1436a

Identification code	cd1332
Empirical formula	$C_{27}H_{22}N_2O_2$
Formula weight	406.48
Crystal system	Triclinic
Space group	*P -1*
a (Å)	5.7431(13)
b (Å)	12.655(3)
c (Å)	15.118(4)
α (°)	69.385(6)
β (°)	82.282(6)
γ (°)	87.431(7)
Volume (Å^3)	1019.1(4)
Z	2
ρ (calc.)	1.325
λ	0.71075
Temp. (K)	98(2)
F(000)	428
μ (mm^{-1})	0.084
T_{min}, T_{max}	0.546, 1.000
$2θ_{range}$ (°)	6.46 to 50.1

Reflections collected	6632
Independent reflections	3572<br>[R(int) = 0.0627]
Completeness	99.5%
Data / restraints / parameters	3572 / 0 / 301
Observed data<br>[I > 2σ(I)]	2697
wR(F^2 all data)	0.1385
R(F obsd data)	0.0610
Goodness-of-fit on F^2	1.00
largest diff. peak and hole (e Å^{-3})	0.30 / -0.34

$$wR_2 = \{ \Sigma [w(F_o^2 - F_c^2)^2] / \Sigma [w(F_o^2)^2] \}^{1/2}$$

$$R_1 = \Sigma ||F_o| - |F_c|| / \Sigma |F_o|$$

References

1. Marichev, K. O.; Qiu, H.; Offield, A. C.; Arman, H.; Doyle, M. P. *J. Org. Chem.* **2016**, *81*, 9235.
2. Yokomatsu, T.; Arakawa, A.; Shibuya, S. *J. Org. Chem.* **1994**, *59*, 3506.
3. Kuwano, R.; Kameyama, N.; Ikeda, R. *J. Am. Chem. Soc.* **2011**, *133*, 7312.
4. Morcuende, A.; Ors, M.; Valverde, S.; Herradón, B. *J. Org. Chem.* **1996**, *61*, 5264.
5. Petersson, M. J.; Jenkins, I. D.; Loughlin, W. A. *Org. Biomol. Chem.* **2009**, *7*, 739.
6. Lao, J.-H.; Zhang, X.-J.; Wang, J.-J.; Li, X.-M.; Yan, M.; Luo, H.-B. *Tetrahedron: Asymmetry* **2009**, *20*, 2818.
7. Zhang, Z.; Yin, Z.; Meanwell, N. A.; Kadow, J. F.; Wang, T. *Org. Lett.* **2003**, *5*, 3399.
8. Wang, T.; Zhang, Z.; Meanwell, N. A. *Tetrahedron Lett.* **1999**, *40*, 6745.
9. Murahashi, S.-I.; Naota, T.; Nakajima, N. *Chem. Lett.* **1987**, *5*, 879.
10. *CrystalClear User's Manual*, Rigaku/MSC Inc., Rigaku Corporation, the Woodlands, TX, **2011**.
11. Higashi, T. *ABSCOR*, Rigaku Corporation, Tokyo, Japan, **1995**.
12. Dolomanov, O. V.; Bourhis, L. J.; Gildea, R. J.; Howard, J. A. K.; Puschmann, H. *J. Appl. Cryst.* **2009**, *42*, 339.
13. Sheldrick, G. M. *Acta Cryst.* **2015**, *A71*, 3.
14. Sheldrick, G. M. *Acta Cryst.* **2008**, *A64*, 112.

^{1}H and ^{13}C NMR Spectra of Products

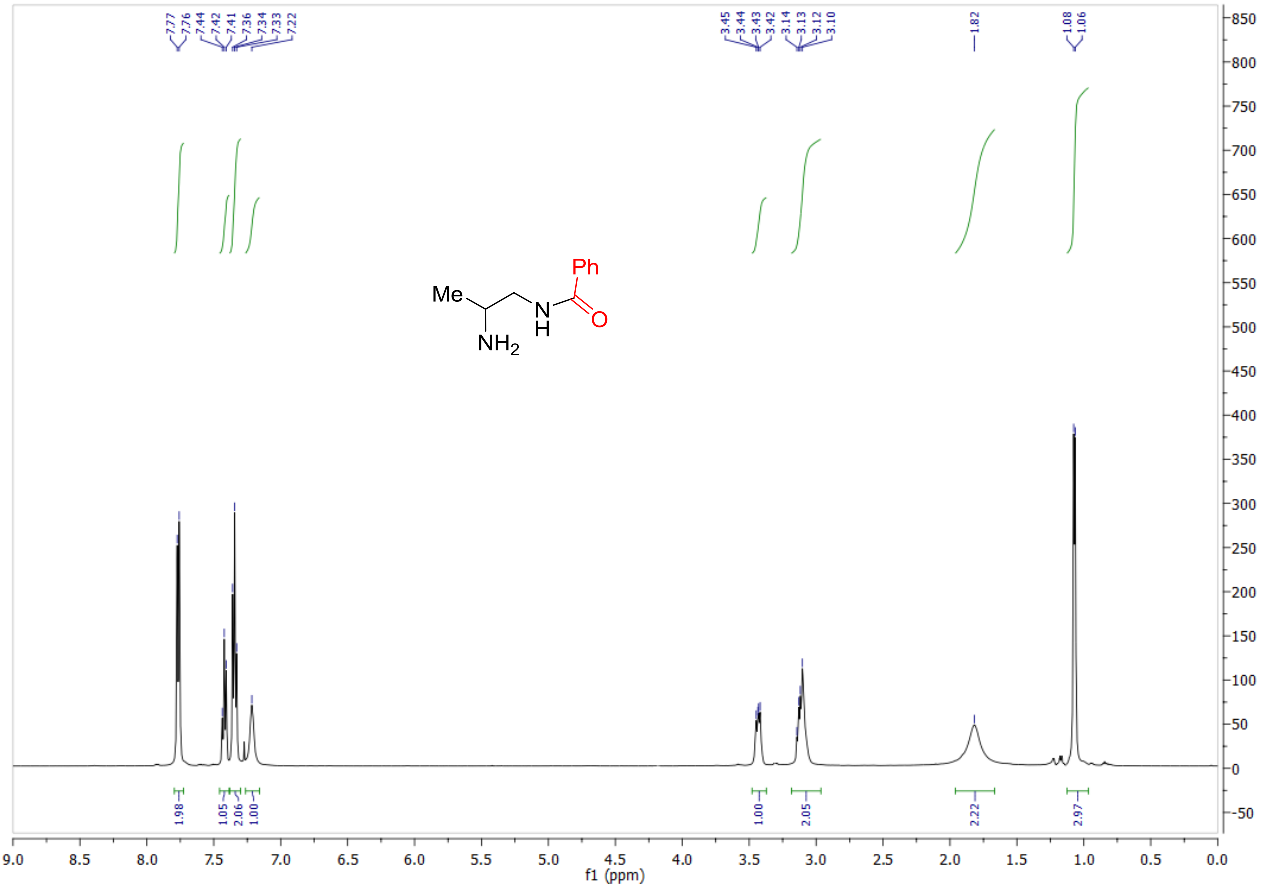

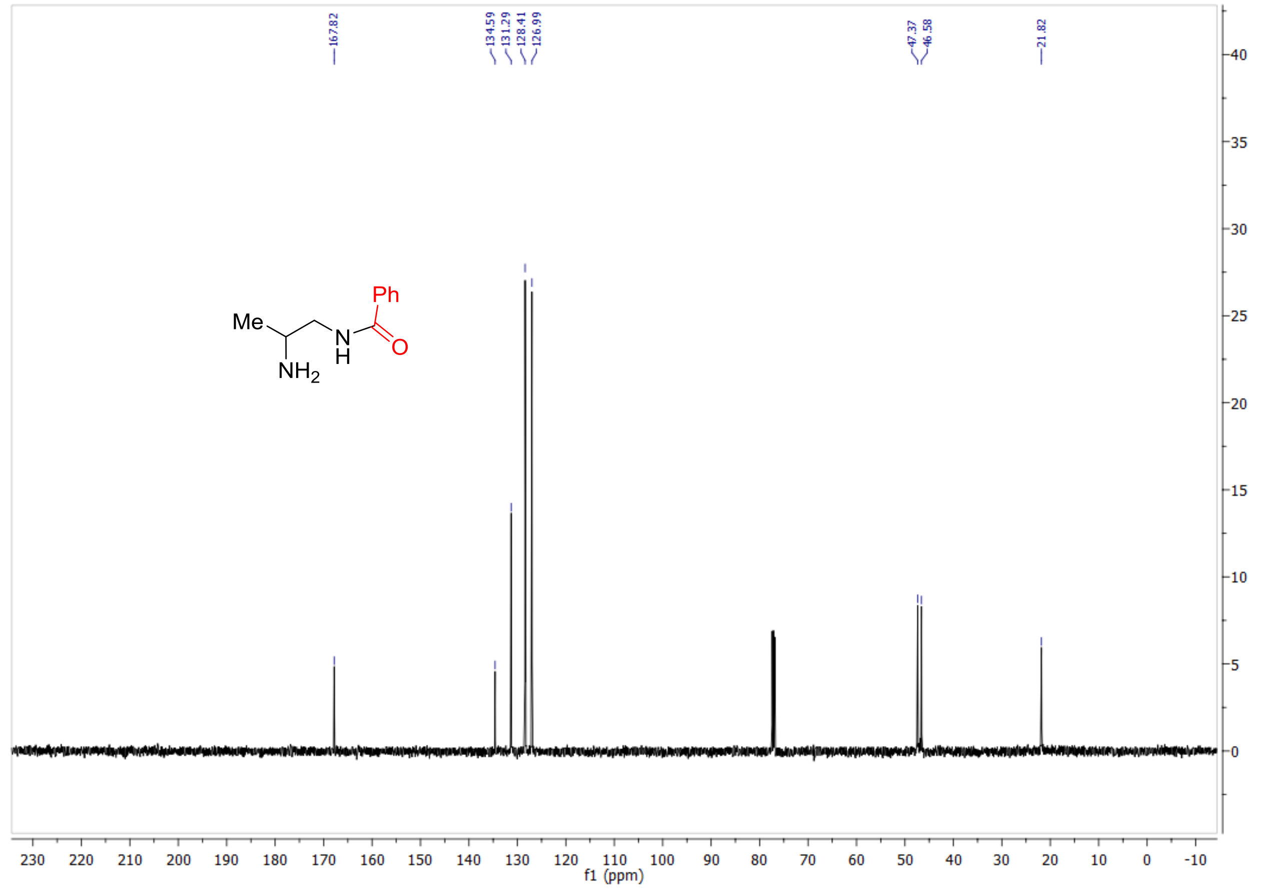

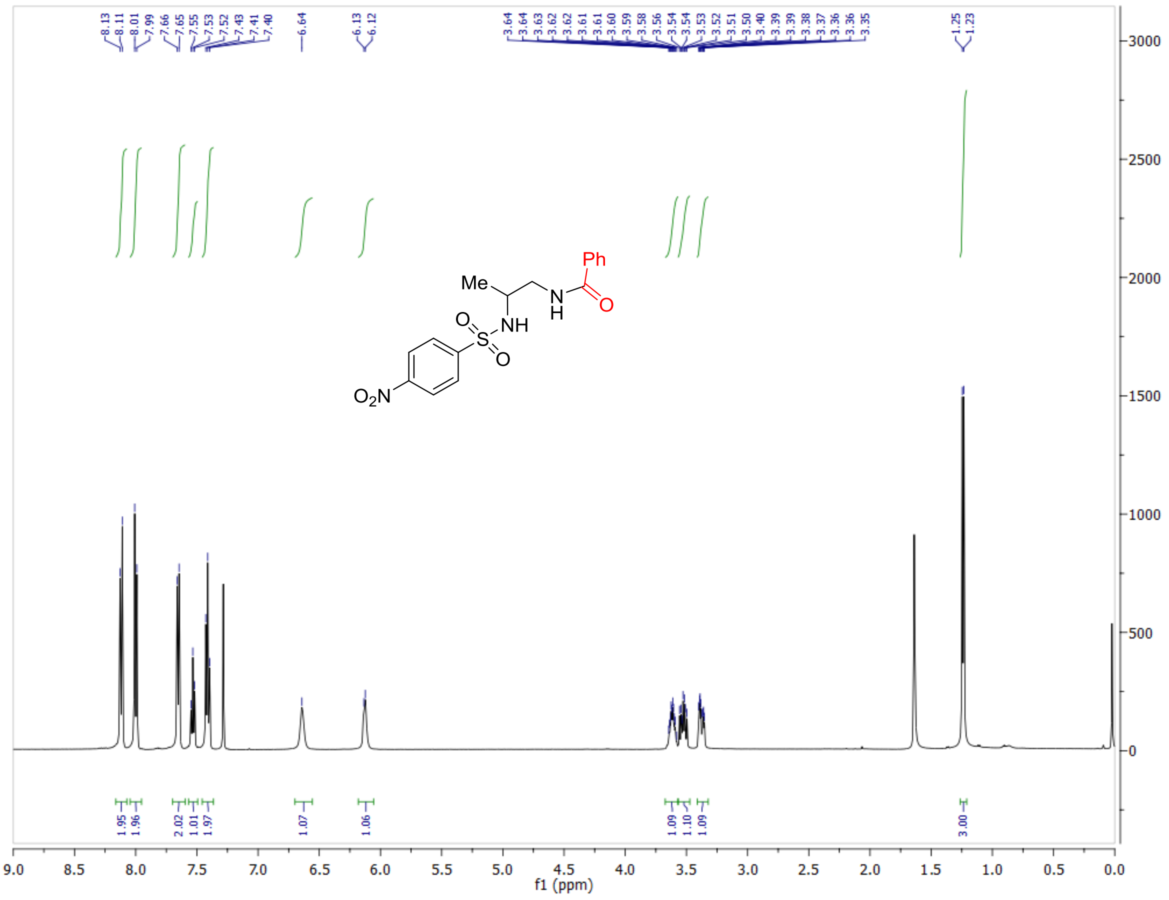
8.13
8.11
8.01
7.99
7.66
7.65
7.55
7.53
7.52
7.43
7.41
7.40
6.64
6.13
6.12
1.25
1.23
Me
Ph
N
H
O
NH
S
O
O
O_2N
1.95
1.96
2.02
1.01
1.97
1.07
1.06
1.09
1.10
1.09
3.00
f1 (ppm)

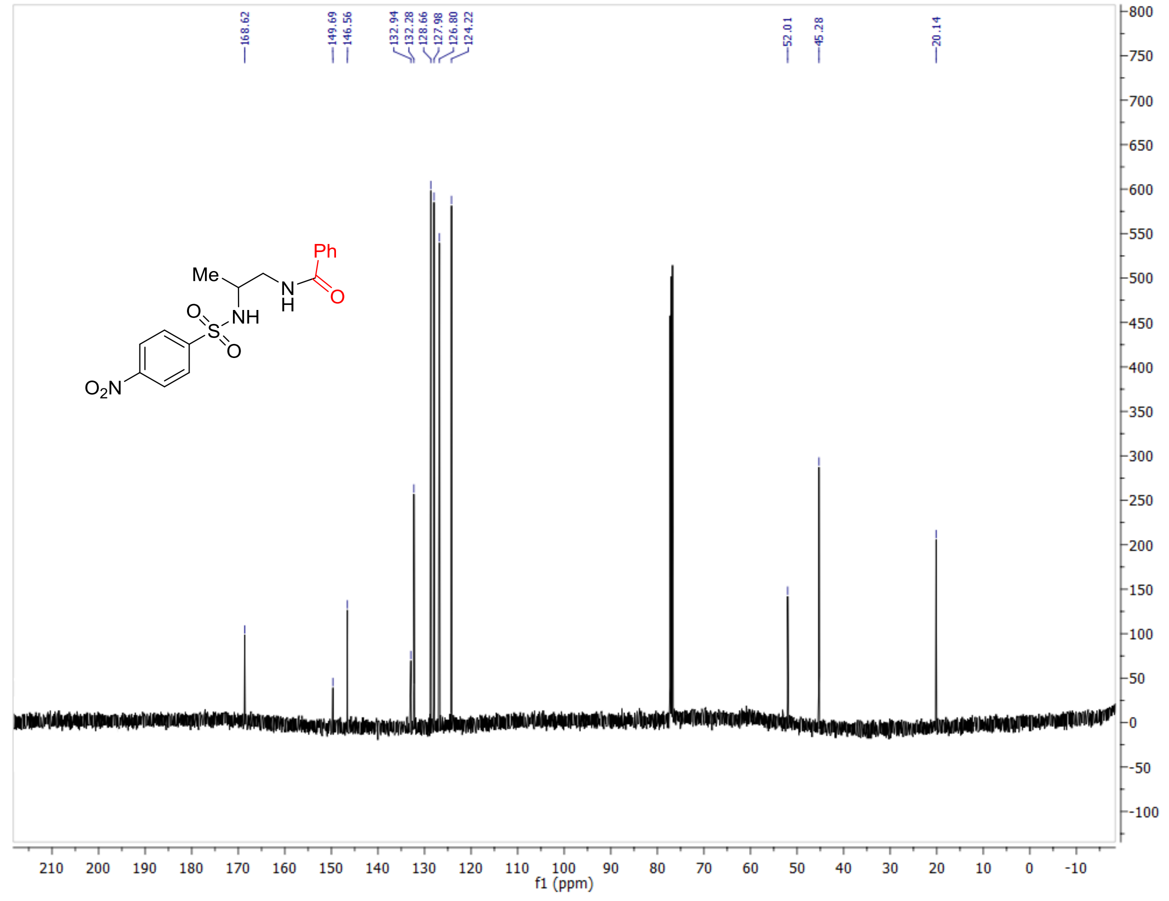
168.62
149.69
146.56
132.94
132.28
128.66
127.98
126.80
124.22
52.01
45.28
20.14
Me
Ph
N
H
O
NH
S
O
O
O_2N
f1 (ppm)

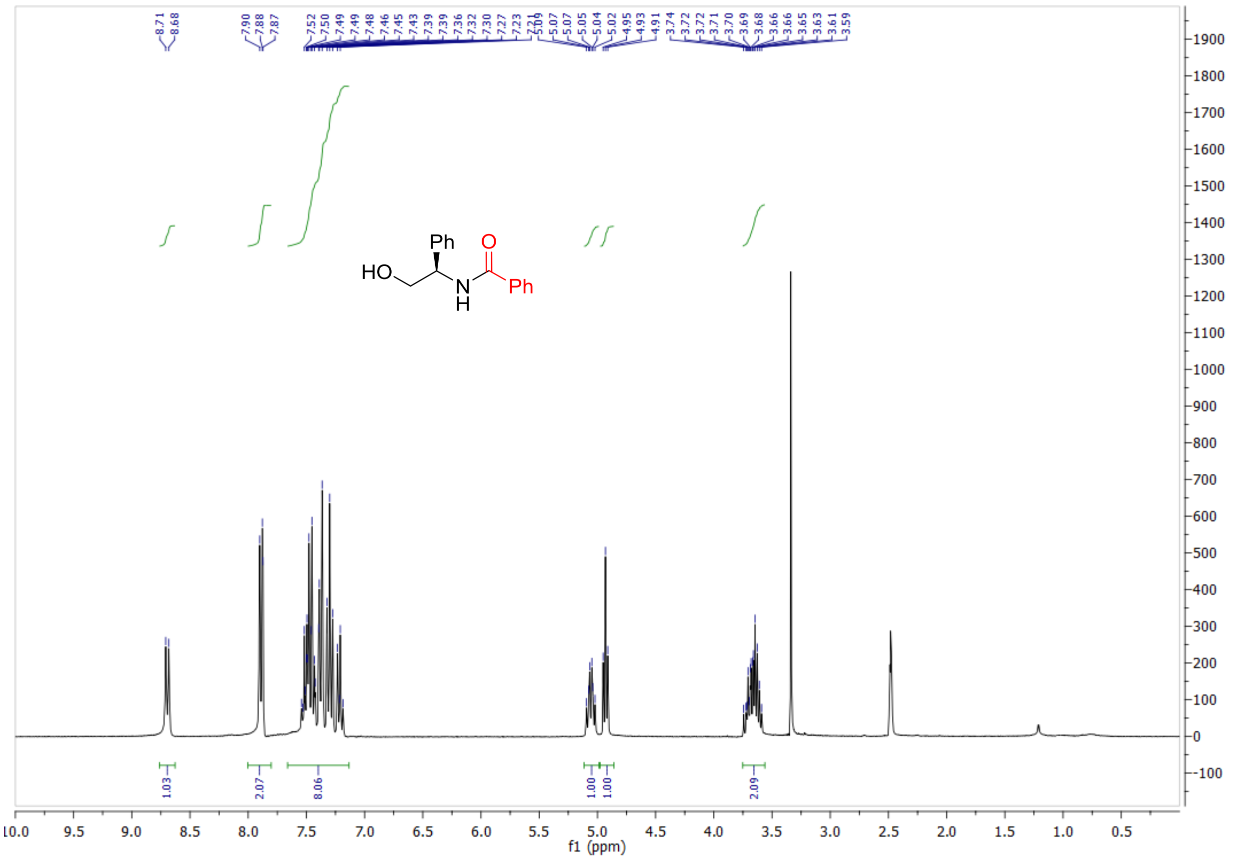

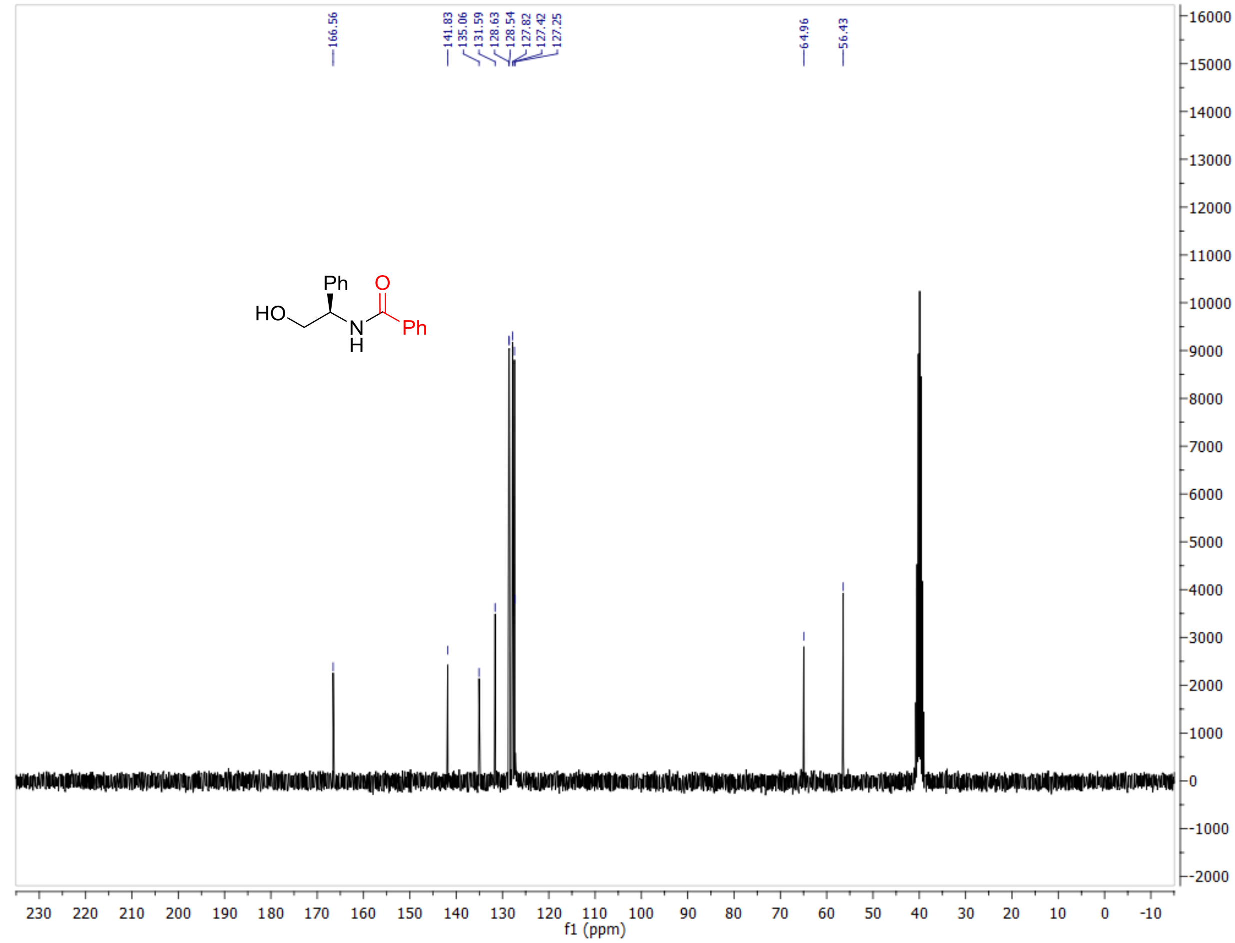

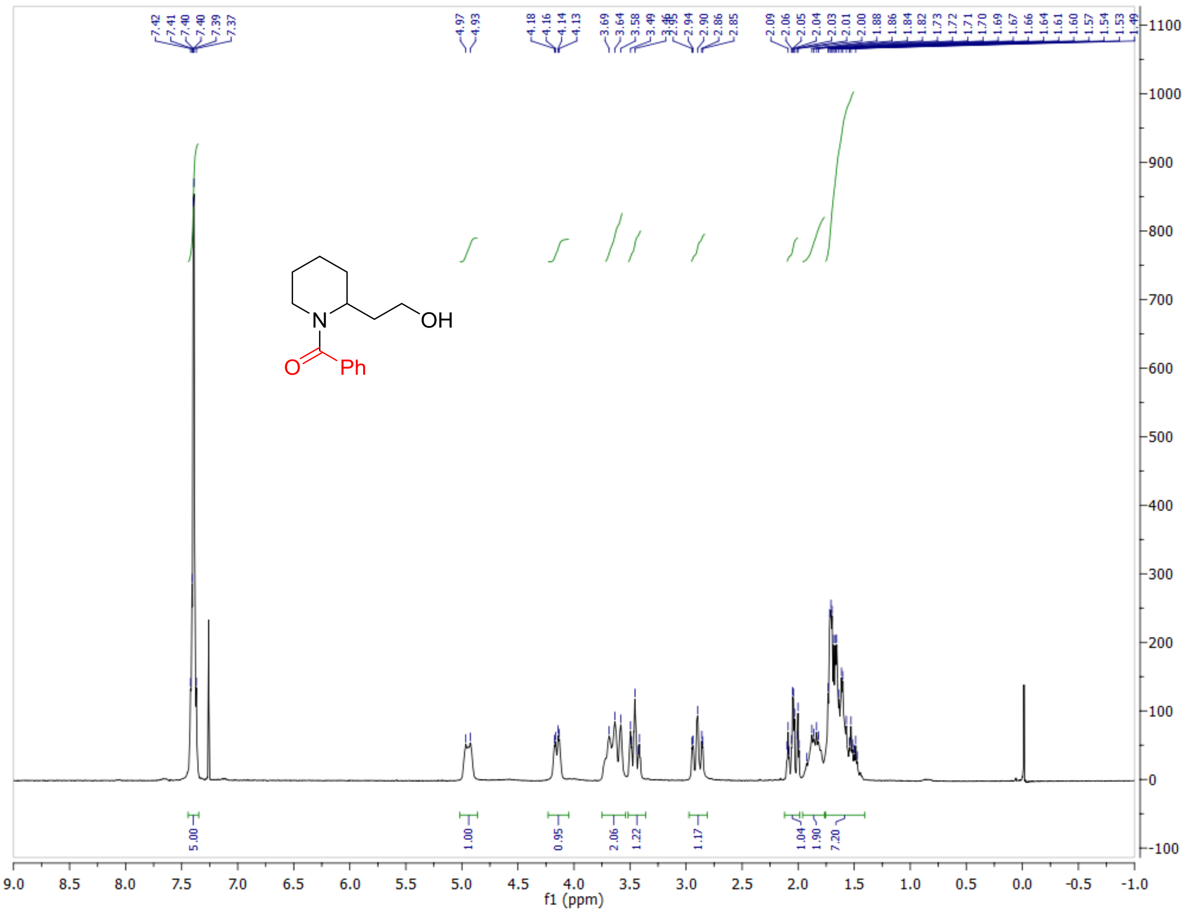

7.42
7.41
7.40
7.40
7.39
7.37
4.97
4.93
4.18
4.16
4.14
4.13
3.69
3.64
3.58
3.49
2.94
2.90
2.86
2.85
2.09
2.06
2.05
2.04
2.03
2.01
2.00
1.88
1.86
1.84
1.82
1.73
1.72
1.71
1.70
1.69
1.67
1.66
1.64
1.61
1.60
1.57
1.54
1.53
N
OH
O
Ph
5.00
1.00
0.95
2.06
1.22
1.17
1.04
1.90
7.20
f1 (ppm)

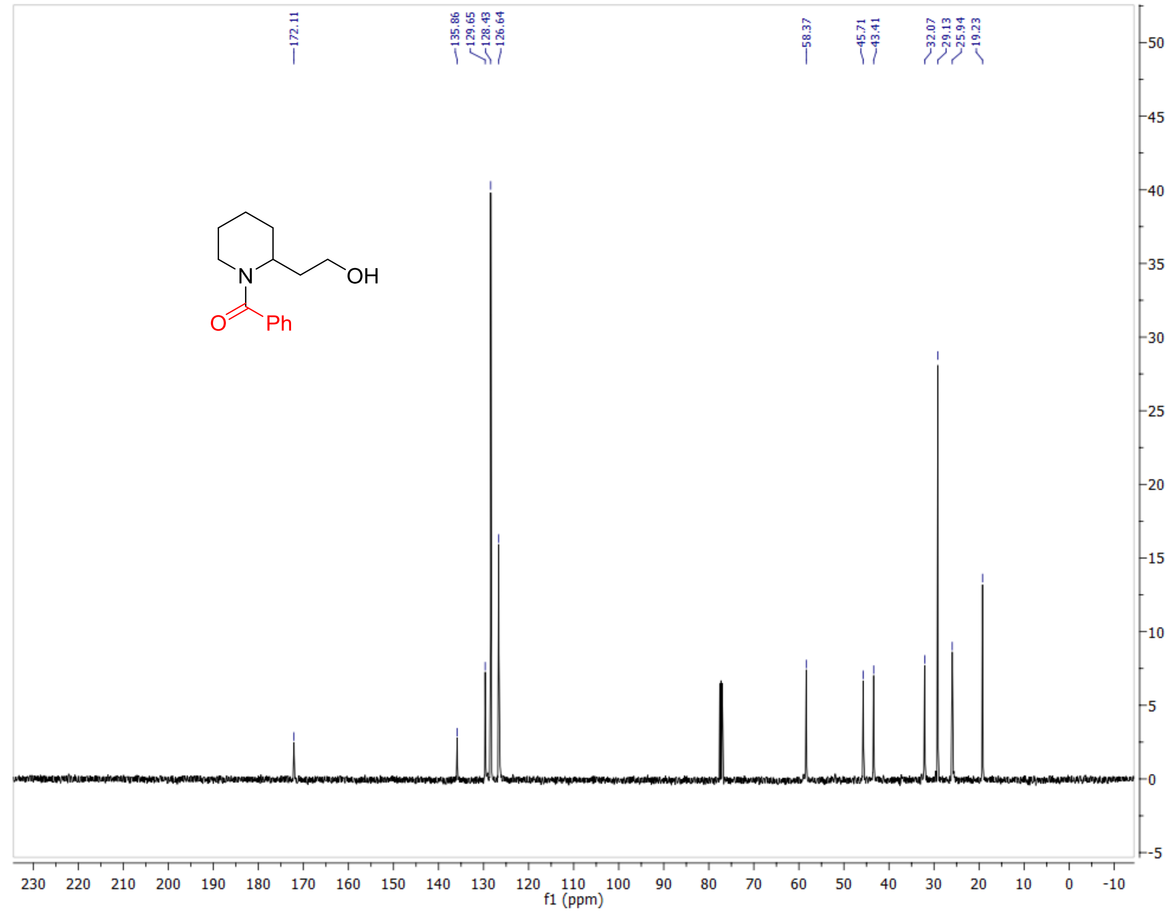

172.11
135.86
129.65
128.43
126.64
58.37
45.71
43.41
32.07
29.13
25.94
19.23
N
OH
O
Ph
f1 (ppm)

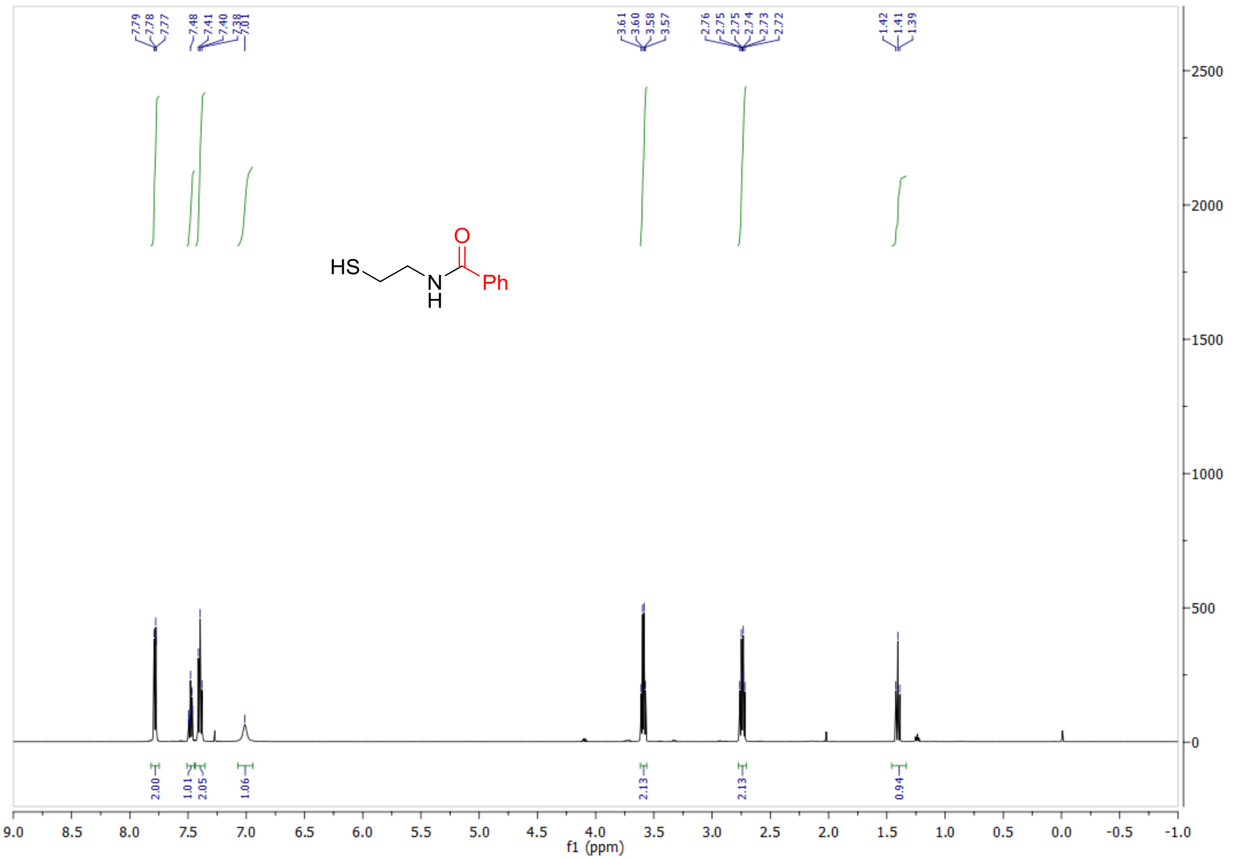
7.79
7.78
7.77
7.48
7.41
7.40
7.38
7.01
3.61
3.60
3.58
3.57
2.76
2.75
2.75
2.74
2.73
2.72
1.42
1.41
1.39
HS
N
H
O
Ph
2.00
1.01
2.05
1.06
2.13
2.13
0.94
f1 (ppm)

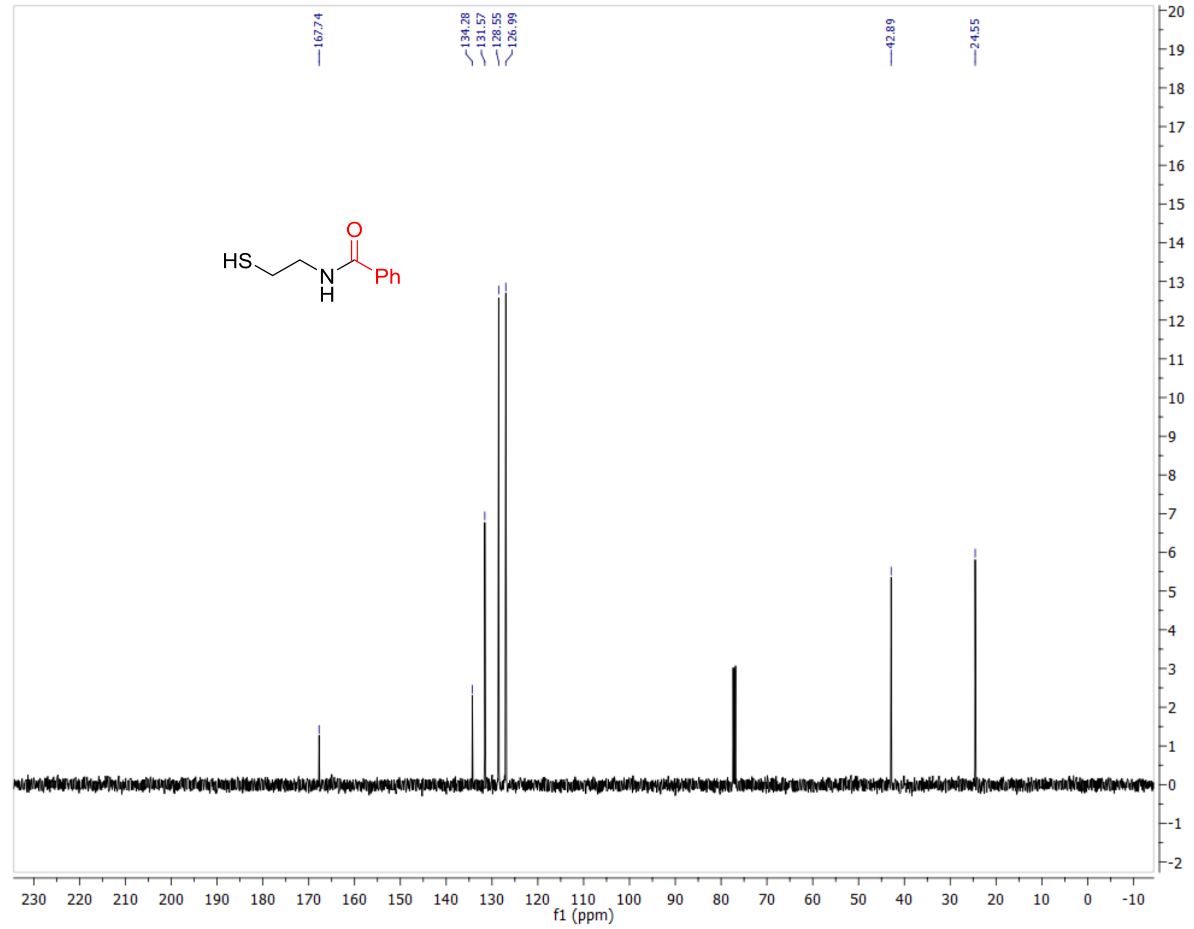
167.74
134.28
131.57
128.55
126.99
42.89
24.55
HS
N
H
O
Ph
f1 (ppm)

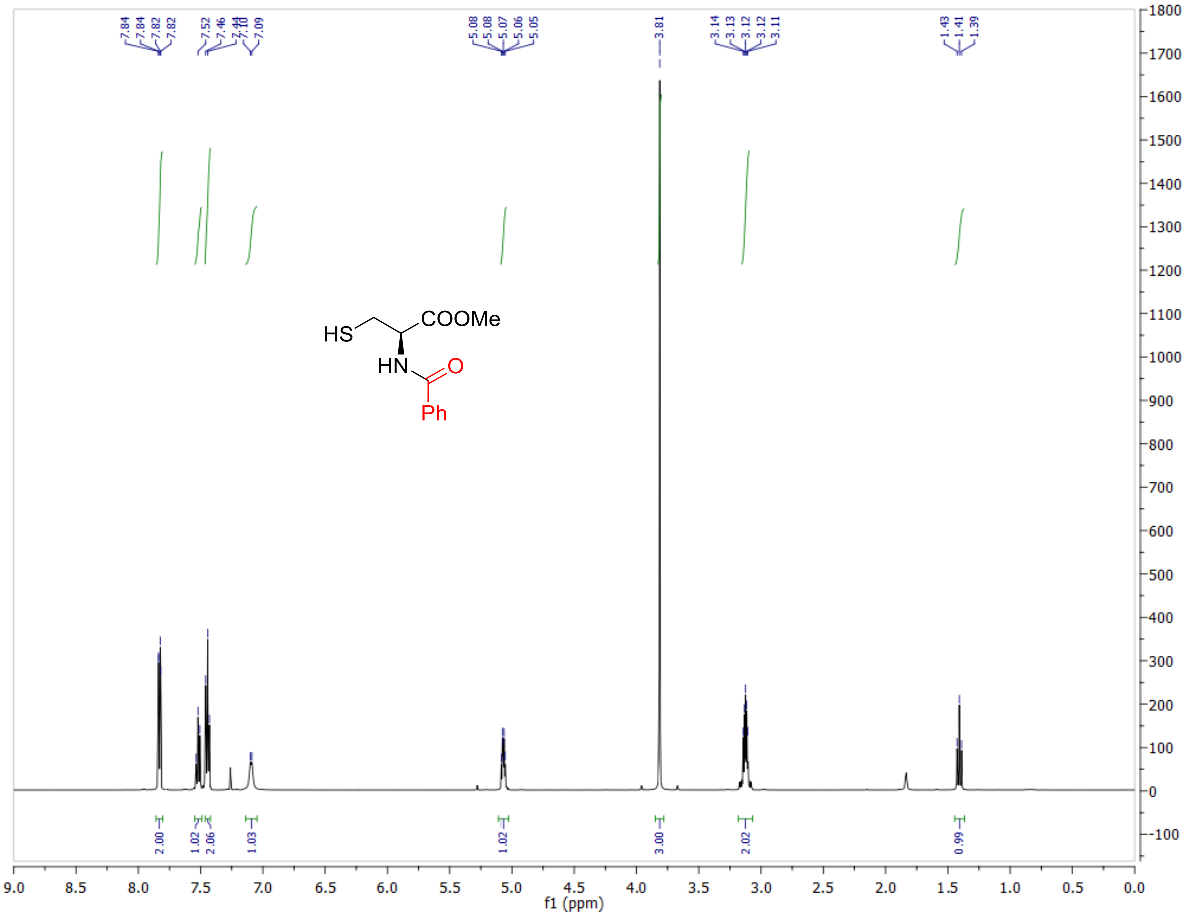

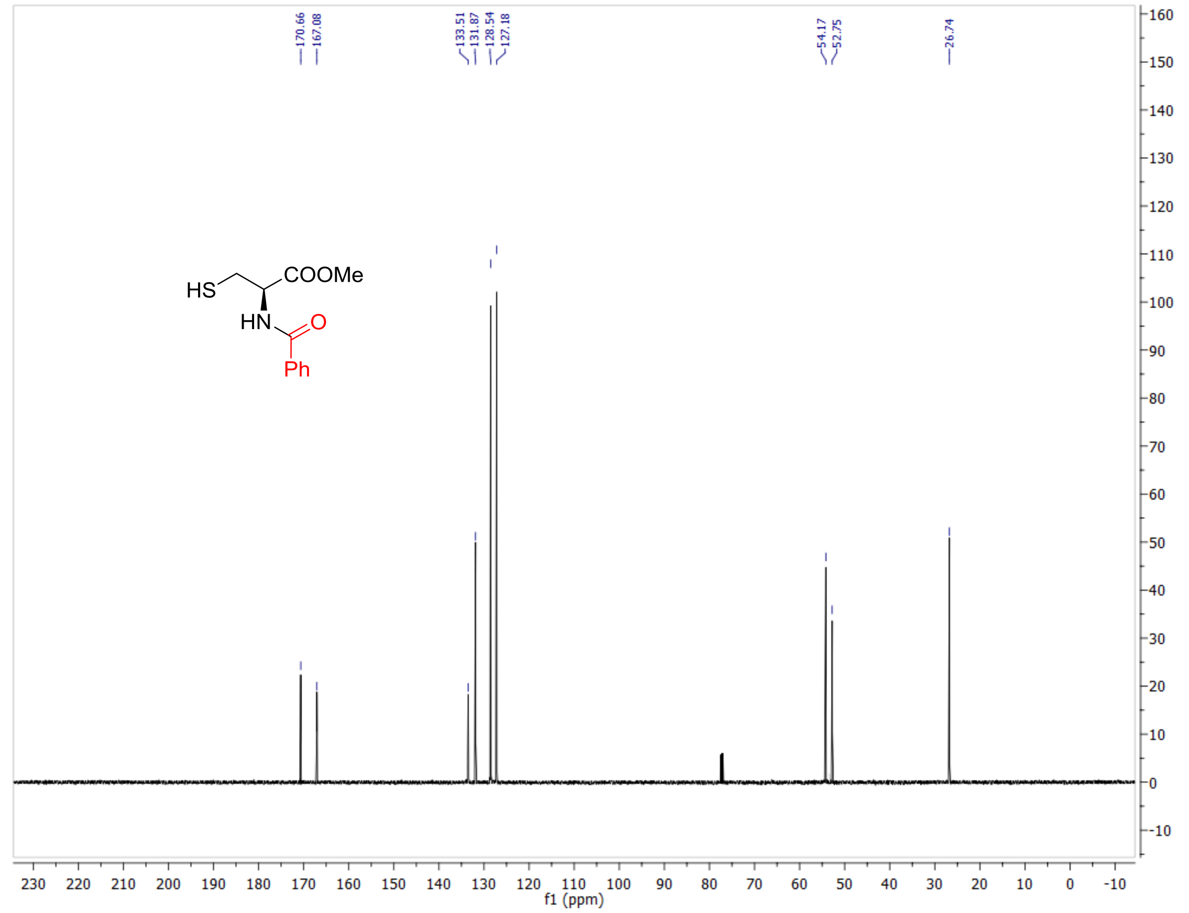

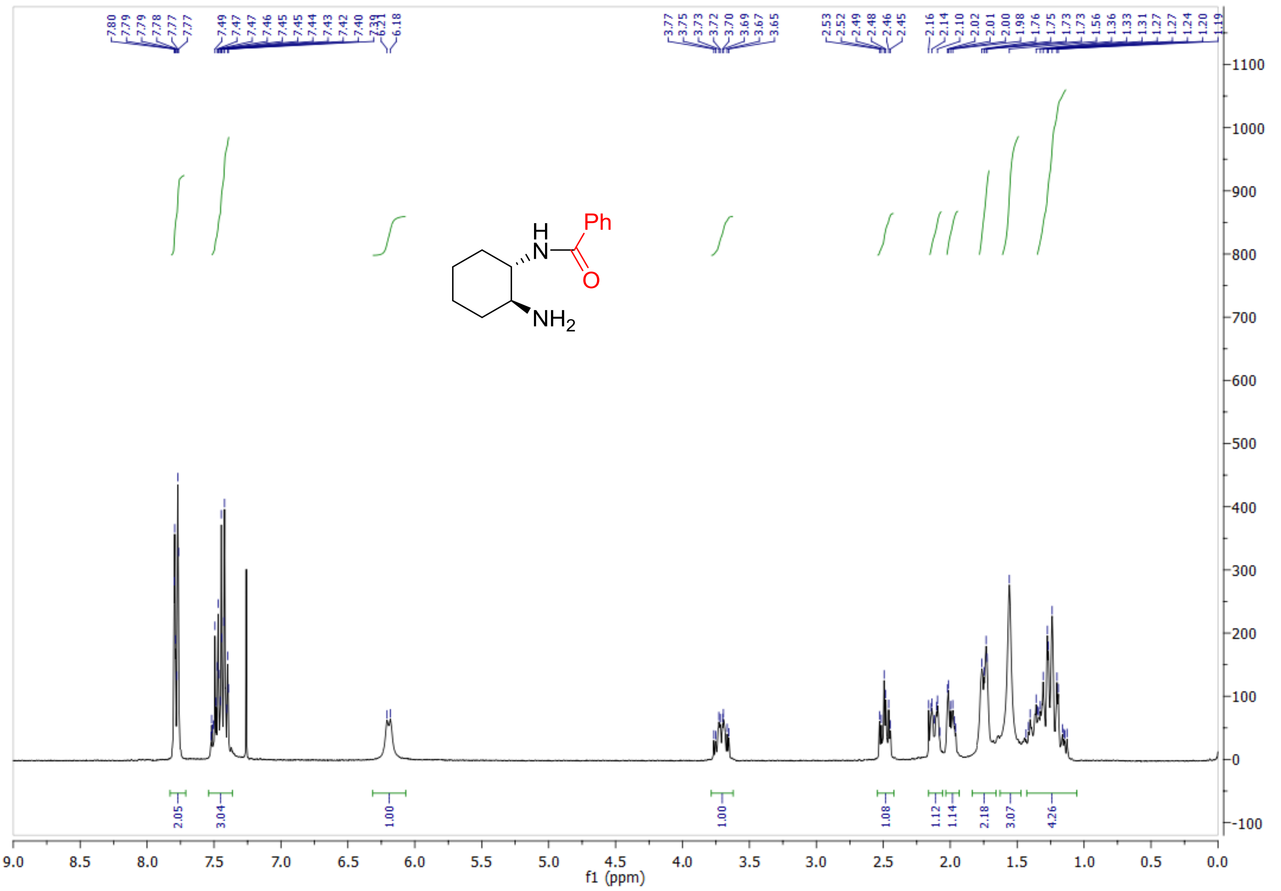
7.80 7.79 7.79 7.78 7.77 7.77
7.49 7.47 7.47 7.46 7.45 7.45 7.44 7.43 7.42 7.40
6.21 6.18
3.77 3.75 3.73 3.72 3.70 3.69 3.67 3.65
2.53 2.52 2.49 2.48 2.46 2.45
2.16 2.14 2.10 2.02 2.01 2.00 1.98 1.76 1.75 1.73 1.73 1.56 1.36 1.33 1.31 1.27 1.27 1.24 1.20 1.19
Ph
H
N
O
NH2
2.05
3.04
1.00
1.00
1.08
1.12
1.14
2.18
3.07
4.26
f1 (ppm)

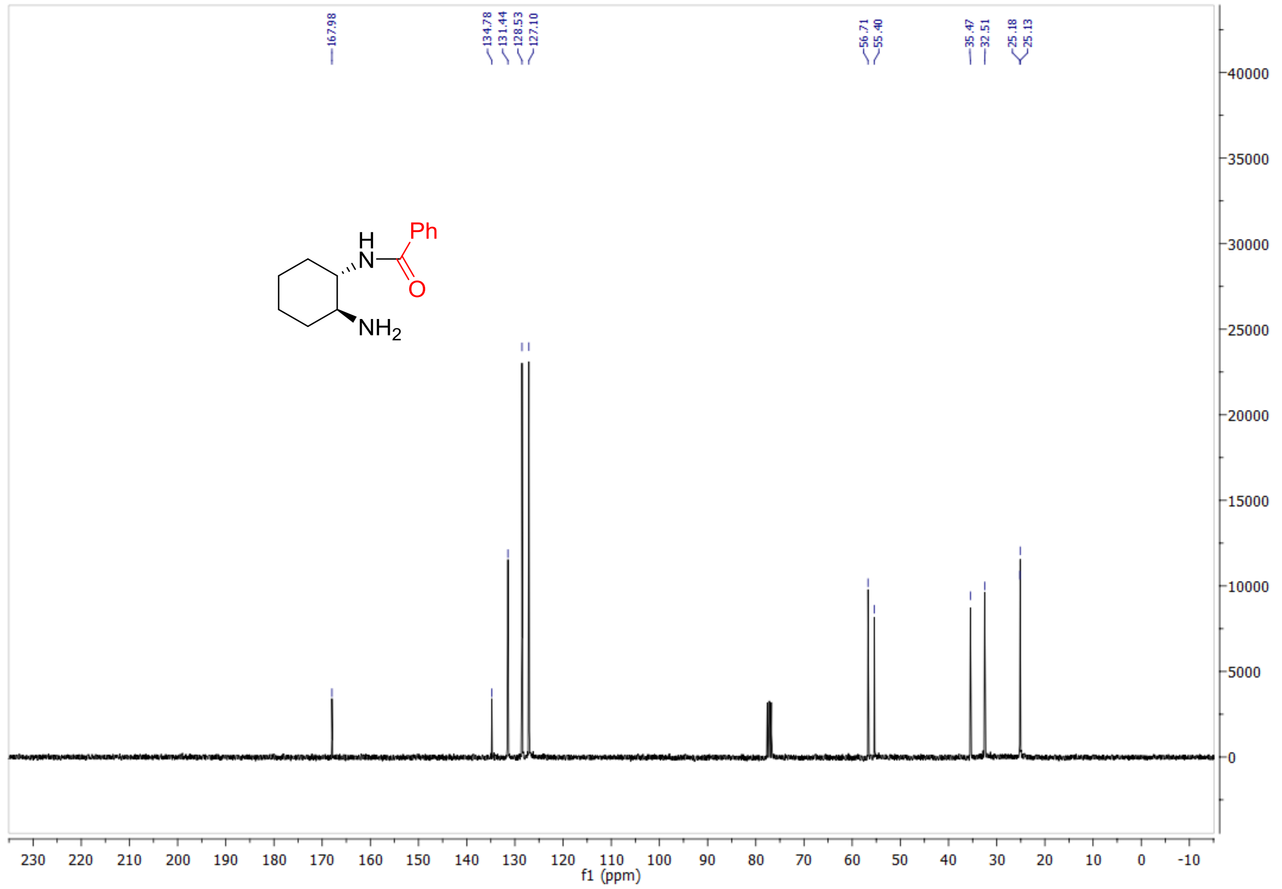
167.98
134.78 131.44 128.53 127.10
56.71 55.40
35.47 32.51 25.18 25.13
Ph
H
N
O
NH2
f1 (ppm)

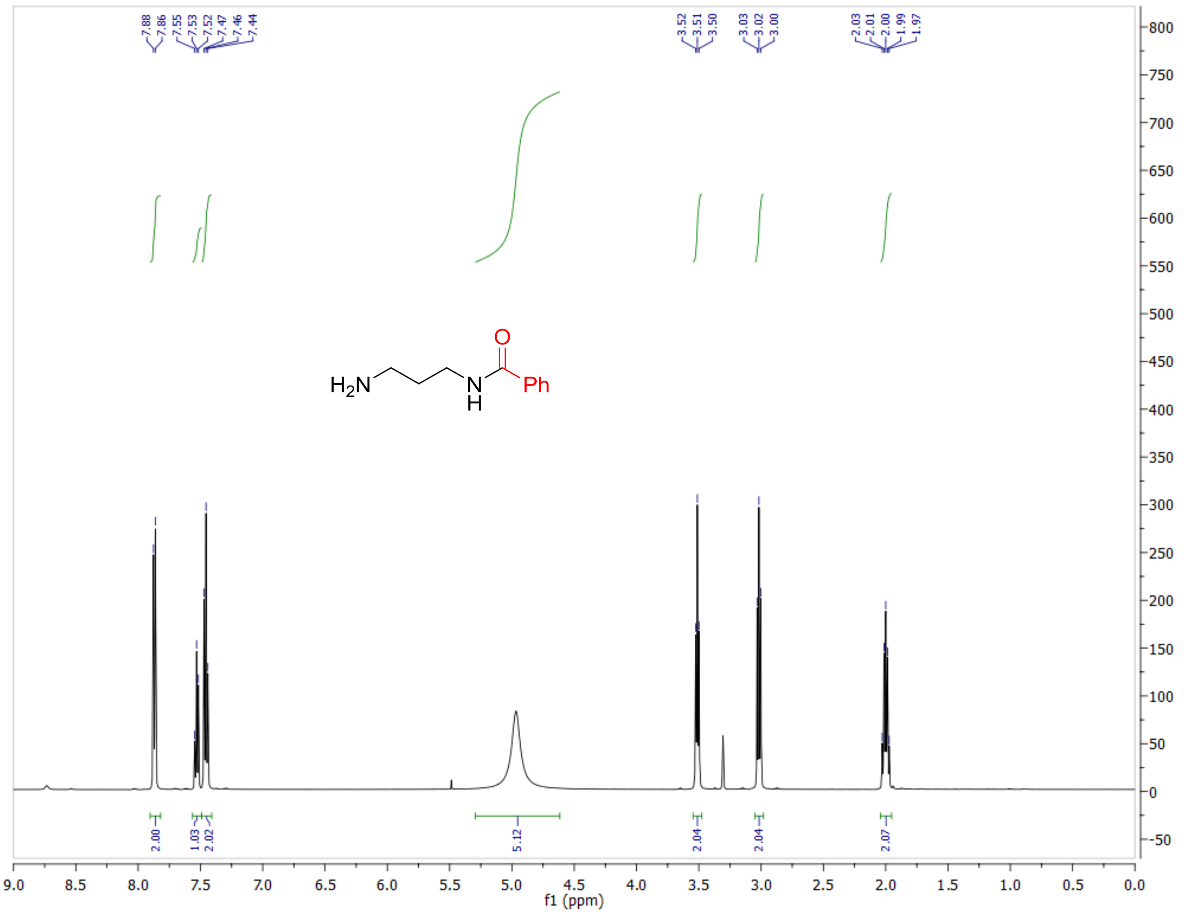
7.88
7.86
7.55
7.53
7.52
7.47
7.46
7.44
3.52
3.51
3.50
3.03
3.02
3.00
2.03
2.01
2.00
1.99
1.97
H2N
N
H
O
Ph
2.00
1.03
2.02
5.12
2.04
2.04
2.07
f1 (ppm)

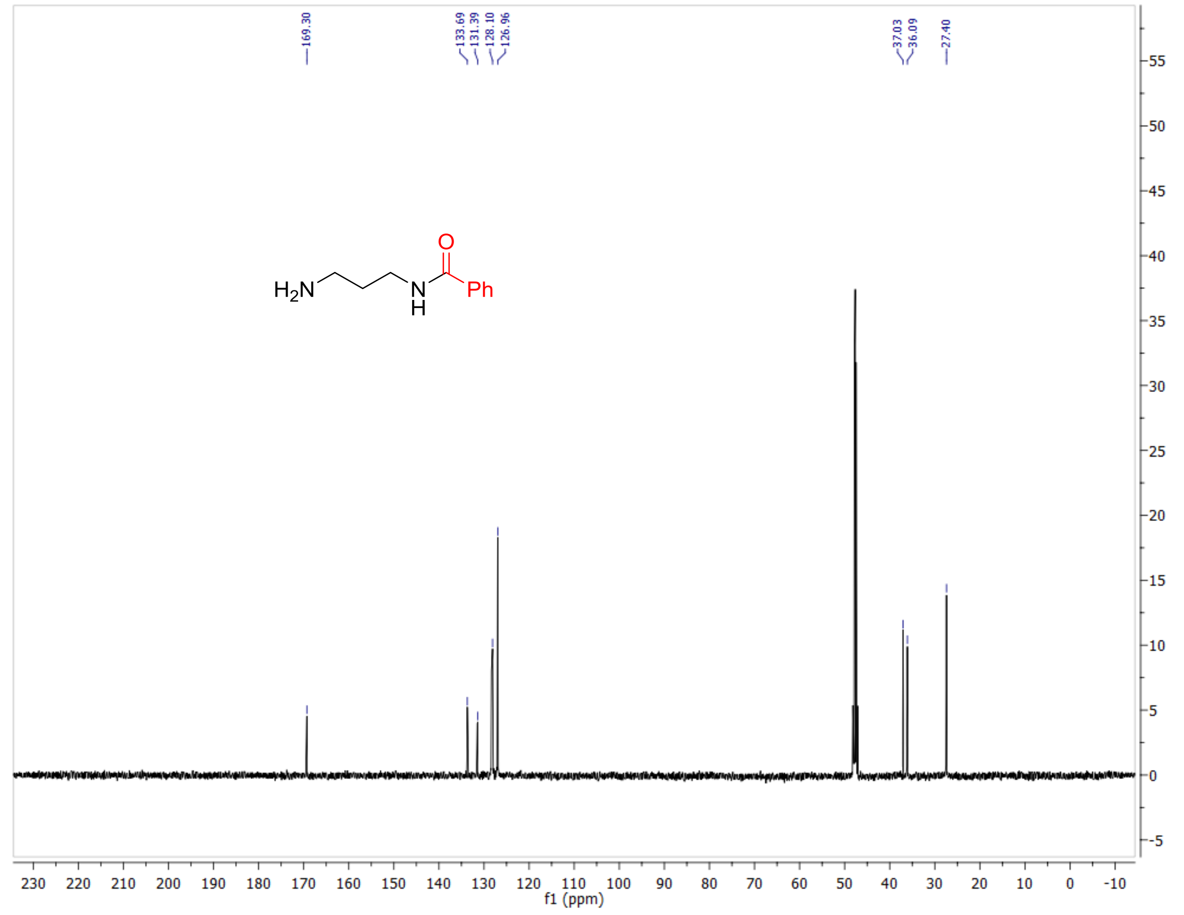
169.30
133.69
131.39
128.10
126.96
37.03
36.09
27.40
H2N
N
H
O
Ph
f1 (ppm)

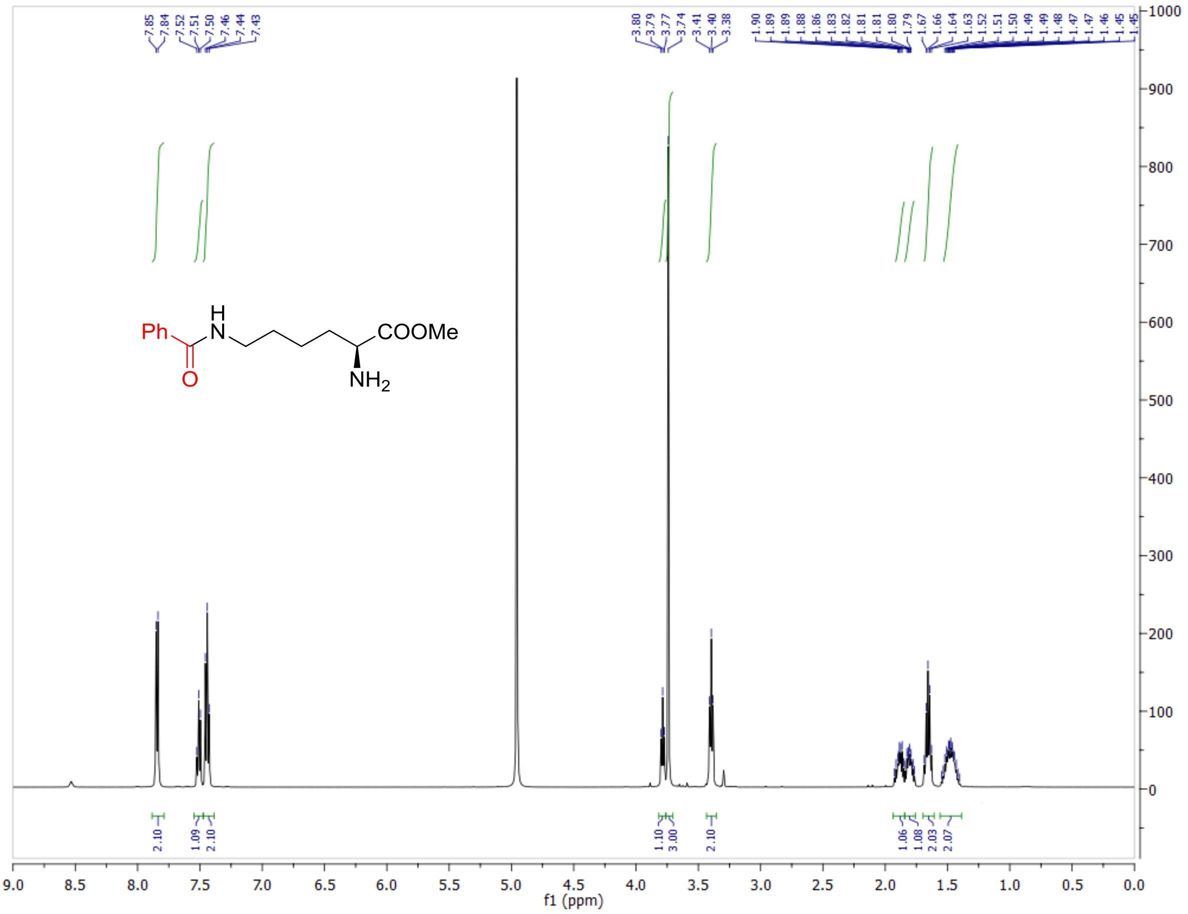

7.85
7.84
7.52
7.51
7.50
7.46
7.44
7.43
3.80
3.79
3.77
3.74
3.41
3.40
3.38
1.90
1.89
1.89
1.88
1.86
1.83
1.82
1.81
1.81
1.80
1.79
1.67
1.66
1.64
1.63
1.52
1.51
1.50
1.49
1.49
1.48
1.47
1.47
1.46
1.45
1.45
Ph
H
N
COOMe
O
NH2
2.10
1.09
2.10
1.10
3.00
2.10
1.06
1.08
2.03
2.07
f1 (ppm)

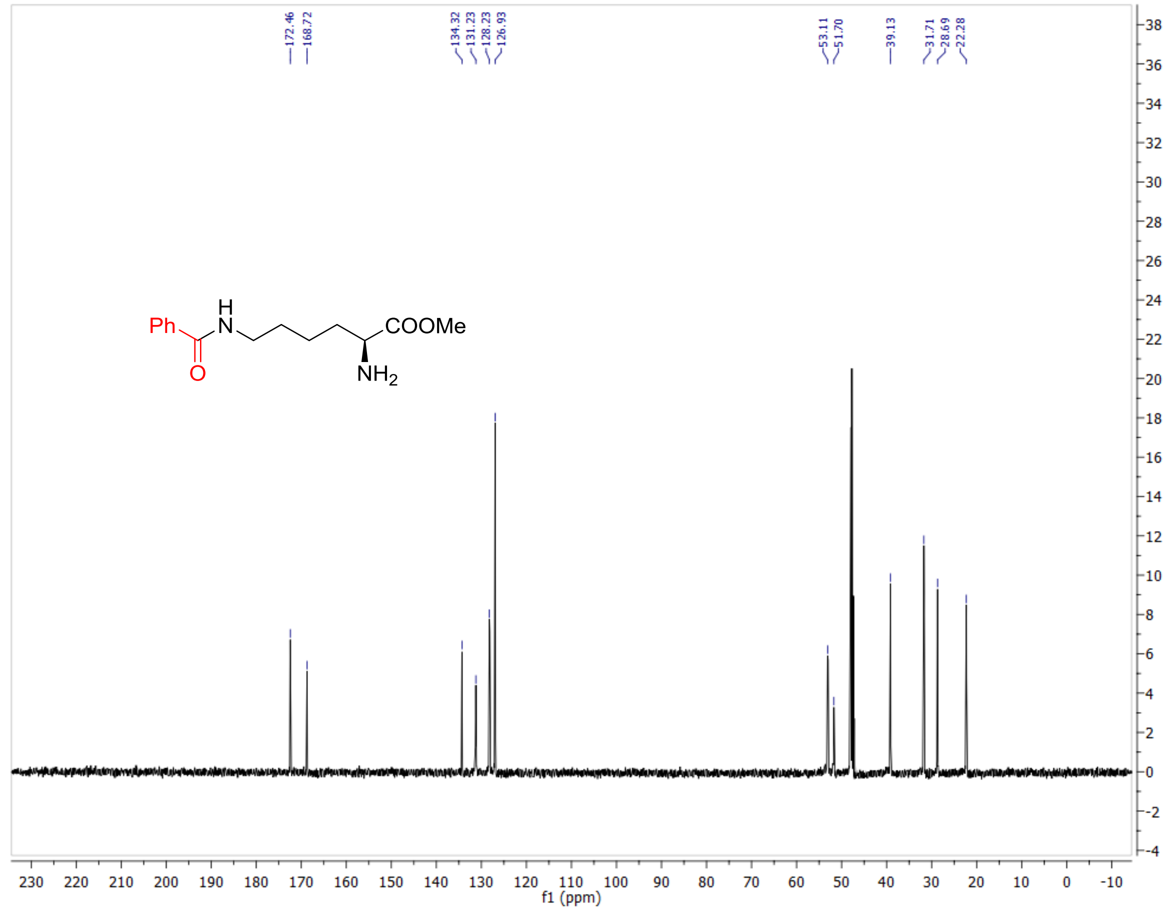

172.46
168.72
134.32
131.23
128.23
126.93
53.11
51.70
39.13
31.71
28.69
22.28
Ph
H
N
COOMe
O
NH2
f1 (ppm)

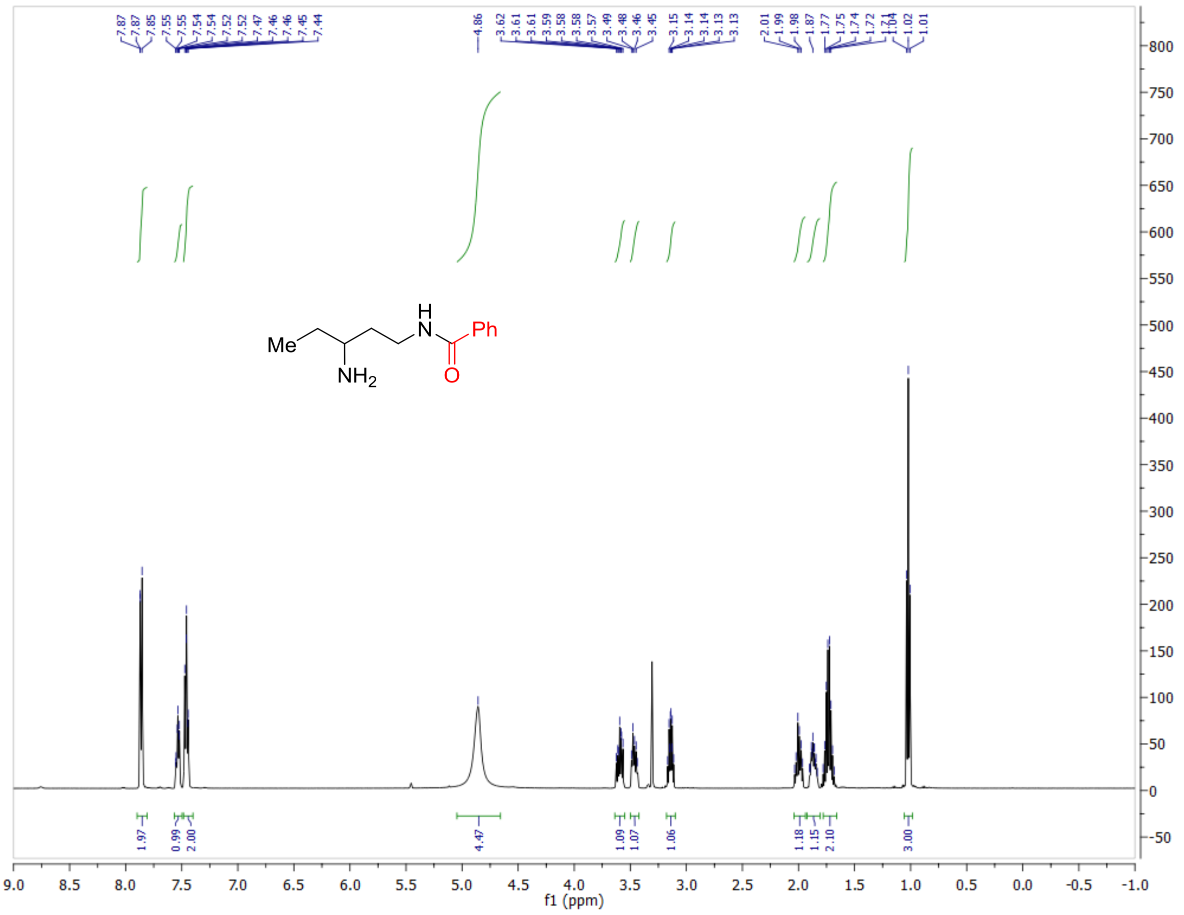

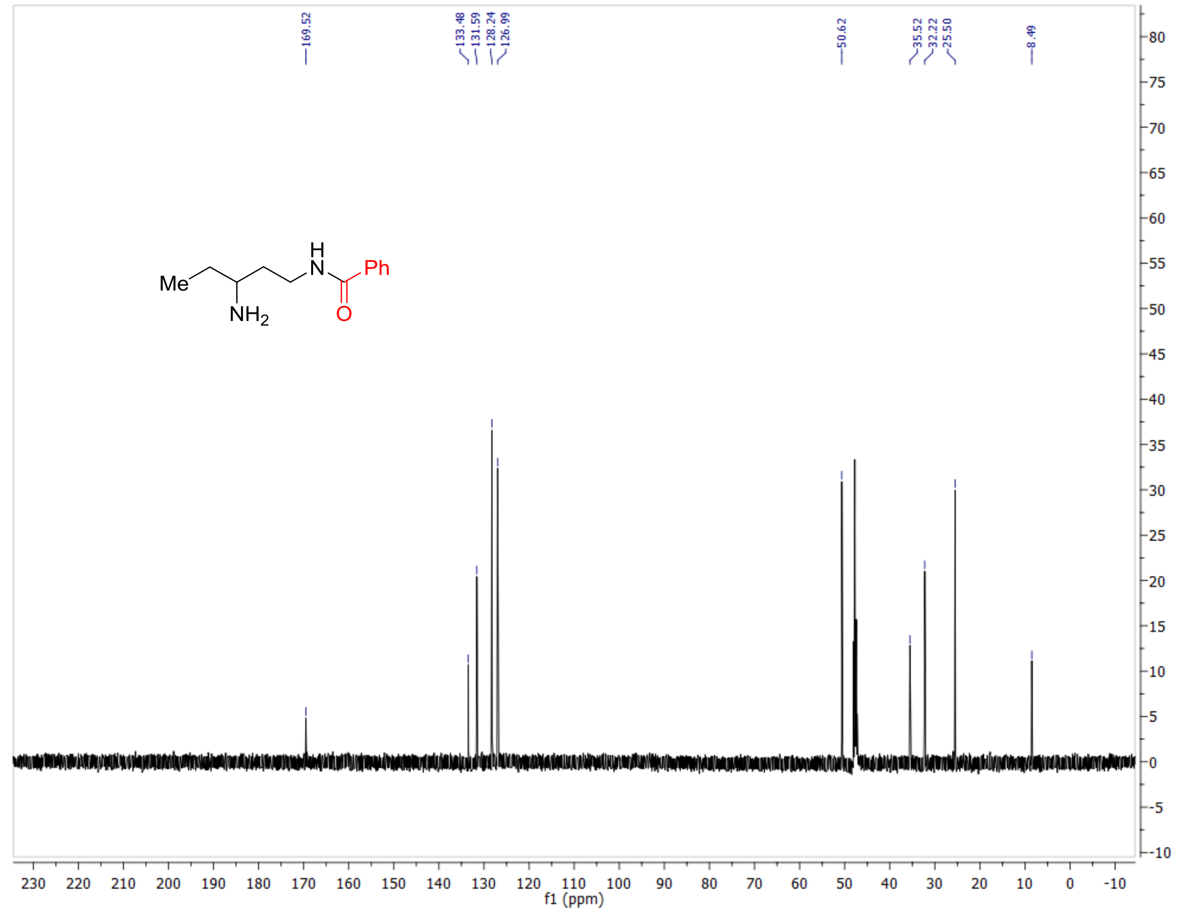

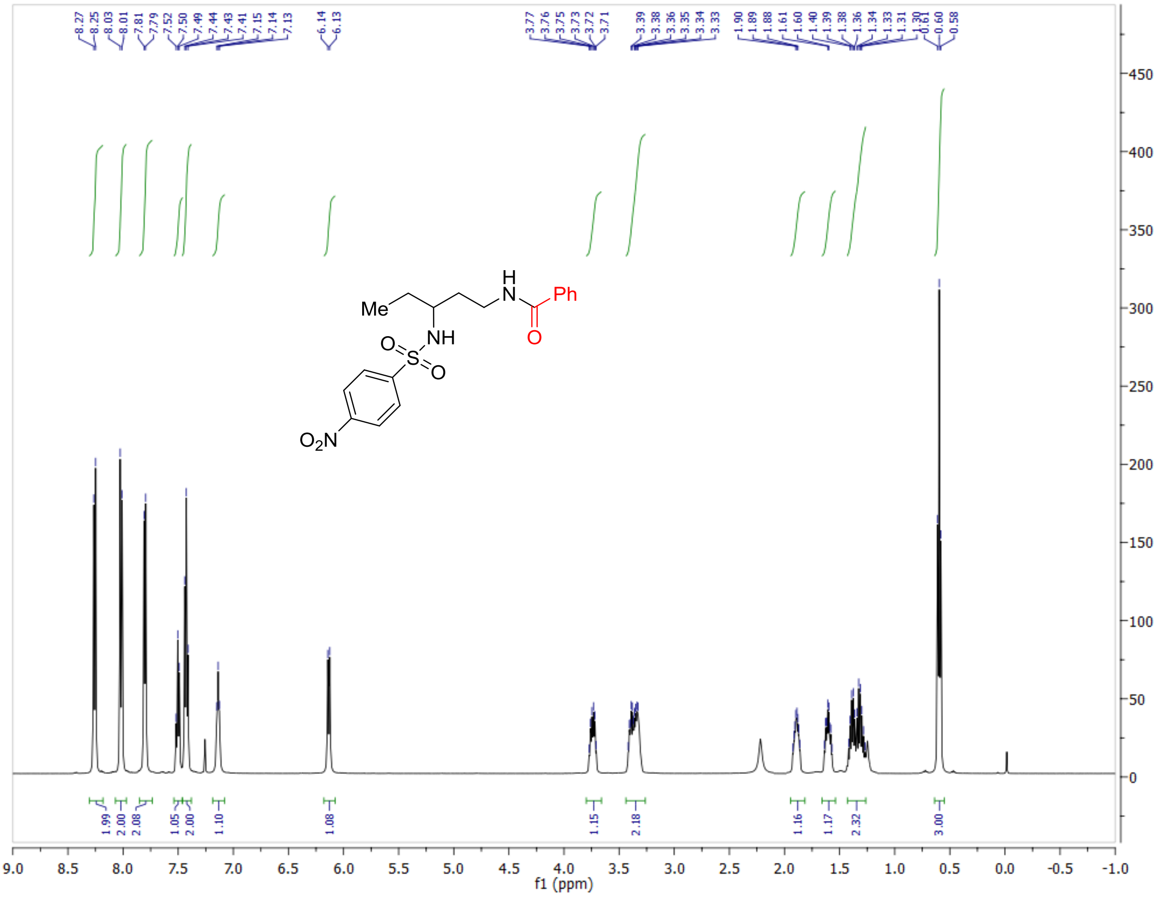
Me
H
N
Ph
O
NH
O
S
O
O2N
f1 (ppm)

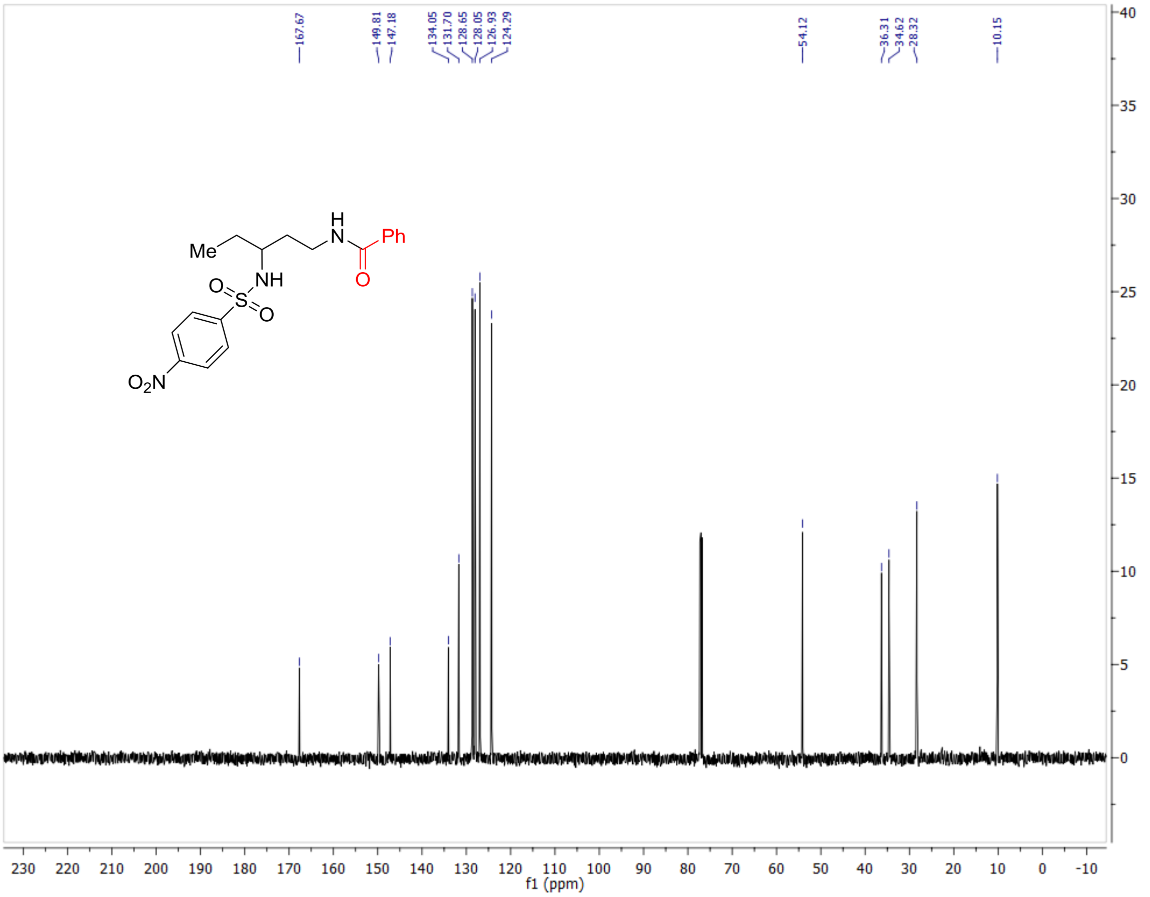
Me
H
N
Ph
O
NH
O
S
O
O2N
f1 (ppm)

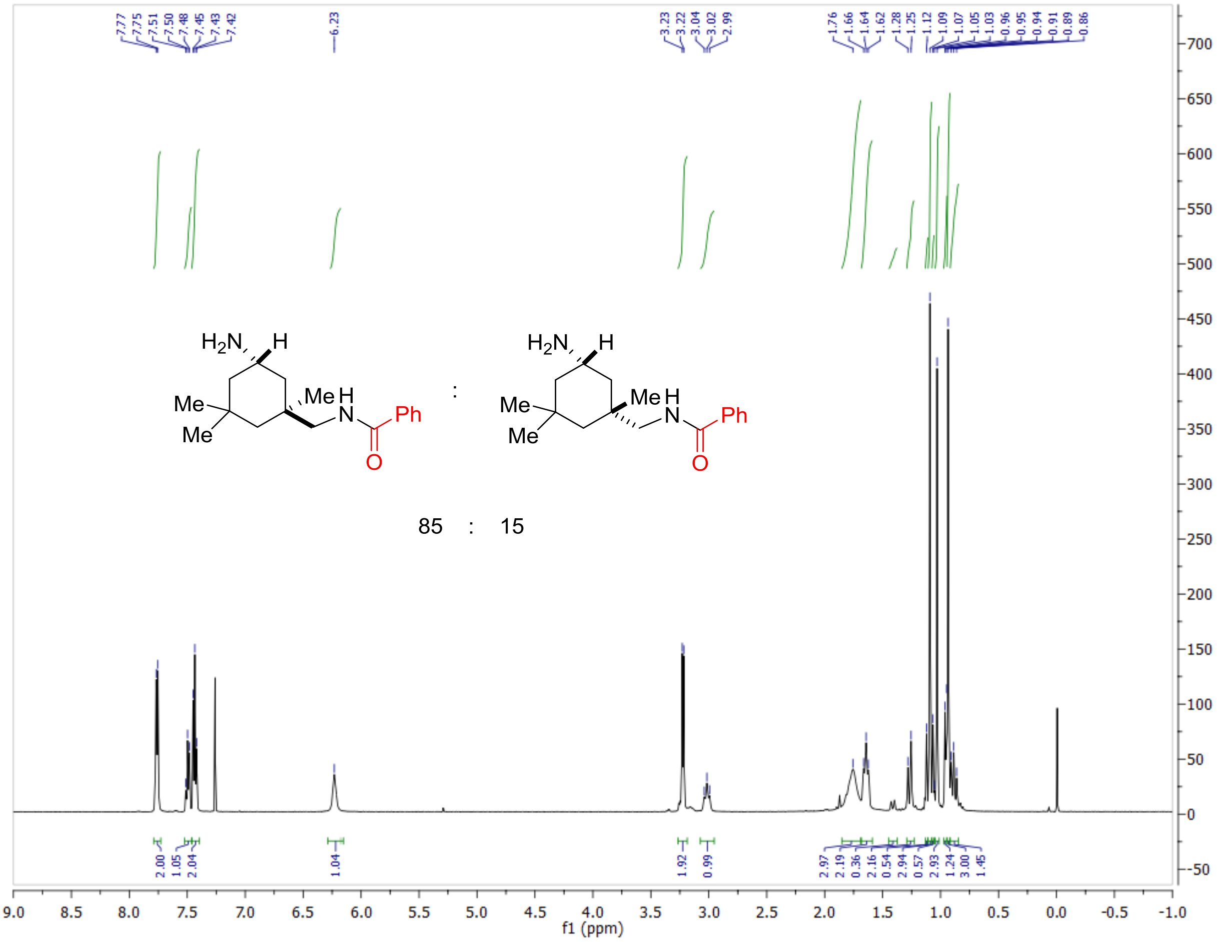
7.77
7.75
7.51
7.50
7.48
7.45
7.43
7.42
6.23
3.23
3.22
3.04
3.02
2.99
1.76
1.66
1.64
1.62
1.28
1.25
1.12
1.09
1.07
1.05
1.03
0.96
0.95
0.94
0.91
0.89
0.86
85 : 15
2.00
1.05
2.04
1.04
1.92
0.99
2.97
2.19
0.36
2.16
0.54
2.94
0.57
2.93
1.24
3.00
1.45
f1 (ppm)

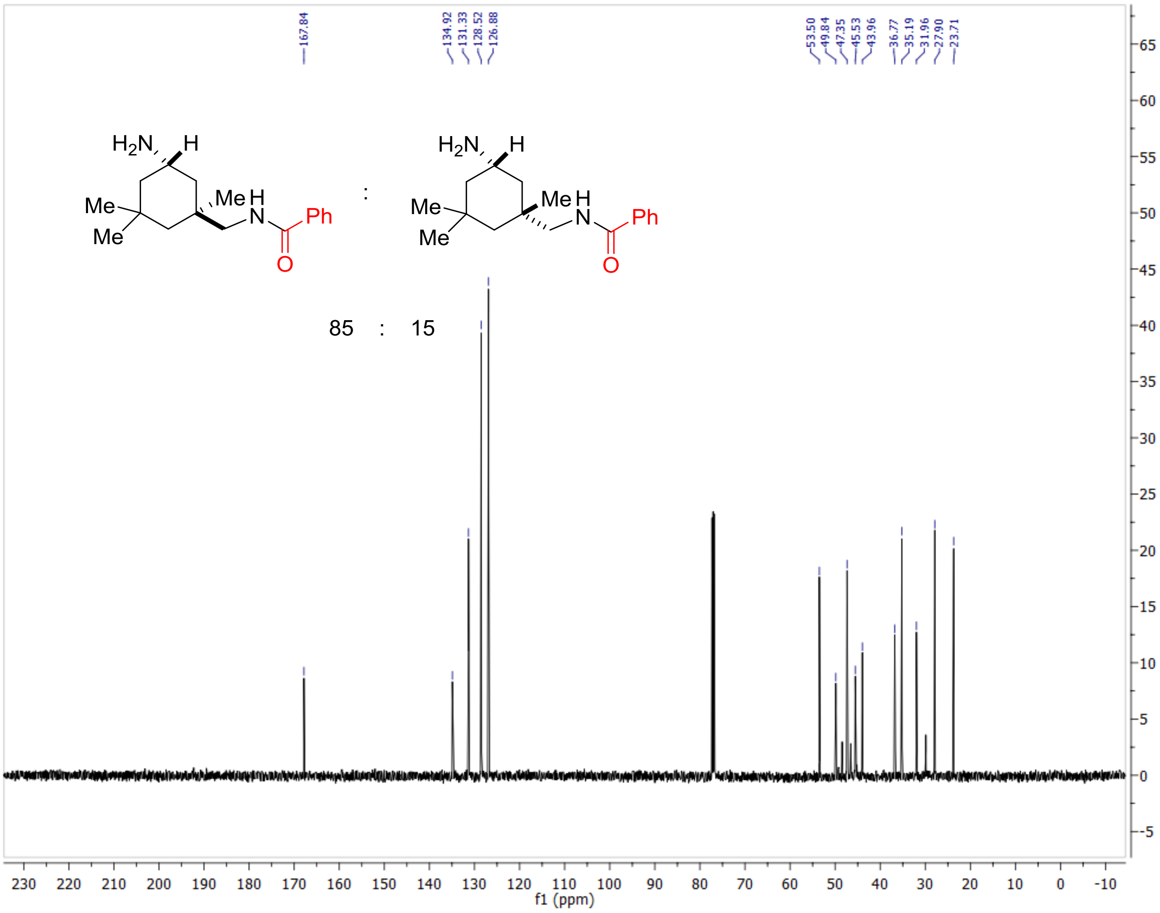
167.84
134.92
131.33
128.52
126.88
53.50
49.84
47.35
45.53
43.96
36.77
35.19
31.96
27.90
23.71
85 : 15
f1 (ppm)

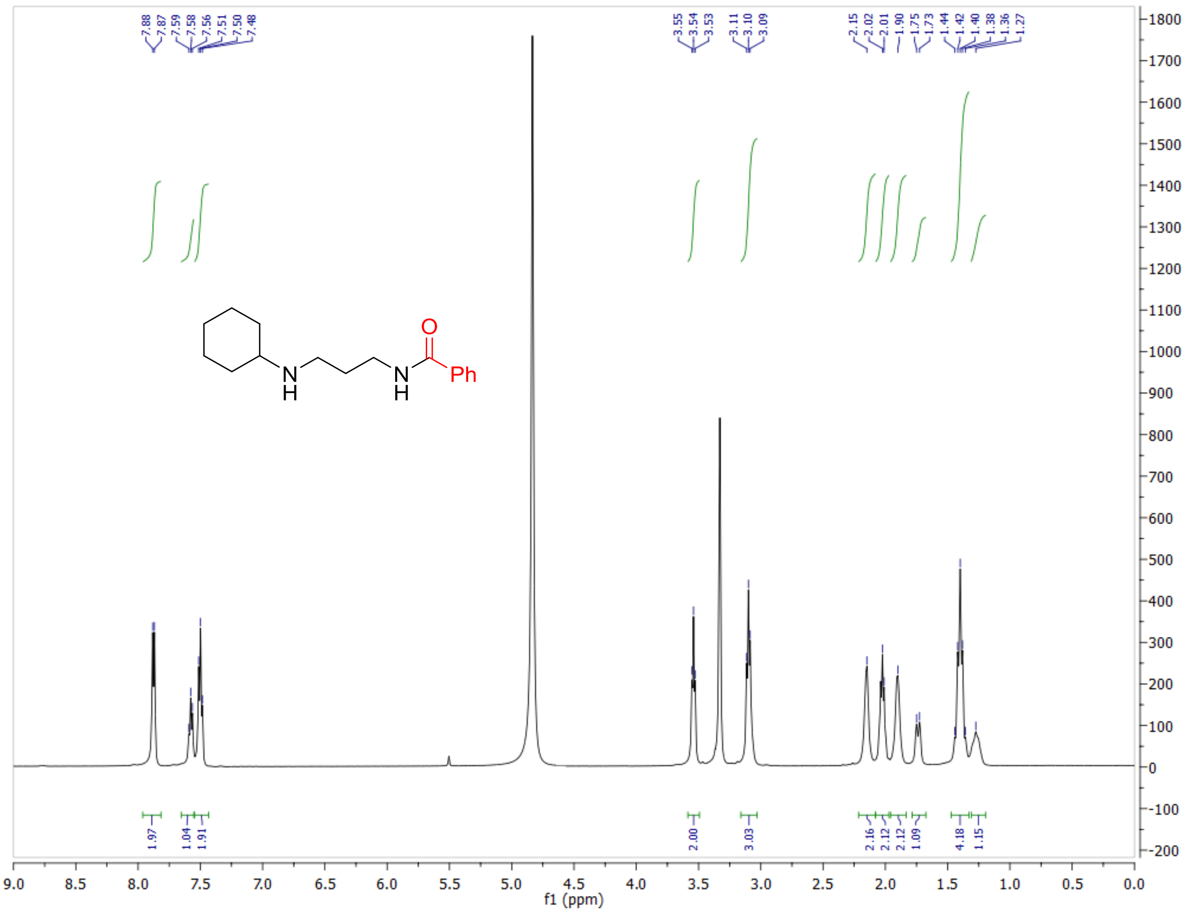
7.88
7.87
7.59
7.58
7.56
7.51
7.50
7.48
3.55
3.54
3.53
3.11
3.10
3.09
2.15
2.02
2.01
1.90
1.75
1.73
1.44
1.42
1.40
1.38
1.36
1.27
N
H
N
H
O
Ph
1.97
1.04
1.91
2.00
3.03
2.16
2.12
2.12
1.09
4.18
1.15
f1 (ppm)

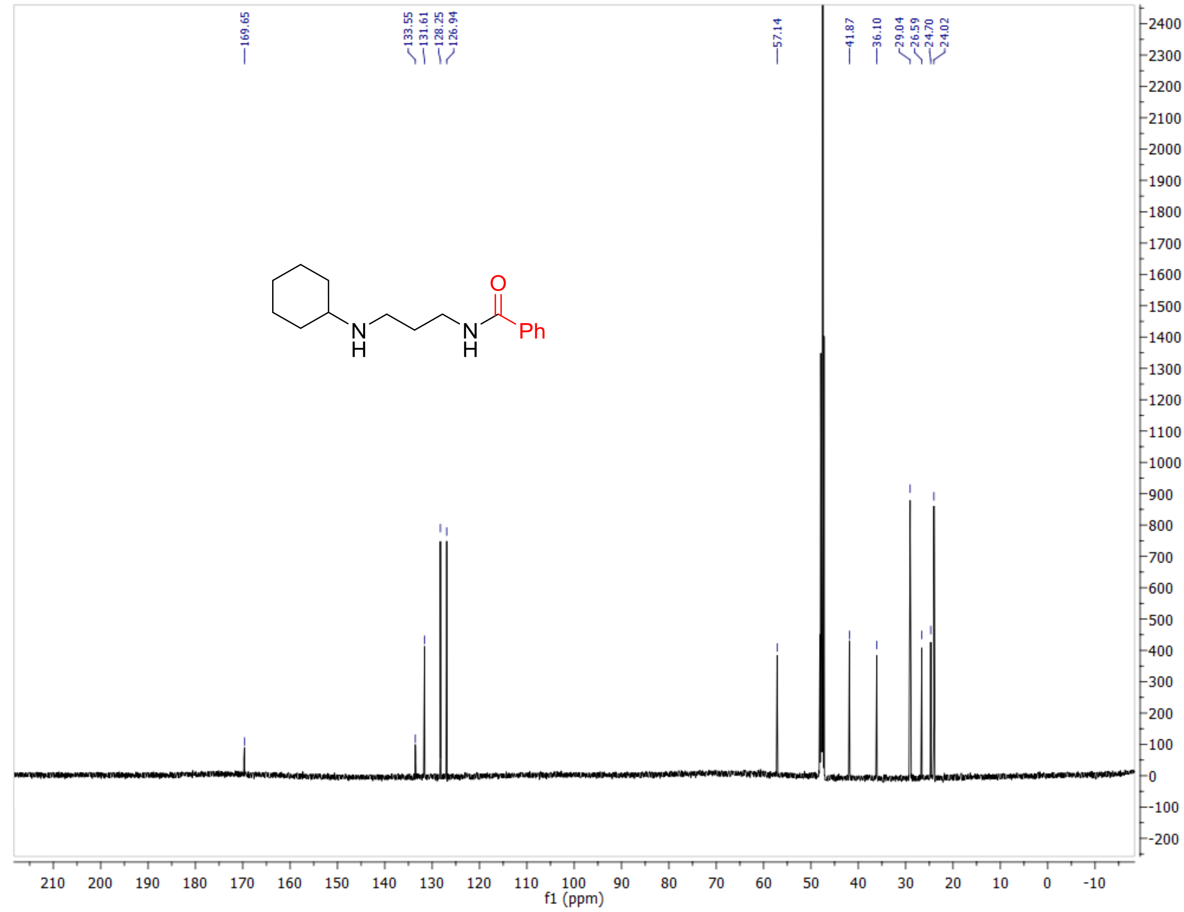
169.65
133.55
131.61
128.25
126.94
57.14
41.87
36.10
29.04
26.59
24.70
24.02
N
H
N
H
O
Ph
f1 (ppm)

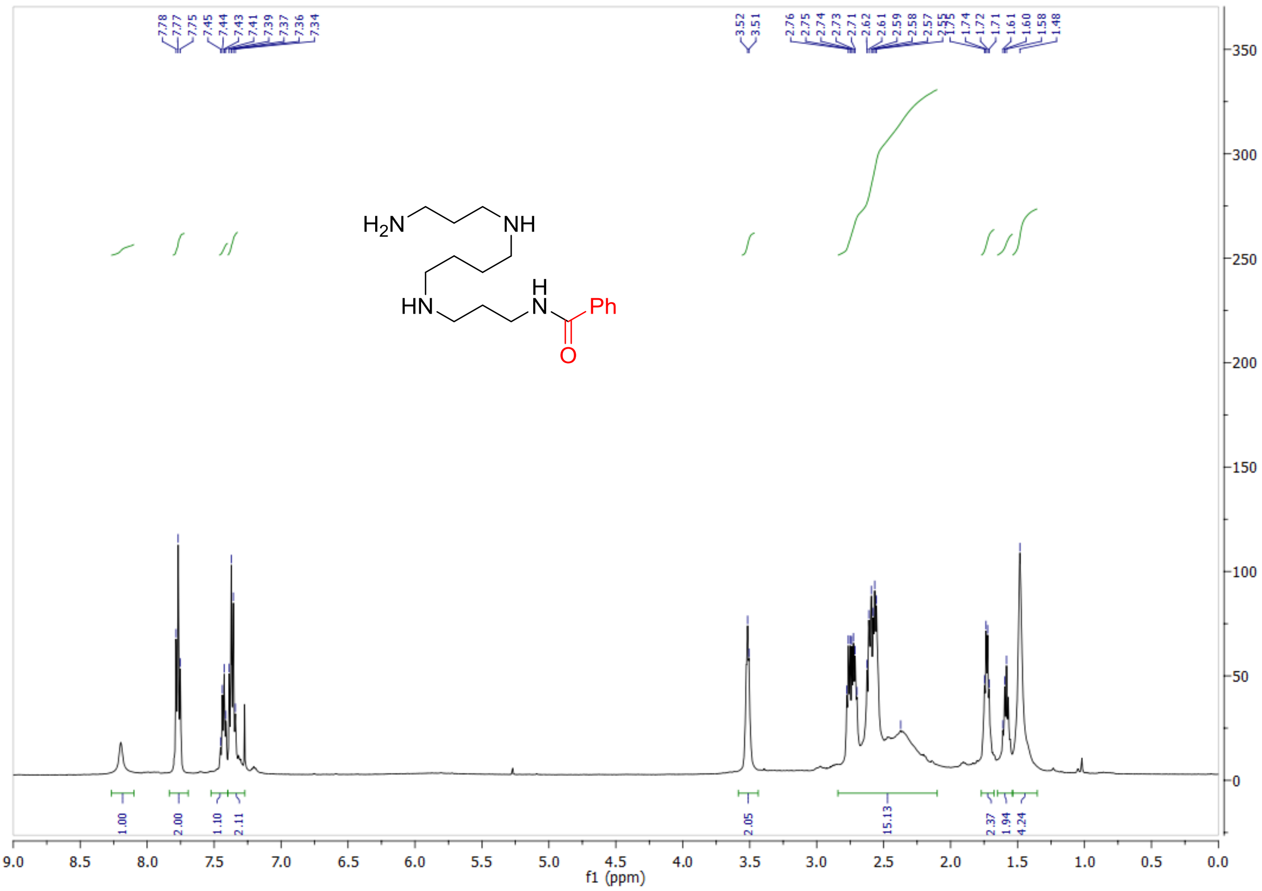
H2N
NH
HN
H
N
Ph
O
f1 (ppm)

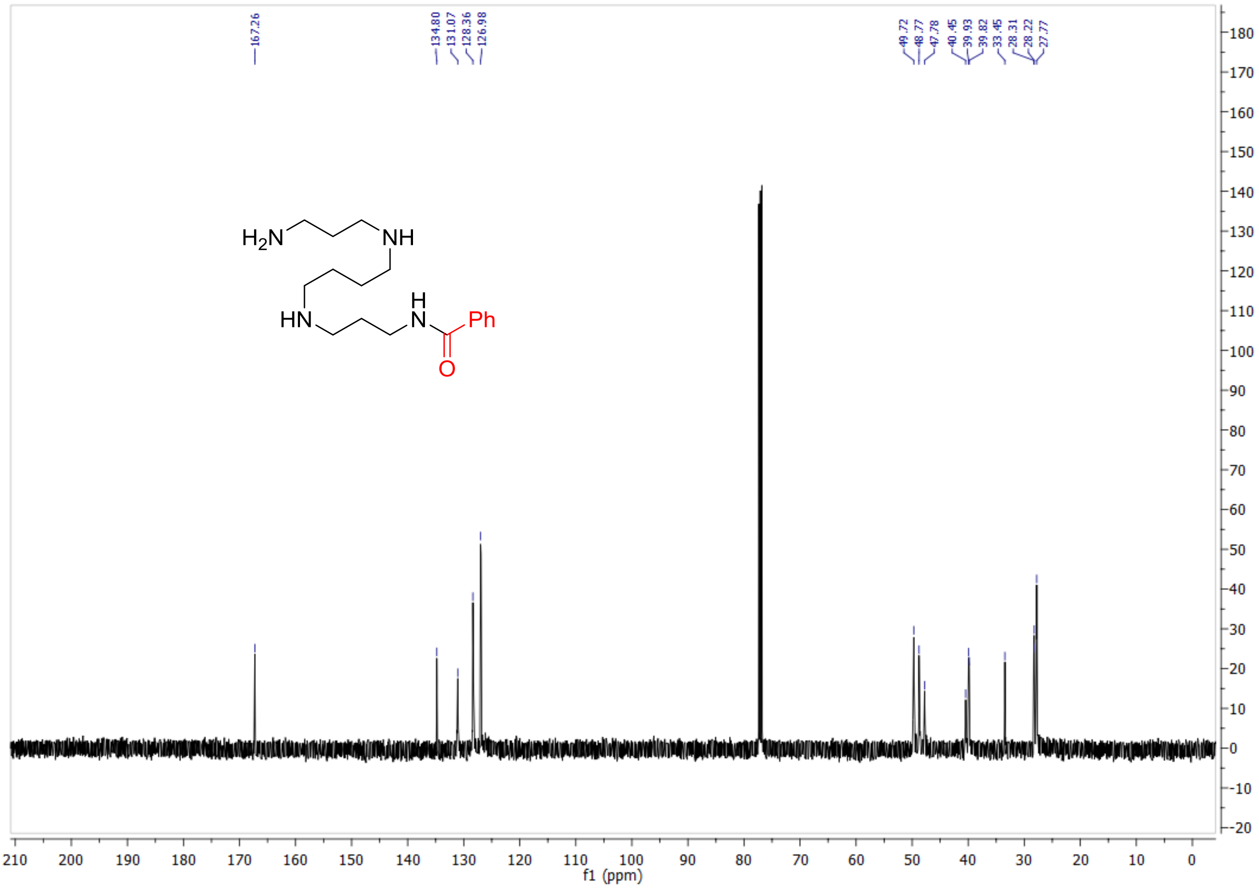
167.26
134.80
131.07
128.36
126.98
H2N
NH
HN
H
N
Ph
O
f1 (ppm)

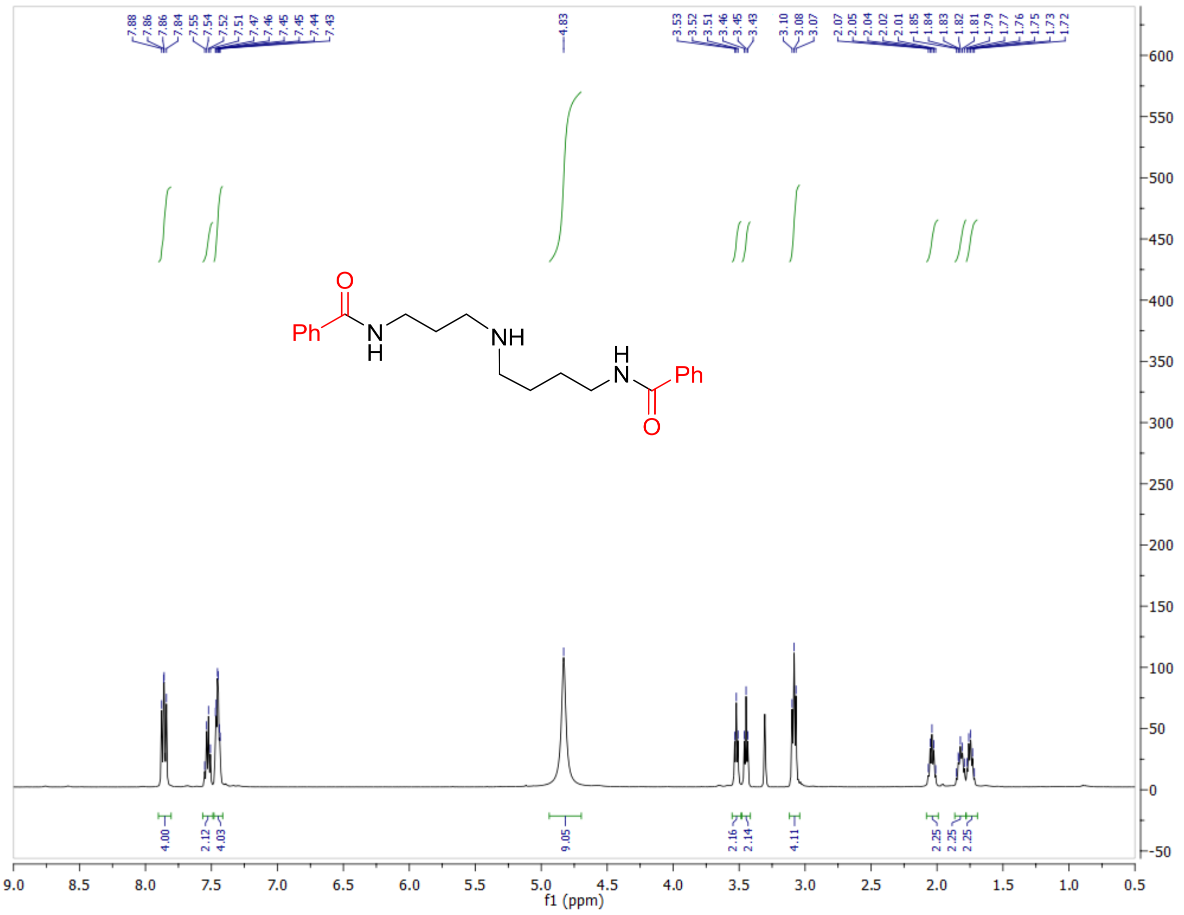
7.88
7.86
7.86
7.84
7.55
7.54
7.52
7.51
7.47
7.46
7.45
7.45
7.44
7.43
4.83
3.53
3.52
3.51
3.46
3.45
3.43
3.10
3.08
3.07
2.07
2.05
2.04
2.02
2.01
1.85
1.84
1.83
1.82
1.81
1.79
1.77
1.76
1.75
1.73
1.72
O
Ph
N
H
NH
H
N
Ph
O
4.00
2.12
4.03
9.05
2.16
2.14
4.11
2.25
2.25
2.25
f1 (ppm)

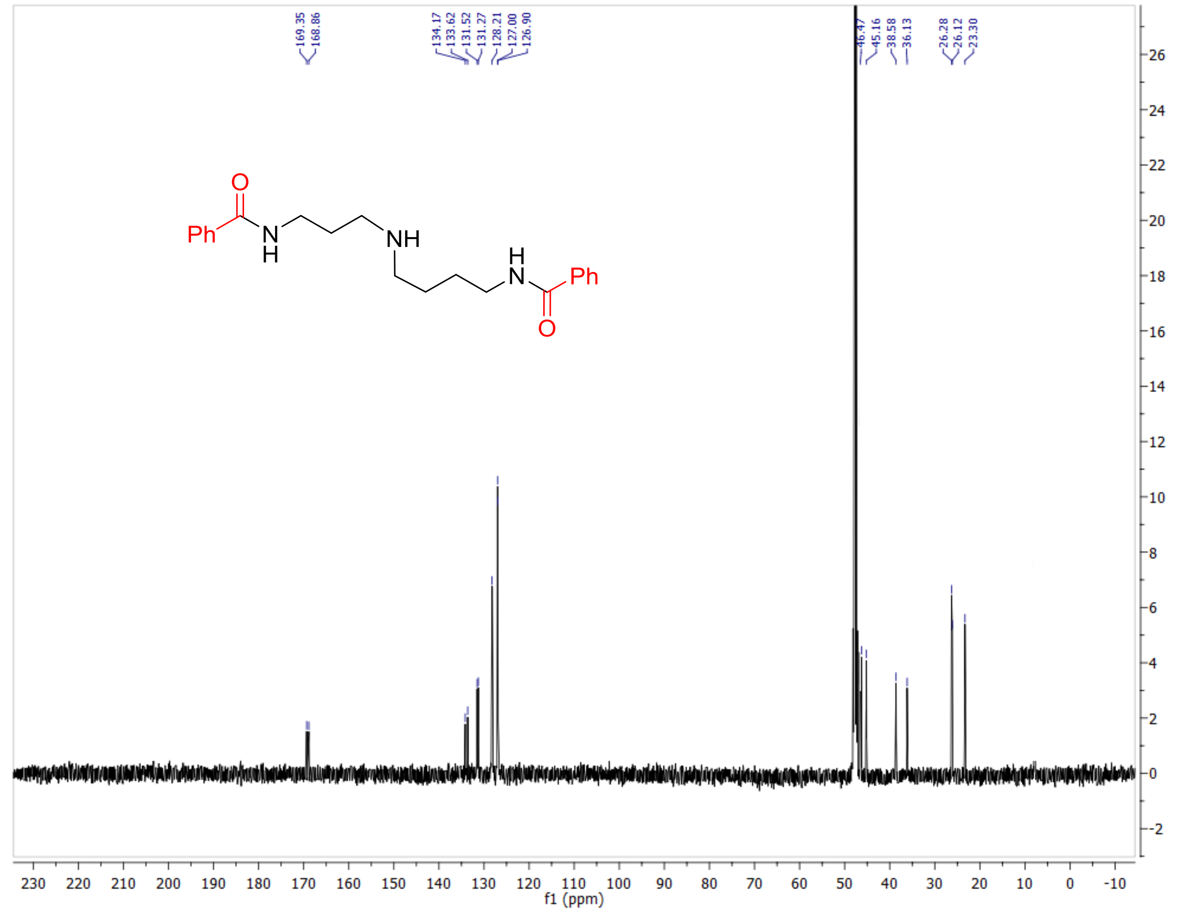
169.35
168.86
134.17
133.62
131.52
131.27
128.21
127.00
126.90
46.47
45.16
38.58
36.13
26.28
26.12
23.30
O
Ph
N
H
NH
H
N
Ph
O
f1 (ppm)

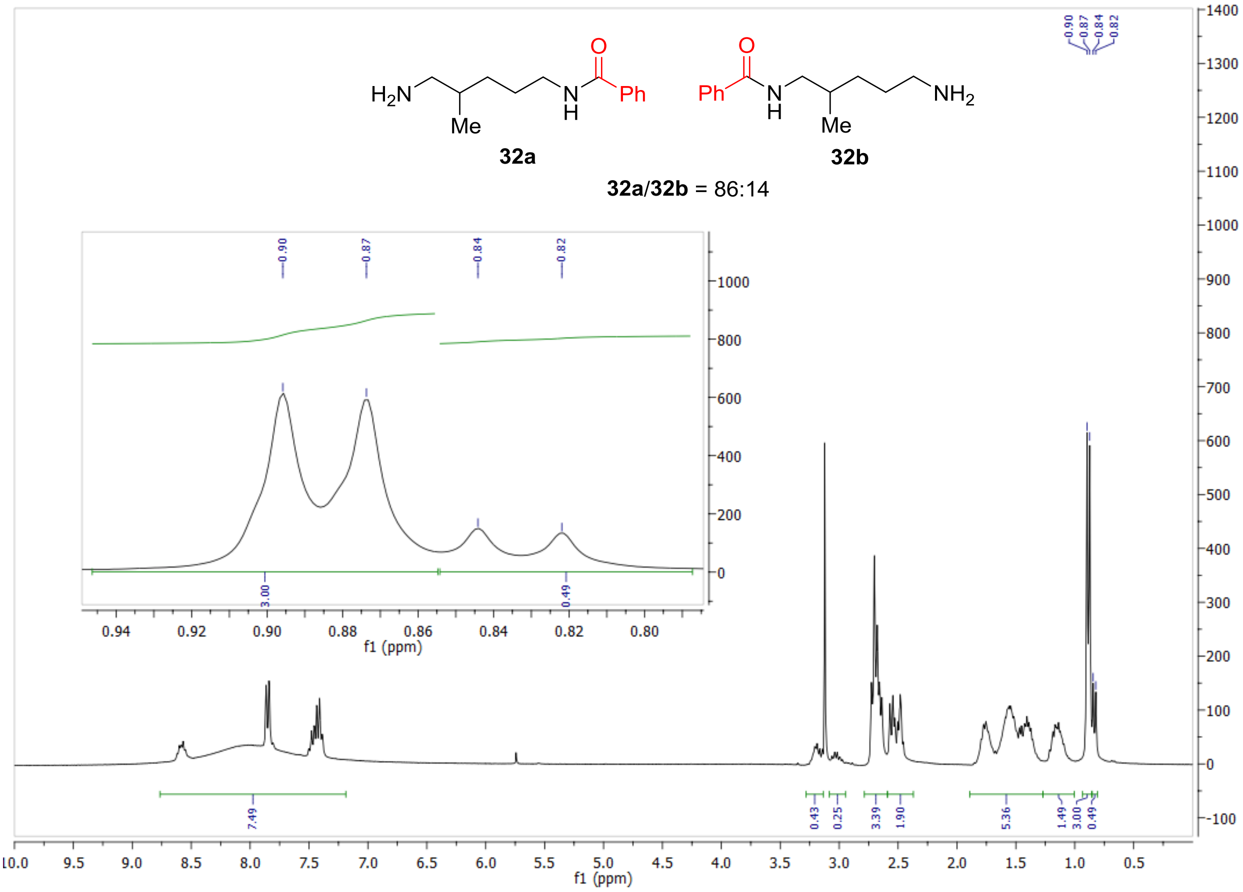

32a
32b
32a/32b = 86:14
f1 (ppm)

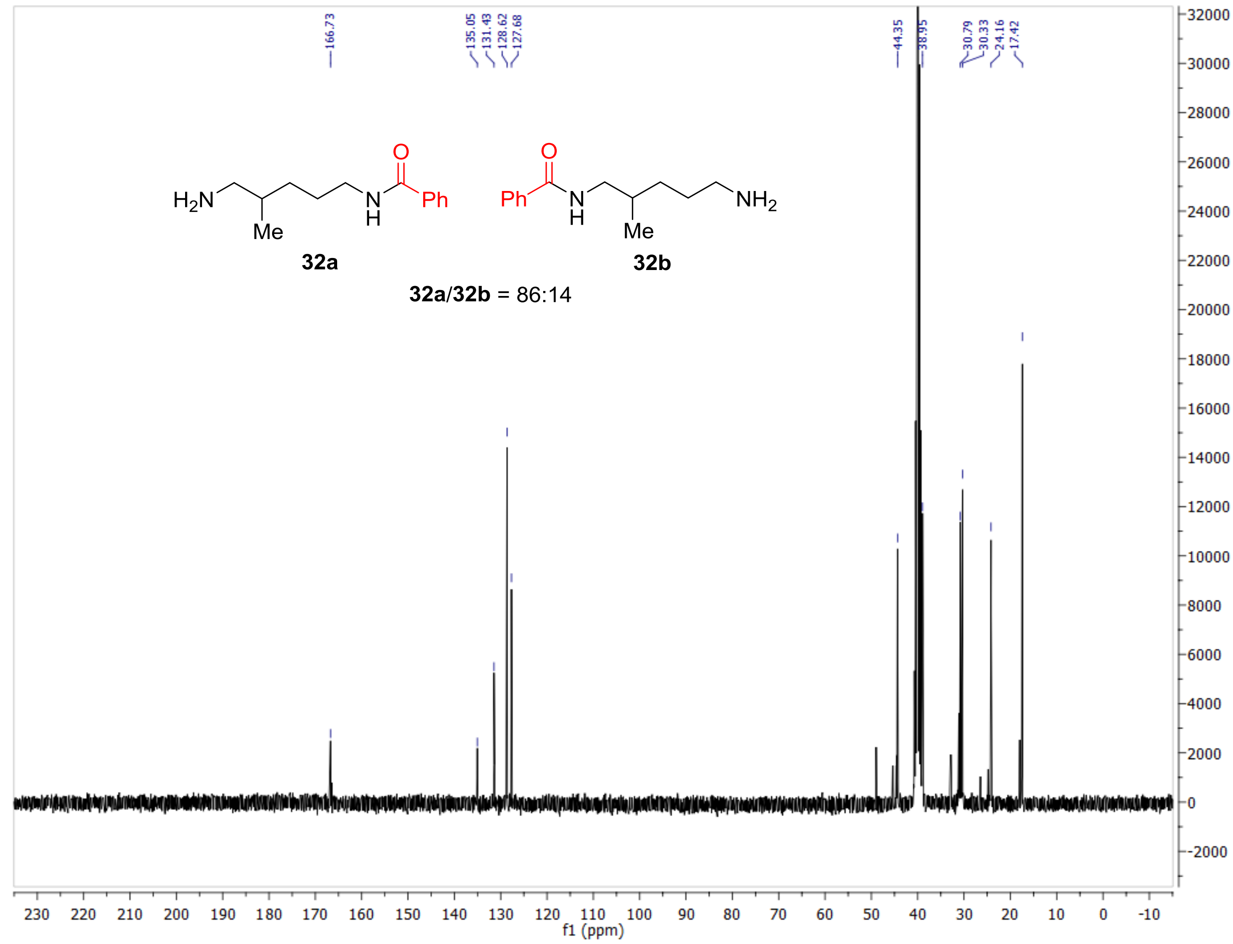

32a
32b
32a/32b = 86:14
f1 (ppm)

Selective 1D TOCSY ^{1}H NMR experiment for the mixture of **32a** and **32b**:

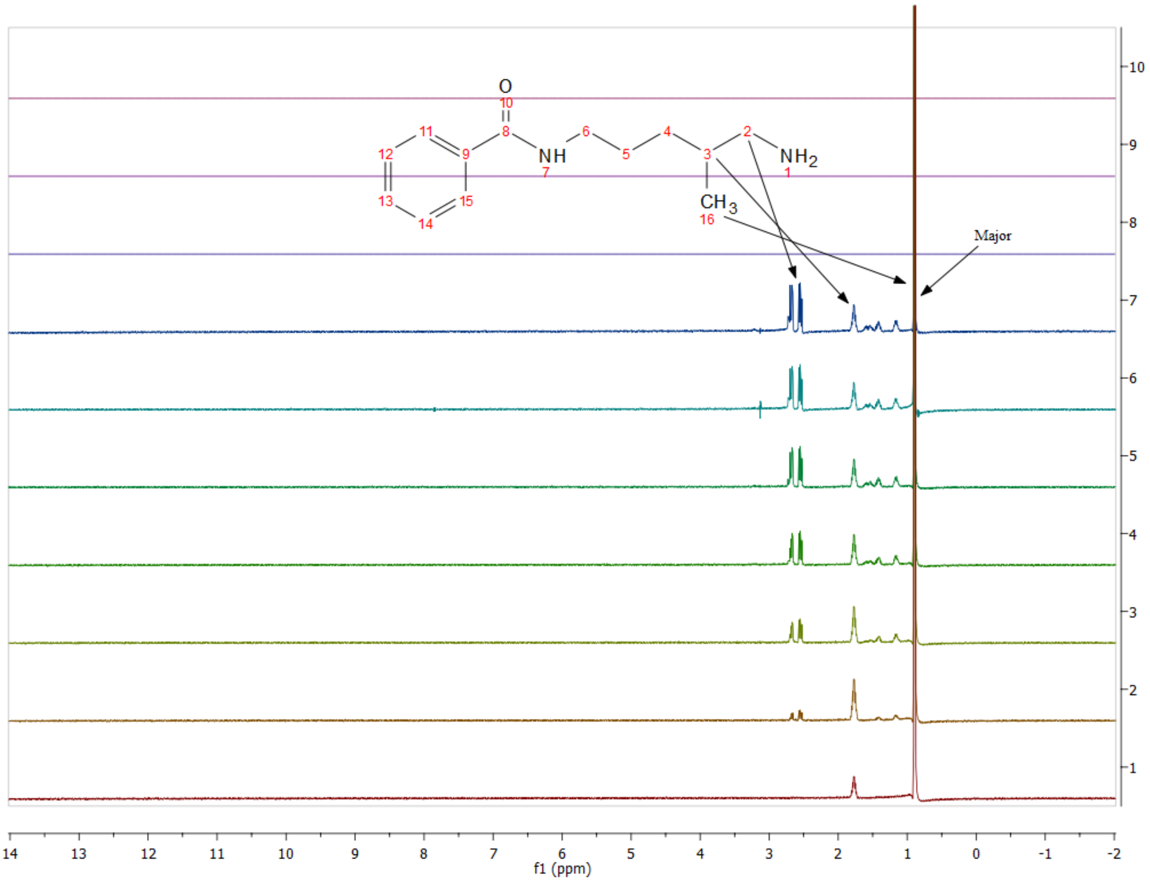

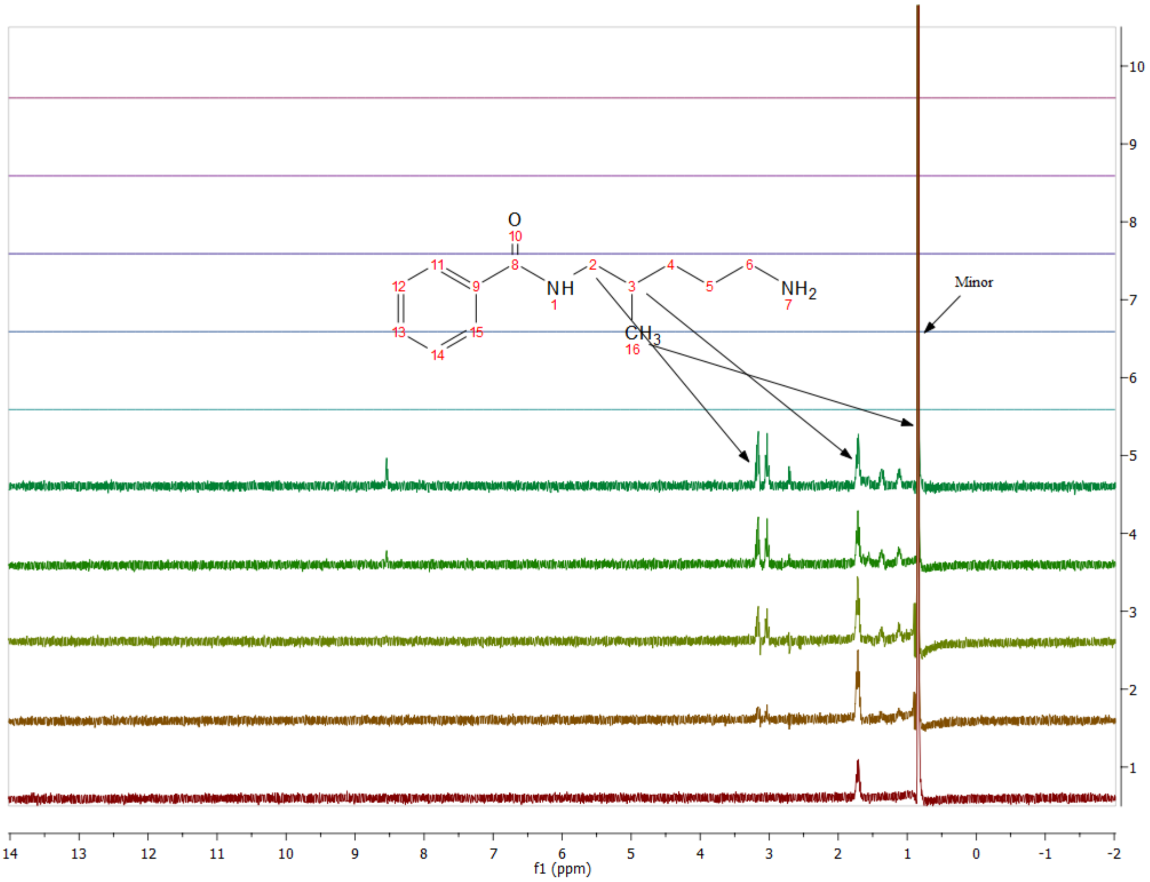

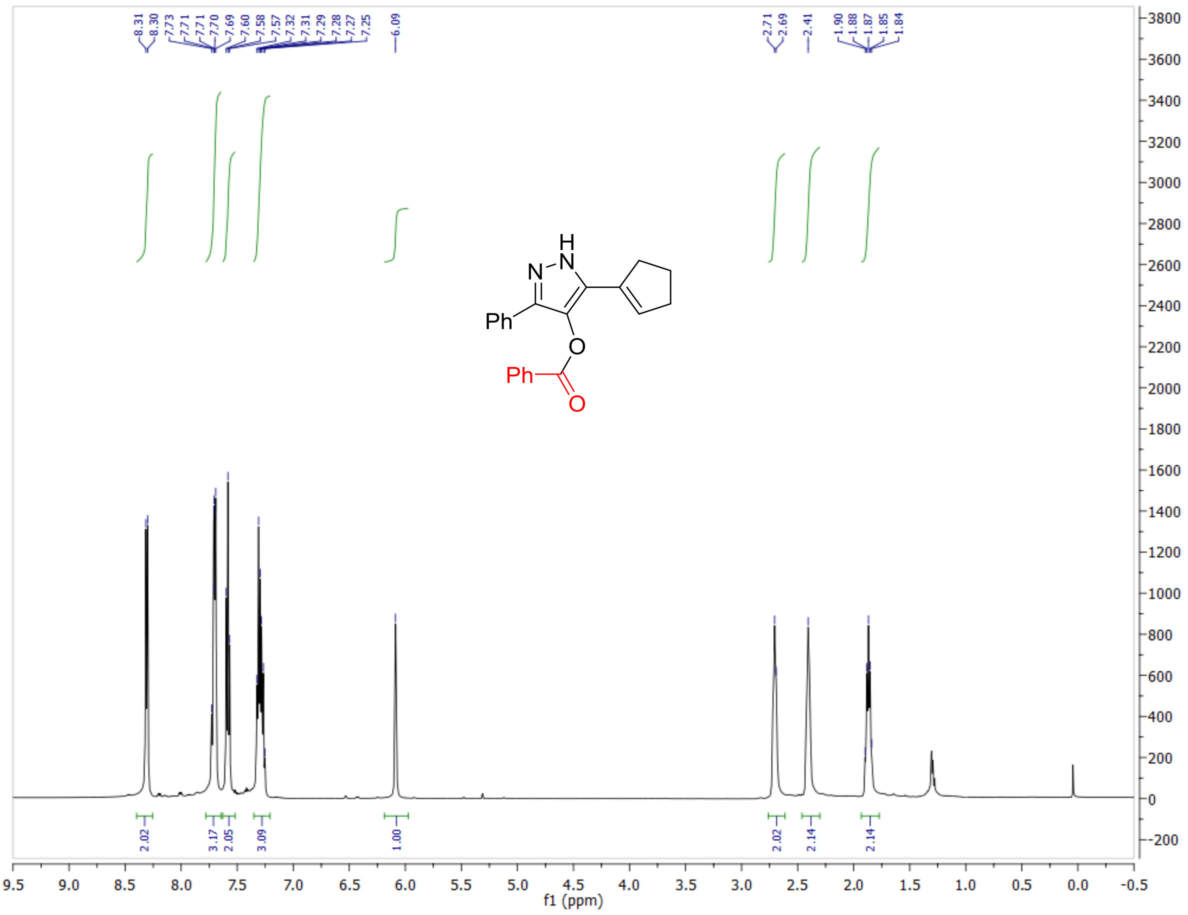
8.31
8.30
7.73
7.71
7.71
7.70
7.69
7.60
7.58
7.57
7.32
7.31
7.29
7.28
7.27
7.25
6.09
2.71
2.69
2.41
1.90
1.88
1.87
1.85
1.84
Ph
Ph
N
N
H
O
O
2.02
3.17
2.05
3.09
1.00
2.02
2.14
2.14
f1 (ppm)

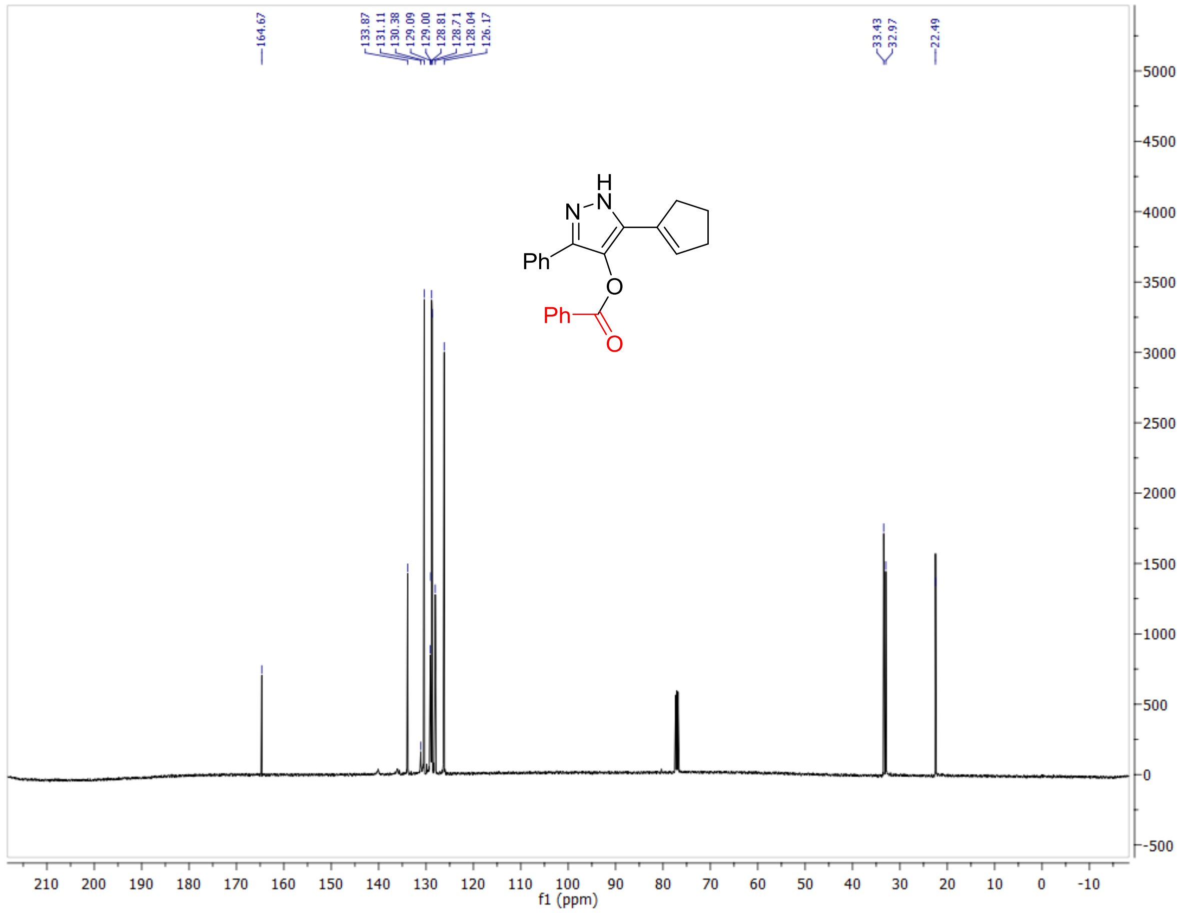
164.67
133.87
131.11
130.38
129.09
129.00
128.81
128.71
128.04
126.17
33.43
32.97
22.49
Ph
Ph
N
N
H
O
O
f1 (ppm)

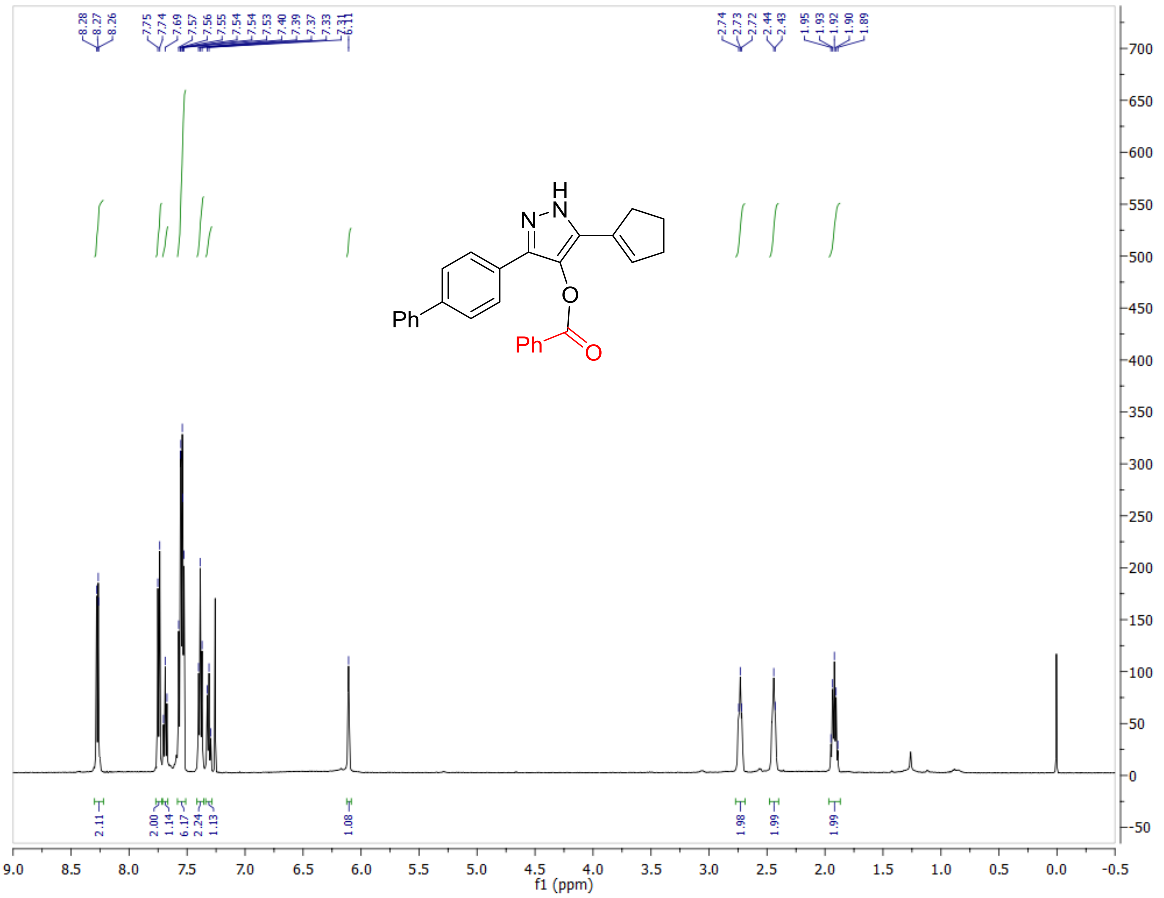
8.28
8.27
8.26
7.75
7.74
7.69
7.57
7.56
7.55
7.54
7.54
7.53
7.40
7.39
7.37
7.33
7.31
6.11
2.74
2.73
2.72
2.44
2.43
1.95
1.93
1.92
1.90
1.89
2.11
2.00
1.14
6.17
2.24
1.13
1.08
1.98
1.99
1.99
Ph
Ph
O
O
N
N
H
f1 (ppm)

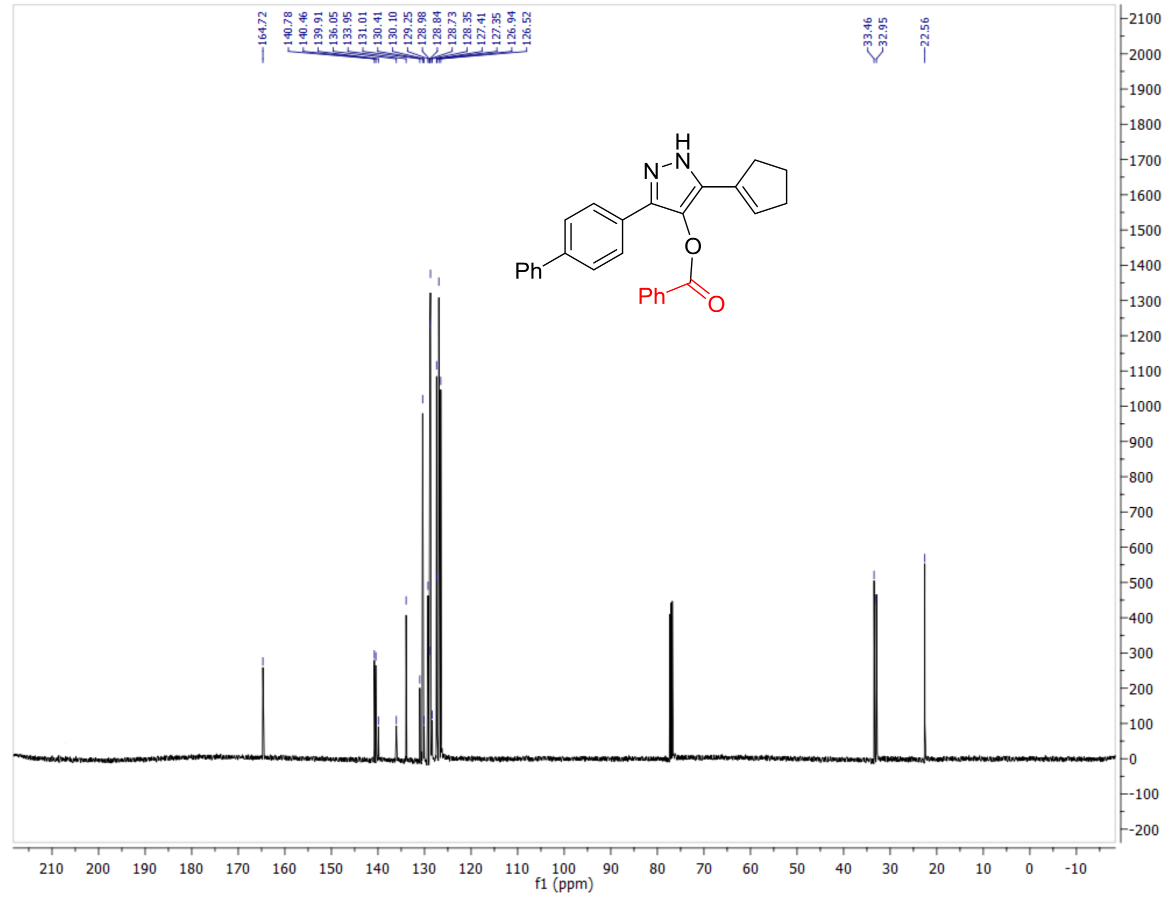
164.72
140.78
140.46
139.91
136.05
133.95
131.01
130.41
130.10
129.25
128.98
128.84
128.73
128.35
127.41
127.35
126.94
126.52
33.46
32.95
22.56
Ph
Ph
O
O
N
N
H
f1 (ppm)

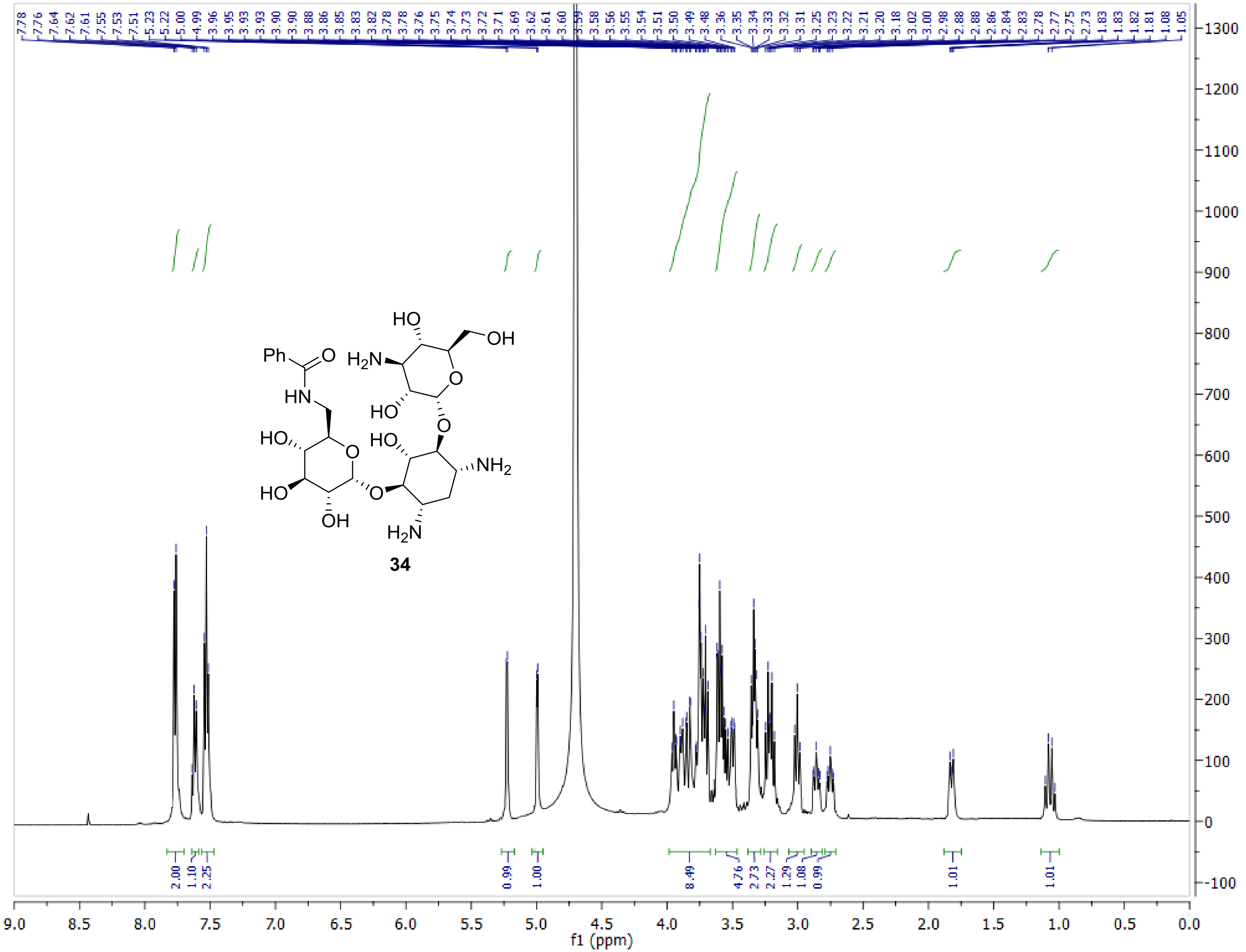
Ph
O
HN
HO
HO
OH
O
O
H2N
HO
OH
O
HO
O
HO
NH2
H2N
34
f1 (ppm)

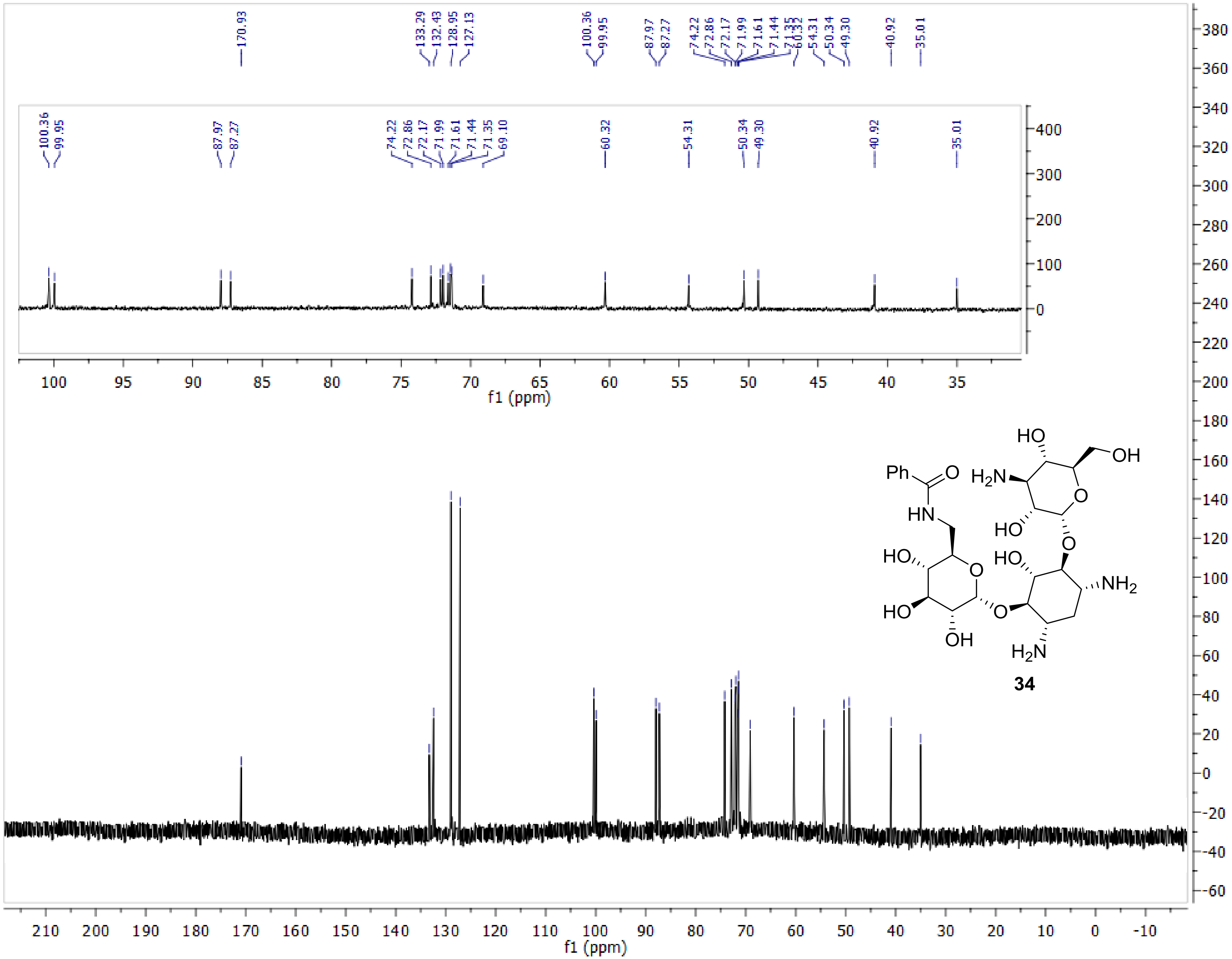
170.93
133.29
132.43
128.95
127.13
100.36
99.95
87.97
87.27
74.22
72.86
72.17
71.99
71.61
71.44
71.35
60.32
54.31
50.34
49.30
40.92
35.01
69.10
f1 (ppm)
Ph
O
HN
HO
HO
OH
H2N
NH2
OH
34

Kanamycin (**33**) – blue (CH) red (CH_2)
N-6'-Benzoylkanamycin (**34**) green (CH) Black (CH_2)

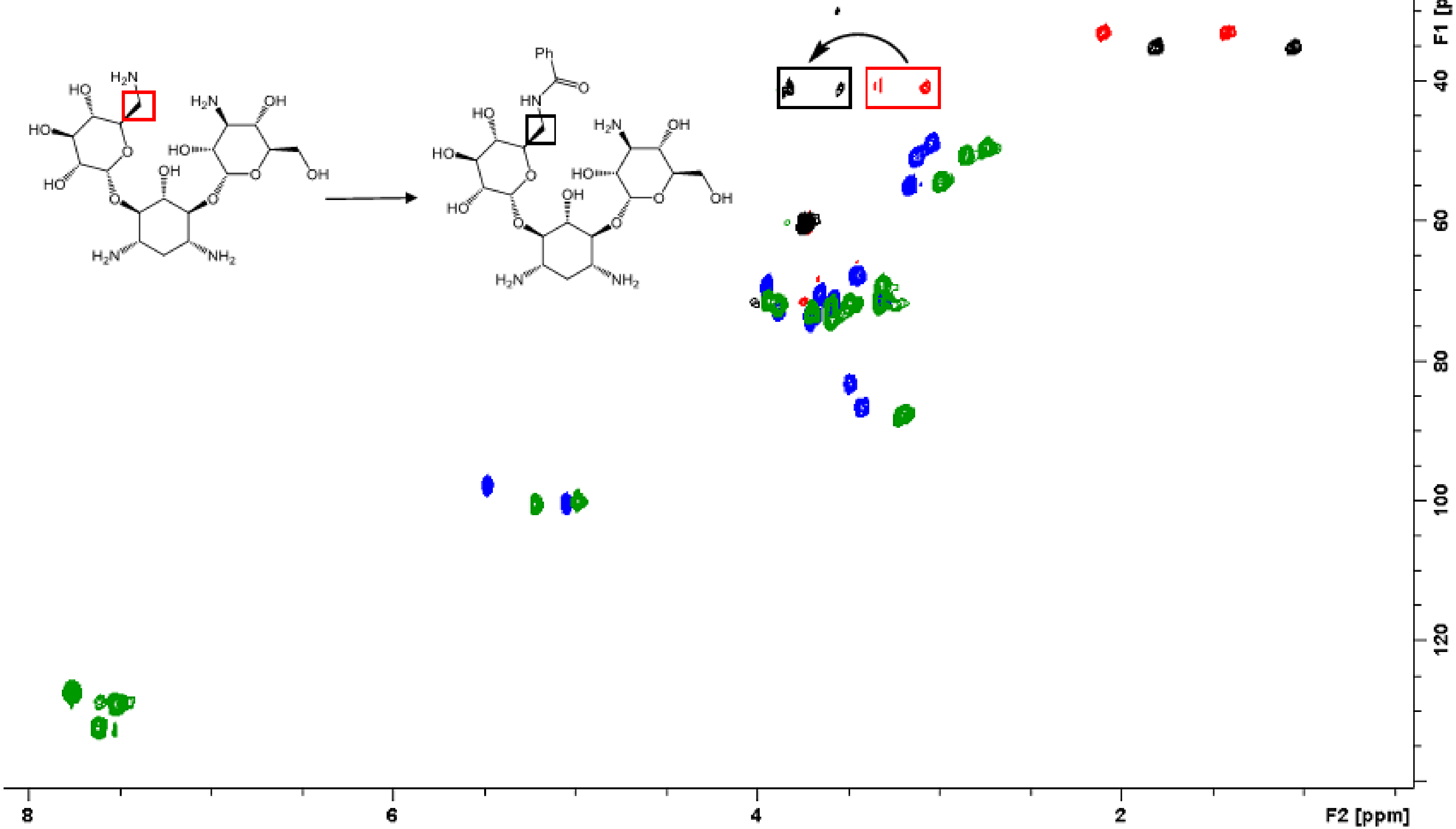

Overlay of the HSQC spectra of kanamycin (**33**) (CH blue, CH_2 red) and *N*-6'-Benzoylkanamycin (**34**) (CH green, CH_2 black). The methylene in the red box are the only peaks to shift down-field due to the anisotropy of the carbonyl of the benzoyl group.

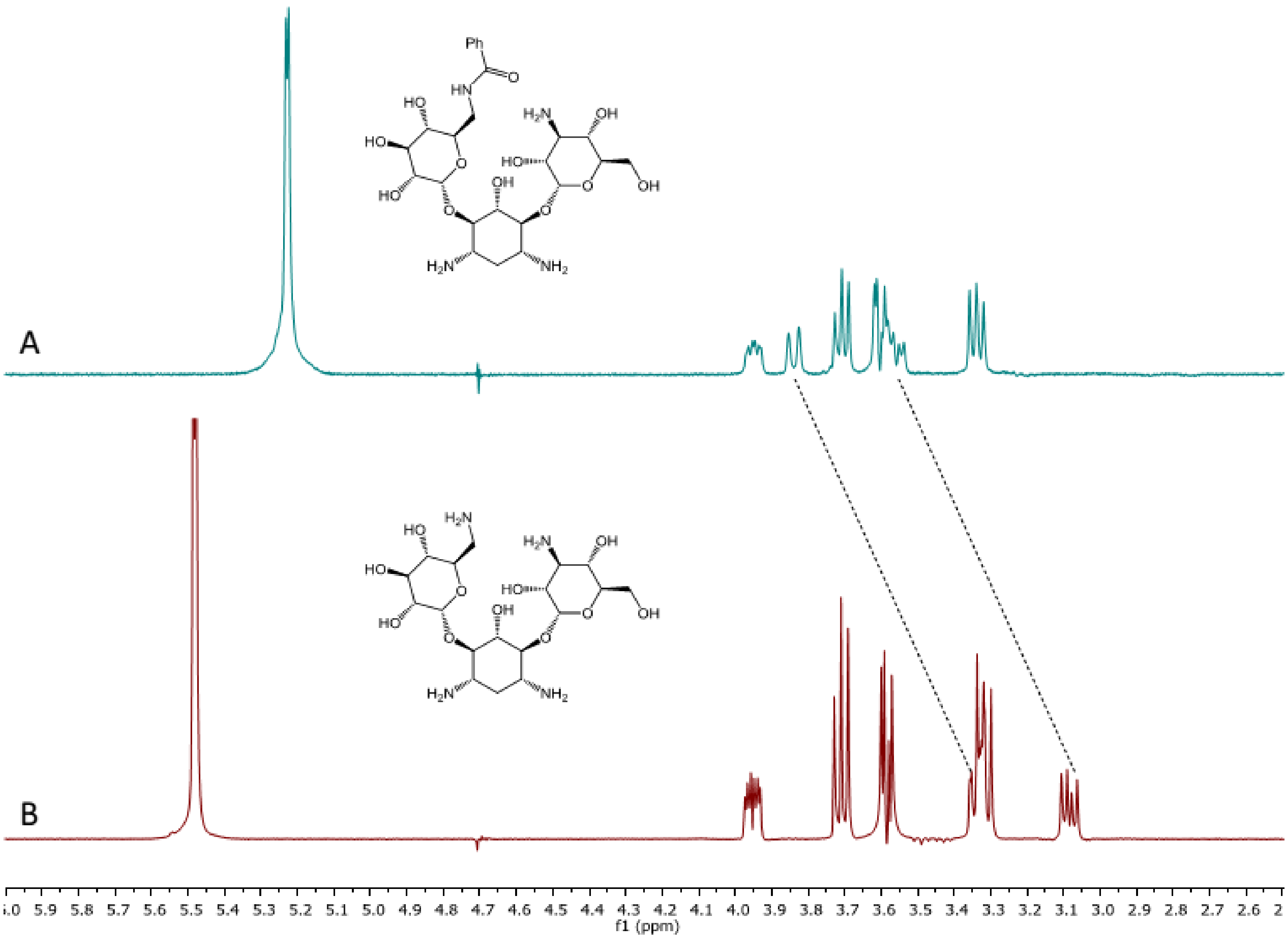

Selective 1D TOCSY spectra of (A) *N*-6'-Benzoylkanamycin (**34**) and (B) Kanamycin (**33**). Dotted lines show the large downfield shift of the benzoylated methylene.

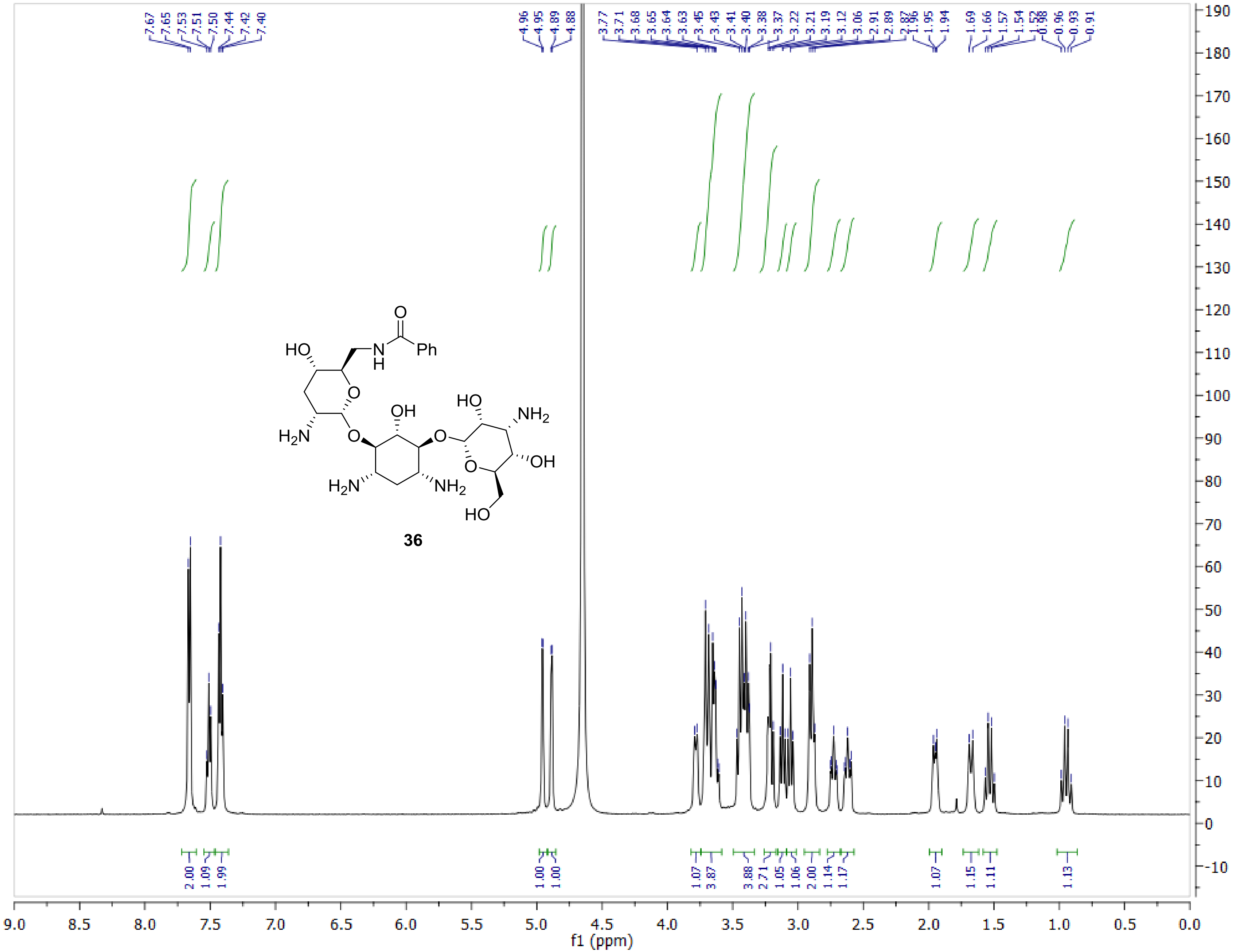
36
f1 (ppm)

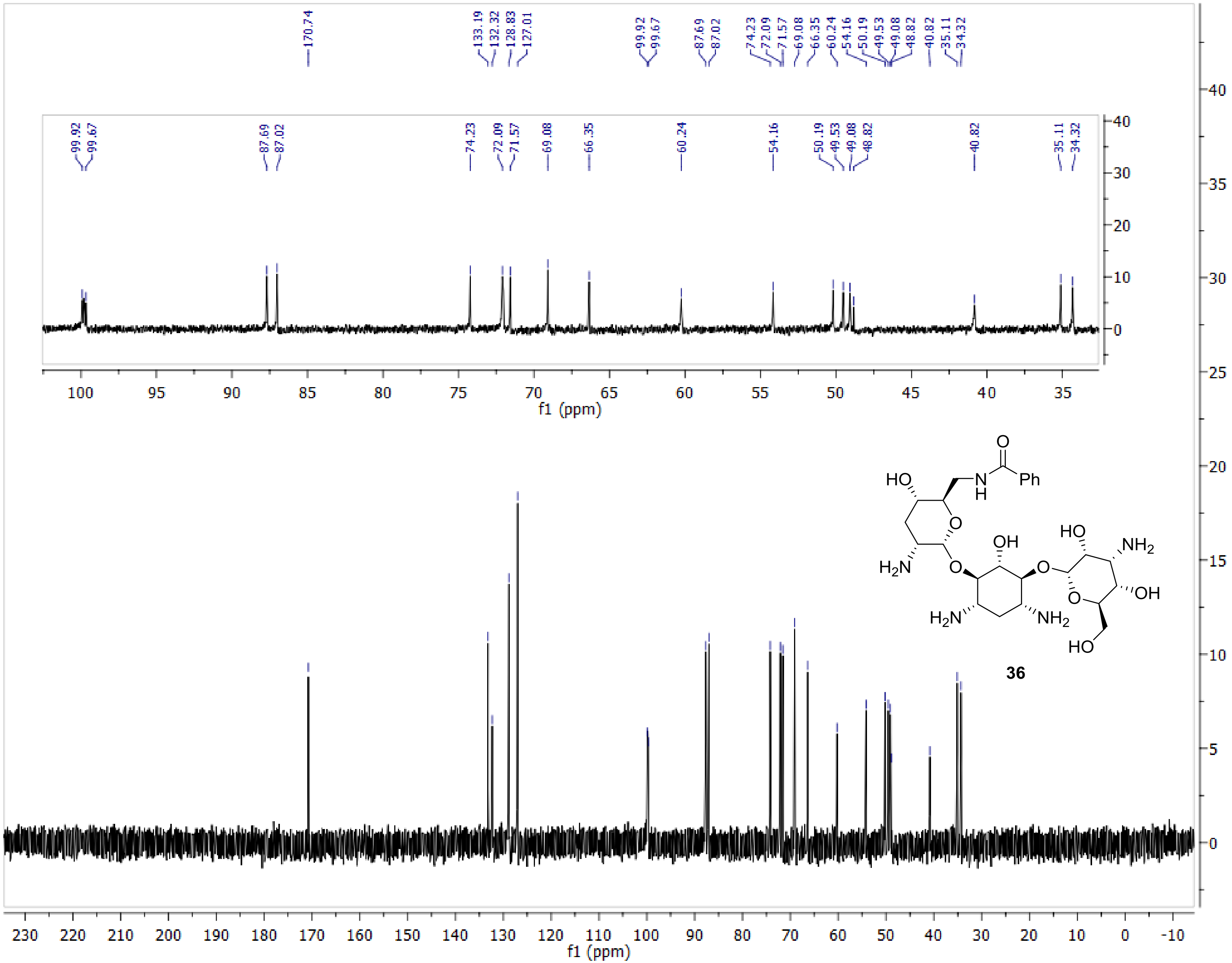
170.74
133.19
132.32
128.83
127.01
99.92
99.67
87.69
87.02
74.23
72.09
71.57
69.08
66.35
60.24
54.16
50.19
49.53
49.08
48.82
40.82
35.11
34.32
f1 (ppm)
f1 (ppm)
HO
N
H
O
Ph
O
H2N
O
OH
HO
NH2
O
O
OH
H2N
NH2
HO
36

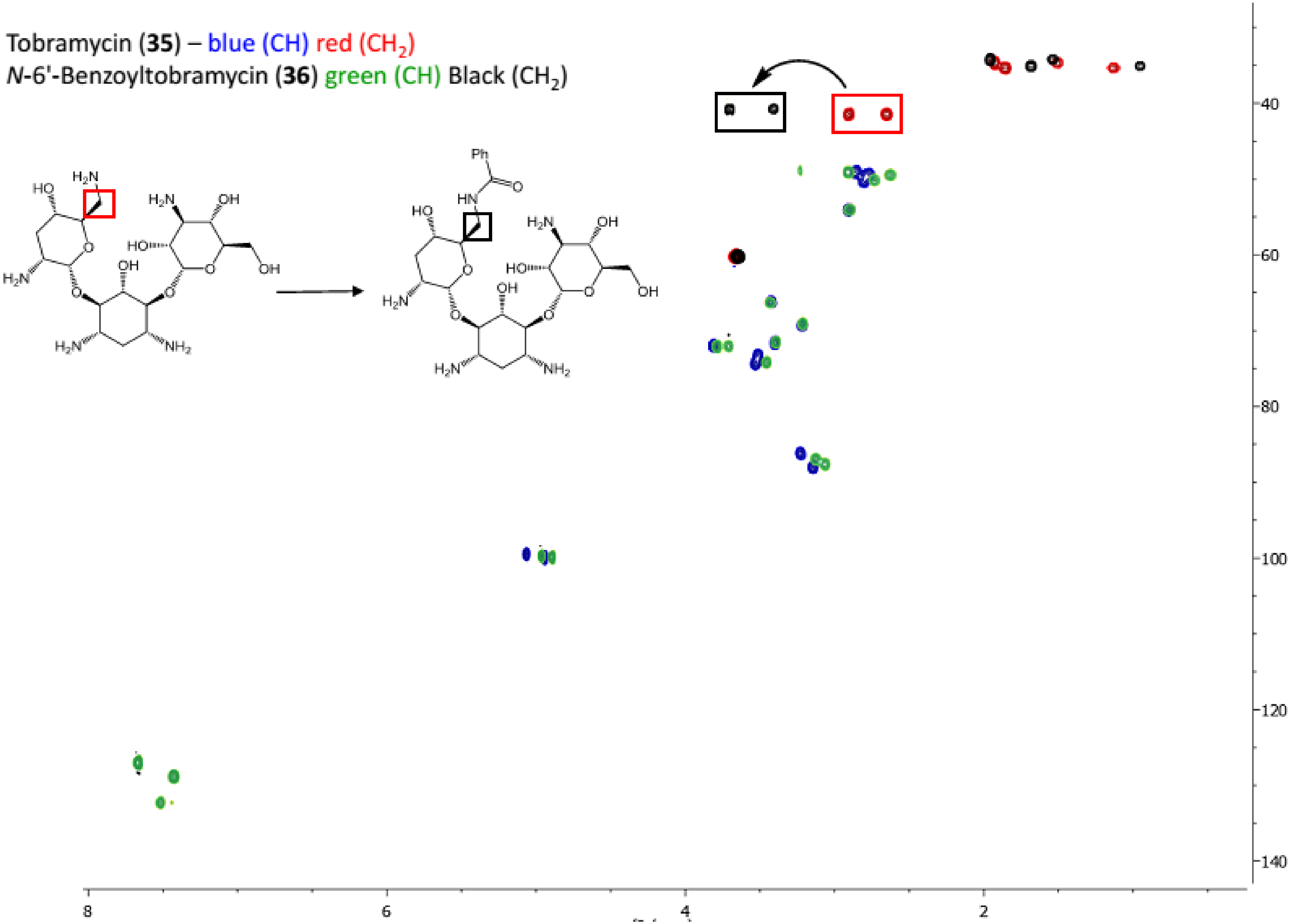

Overlay of the HSQC spectra of Tobramycin (**35**) (CH blue, CH_2 red) and *N*-6'-Benzoyltobramycin (**36**) (CH green, CH_2 black). The methylene in the red box are the only peaks to shift down-field due to the anisotropy of the carbonyl of the benzoyl group.

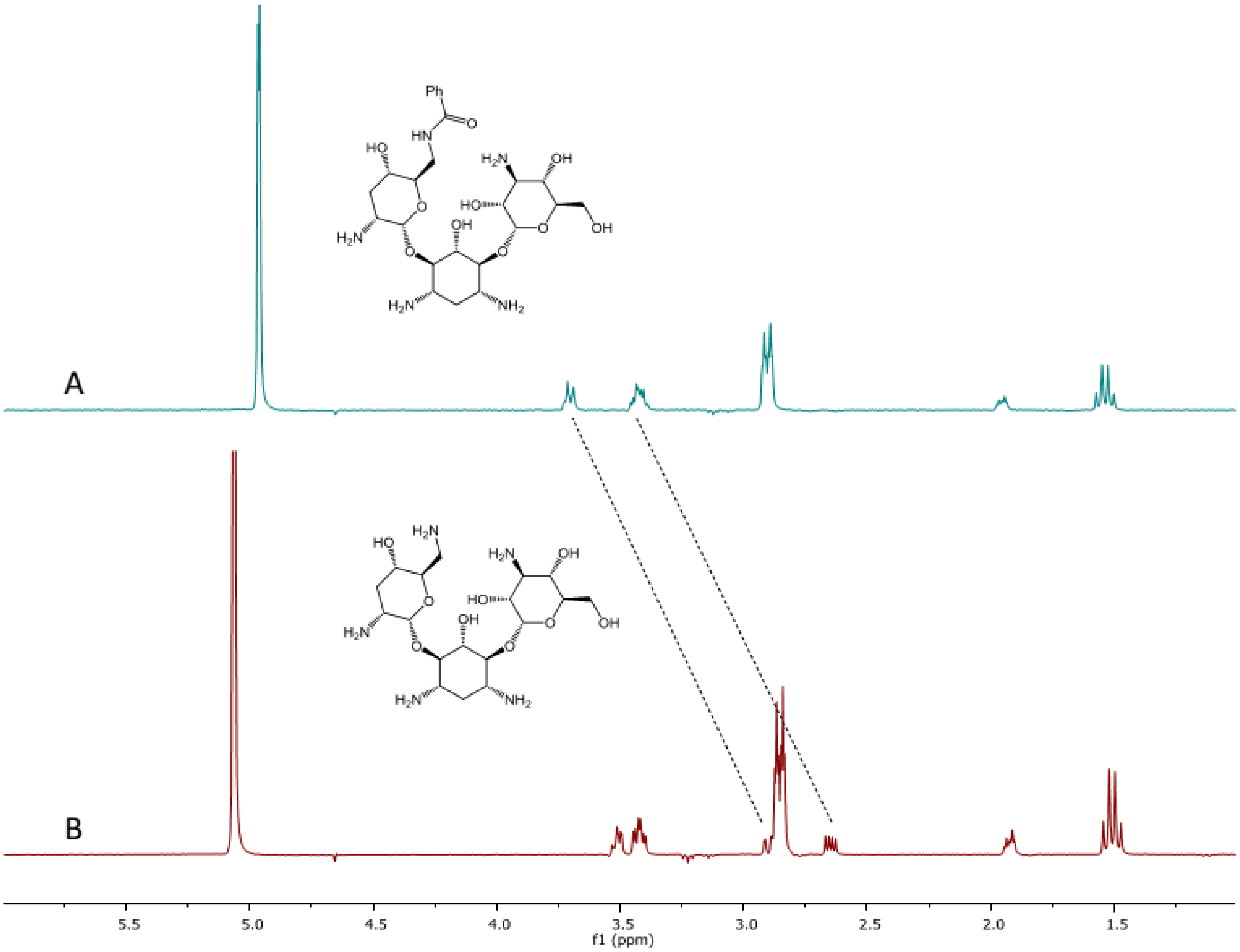

Selective 1D TOCSY spectra of (A) *N*-6'-Benzoyltobramycin (**36**) and (B) Tobramycin (**35**). Dotted lines show the large downfield shift of the benzoylated methylene.

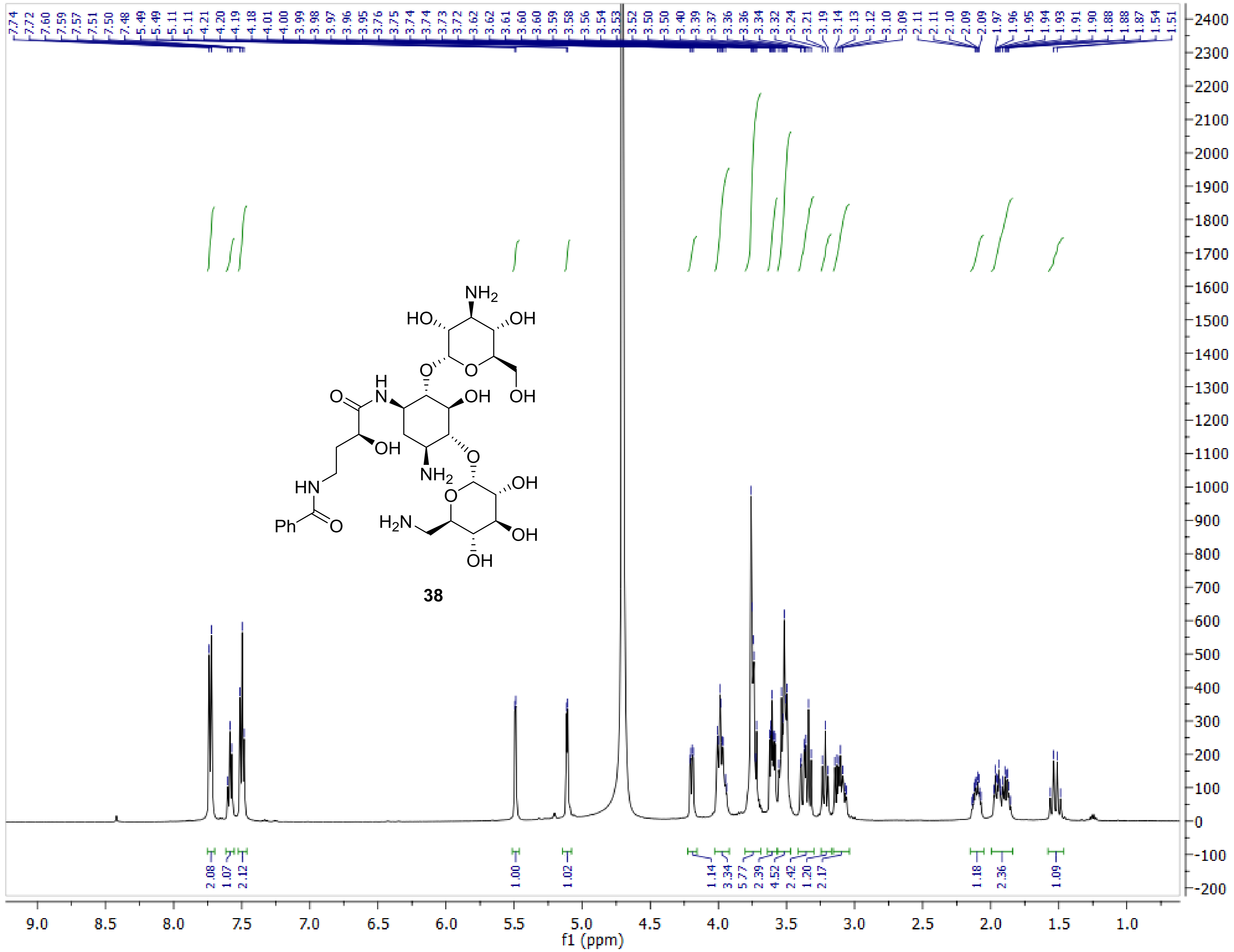

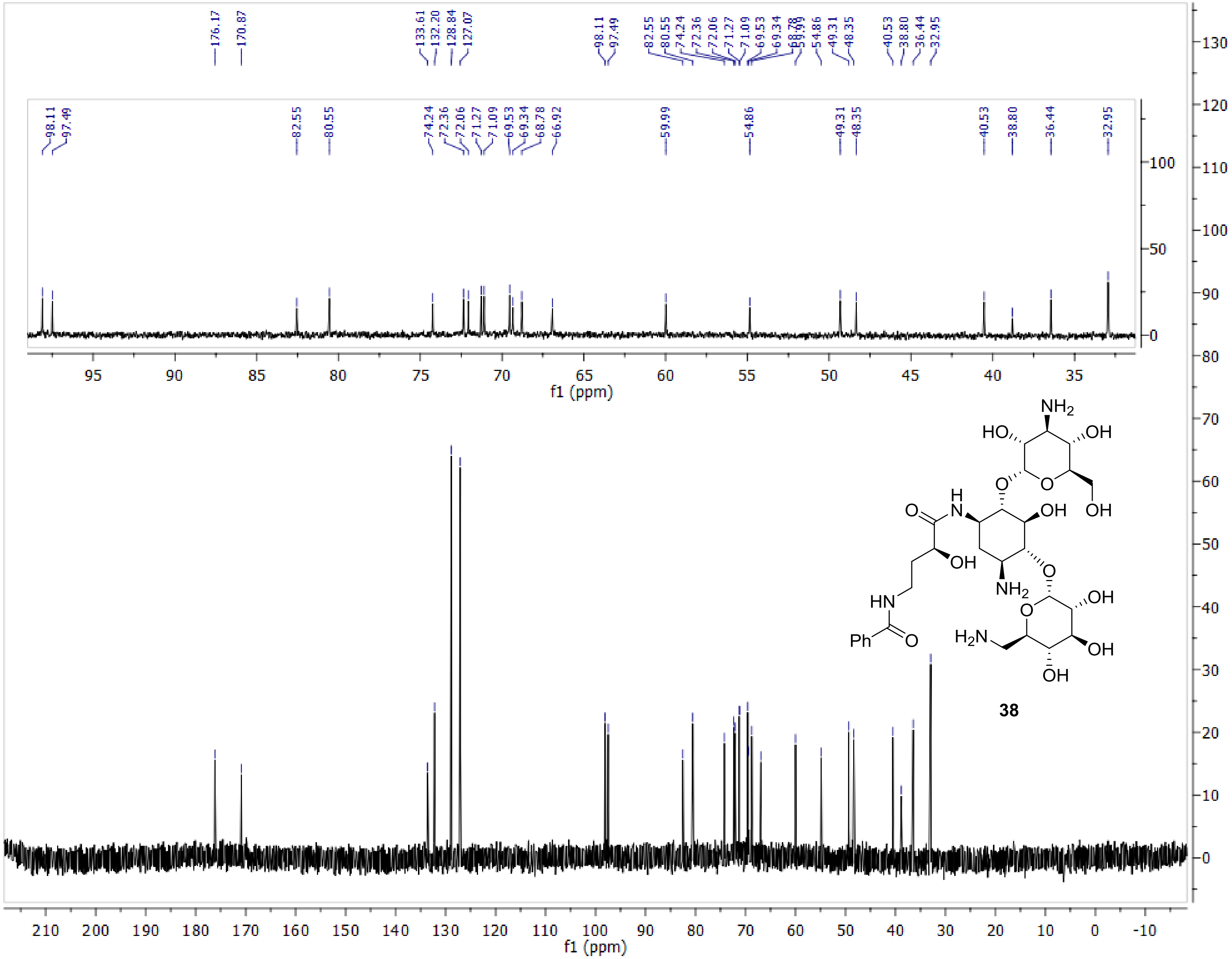

176.17
170.87
133.61
132.20
128.84
127.07
98.11
97.49
82.55
80.55
74.24
72.36
72.06
71.27
71.09
69.53
69.34
68.78
66.92
59.99
54.86
49.31
48.35
40.53
38.80
36.44
32.95
f1 (ppm)
f1 (ppm)
NH_2
HO
OH
O
O
OH
O
H
N
OH
OH
NH_2
O
OH
HN
O
H_2N
OH
Ph
O
OH
OH
38

Amikacin (**37**) – blue (CH) red (CH_2)

N-AHB-Benzoylamikacin (**38**) green (CH) Black (CH_2)

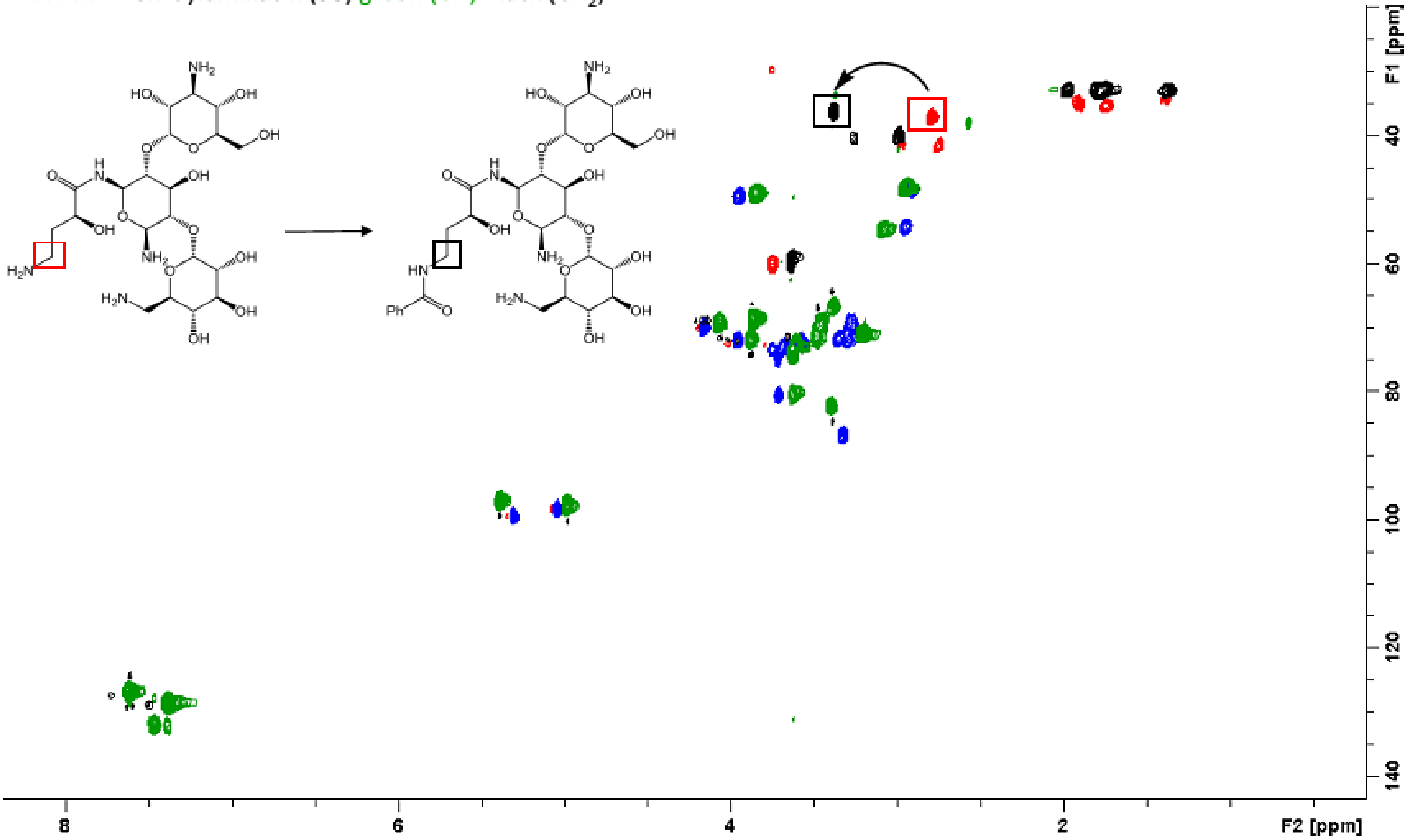

Overlay of the HSQC spectra of Amikacin (**37**) (CH blue, CH_2 red) and *N*-AHB-Benzoylamikacin (**38**) (CH green, CH_2 black). The methylene in the red box are the only peaks to shift down-field due to the anisotropy of the carbonyl of the benzoyl group.

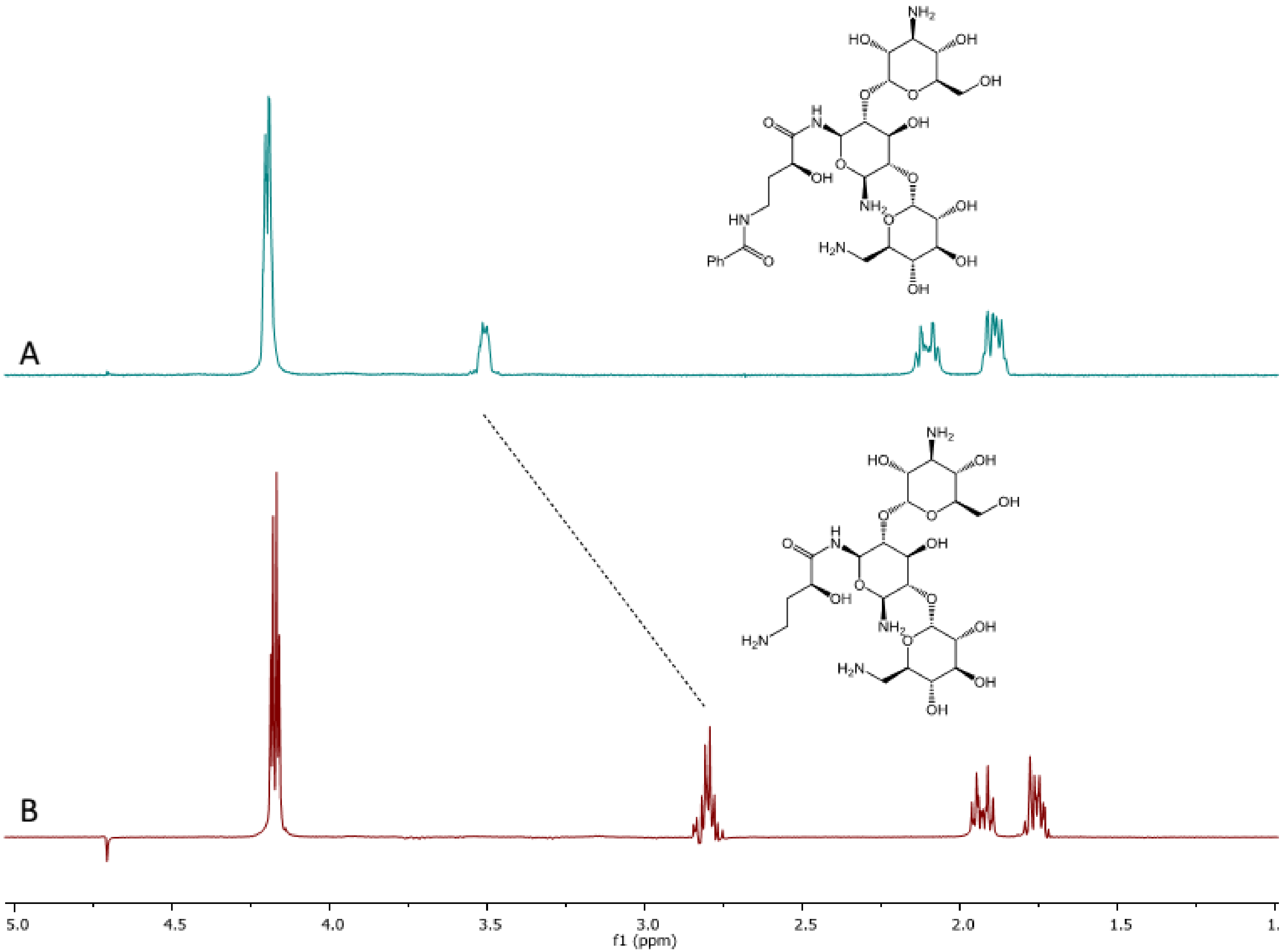

Selective 1D TOCSY spectra of (A) *N*-AHB-Benzoylamikacin (**38**) and (B) Amikacin (**37**). Dotted lines show the large downfield shift of the benzoylated methylene.

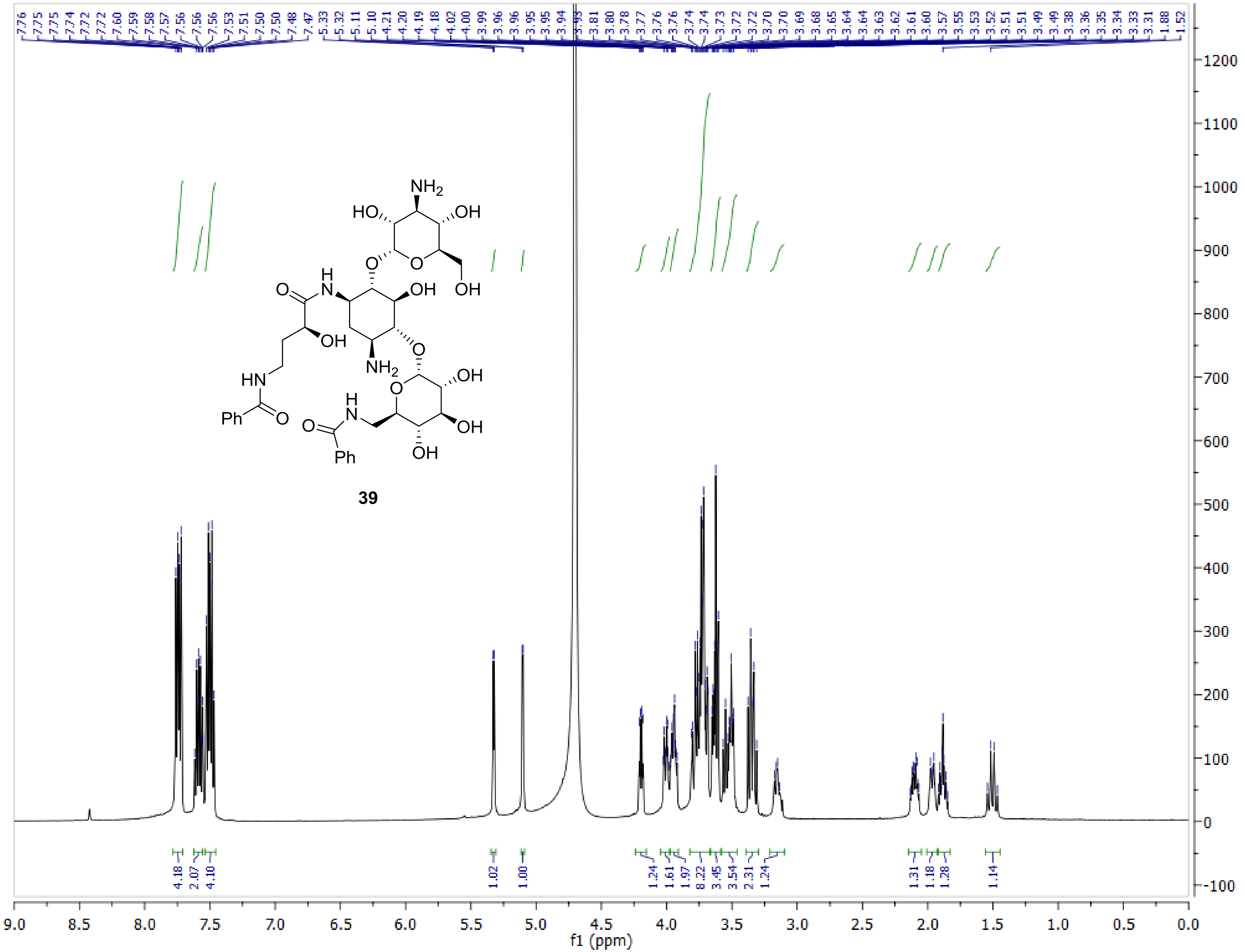
39
f1 (ppm)

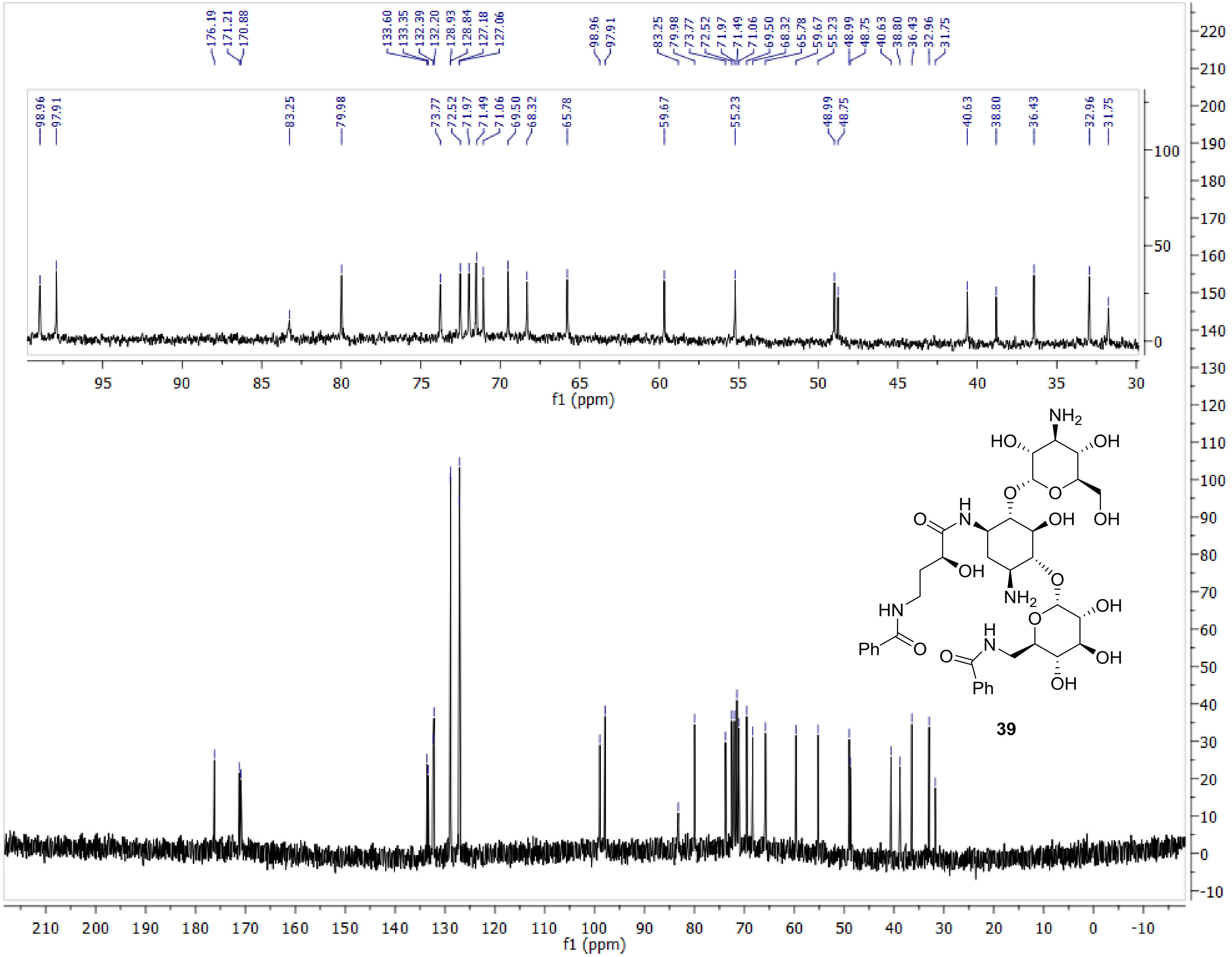
176.19
171.21
170.88
133.60
133.35
132.39
132.20
128.93
128.84
127.18
127.06
98.96
97.91
83.25
79.98
73.77
72.52
71.97
71.49
71.06
69.50
68.32
65.78
59.67
55.23
48.99
48.75
40.63
38.80
36.43
32.96
31.75
f1 (ppm)
NH2
HO
OH
O
H
N
OH
OH
OH
NH2
HN
Ph
O
O
H
N
O
Ph
OH
OH
OH
39

Amikacin (**37**) – blue (CH) red (CH_2)
N-6'-*N*-AHB-Dibenzoylamikacin (**39**) green (CH) Black (CH_2)

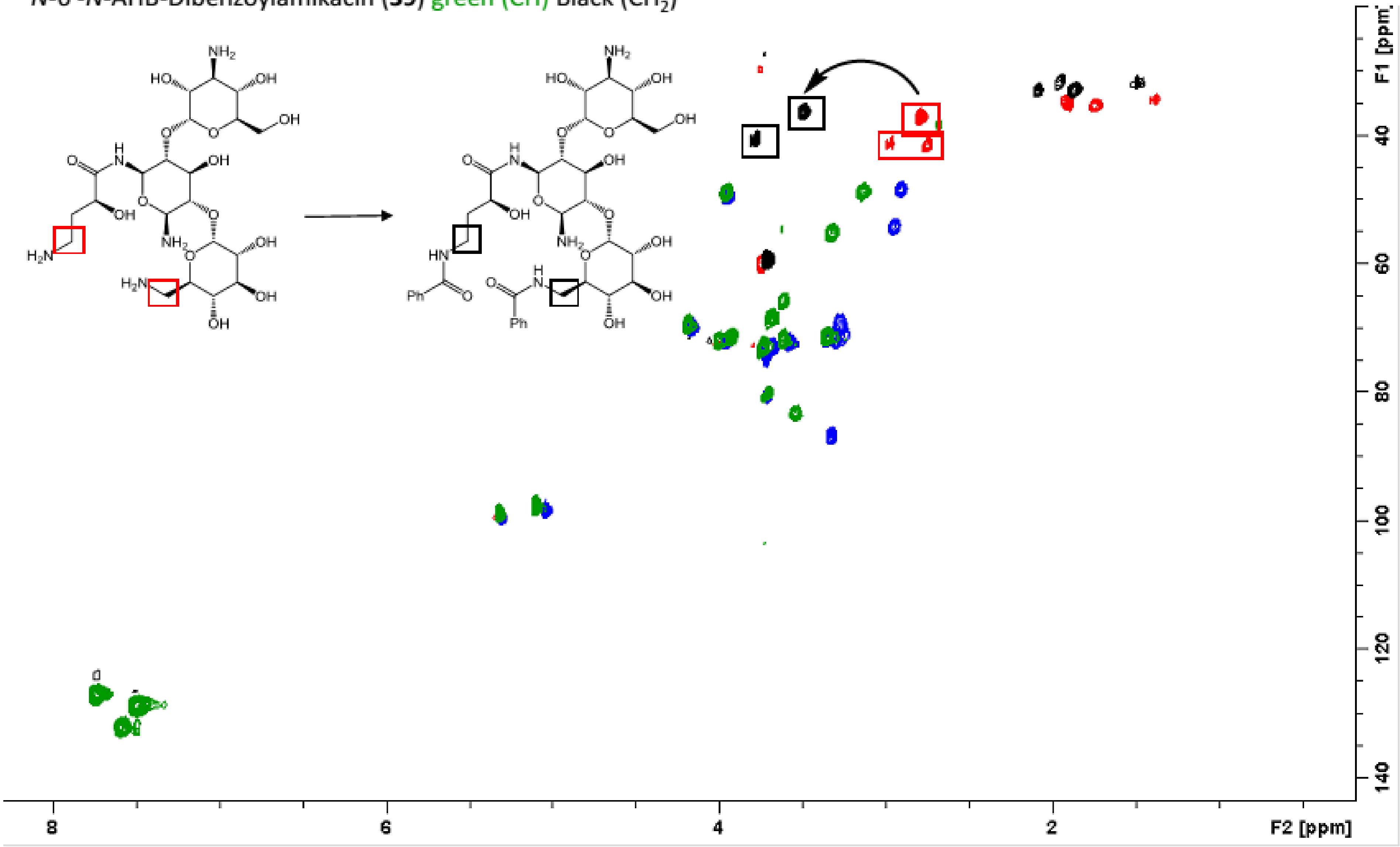

Overlay of the HSQC spectra of Amikacin (**37**) (CH blue, CH_2 red) and *N*-6'-*N*-AHB-Dibenzoylamikacin (**39**) (CH green, CH_2 black). The methylenes in the red box are the only peaks to shift down-field due to the anisotropy of the carbonyl of the benzoyl group.

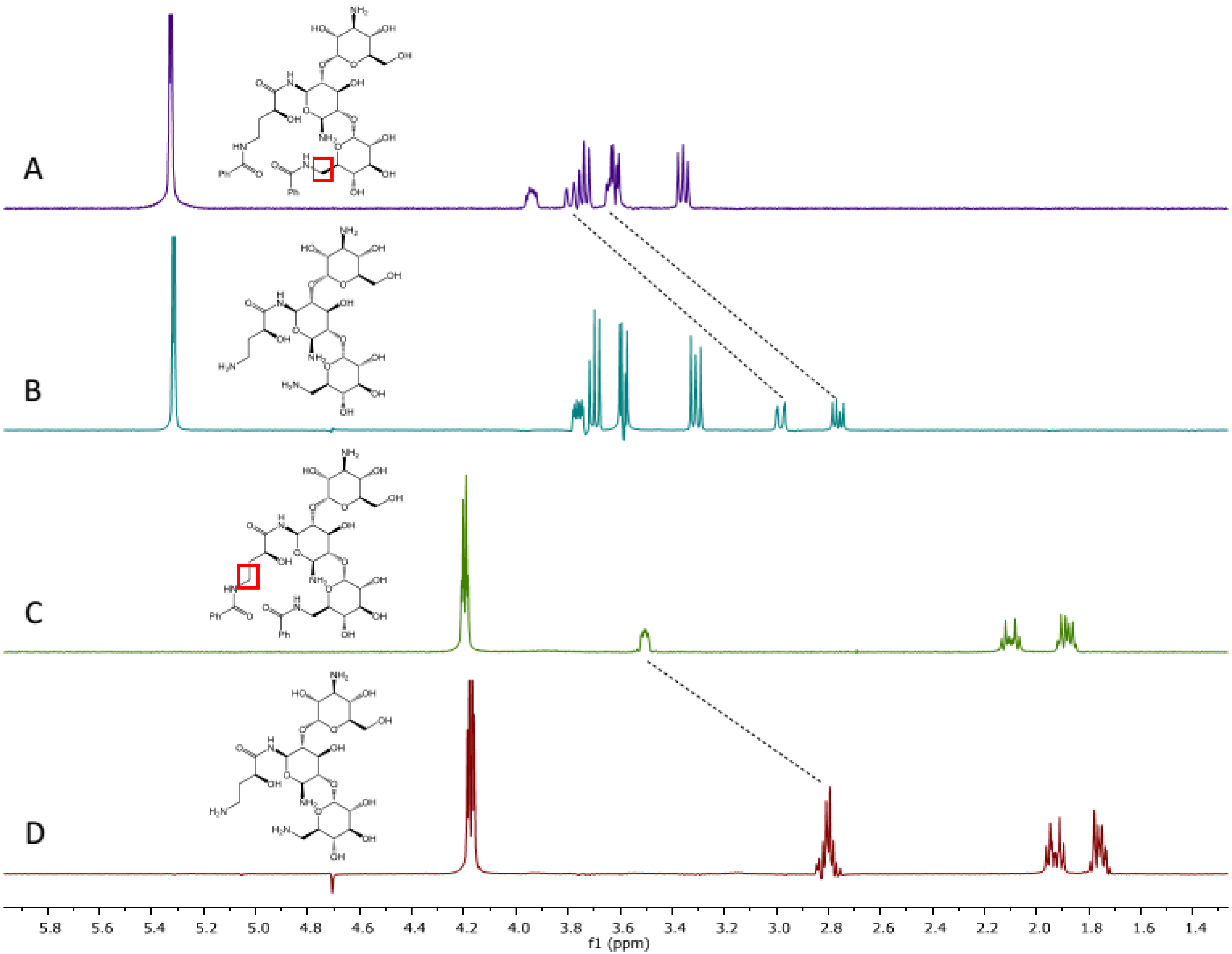

Selective 1D TOCSY spectra of (A and C) *N*-6'-*N*-AHB-Dibenzoylamikacin (**39**) and (B and D) Amikacin (**37**). Dotted lines show the large downfield shift of the benzoylated methylenes.

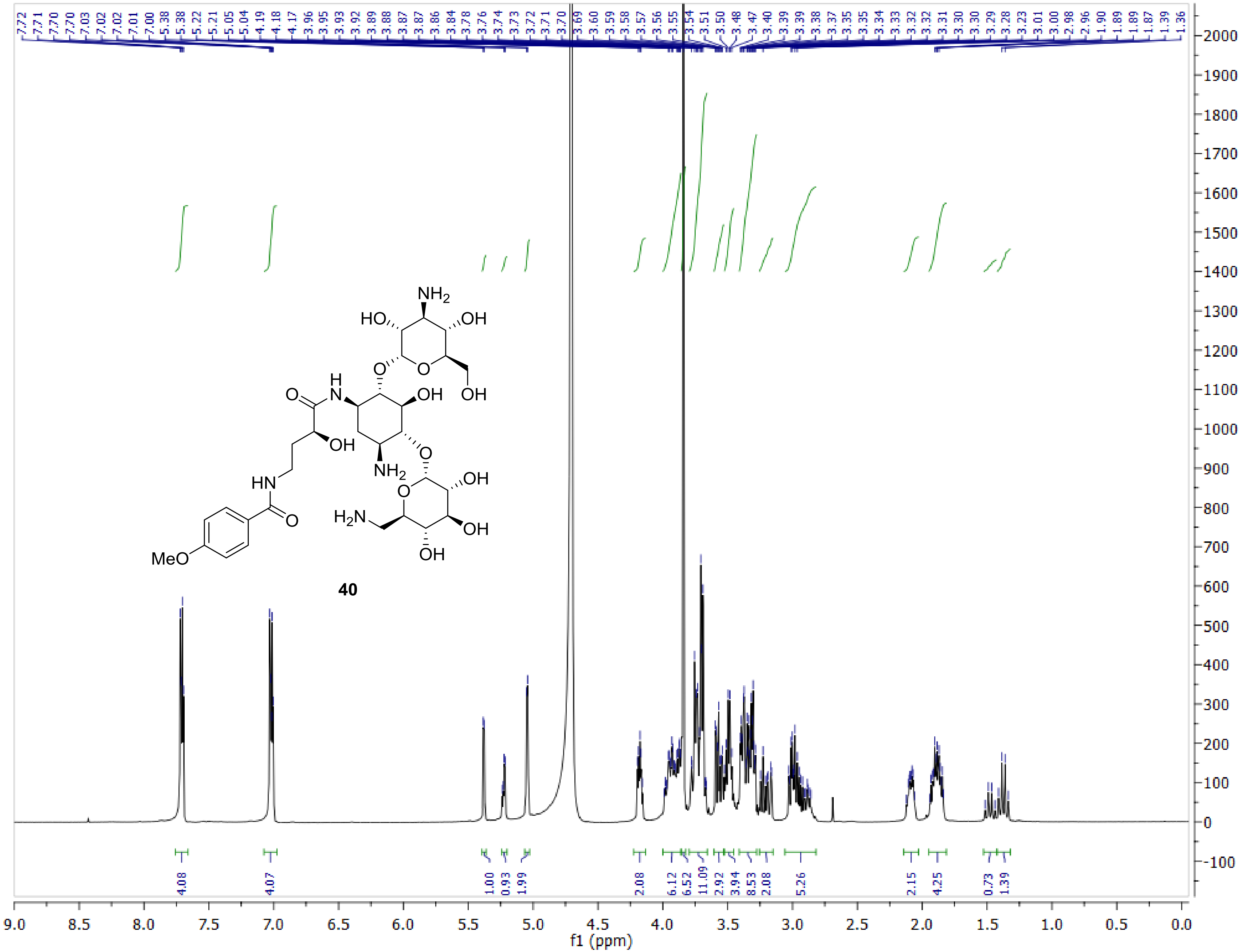

40

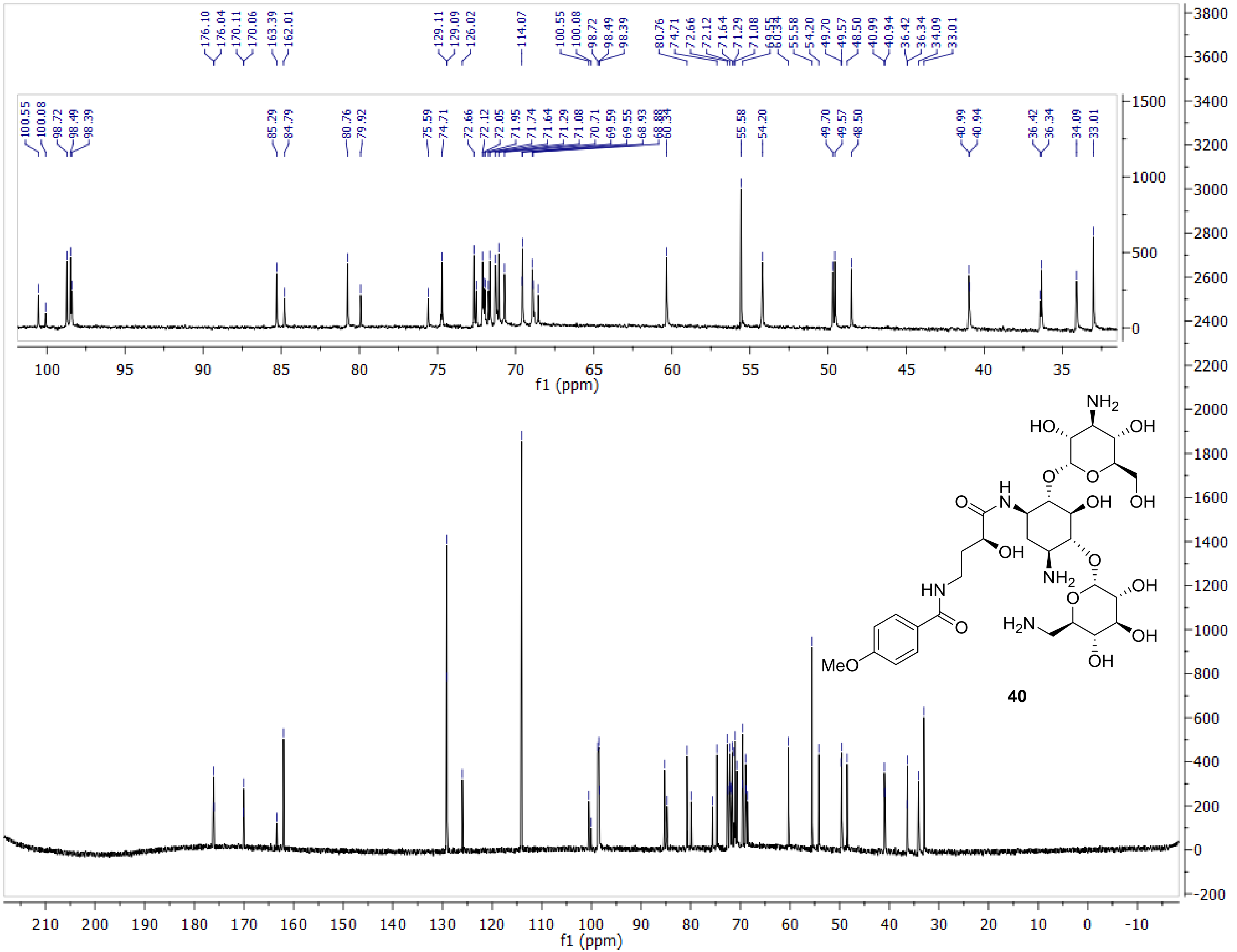
176.10
176.04
170.11
170.06
163.39
162.01
129.11
129.09
126.02
114.07
100.55
100.08
98.72
98.49
98.39
80.76
74.71
72.66
72.12
71.64
71.29
71.08
69.55
60.34
55.58
54.20
49.70
49.57
48.50
40.99
40.94
36.42
36.34
34.09
33.01
100.55
100.08
98.72
98.49
98.39
85.29
84.79
80.76
79.92
75.59
74.71
72.66
72.12
72.05
71.95
71.74
71.64
71.29
71.08
70.71
69.59
69.55
68.93
68.88
60.34
55.58
54.20
49.70
49.57
48.50
40.99
40.94
36.42
36.34
34.09
33.01
f1 (ppm)
f1 (ppm)
NH_2
HO
OH
O
O
H
N
OH
OH
O
OH
NH_2
HN
O
OH
O
H_2N
OH
O
OH
MeO
40

Amikacin (**37**) – blue (CH) red (CH_2)
N-AHB-(4-Methoxybenzoyl)amikacin (**40**) green (CH) Black (CH_2)

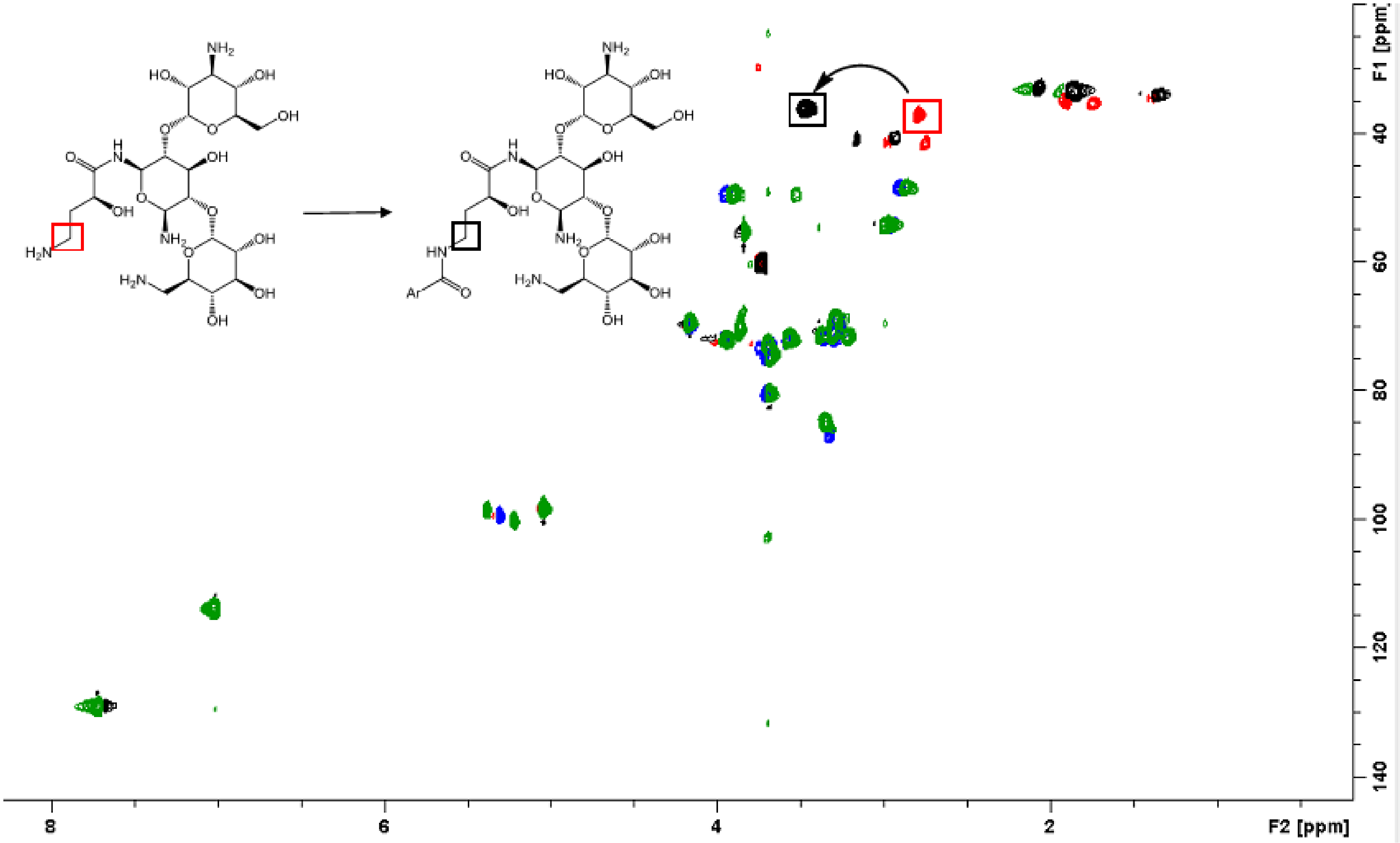

Overlay of the HSQC spectra of Amikacin (**37**) (CH blue, CH_2 red) and *N*-AHB-(4-Methoxybenzoyl)amikacin (**40**) (CH green, CH_2 black). The methylene in the red box are the only peaks to shift down-field due to the anisotropy of the carbonyl of the benzoyl group.

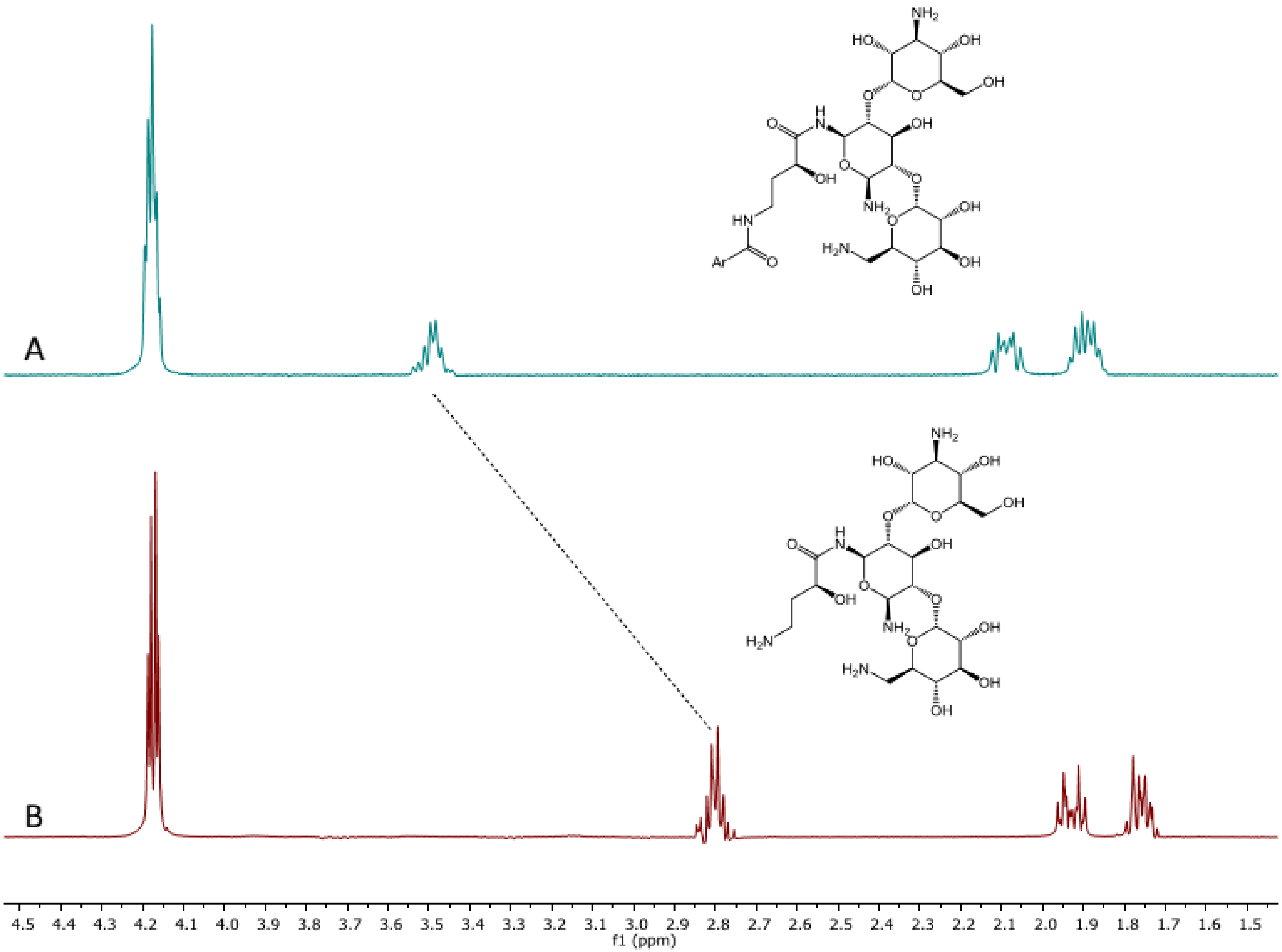

Selective 1D TOCSY spectra of (A) *N*-AHB-(4-Methoxybenzoyl)amikacin (**40**) and (B) Amikacin (**37**). Dotted lines show the large downfield shift of the 4-methoxybenzoylated methylene.

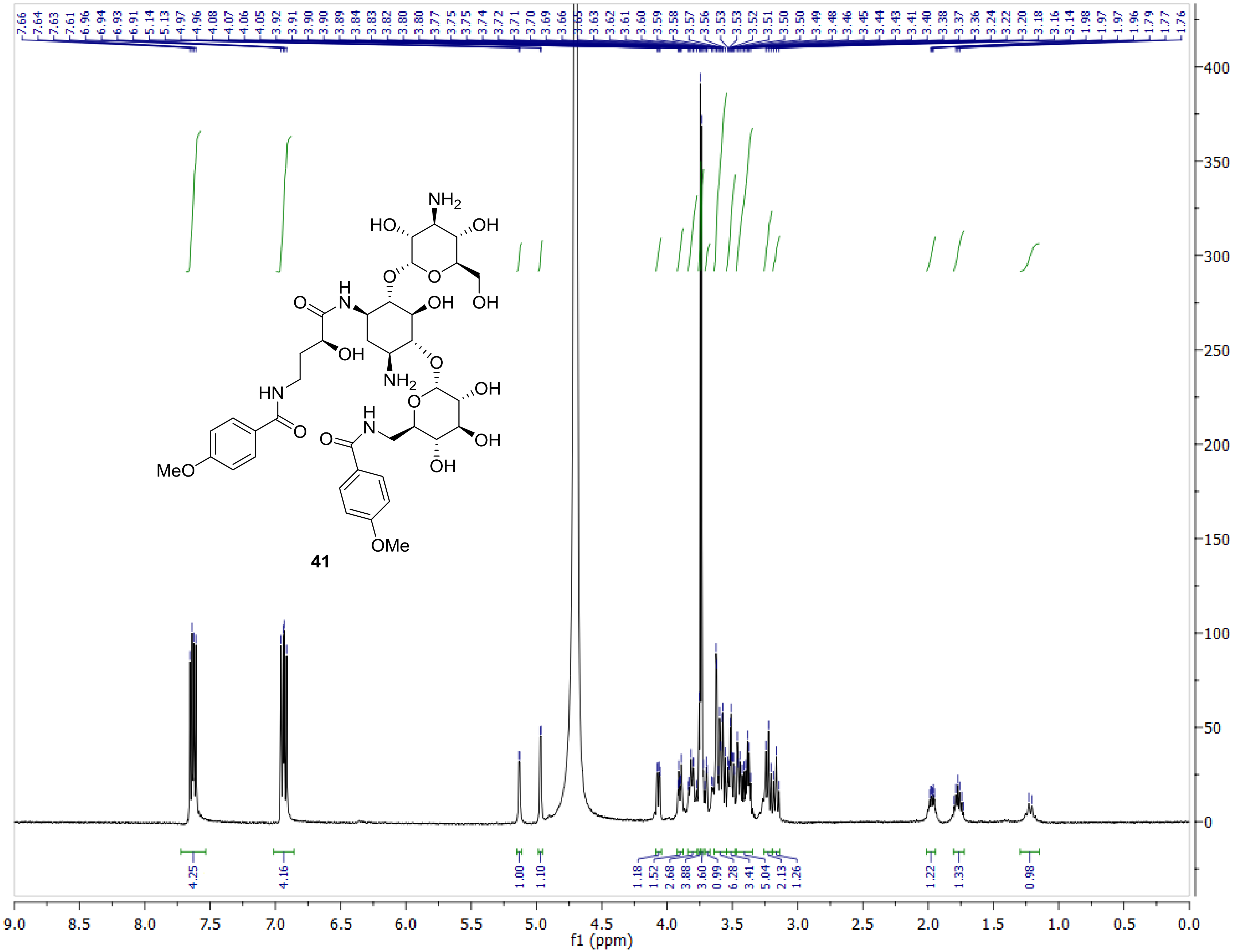

NH2
HO
OH
O
O
H
N
OH
OH
O
OH
NH2
O
HN
O
OH
H
N
O
OH
OH
MeO
OMe
41
f1 (ppm)

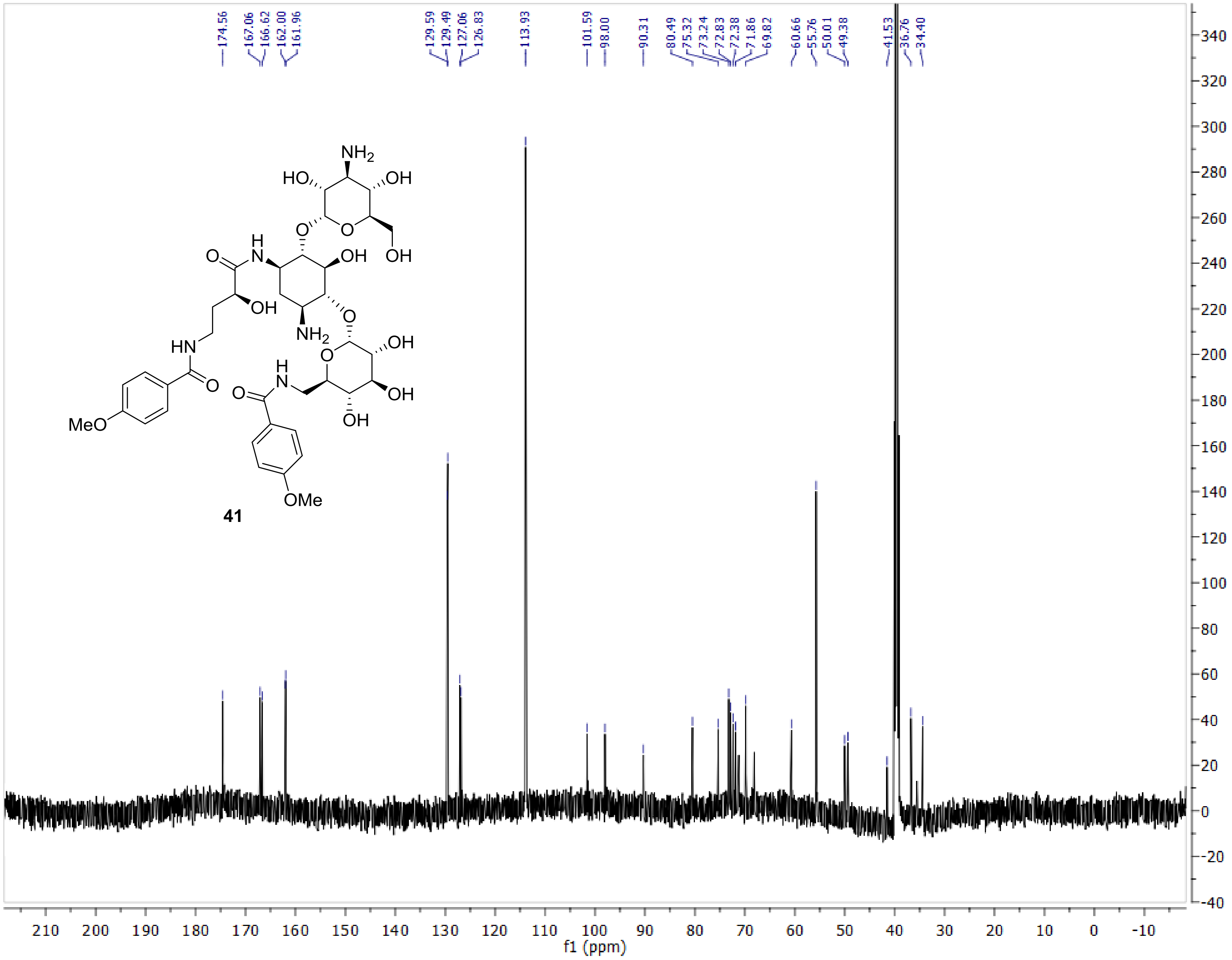
174.56
167.06
166.62
162.00
161.96
129.59
129.49
127.06
126.83
113.93
101.59
98.00
90.31
80.49
75.32
73.24
72.83
72.38
71.86
69.82
60.66
55.76
50.01
49.38
41.53
36.76
34.40
NH2
HO
OH
O
H
N
O
OH
OH
OH
NH2
O
OH
HN
O
H
N
O
O
OH
OH
MeO
OMe
41
f1 (ppm)

Amikacin (**37**) – blue (CH) red (CH_2)

N-6'-*N*-AHB-Bis(4-methoxybenzoyl)amikacin (**41**) green (CH) Black (CH_2)

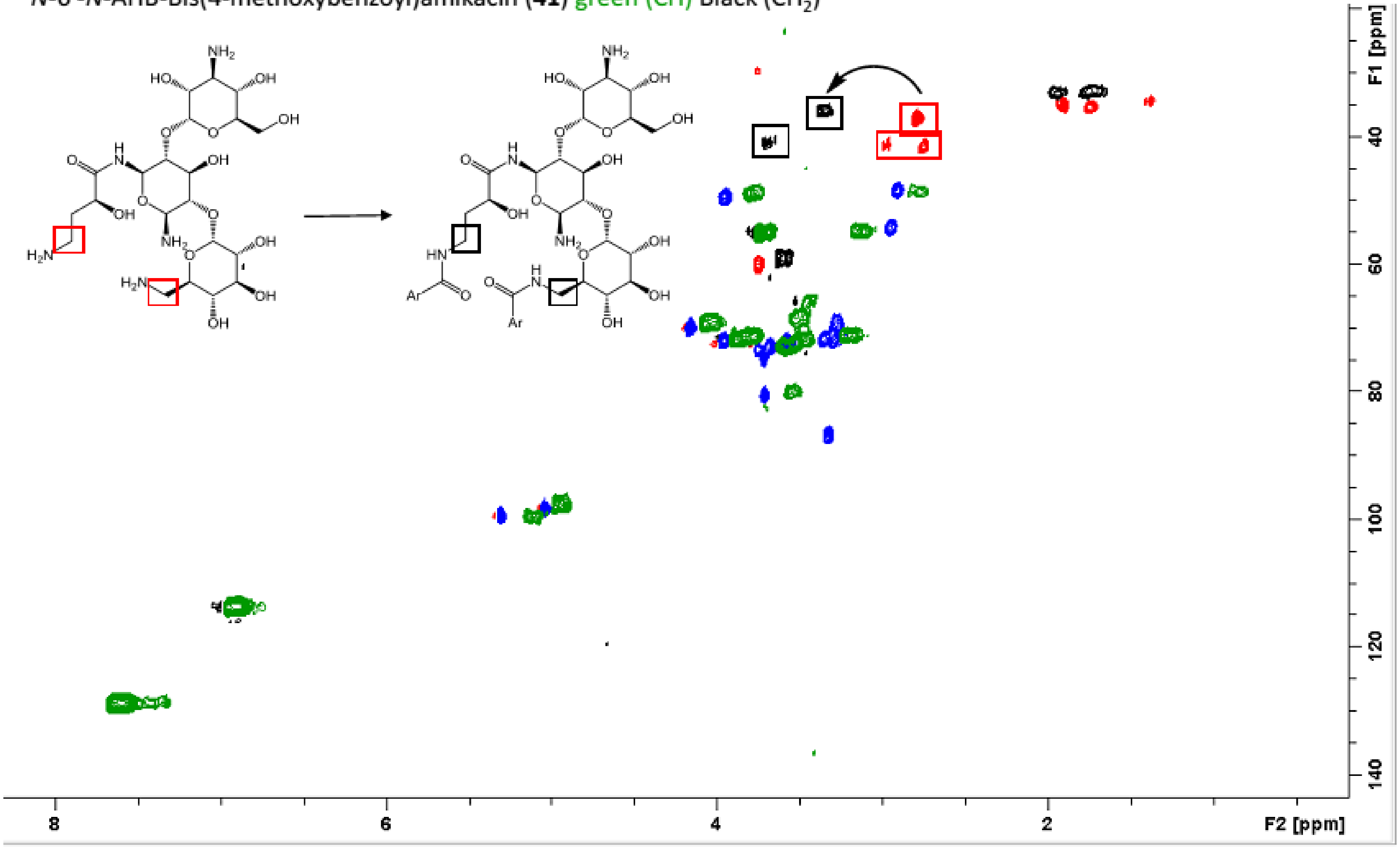

Overlay of the HSQC spectra of Amikacin (**37**) (CH blue, CH_2 red) and *N*-6'-*N*-AHB-Bis(4-methoxybenzoyl)amikacin (**41**) (CH green, CH_2 black). The methylenes in the red box are the only peaks to shift down-field due to the anisotropy of the carbonyl of the benzoyl group.

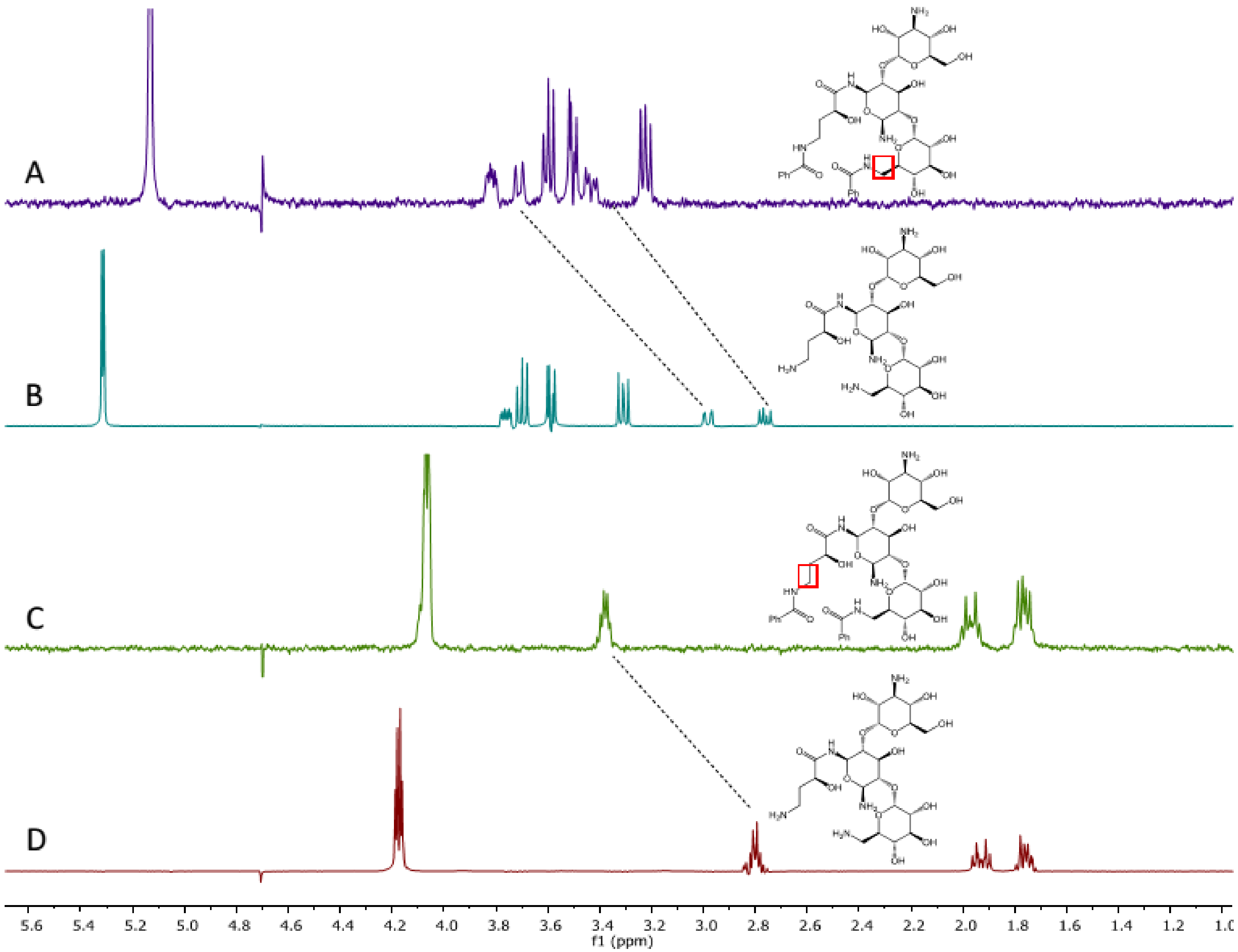

Selective 1D TOCSY spectra of (A and C) *N*-6'-*N*-AHB-Bis(4-methoxybenzoyl)amikacin (**41**) and (B and D) Amikacin (**37**). Dotted lines show the large downfield shift of the 4-methoxy benzoylated methylenes.

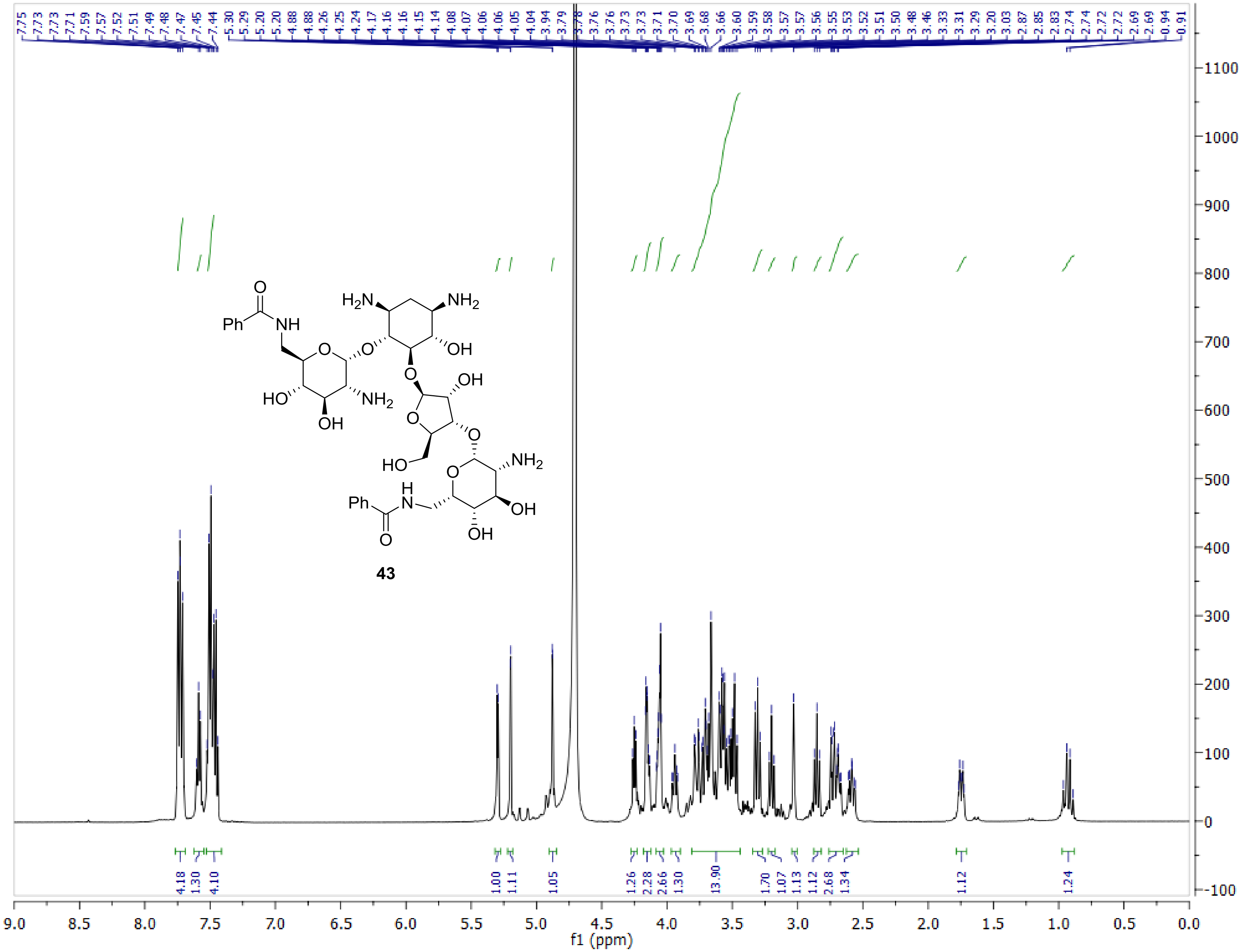
7.75 7.73 7.73 7.71 7.59 7.57 7.52 7.51 7.49 7.48 7.47 7.45 7.44 5.30 5.29 5.20 5.20 4.88 4.88 4.26 4.25 4.24 4.17 4.16 4.16 4.15 4.14 4.08 4.07 4.06 4.06 4.05 4.04 3.94 3.79 3.78 3.76 3.76 3.73 3.73 3.71 3.70 3.69 3.68 3.66 3.60 3.59 3.58 3.57 3.57 3.56 3.55 3.53 3.52 3.51 3.50 3.48 3.46 3.33 3.31 3.29 3.20 3.03 2.87 2.85 2.83 2.74 2.74 2.72 2.72 2.69 2.69 0.94 0.91
4.18 1.30 4.10 1.00 1.11 1.05 1.26 2.28 2.66 1.30 13.90 1.70 1.07 1.13 1.12 2.68 1.34 1.12 1.24
f1 (ppm)
43

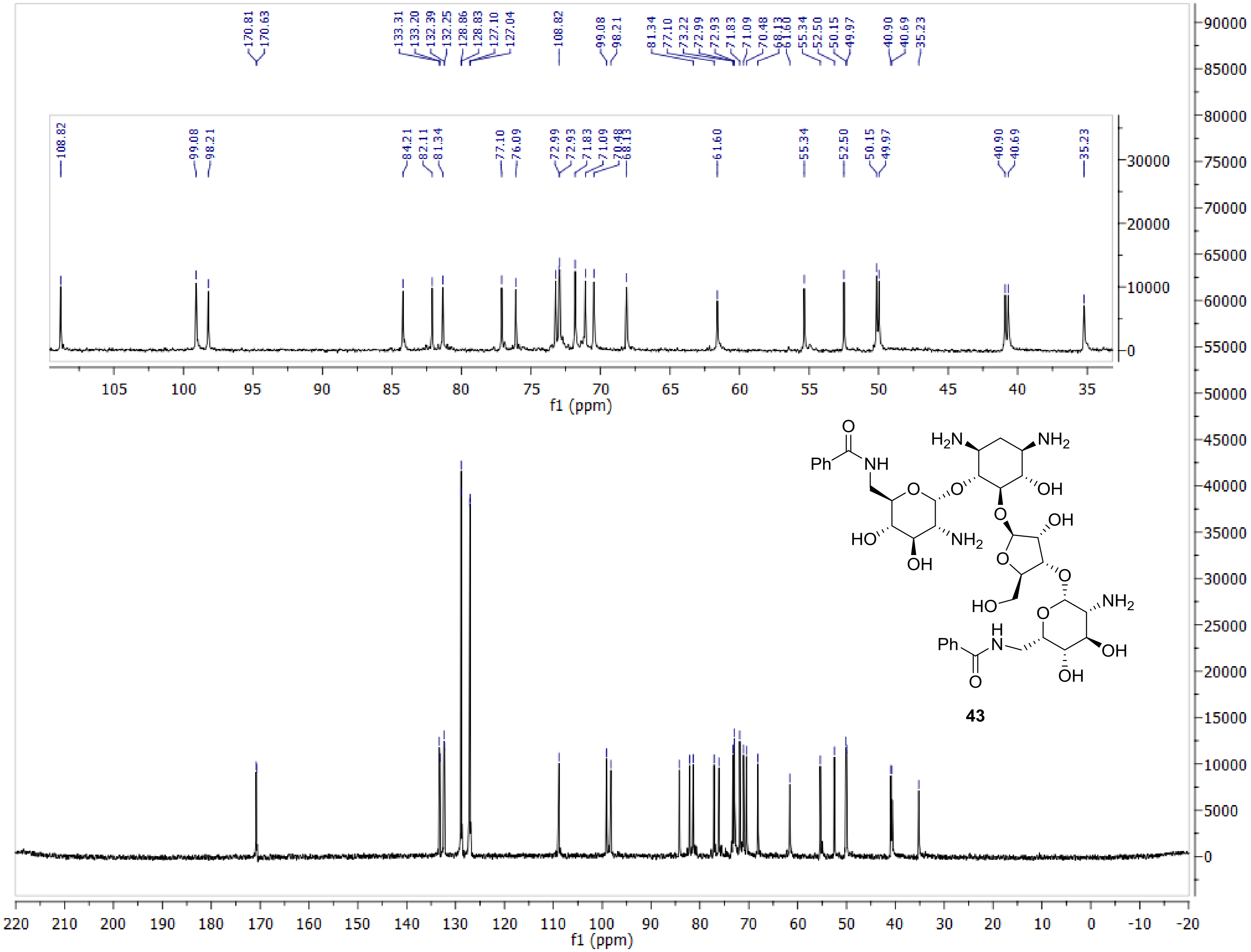
170.81
170.63
133.31
133.20
132.39
132.25
128.86
128.83
127.10
127.04
108.82
99.08
98.21
81.34
77.10
73.22
72.99
72.93
71.83
71.09
70.48
68.13
61.60
55.34
52.50
50.15
49.97
40.90
40.69
35.23
108.82
99.08
98.21
84.21
82.11
81.34
77.10
76.09
72.99
72.93
71.83
71.09
70.48
68.13
61.60
55.34
52.50
50.15
49.97
40.90
40.69
35.23
f1 (ppm)
f1 (ppm)
Ph
NH
O
H2N
NH2
OH
HO
NH2
OH
OH
HO
NH2
OH
OH
43

Neomycin B (**42**) – blue (CH) red (CH_2)
N-6'-*N*-6'''-Dibenzoylneomycin B (**43**) green (CH) Black (CH_2)

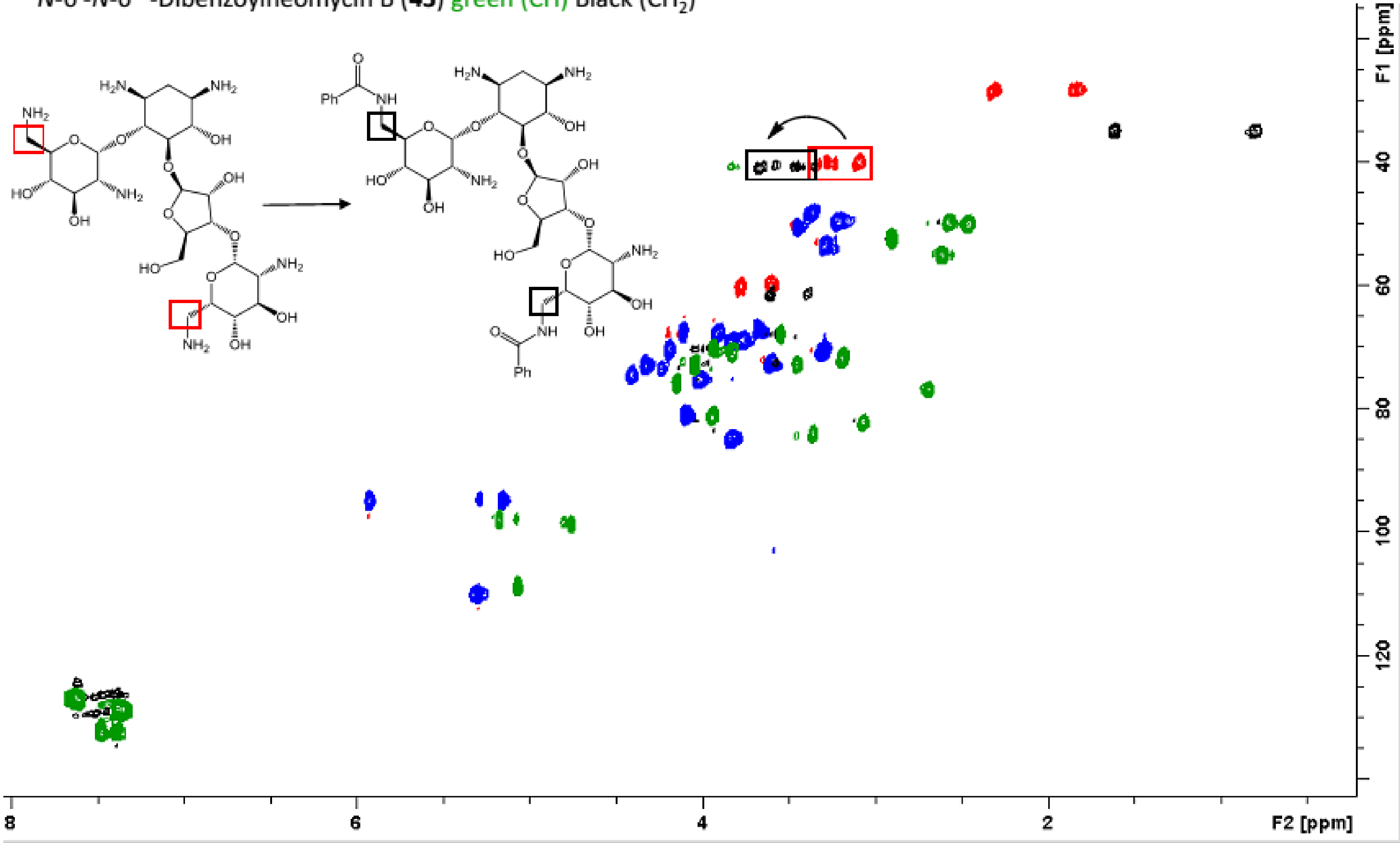

Overlay of the HSQC spectra of Neomycin B (**42**) (CH blue, CH_2 red) and *N*-6'-*N*-6'''-Dibenzoylneomycin B (**43**) (CH green, CH_2 black). The methylenes in the red box are the only peaks to shift down-field due to the anisotropy of the carbonyl of the benzoyl group.

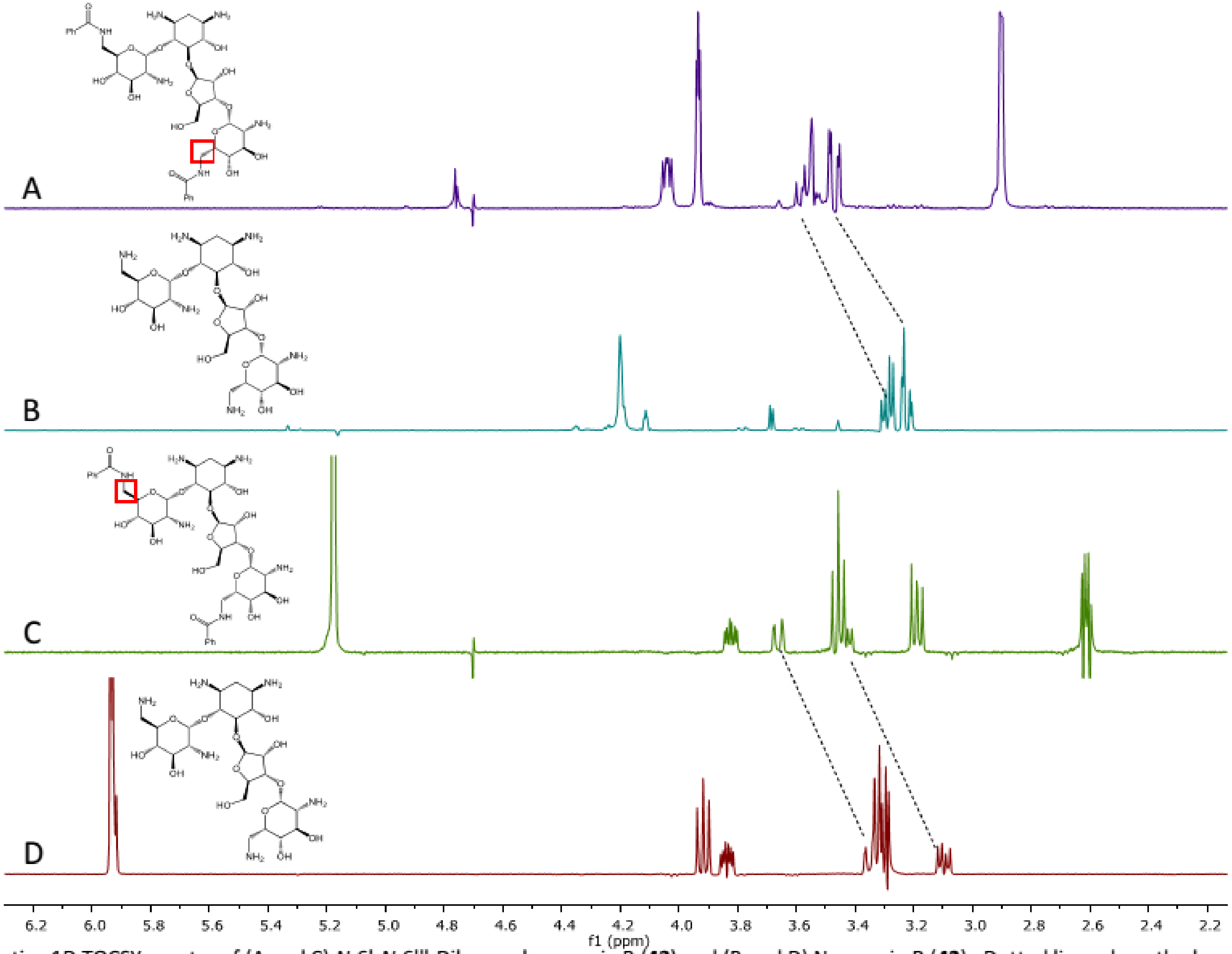

Selective 1D TOCSY spectra of (A and C) *N*-6'-*N*-6'''-Dibenzoylneomycin B (**43**) and (B and D) Neomycin B (**42**). Dotted lines show the large downfield shift of the benzoylated methylenes.

HPLC Traces

<u>Mixture of standards</u>: Phenomenex silica-based column (Lichrosorb 5 Sil 60A 250×4.60 mm), flow rate 1.5 mL/min, hexanes/*i*-PrOH = 85:15

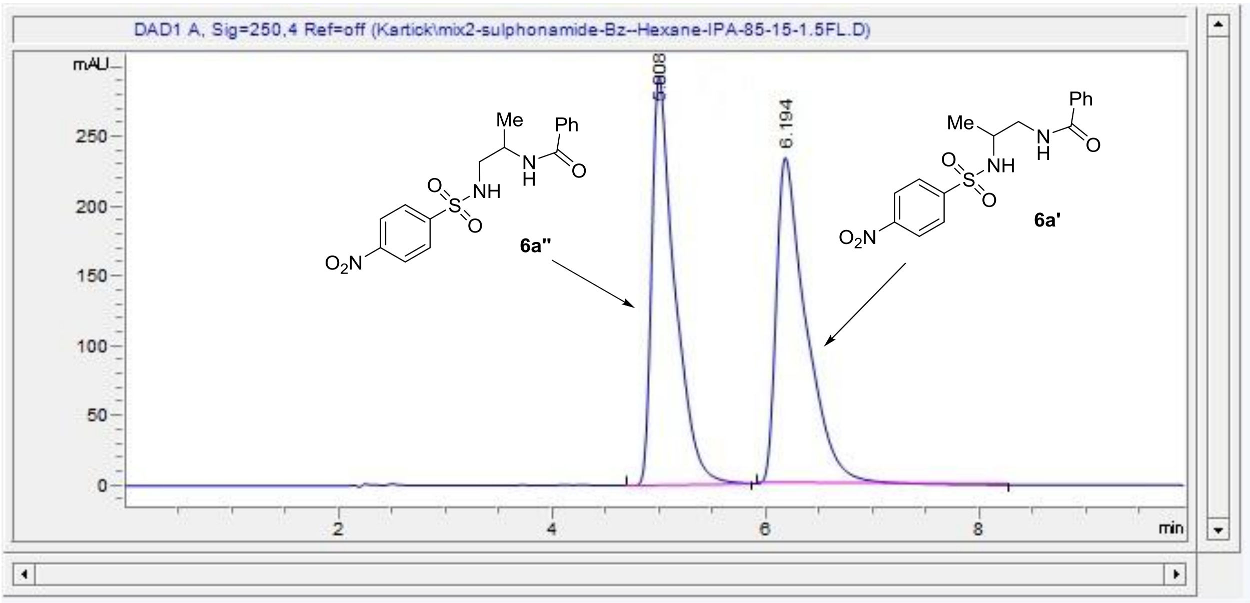

#	Time	Area	Height	Width	Area%	Symmetry
1	5.008	4468.3	295	0.2524	49.243	0.456
2	6.194	4605.7	234.5	0.2812	50.757	0.402

<u>Site-selective reaction with BCPP **1a**</u>: Phenomenex silica-based column (Lichrosorb 5 Sil 60A 250×4.60 mm), flow rate 1.5 mL/min, hexanes/*i*-PrOH = 85:15

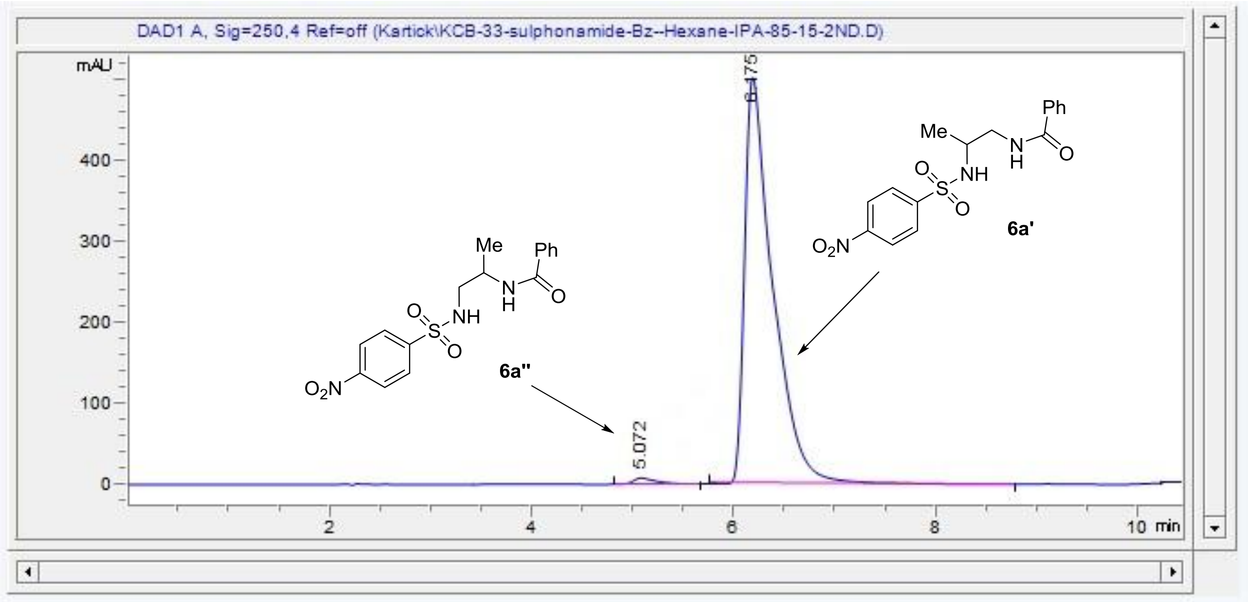

#	Time	Area	Height	Width	Area%	Symmetry
1	5.072	157.4	8.4	0.3113	1.575	0.524
2	6.175	9838.5	504.6	0.2755	98.425	0.374

<u>Mixture of standards</u>: Phenomenex silica-based column (Lichrosorb 5 Sil 60A 250×4.60 mm), flow rate 1.0 mL/min, hexanes/*i*-PrOH = 85:15

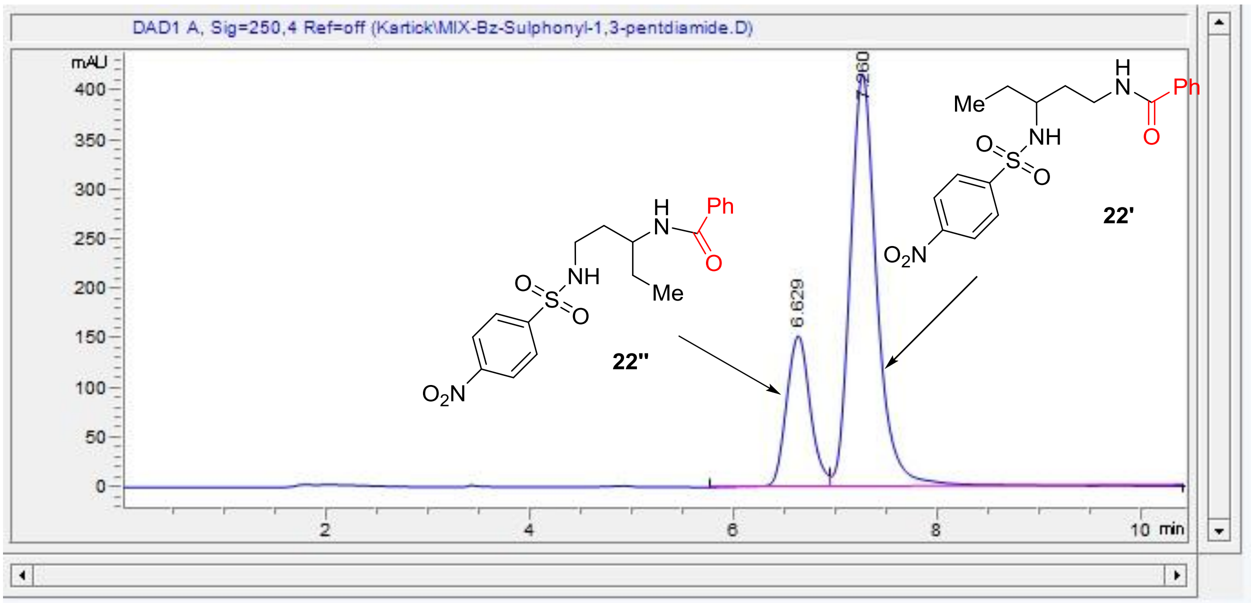

#	Time	Area	Height	Width	Area%	Symmetry
1	6.629	2482.9	152.5	0.2539	24.444	0.942
2	7.26	7674.5	416.6	0.2786	75.556	0.767

<u>Site-selective reaction with BCPP **1a**</u>: Phenomenex silica-based column (Lichrosorb 5 Sil 60A 250×4.60 mm), flow rate 1.0 mL/min, hexanes/*i*-PrOH = 85:15

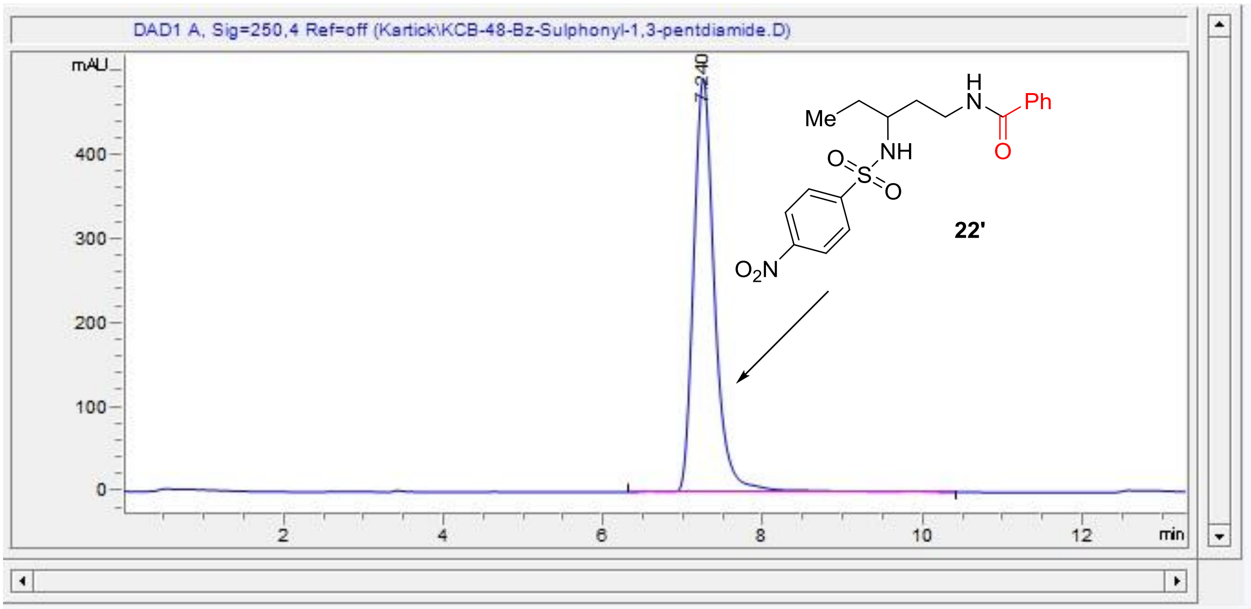

#	Time	Area	Height	Width	Area%	Symmetry
1	7.24	9090.4	495.4	0.2793	100.000	0.771

HPLC Traces of Aminoglycosides

Lichrospher® 100 NH_2 (5µm) C18 column, flow rate 0.5 mL/min, H_2O/CH_3CN = 70:30

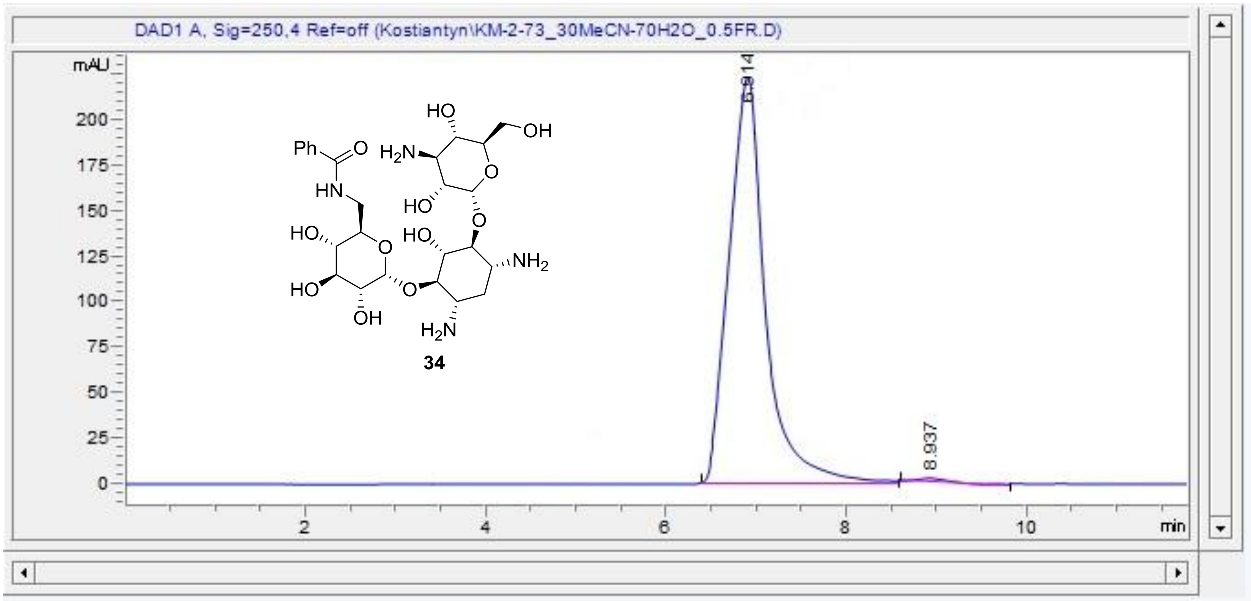

#	Time	Area	Height	Width	Area%	Symmetry
1	6.914	6207.4	220.1	0.4701	99.042	0.987
2	8.937	60	2.2	0.3837	0.958	0.498

Lichrospher® 100 NH_2 (5µm) C18 column, flow rate 0.5 mL/min, H_2O/CH_3CN = 70:30

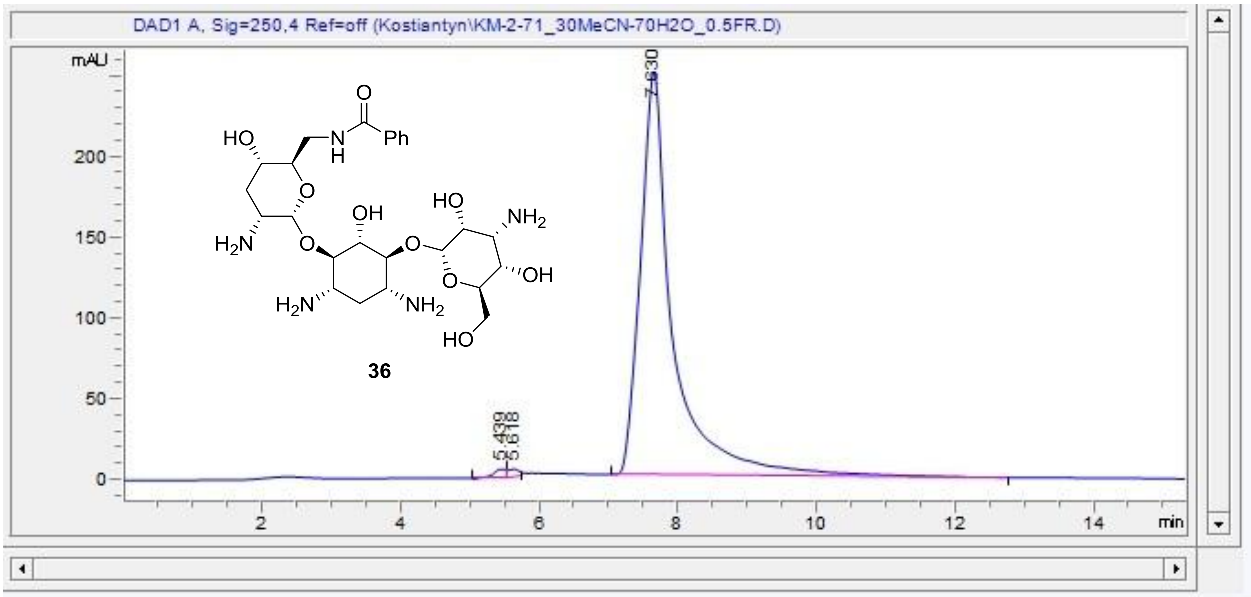

#	Time	Area	Height	Width	Area%	Symmetry
1	5.439	82.4	5.6	0.2212	0.994	1.857
2	5.618	59.5	5.4	0.1572	0.718	0.716
3	7.63	8154.3	249.9	0.4507	98.289	0.609

Lichrospher® 100 NH_2 (5µm) C18 column, flow rate 0.5 mL/min, H_2O/CH_3CN = 60:40

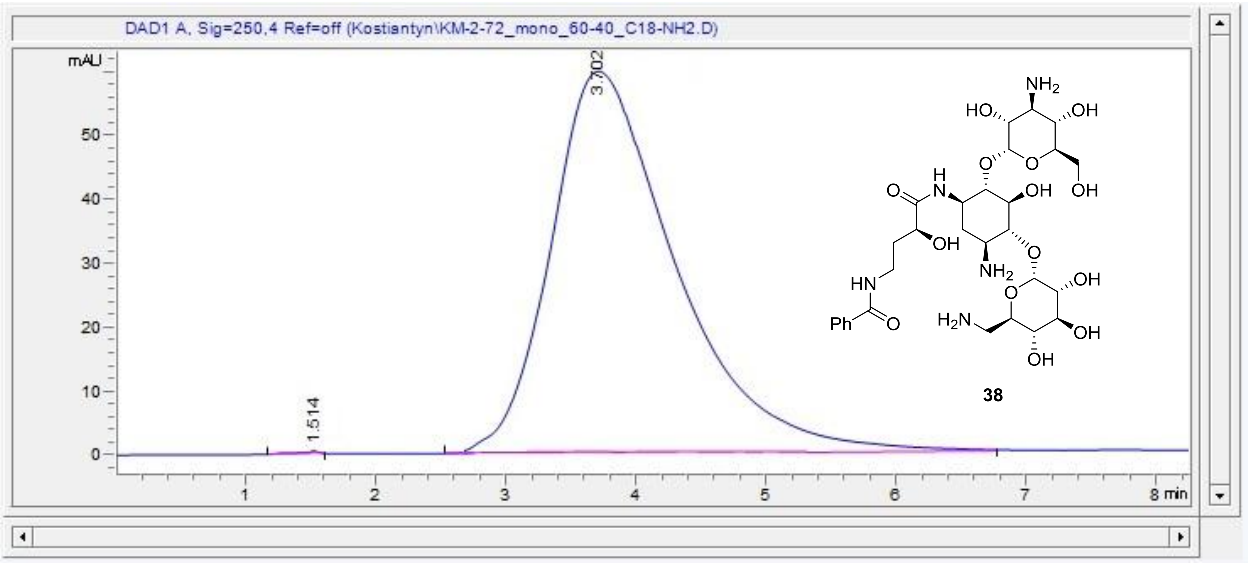

#	Time	Area	Height	Width	Area%	Symmetry
1	1.514	4.1	4.1E-1	0.1319	0.095	3.694
2	3.702	4289.3	59.6	1.1987	99.905	0.722

Lichrospher® 100 NH_2 (5µm) C18 column, flow rate 0.5 mL/min, H_2O/CH_3CN = 60:40

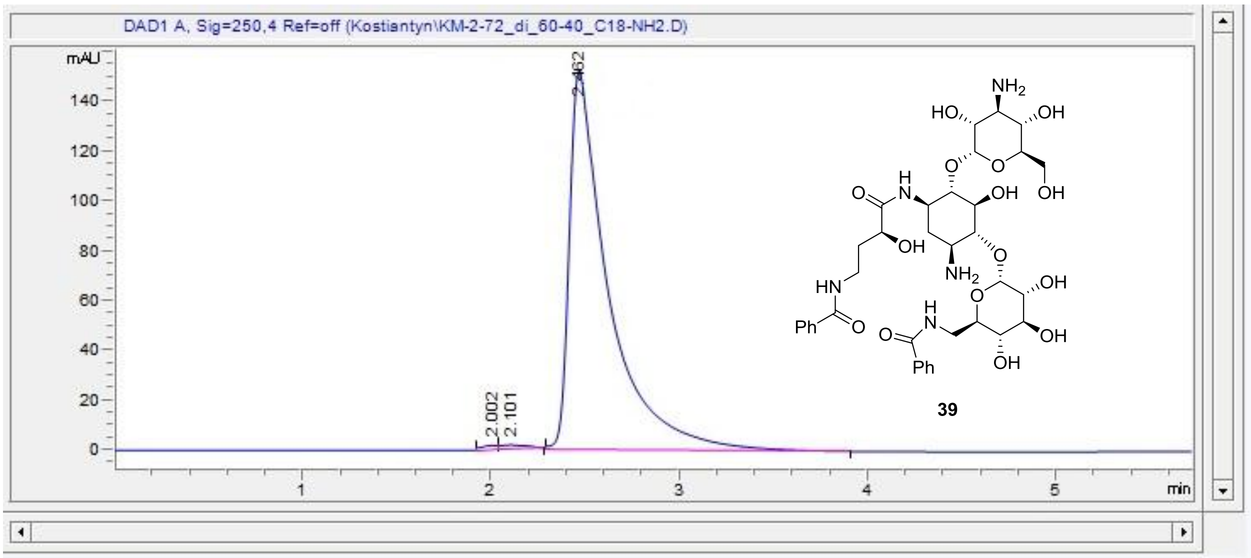

#	Time	Area	Height	Width	Area%	Symmetry
1	2.002	14.3	2.3	0.0913	0.502	2.079
2	2.101	19	2	0.1262	0.665	0.606
3	2.462	2822.7	152	0.3095	98.833	0.233

Lichrospher® 100 NH_2 (5µm) C18 column, flow rate 0.5 mL/min, H_2O/CH_3CN = 70:30

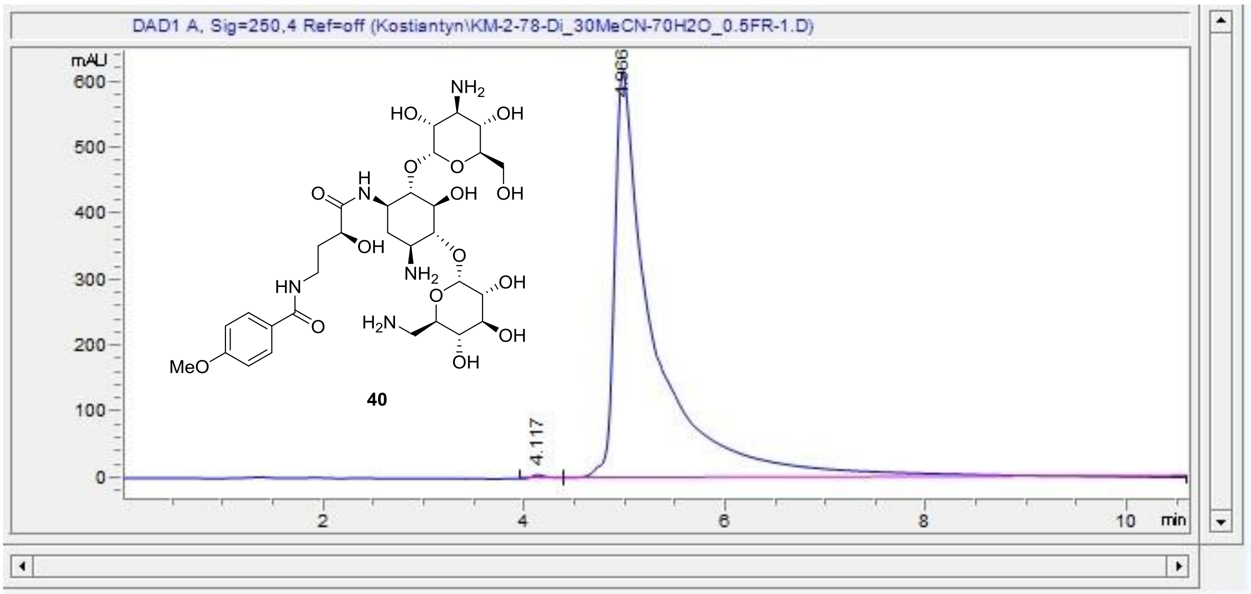

#	Time	Area	Height	Width	Area%	Symmetry
1	4.117	49.8	5.4	0.1373	0.283	0.645
2	4.966	17552.2	616.2	0.3783	99.717	0.226

Lichrospher® 100 NH_2 (5µm) C18 column, flow rate 0.5 mL/min, H_2O/CH_3CN = 70:30

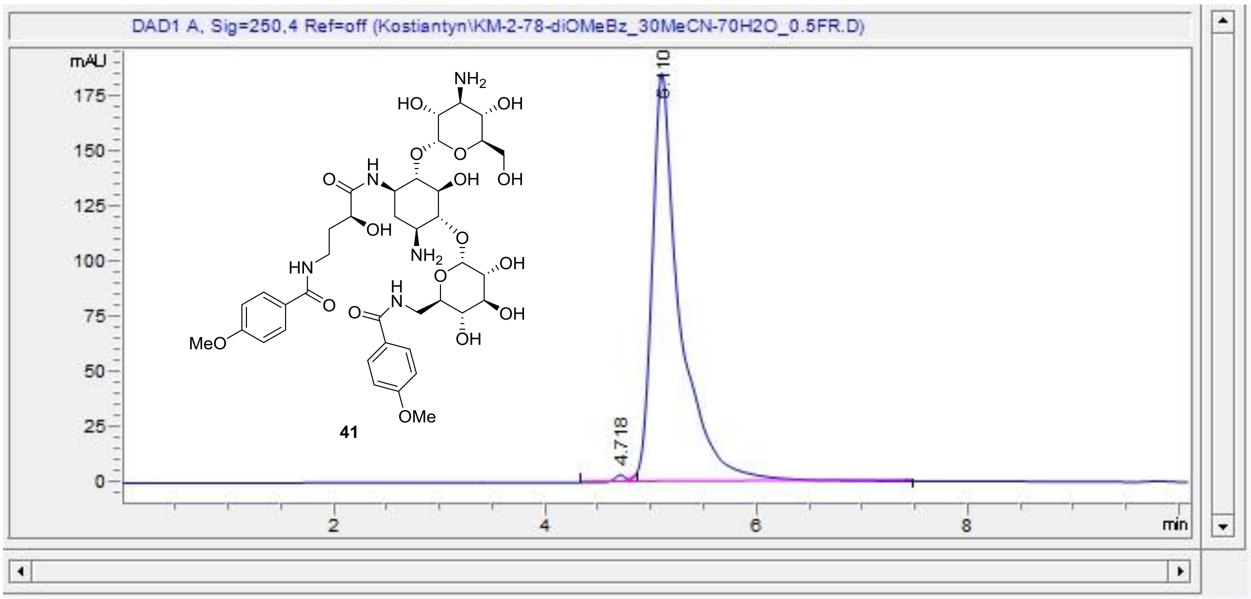

#	Time	Area	Height	Width	Area%	Symmetry
1	4.718	24	3.2	0.1102	0.693	1.067
2	5.11	3433.2	185.4	0.26	99.307	0.498

Lichrospher® 100 NH_2 (5µm) C18 column, flow rate 0.5 mL/min, H_2O/CH_3CN = 70:30

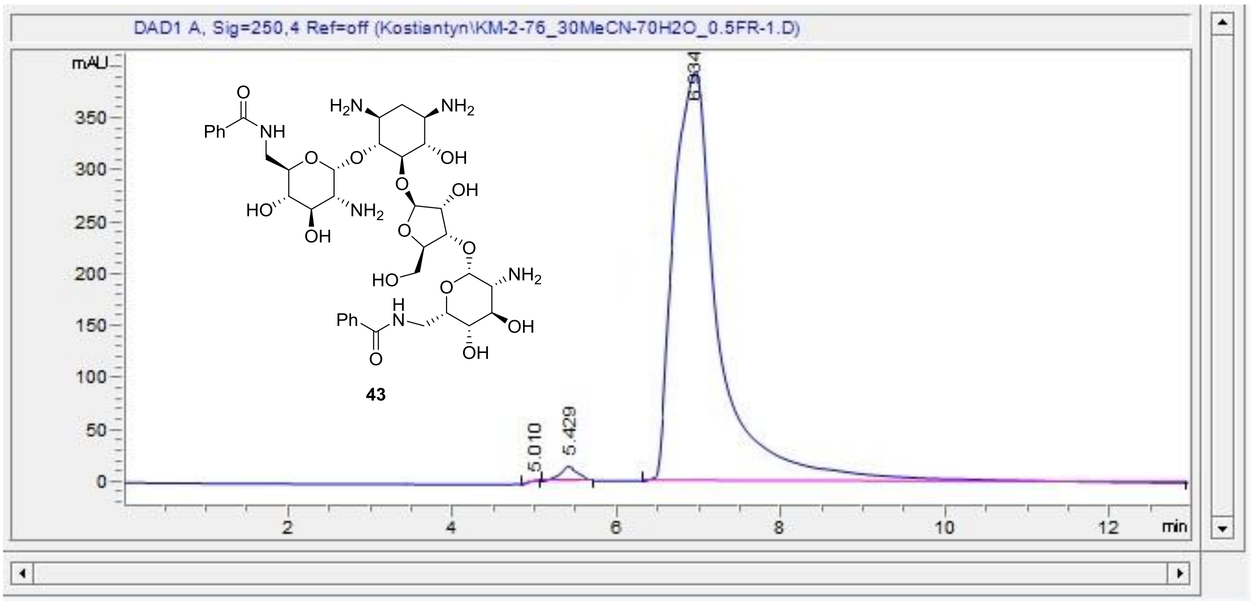

#	Time	Area	Height	Width	Area%	Symmetry
1	5.01	17.2	1.8	0.1615	0.082	3.566
2	5.429	341.6	15.9	0.2941	1.630	0.684
3	6.934	20602	393	0.6736	98.288	1.551
